# Systemic factors associated with intraocular pressure among subjects in a health examination program in Japan

**DOI:** 10.1371/journal.pone.0234042

**Published:** 2020-06-03

**Authors:** Satsuki Takahashi, Katsunori Hara, Ichiya Sano, Keiichi Onoda, Atsushi Nagai, Shuhei Yamaguchi, Masaki Tanito

**Affiliations:** 1 Department of Ophthalmology, Shimane University Faculty of Medicine, Izumo, Japan; 2 Department of Neurology, Shimane University Faculty of Medicine, Izumo, Japan; National Yang-Ming University Hospital, TAIWAN

## Abstract

**Background:**

To elucidate the possible effect of various systemic factors on intraocular pressure (IOP) using a dataset from a health examination program database in Japan.

**Methods:**

This cross-sectional study included 1569 subjects selected from the 2287 subjects who comprised the database. Various systemic parameters including age, sex, height, body weight, waist circumference, percent body fat, blood pressure (BP), pulse rate, body mass index, 28 blood examination values, intimal medial thicknesses of both carotid arteries, and intraocular pressure (IOP) values measured by non-contact tonometry in both eyes were collected. The possible correlation between the IOP and other parameters was assessed initially by univariate analyses followed by multivariate analyses.

**Results:**

Stepwise multivariate analyses, which included all parameters extracted by the univariate analyses (p<0.1) and sex, identified the same six parameters as indicators of the IOP values for each right and left IOP model. Among the parameters, age (r = -0.05 and -0.04/year for right and left IOPs, respectively) was associated negatively and the percent body fat (r = 0.06 and 0.05/%), systolic BP (r = 0.02 and 0.03/mmHg), pulse rate (r = 0.03 and 0.03/counts/minutes), albumin (r = 1.12 and 1.00/g/dL), and hemoglobin A1c (r = 0.38 and 0.44/%) were associated positively with the IOP in each eye.

**Conclusions:**

Older age was associated with low IOP, while factors reflecting the metabolic syndrome were associated with high IOP in our study population.

## Introduction

Intraocular pressure (IOP) is the only treatable risk factor of glaucoma, which is one of the world’s leading causes of irreversible blindness [1]. IOP is maintained primarily by the balance between the rates of aqueous humor production by the ciliary body and aqueous outflow via Schlemm’s canal and uveoscleral tissues that drain into the veins; thus, the episcleral vein and intraorbital tissue pressures are involved in the maintenance of IOP [2]. Previous studies have suggested that some local factors, such as resistance of the trabecular meshwork and juxtacanalicular connective tissues, are the major determinants of IOP [3,4]. However, the exogenous effects on the glaucoma [5,6] or IOP [7] by various systemic factors, lifestyle, and diet also have been suggested recently to be contributors.

The current cross-sectional study evaluated the possible effects of the various systemic factors on the IOP using a dataset from a health examination program in Japan.

## Methods

### Subjects

Institutional Review Board of the Shimane University Faculty of Medicine approved this study (IRB No. 20160217–1), which was conducted according to the tenets of the Declaration of Helsinki. Each participant provided written consent. The cohort database included 2287 Japanese subjects who participated in a health examination system in the Shimane Institute of Health Science [8, 9] from April 20, 2005, to February 13, 2013. We chose 1569 subjects from the database who had a complete dataset of the following parameter values including age, sex, height, waist circumference, percent body fat, systolic blood pressure (sBP), diastolic blood pressure (dBP), and mean blood pressure, pulse rate, body mass index (BMI), percent body fat, 28 blood examination values, intimal medial thickness (IMT) of both carotid arteries, and IOP values in both eyes. The BMI was calculated as the body weight (kg) divided by the square of the body height (m). The percent body fat was measured using a body fat scale (Tanita Body Composition Analyzer TBF-215, Tanita Corporation, Tokyo, Japan) based on a bioelectrical impedance analysis method that measures the electrical resistance of the body (bioelectrical impedance) to estimate body fat composition (http://pro.tanita.co.jp/tech/tn01.html). The blood examination included measurement of the brain natriuretic peptide (BNP), total protein (TP), albumin, albumin/globulin ratio (A/G), bilirubin, aspartate aminotransferase, alanine aminotransferase (ALT), guanosine triphosphate, alkaline phosphatase, total cholesterol, triglyceride, high-density lipoprotein (HDL) cholesterol, low-density lipoprotein (LDL) cholesterol, hemoglobin A1c (HbA1c), white blood cell count, red blood cell count, hemoglobin, hematocrit, platelet count, fibrinogen, blood urea nitrogen (BUN), creatinine, sodium, potassium, chlorine, calcium, uric acid, and amylase. The IMT was measured by ultrasonography (HI VISION Avius, Hitachi, Ltd., Tokyo, Japan). Experienced laboratory technicians measured the IOP using a non-contact tonometer (Full Auto Tonometer TX-F, Canon Incorporated, Tokyo, Japan). Dataset of this manuscript is shown in S1 Table.

### Statistical analysis

All data analyses were performed using JMP statistical software, version 10 (SAS Institute Japan, Tokyo, Japan) on a Macintosh personal computer. Continuous variables were expressed as the mean ± standard deviation. To assess the possible correlation between the IOP and other systemic parameters, the Spearman rank correlation coefficient was used in the univariate analyses in which either the IOP of the right or left eye served as the dependent variable and other parameters served as independent variables. For further analyses, multiple regression analyses were performed in which either the right or left IOP served as a dependent variable and all parameters with p-values less than 0.1 in the univariate analyses and dummy variables converted gender to male = 0 and female = 1 which served as independent variables; for model construction, a stepwise forward selection method with a minimal Bayesian information criterion stopping rule was chosen.

## Results

The characteristics of the 1569 study subjects (819 men, 52%; 750 women, 48%; mean age, 62.2±8.7 years; range, 27–92 years) are summarized in Table 1. The mean IOP was 12.8±3.0 mmHg (range, 7–33.1 mmHg) in the right eye and 12.8±3.0 mmHg (range, 7–33.8 mmHg) in the left eye.

**Table 1:** Subjects’ demographic data (N = 1569).

Parameters	Mean±SD	Range
Right IOP (mmHg)	12.8±3.0	7–33.1
Left IOP (mmHg)	12.8±3.0	7–33.8
Age (years)	62.2±8.7	27–92
Male/female	819	750
Height (cm)	159.6±8.9	131.3–184.3
Weight (kg)	59.5±11.4	33.5–171.6
BMI	23.2±3.1	14.6–51.1
Percent body fat (%)	25.8±6.5	8.2–51.7
Waist circumference (cm)	80.4±9.7	55.5–146
Systolic BP (mmHg)	130.1±17.4	85–195
Diastolic BP (mmHg)	74.1±11.7	41–115
Mean BP (mmHg)	92.8±12.8	60–140.3
Pulse rate (counts/min)	65.3±11.1	38–141
BNP (pg/ml)	23.4±36.4	2–663
TP (g/dl)	7.4±0.4	4.6–9
Albumin (g/dl)	4.4±0.2	2.6–5.2
A/G	1.5±0.2	0.9–4.6
Total bilirubin (mg/dl)	0.8±0.3	0.2–2.5
AST (IU/l)	25.2±9.6	12–114
ALT (IU/l)	23.8±15.0	1–163
γGTP (IU/l)	42.6±49.8	1–875
ALP (IU/l)	227.4±68.7	71–929
Total cholesterol (mg/dl)	210.1±31.0	112–327
Triglycerides (mg/dl)	119.5±76.0	28–801
HDL-C (mg/dl)	62.4±16.3	5–177
LDL-C (mg/dl)	122.0±29.3	13–243.8
HbA1c (%)	5.5±0.7	3.8–11
WBC (x10^2^/μl)	59.0±15.5	25.5–151.9
RBC (x10^4^/μl)	460.7±41.8	294–610
Hemoglobin (g/dl)	14.3±1.4	4.5–19.3
Hematocrit (%)	42.9±3.6	28.2–58.2
Platelet (x10^4^/μl)	22.5±5.3	5.2–56.3
Fibrinogen (mg/dl)	289.1±56.5	106–663
BUN (mg/dl)	14.8±3.7	6.4–60.4
Creatinine (mg/dl)	0.7±0.2	0.4–3.6
Na (mEq/l)	142.3±1.9	130–148
K (mEq/l)	4.2±0.3	3.2–5.6
Cl (mEq/l)	103.4±2.4	89–111
Ca (mg/dl)	9.3±0.3	7.9–11.6
Uric acid (mg/dl)	5.3±1.3	0.8–9.4
Amylase (IU/l)	81.7±27.7	24–297
Right IMT (mm)	0.8±0.4	0–3.7
Left IMT (mm)	0.9±0.4	0–6.1

IOP, intraocular pressure; SD, standard deviation; BMI, body mass index; BP, blood pressure; BNP, brain natriuretic peptide; TP, total protein; A/G, albumin/globulin; AST, aspartate aminotransferase; ALT, alanine aminotransferase; γGTP, guanosine triphosphate; ALP, alkaline phosphatase; HDL-C, high-density lipoprotein cholesterol; LDL-C, low-density lipoprotein cholesterol; HbA1c, glycosylated hemoglobin A1c; WBC, white blood cell; RBC, red blood cell; BUN, blood urea nitrogen; Na, sodium; k, potassium; Cl, chlorine; Ca, calcium; IMT, intimal-medial thickness.

During screening analyses, the associations between various systemic parameters and the IOP were analyzed by univariate analyses (Table 2). Among the parameters, possible associations with the IOP (p<0.1) were found in age, height, BMI, percent body fat, waist circumference, sBP, dBP, mean BP, pulse rate, TP, albumin, alanine aminotransferase, guanosine triphosphate, triglycerides, HbA1c, white blood cell count, red blood cell count, hemoglobin, hematocrit, platelet count, creatinine, calcium, and amylase in both the right and left eyes. The BNP, albumin/globulin ratio, total cholesterol, LDL cholesterol, and both the right and left IMTs were possible correlations only for the right eyes. The waist circumference, BUN, and chlorine were possible correlations only for the left eyes. Of these parameters, the age, height, BNP, BUN, creatinine, chlorine, amylase, and both the right and left IMTs were correlated negatively with the IOP, while others were correlated positively with the IOP. Both the right and left IOPs of women (mean, 13.0±2.8 mmHg for the right eyes; mean, 13.0±2.8 mmHg for left eyes) were slightly higher than those of the men (mean, 12.6±3.1 mmHg for the right eyes; mean, 12.6±3.1 mmHg for the left eyes) (p = 0.0048 for the right eyes and p = 0.0304 for the left eyes, Student t-test). Possible associations between each pair of parameters other than IOPs are shown in S2 Table.

**Table 2:** Univariate analyses for possible correlations between IOP and various systemic parameters.

	Right IOP		Left IOP	
Age (years)	r = -0.1087	p = 0.0001*	r = -0.0890	p = 0.0004*
Height (cm)	-0.0656	0.0094*	-0.0655	0.0095*
Weight (kg)	0.0214	0.3961	0.0325	0.1976
BMI	0.0937	0.0002*	0.1026	<0.0001*
Percent body fat (%)	0.1846	<0.0001*	0.1679	<0.0001*
Waist circumference (cm)	0.0638	0.0115*	0.0703	0.0054*
Systolic BP (mmHg)	0.1166	<0.0001*	0.1521	<0.0001*
Diastolic BP (mmHg)	0.0918	0.0003*	0.1078	<0.0001*
Mean BP (mmHg)	0.1064	<0.0001*	0.1327	<0.0001*
Pulse rate (counts/min)	0.1282	<0.0001*	0.1156	<0.0001*
BNP (pg/ml)	-0.0619	0.0141*	-0.0407	0.1069
TP (g/dl)	0.1110	<0.0001*	0.1059	<0.0001*
Albumin (g/dl)	0.1379	<0.0001*	0.1353	<0.0001*
A/G	0.0434	0.0855*	0.0381	0.1315
Total bilirubin (mg/dl)	-0.0006	0.9818	-0.0040	0.8728
AST (IU/l)	0.0108	0.6679	0.0318	0.2076
ALT (IU/l)	0.0601	0.0173*	0.0832	0.0010*
γGTP(IU/l)	0.0418	0.0981*	0.0497	0.0489*
ALP (IU/l)	0.0016	0.9495	-0.0092	0.7143
Total cholesterol (mg/dl)	0.0578	0.0220*	0.0296	0.2414
Triglycerides (mg/dl)	0.1036	<0.0001*	0.0998	<0.0001*
HDL-C (mg/dl)	-0.0109	0.6671	-0.0077	0.7598
LDL-C (mg/dl)	0.0422	0.0944*	0.0000	0.9993
HbA1c (%)	0.0596	0.0182*	0.0631	0.0124*
WBC (x10^2^/μl)	0.1098	<0.0001*	0.0948	0.0002*
RBC (x10^4^/μl)	0.1092	<0.0001*	0.0943	0.0002*
Hemoglobin (g/dl)	0.0813	0.0013*	0.0827	0.0010*
Hematocrit (%)	0.0849	0.0008*	0.0780	0.0020*
Platelet count (x10^4^/μl)	0.0618	0.0143*	0.0613	0.0152*
Fibrinogen (mg/dl)	0.0169	0.5031	0.0062	0.8053
BUN (mg/dl)	-0.0370	0.1425	-0.0441	0.0805*
Creatinine (mg/dl)	-0.0793	0.0017*	-0.0701	0.0054*
Na (mEq/l)	0.0224	0.3758	0.0092	0.7168
K (mEq/l)	-0.0135	0.5937	-0.0130	0.6076
Cl (mEq/l)	-0.0324	0.1996	-0.0451	0.0744*
Ca (mg/dl)	0.0963	0.0001*	0.0900	0.0004*
Uric acid (mg/dl)	0.0050	0.8441	0.0276	0.2745
Amylase (IU/l)	-0.0512	0.0424*	-0.0528	0.0365*
Right IMT (mm)	-0.0529	0.0363*	-0.0309	0.2207
Left IMT (mm)	-0.0463	0.0665*	-0.0112	0.6563

All analyses were performed by Spearman’s rank correlation test. The asterisk (*) indicates p<0.1.

r, regression coefficient; IOP, intraocular pressure; BMI, body mass index; BP, blood pressure; BNP, brain natriuretic peptide; TP, total protein; A/G, albumin/globulin; AST, aspartate aminotransferase; ALT, alanine aminotransferase; γGTP, guanosine triphosphate; ALP, alkaline phosphatase; HDL-C, high-density lipoprotein cholesterol; LDL-C, low-density lipoprotein cholesterol; HBA1c, glycosylated hemoglobin A1c; WBC, white blood cell; RBC, red blood cell; BUN, blood urea nitrogen; Na, sodium; k, potassium; Cl, chlorine; Ca, calcium; IMT, intimal-medial thickness.

To determine the significant parameters associated with IOP, stepwise multivariate analyses that included all parameters obtained from the univariate analyses (p<0.1) and sex were performed. From the independently performed analyses for the right and left IOPs, the same six parameters, i.e., age, percent body fat, sBP, pulse rate, albumin, and HbA1c, were detected as significant parameters for both the right and left IOP models (Table 3). Among them, age was associated negatively with both the right and left IOPs, while the other five parameters were associated positively with the right and left IOPs.

**Table 3:** Multivariate analyses for possible correlations between IOP levels and various systemic parameters.

	Right IOP			Left IOP		
	r (95% CI)	p-value	Standard β	R	p-value	Standard β
Age (year)	-0.05 (-0.07–-0.03)	2.8x10^-8^	-0.14	-0.04 (-0.06–-0.03)	5.4x10^-7^	-0.13
Percent body fat (%)	0.06 (0.04–0.08)	4.8x10^-7^	0.13	0.05 (0.03–0.07)	7.5x10^-6^	0.11
Systolic BP (mmHg)	0.02 (0.01–0.03)	2.2x10^-5^	0.11	0.03 (0.02–0.03)	2.9x10^-9^	0.15
Pulse rate (counts/min)	0.03 (0.02–0.04)	1.0x10^-5^	0.11	0.03 (0.01–0.04)	2.5x10^-4^	0.09
Albumin (g/dl)	1.12 (0.47–1.78)	8.1x10^-4^	0.08	1.00 (0.35–1.66)	2.6x10^-3^	0.08
HbA1c (%)	0.38 (0.18–0.59)	2.9x10^-4^	0.09	0.44 (0.24–0.65)	2.6x10^-5^	0.10

Parameters are chosen by stepwise multivariate analyses. The analyses for right and left eyes are performed independently.

IOP, intraocular pressure; BP, blood pressure; HbA1c, hemoglobin A1c; r, regression coefficient; CI, confidence interval.

## Discussion

The current study found that percent body fat, sBP, pulse rate, albumin, and HbA1c were correlated positively with IOP, while age was correlated negatively with IOP. These associations have been detected in multivariate models for IOPs of both eyes; thus, the detection of the same six parameters in independent models strengthened our results.

Cross-sectional studies in Italy [23] and in the United States [10, 11] reported the positive association between age and IOP in general populations, while in Korea [12–15] reported a negative association, as did in Taiwan [16, 17], in China [18], and in Japan [19–21]. A longitudinal family-based cohort study also reported a negative association in Koreans and Mongolians [22]. A negative association was significant only in men from community-based studies in Korea [15] and Taiwan [17] while only in women from healthy participants of a health examination system in Korea [13]. We found a slight but significant difference in IOP between men and women (higher IOP in women), although we did not find a significant association between sex and IOP by multivariate analysis. In other studies, the distributions of the IOPs did not differ significantly between the sexes [14, 15, 19, 21], while other studies have reported a significant association between sex and IOP (higher IOP in men) [16, 23, 24]. Previously, a significant association between sex and IOP seen in univariate regression analysis was explained by the interaction of other variables such as the sex difference in insulin resistance indices, sBP, total cholesterol, and HDL cholesterol [13, 14]. Thus, relationships between IOP and age may vary according to race and sex. The IOP is determined by the balance between aqueous humor inflow and outflow. Aging may cause a decrease in aqueous humor production [25–27] and increased trabecular meshwork resistance [28]; dominance of former change than later change with aging may explain our observation. A large difference in the myopia prevalence, lifestyle, diet, and systemic factors such as obesity can explain the different trends between Western and Asian populations [22, 29].

In both Western and Asian populations, obesity or high BMI was associated with high IOP [11–13, 15–17, 19–21, 30, 31]. In the current analysis, although several obesity-related parameters such as BMI, percent body fat, waist circumference, total cholesterol, triglycerides, LDL cholesterol, and IMT possibly were associated with IOP in univariate analysis, only the percent body fat remained significant in multivariate analysis. In a Korean population-based studies, in non-glaucomatous participants, increased body fat was associated with high IOP in both men and women, while greater lean body mass was associated with lower IOP in women [32]; and in both glaucomatous and non-glaucomatous participants, increased body fat was associated with a low prevalence of primary open-angle glaucoma (POAG) (including normal tension glaucoma) in women, while increased body muscle volume was associated with high POAG prevalence in men [33]. Thus, the roles of body fat on glaucoma may differ between sexes when the fat and skeletal muscle masses were separately evaluated. Collectively, the percent body fat may be a good indicator for exploring the effects of obesity on ocular status. Obesity has been suspected to be associated with increasing IOP by sympathetic hyperactivation, increased corticosteroid, excessive intraorbital adipose tissue, increases in blood viscosity with high hemoglobin and hematocrit values, increased episcleral venous pressure, and a consequent decrease in the facility of aqueous outflow [29, 30, 34, 35]. It is well established that sympathetic hyperactivation is a common feature of obesity, whereas topical beta-adrenergic antagonist reduces IOP. Beta-adrenergic receptor polymorphism is associated with obesity, insulin resistance, IOP, and POAG [36–38]. Another study has suggested that increased IOP may be due to transitory elevations in IOP resulting from breath-holding and thorax compression while tonometry is performed during slit-lamp examinations in obese patients [39].

Previous studies have reported a significant association with IOP elevation and high sBP [17, 19, 21, 22, 31, 40, 41] or both high sBP and dBP [11, 13, 15, 16, 42]. The proposed mechanisms for the roles of BP in IOP elevation have postulated that increased BP leads to an increased filtration fraction of the aqueous humor through elevated ciliary artery pressure, and increased serum corticoids and sympathetic tone result in elevated IOP [30, 43]. Increased sympathetic tone may explain the positive association between heart rate and IOP observed in the current study. Previous studies have reported a relationship between elevated IOP and high HbA1c [11, 44, 45] or high fasting blood sugar [13, 16, 17]. Meta analyses have reported an increased glaucoma risk in diabetic patients (relative risk, 1.48) [46], while Tan et al. (2009) reported that diabetes was associated with higher IOP but persons with diabetes were not more likely to have glaucomatous optic neuropathy or POAG. The autonomic dysfunction in diabetes and the osmotic gradient induced by elevated blood glucose with a consequent fluid shift into the intraocular space may explain the association between glucose and IOP [47, 48]. The positive correlation between blood albumin and IOP that we observed was unique in the literature, although the mechanism of the association requires elucidation.

The current study had several limitations, the first of which was its cross-sectional design, which provided only epidemiologic data and did not address mechanistic analysis. Therefore, the causal relationship could not be determined. This study was based on a self-paid health examination program; and probably has selection bias. Since the participants did not undergo detailed ocular evaluations, such as slit-lamp and fundus examinations, we could not determine the prevalence of ocular diseases in the current subjects. Use of non-contact tonometry, which is generally believed to be less reliable than Goldmann applanation tonometry, and the absence of central corneal thickness measurements that were related to measurement errors during tonometry [49, 50] were other limitations. Absent of information regarding oral medications that possibly affect the IOP is another limitation of this study.

In conclusion, in a Japanese population, the members of which were enrolled in a health examination program, we determined the systemic factors associated with IOP. Other than age, the factors reflecting metabolic syndrome were found to be associated with a risk of high IOP in our population.

## Supporting information

S1 Table Dataset underlying the findings described in this manuscript. (PDF)

S2 Table Possible associations between each pair of parameters other than IOPs. (PDF)
