# Supplementary material for: Systemic factors associated with intraocular pressure among subjects in a health examination program in Japan
Source: PLoS One. 2020 Jun 3;15(6):e0234042. doi: 10.1371/journal.pone.0234042 (PMC7269229; doi:10.1371/journal.pone.0234042)
Supplement: S1 Table — (PDF) [file pone.0234042.s001.pdf]

Table S1. Dataset

Age	Sex	Height	BW	BMI	%Fat	Waist	SBP	DBP	MBP	Pulse	BNP	TP	Alb	A/G	Tbil	AST	ALT	γGTP	Alp	TC	TG	HDL	LDL	HbA1c	WBC	RBC	Hb	Ht	Plt	Fib	BUN	Cre	Na	K	Cl	Ca	UA	Amy	RIOP	LIOP	RIMT	LIMT
73	M	168.9	56.2	19.7	20.7	82	120	60	80	73	5.5	7.4	4.4	1.5	0.4	16	13	23	251	147	41	43	95.8	7.6	66.4	451	14.1	42.1	19	221	22.4	0.85	143	3.9	107	9.1	5	65	12.4	9.2	0.7	0.7
71	F	147.9	51.3	23.45	32.1	74	129	70	89.67	71	21.6	7.3	4	1.2	0.4	52	51	61	330	256	108	69	165.4	5.8	62.1	401	12.3	35.9	22.3	303	13.3	0.46	144	3.6	108	8.8	4.1	73	13.5	14.5	0.7	0.7
85	M	161.9	43	16.4	11.7	70	121	69	86.33	64	19.1	8.3	4.4	1.1	0.5	23	10	24	186	173	53	75	87.4	5.1	64.5	441	14.6	42.8	21.6	341	15.9	0.58	140	4.6	101	9.8	5.3	117	11.1	8.7	0.8	0.8
72	M	158.3	62.9	25.1	24.7	89	118	60	79.33	54	7.9	8.2	4.6	1.3	0.8	39	50	72	287	235	109	63	150.2	6.5	49.7	466	14.6	43.2	25.1	308	9.2	0.77	144	3.4	104	9	7.8	47	10.1	10.5	0.6	0.6
55	F	152.3	52.5	22.63	31.1	75	104	56	72	55	7	7.7	4.5	1.4	0.7	29	41	26	285	304	107	65	217.6	6	44.7	468	14.8	43.5	19.7	318	11	0.53	143	3.9	105	9.2	4.2	63	12.9	11	0.8	0.6
64	M	168.2	66.3	23.43	23.1	83	115	59	77.67	51	19.9	6.9	4.3	1.7	0.8	23	21	43	194	177	116	47	106.8	10.4	67.3	412	13.2	39	21.6	335	18.1	0.8	140	4.4	106	9	6.3	55	16.5	19.4	0.7	0.6
70	M	164.7	61.6	22.71	20.8	84	147	66	93	81	29.2	7.3	4.5	1.6	0.9	23	18	50	154	203	109	57	124.2	5.6	71.3	450	14.9	44.8	23.8	261	19.9	0.67	143	4.3	106	9.1	6.5	56	7.7	7.8	0.7	1.1
69	F	159	60.2	23.81	33.8	81	157	81	106.3	62	53	7.6	4.1	1.2	0.7	22	18	20	183	201	135	61	113	5.1	77.3	421	13	39.4	33	272	16	0.57	143	4.1	107	9.4	4.3	81	10.4	11	1.1	1
57	M	173.2	66.3	22.1	19.1	86	134	79	97.33	54	17.8	6.6	4.4	2	1	23	19	28	245	209	139	50	131.2	5.2	61.9	483	15.1	44.7	18.7	279	11.9	0.87	147	4.2	106	9.4	7.2	85	7.9	7.7	0.7	0.6
73	M	165.7	59	21.49	21.4	88.5	135	81	99	76	3	7.6	4.7	1.6	0.7	32	61	47	246	227	126	46	155.8	5.5	47.2	466	15.5	46.7	28.7	283	12.3	0.88	144	4.4	105	9.7	5.6	97	11.9	20.1	0.8	0.9
49	M	174.1	68.6	22.63	21	89	113	60	77.67	52	3.2	6.3	4.2	2	0.7	18	23	48	317	213	89	46	149.2	5.3	57.5	464	15	45.4	23.6	279	11.5	0.71	147	4.1	110	8.9	4.3	126	8.3	10.6	0.6	0.5
54	M	174.9	89	29.09	29.1	101	105	60	75	71	12	7	4.6	1.9	0.6	23	28	115	258	217	169	46	137.2	6.2	62	492	16.2	48.6	21.9	226	15.4	0.67	144	4.4	104	9.1	4.7	34	11	10.5	0.5	0.6
57	M	173.3	66.8	22.24	18.8	82	119	69	85.67	59	18.4	7.3	4.4	1.5	0.8	21	13	41	243	236	68	72	150.4	5.8	42.6	394	13.9	42.1	23.3	414	19.4	0.81	146	4.7	109	9.1	6.6	58	18.8	15.3	0.8	0.7
58	M	167.7	75.8	26.95	24.2	96.5	125	76	92.33	57	31.8	6.8	4.2	1.6	0.4	17	13	66	280	238	162	53	152.6	5.8	93.3	438	14.2	42	28.7	296	12.5	0.71	145	4.5	108	9.1	6.5	50	12.5	11.3	0.7	0.8
68	F	149.1	49.1	22.09	26.7	71	96	51	66	68	13.7	7	4.3	1.6	0.5	21	13	13	235	216	81	76	123.8	4.9	49.4	405	12.9	39.6	24.1	320	17.5	0.48	146	4.2	107	9.1	3.9	86	14.4	14.9	0.7	0.7
64	F	155.2	57	23.66	31.2	76	125	66	85.67	64	32.2	7.2	4.3	1.5	0.9	25	19	28	202	250	81	80	153.8	4.8	44.8	394	12.4	37.8	20.9	247	17.2	0.51	145	4.3	108	9	3.1	49	14.1	12.7	0.9	0.8
61	F	163.3	63.3	23.74	33.8	76	132	64	86.67	62	10.1	7.2	4.7	1.9	0.9	24	27	38	214	249	105	60	168	5.3	58.9	415	13.1	38.5	22.9	275	18.4	0.52	143	4.1	107	9.6	4.6	57	16.5	15.2	0.5	0.5
79	M	163.3	72.4	27.15	26.3	97.5	117	62	80.33	72	41.1	7.3	4.4	1.5	1	26	31	67	173	223	86	62	143.8	4.7	58.1	430	14.1	42	14.1	226	15.7	0.51	142	4.1	101	9	4.7	41	9.8	8.5	0.6	1
54	F	154.5	52.3	21.91	27.8	75	120	72	88	73	15.4	7.1	4.2	1.4	0.7	16	19	17	218	245	168	64	147.4	4.9	50.7	395	12.4	37.9	24.8	301	11	0.58	141	3.9	104	9.1	3.6	135	9.7	10.4	0.5	0.6
40	M	165.9	86.2	31.32	29.7	99	142	80	100.7	53	2.5	7.5	4.7	1.7	0.9	55	113	112	162	240	481	41	102.8	5.1	63.2	521	15.6	45	26.6	219	10.7	0.8	143	4	105	9.5	5.1	45	15.8	17.4	0.7	0.7
39	F	157.4	71.6	28.9	45.2	86	116	50	72	62	2.7	7.8	4.4	1.3	0.5	22	24	33	174	196	58	51	133.4	5.6	64.4	462	14.2	42	22.4	301	14.7	0.64	140	4.5	105	9.3	7.2	61	11.7	13.3	0.5	0.6
58	M	171	66.5	22.74	20.6	83.5	127	76	93	79	4.8	7.5	4.7	1.7	0.5	26	24	31	219	242	70	84	144	5.2	65.4	472	15.7	45.6	23.3	394	15.3	0.8	141	4.6	107	9.4	8.7	68	12.8	13	0.8	0.7
64	M	162.9	73.2	27.58	30.4	95	132	66	88	62	8.9	7.4	4.3	1.4	0.6	21	27	41	290	232	139	67	137.2	8.5	74	480	15.6	45.4	33.6	331	17.1	0.73	141	4.5	105	9.4	5	52	13.1	14	0.9	1.3
58	M	176.5	65.8	21.12	24.4	86.5	109	56	73.67	49	15.1	7.7	4.2	1.2	1	18	18	35	170	190	71	62	113.8	4.9	75	428	13.7	41.5	26.2	250	17	0.53	141	4.1	105	9.3	5.4	68	13.9	13.1	0.8	0.8
55	F	152.5	62.8	27	43.4	86	128	62	84	58	5.8	7.4	4.4	1.5	0.7	20	24	56	253	234	206	58	134.8	5.6	49.5	445	13.3	40.5	30.4	271	15.2	0.67	142	4.5	105	9.7	4.9	68	17.5	16.7	0.6	0.6
62	F	157.3	49.7	20.09	26.5	71	96	60	72	62	10.6	7.3	4.3	1.4	0.6	40	40	175	480	262	91	68	175.8	5.1	91.3	437	13.6	41.8	27.9	349	14.5	0.75	143	4.1	105	9.5	5.6	89	13.9	12	0.7	0.8
50	F	158.1	50.3	20.12	28.6	70.5	111	64	79.67	68	4.7	8	4	1	0.3	20	12	29	247	180	80	70	94	5.2	91.9	444	9.6	32.3	56.3	279	15.6	0.47	140	4.5	103	8.7	4	51	15.8	12.9	0.5	0.6
46	M	173.2	67.6	22.53	21.4	82	120	72	88	55	18.2	7.4	4.2	1.3	0.4	21	11	18	192	168	161	41	94.8	5.9	51.9	408	14.3	42.1	23.3	301	21.5	0.94	146	4.5	108	8.9	7.6	45	9.1	12.8	0.9	0.7
58	M	165.1	68.8	25.24	24.4	85	159	90	113	60	11.2	6.6	4.2	1.8	0.8	16	13	23	168	162	88	37	107.4	5.1	51.2	471	15.4	44.5	18.1	279	12.1	0.85	141	4.2	107	9.2	5.4	56	9.9	9.7	0.6	0.5
52	M	162.4	54.1	20.51	14.6	71	109	66	80.33	54	14.5	6.6	4.1	1.6	0.8	20	14	23	109	192	76	66	110.8	5.3	61.6	395	12.8	37.8	20.2	233	21.9	0.92	146	3.9	110	9.3	5.4	145	10.4	11.4	0.5	0.5
52	M	170.6	59.3	20.37	19	71	124	62	82.67	52	8.4	7.5	4.5	1.5	1.1	17	11	17	148	157	44	58	90.2	4.7	40.1	457	14.6	42.1	24.5	184	14.7	0.81	143	3.8	106	9.1	6.1	73	9.4	9.2	0.5	0.5
50	M	167.8	73.7	26.17	24.5	86	157	96	116.3	61	2	7.6	4.6	1.5	0.7	36	58	47	183	190	154	31	128.2	5	61.6	533	17.6	50.5	23.7	196	16.6	1.12	143	4.2	104	9.6	6.6	73	16.7	13.6	0.8	0.9
49	M	180.1	86.2	26.58	23.3	88	128	65	86	55	8.8	7.5	4.4	1.4	0.7	22	28	86	163	233	189	60	135.2	4.9	50.4	397	13.7	38.9	32.3	244	15.4	0.8	143	4.1	105	9.1	6.4	46	17.5	17.2	0.7	0.7
68	M	162.1	48.5	18.46	14.4	69	122	75	90.67	63	10.6	7.2	4.5	1.7	1.6	28	17	66	186	195	37	73	114.6	4.9	38.6	475	15.6	46.2	29.6	296	16.4	0.58	144	4.4	105	9.4	3.3	94	13	11	0.5	0.7
57	M	169.7	71.2	24.72	24	87.5	155	76	102.3	44	12	7.4	4.3	1.4	1.5	22	17	22	164	229	100	72	137	5.2	60.9	423	13.8	41	30.3	244	24.2	0.76	144	4.4	106	9.5	5.8	44	11.5	8.8	0.5	0.7
61	F	149	51.3	23.11	26.7	61	158	79	105.3	59	40.9	7.8	4.7	1.5	0.7	29	19	33	188	252	64	81	158.2	5.3	55.6	425	12.8	38.2	29.1	320	13.9	0.74	144	4.1	104	10	4.9	82	12.7	12.2	0.6	0.6
65	F	149.1	48.7	21.91	27.3	70	110	66	80.67	65	6.8	7.6	4.4	1.4	1.1	19	15	29	143	244	150	50	164	5.1	43.3	445	14	41.5	17.9	279	16.4	0.6	143	4.1	107	9.5	5.2	93	22.2	19.9	0.7	0.6
63	F	156.9	55.8	22.67	28.8	72	114	66	82	76	3.6	7.2	4.3	1.5	0.7	28	27	15	169	234	83	45	172.4	5.3	53.1	429	13.5	39.8	25.3	315	14.2	0.59	144	3.9	107	9.3	4.9	68	15.9	14.6	0.4	0.4
53	M	170.5	83.4	28.69	28.5	94.5	122	56	78	57	11.4	6.8	4.2	1.6	0.6	17	22	36	230	174	208	35	97.4	5.3	83.4	503	15.1	44.3	30.4	414	14.8	0.92	143	4.2	106	9.3	6.3	72	18.5	17.2	0.6	0.9
55	M	172.5	67.2	22.58	26.4	85	133	81	98.33	63	3	8.3	4.7	1.3	0.5	20	16	65	243	180	223	43	92.4	4.4	57.3	440	14.4	41.7	24.5	253	18.1	0.88	142	4.5	105	9.2	6.5	66	16.1	14.4	0.7	0.8
42	M	169.3	92.2	32.17	34.8	103	140	92	108	64	2	7.7	4.6	1.5	0.9	33	38	137	189	235	145	66	140	6.2	57	475	15.9	46.3	24	283	14.4	0.96	144	3.9	103	9.4	8.7	85	18.2	17.1	0.8	0.6
55	F	152.8	59.5	25.48	34.1	74	120	77	91.33	61	23.9	7	4.3	1.6	1	18	21	20	235	130	119	51	55.2	5.3	59.2	486	15	44.6	24.5	310	13.5	0.61	145	4	109	9.8	5.4	83	14.6	13	0.6	0.7
57	M	165.3	68.7	25.14	25.8	87	106	59	74.67	61	4.1	6.8	4.4	1.8	0.3	15	15	47	309	233	197	41	152.6	5.3	100.8	438	15	44.8	30.7	361	16.3	1	143	4.6	105	9.3	6.1	54	8.3	10	0.7	0.7
59	M	168.8	62.7	22.01	19.3	77	114	58	76.67	49	19.5	6.6	4.2	1.8	0.7	14	13	38	219	208	105	46	141	5.1	73.4	472	14.9	43.8	28.3	241	12.2	0.96	143	4.5	106	9.1	8	94	15.2	13.2	0.7	0.5
49	M	169	79.1	27.7	27.6	88	132	86	101.3	77	145	7.3	4.7	1.8	0.3	23	33	53	204	209	116	50	135.8	5.3	88.5	508	15.8	45.8	27.2	253	17.8	0.89	144	3.8	104	9.3	6.9	60	13.8	12.4	0.8	1
54	F	158.9	67.9	26.89	34.4	80	130	74	92.67	62	4.2	7.2	4.5	1.7	1.1	20	33	23	178	231	83	69	145.4	5.1	51.1	469	14.4	41.8	29.2	219	14.8	0.61	144	4.1	106	9.8	6.1	36	14.4	17.4	0.4	0.6
56	M	166.8	69.7	25.05	20.7	86	143	84	103.7	53	2.7	6.9	4.6	2	1	27	22	37	228	211	108	51	138.4	5	45	481	15.9	46.3	17.5	206	12.5	0.87	143	4	106	9.1	6.4	71	12.9	13.3	0.6	0.6
53	M	178.5	73.6	23.1	22.5	84	119	68	85	54	11.9	6.8	4.4	1.8	0.8	14	8	20	173	174	64	62	99.2	6.1	82	439	14.6	42.7	28.1	275	13.3	0.64	141	4	102	9.2	4.6	58	11.5	12.2	0.4	0.5
62	M	166.4	56.4	20.37	18.2	79	125	65	85	67	6.7	6.9	4.5	1.9	0.6	20	17	19	146	166	152	40	95.6	5.2	70	497	16.2	47.3	23.1	315	9.3	0.74	140	4.4	103	9.2	3.2	59	10.3	9.4	0.6	0.6
63	F	149.4	55.5	24.87	32	79	114	61	78.67	60	8.4	7	4.4	1.7	0.6	16	15	26	198	202	115	62	117	5.1	58.4	422	13.3	38.7	19.9	271	13.8	0.64	144	3.8	107	9.5	5.1	47	10.8	10.5	0.9	0.6
68	M	159.3	53.4	21.04	17.9	76.5	135	68	90.33	52	24.6	7.1	4.4	1.6	0.8	19	13	24	242	156	61	56	87.8	4.9	35	419	13.8	42.6	17.4	216	16.4	0.91	144	4.2	106	8.9	5.3	81	17	16.9	0.9	0.8
58	M	171.5	56.3	19.14	18.2	67	105	63	77	40	6.9	6.7	4.3	1.8	0.9	16	17	33	182	262	92	92	151.6	5.7	44.5	453	16	46.8	19.4	296	10.9	0.94	142	4.6	105	9.5	5.9	64	9.1	8.5	0.6	0.4
58	F	148.1	49.4	22.52	30.1	69	132	67	88.67	72	44.5	7.1	4.4	1.6	0.6	21	14	13	197	270	90	74	178	5.3	51.9	534	14	44	18.8	236	12.1	0.65	145	3.7	106	9.3	4.1	99	12.8	13.1	0.5	0.5
63	F	155.6	52.4	21.64	27.9	66.5	135	72	93	57	43.7	7.1	4.4	1.6	0.9	20	15	17	119	209	79	65	128.2	5.4	32.7	422	13.4	40.2	18.6	267	21.4	0.61	145	4.4	108	9.2	5.2	91	10.2	11.1	0.6	0.6
62	M	158.1	55.4	22.16	17.7	71	107	61	76.33	51	10.1	7.1	4.6	1.8	0.7	24	22	17	143	209	44	88	112.2	5.9	36.4	432	13.9	41	19.6	343	20.4	0.8	142	4.7	104	9.9	5.5	86	15.3	16.7	0.6	0.8
60	F	156.9	71.9	29.21	36.5	91	152	88	109.3	58	11.8	7.8	4.4	1.3	1.1	51	82	68	308	216	155	40	145	8.2	67.9	488	14.4	43.9	33.9	326	16.3	0.44	142	4.6	103	9.3	4.3	29	10.4	10.7	0.7	0.7
63	F	147.3	51.7	23.83	30.7	72	139	81	100.3	61	10.8	7.6	4.6	1.5	0.5	21	13	13	220	228	111	48	157.8	5.6	85.1	415	12.5	37.7	20.4	343	17.4	0.78	141	4.7	104	9.4	4.4	55	11.8	13.8	0.6	0.6

62	F	161.1	54.7	21.08	26.7	77	146	71	96	58	3.5	7.1	4.5	1.7	0.4	19	19	26	212	226	108	59	145.4	5.4	46.6	465	14.2	43	25	349	11.3	0.48	143	4	104	9.2	4.4	79	10.4	10.8	0.9	1
50	F	152	54.5	23.59	30.7	67	105	59	74.33	66	14	7.8	4.5	1.4	0.9	16	13	15	242	200	57	77	111.6	4.8	51	443	13.1	38.8	33.9	361	14.1	0.63	143	4.1	107	9.6	4.9	69	14.2	13.8	0.5	0.5
57	M	172.3	76.4	25.73	18.8	82	114	76	88.67	55	33.6	7.3	4.3	1.4	0.9	20	22	27	199	228	77	56	156.6	5.1	56.7	454	14.7	43.7	26.9	320	19.2	0.87	143	4.2	108	9.5	5.7	91	13	11.1	0.6	0.6
53	M	169	71.3	24.96	23.2	83	124	70	88	51	10.7	6.9	4.3	1.7	1.2	24	27	69	243	232	105	51	160	5.7	56	457	15	43.5	33.6	355	10.4	0.83	141	4.1	104	9.1	5.6	80	14.8	12.1	0.6	0.5
54	M	164.5	63.1	23.32	21.1	78	125	76	92.33	57	5.1	7.3	4.3	1.4	0.6	18	13	25	166	148	57	48	88.6	6.5	77.1	433	14.8	43	20.4	275	14.1	0.67	144	4.3	108	9.3	5.9	67	11.8	15.8	0.6	0.6
58	M	160.5	59.4	23.06	22.4	78	106	54	71.33	49	15.4	6.6	4.2	1.8	0.5	26	26	48	160	178	123	51	102.4	6.2	84.2	435	14.7	42.3	28.9	250	10.3	0.86	143	4.4	104	9.7	6.2	97	15.5	12.5	0.6	0.7
59	F	157.3	55.5	22.43	26.8	70	122	65	84	68	30.2	7.8	4.6	1.4	0.7	24	22	57	304	234	73	84	135.4	4.8	42.6	399	12.3	35.6	31.3	296	8.3	0.5	143	3.9	106	10.3	5.2	105	17.5	17.7	0.6	0.5
67	M	170	64.8	22.42	22.9	84.5	123	75	91	65	18.8	7.3	4.5	1.6	1.2	17	12	18	177	222	90	47	157	4.7	58.5	441	14.5	41.3	19.9	320	23.4	0.88	145	4.1	108	9.4	4.7	103	11.6	13.2	0.5	0.7
64	F	151.3	49.8	21.75	26.4	65	100	58	72	65	46.2	7.9	4.5	1.3	0.8	23	13	19	164	269	93	78	172.4	5.2	59.9	411	12.5	36.8	20.4	326	15.4	0.65	142	3.8	104	9.3	4.5	65	13.5	13.7	0.6	0.7
58	M	164.3	63.4	23.49	26.5	84	142	92	108.7	65	15.5	7.4	4.5	1.6	0.6	17	21	110	245	243	148	68	145.4	4.9	97.5	444	15.2	46	29.9	483	13.8	0.9	144	4.5	107	9.7	4.8	72	9.8	8.3	0.7	0.7
61	M	165.7	70.1	25.53	25.9	90	130	74	92.67	63	11.4	7.8	4.7	1.5	0.6	24	19	43	157	214	75	78	121	5.5	50.1	444	14.7	42.2	22.8	198	10.9	0.77	138	4.2	99	8.8	5.3	91	10.9	11.7	0.4	0.6
61	F	163.2	54.4	20.42	21.2	68	121	60	80.33	61	41	7.4	4.2	1.3	0.7	36	35	54	210	228	132	56	145.6	4.6	41.5	446	14.2	42.8	26.8	310	10.8	0.74	146	4	106	9.1	6.1	46	9.2	8.5	0.7	0.7
65	F	148.1	38.3	17.46	17.5	60.5	139	79	99	57	21.7	7	4.4	1.7	0.5	32	29	45	214	211	55	83	117	5.5	44.9	391	12.6	37.4	17.8	355	12.9	0.44	145	3.9	106	9.1	4.1	67	12.5	10.8	0.8	0.9
53	M	161.3	76.7	29.48	33.5	89	128	56	80	54	6.6	7.2	4.4	1.6	0.8	20	14	23	201	175	98	41	114.4	4.7	65	516	17.2	48.6	24	355	18.6	0.67	145	4	105	8.8	6.3	66	17.3	18	0.5	0.5
63	M	162.5	65.8	24.92	21.6	82	105	61	75.67	48	7.3	8	4.7	1.4	0.8	25	25	36	238	178	161	49	96.8	5.2	59.8	531	15.2	44.7	24.5	305	20.5	0.7	143	4.2	104	9.1	5.7	97	11.7	16.8	0.6	0.7
57	F	155.7	50.4	20.79	29.9	71	105	54	71	68	7	7.2	4.4	1.6	0.7	16	14	14	186	180	107	51	107.6	4.7	77.3	444	13.5	39.9	22.5	331	6.7	0.48	141	4.1	104	9	3.4	65	16	15.9	0.5	0.7
66	F	164.1	61.8	22.95	30.1	82	142	67	92	67	5	7.9	4.5	1.3	0.7	24	21	22	306	221	76	71	134.8	7.4	62.6	445	13.7	41.5	24	292	18.2	0.54	143	4.6	104	9.7	4.3	51	11.4	19	0.7	1.1
67	M	164.1	51.2	19.01	16.3	70.5	137	74	95	62	36.7	7.7	4.3	1.3	1.1	22	16	41	241	188	98	51	117.4	4.9	54	397	13.1	38.9	25.8	244	12	0.74	143	4	106	8.6	4.5	86	15.9	15.5	0.6	0.6
57	M	162.1	70.3	26.75	26.7	88	123	61	81.67	59	20.2	7.5	4.6	1.6	0.4	41	44	117	206	164	64	60	91.2	5.5	76.2	436	14.5	42.6	23	315	13.6	0.96	146	4.5	107	9.8	7.4	66	12.4	12.9	0.5	0.6
57	M	159.7	61.2	24	24	77	138	80	99.33	49	13	7.7	4.7	1.5	1.1	31	33	146	154	158	184	47	74.2	5.6	63.3	464	15.5	45.2	23.7	320	16.6	0.87	143	4.5	106	9.5	8.4	75	11.7	11.9	0.7	0.6
57	M	164.1	73.3	27.22	25.7	90	146	86	106	46	15.9	7	4.4	1.7	0.5	24	40	29	249	207	122	56	126.6	4.9	55.4	496	15.2	44.9	21.1	355	15.2	1	143	4.1	106	9.4	6.8	67	10.1	9.5	0.8	0.6
56	F	149.5	50.6	22.64	29.2	65	142	83	102.7	58	8.9	7.1	4.6	1.8	0.8	20	21	16	303	215	58	60	143.4	4.8	42.7	449	14	40.4	26.5	257	16.9	0.51	142	4	105	9.4	3.4	71	9.5	8.8	0.6	0.7
58	F	160.1	50.1	19.55	20	67	91	48	62.33	47	29.7	7.1	4.4	1.6	1.3	37	32	18	187	243	65	95	135	5.1	41.3	390	12.1	35.9	17.7	292	18.7	0.56	142	4	106	9	4.4	98	14	12.5	0.7	0.8
55	M	168.5	71.5	25.18	25.1	86	133	69	90.33	58	5.3	7.4	4.6	1.6	0.7	23	17	24	146	173	97	61	92.6	4.9	55.6	430	14.2	42	22	264	17.1	0.92	144	4	106	9.5	7	54	14.9	16	0.5	0.5
55	F	158.3	52.3	20.87	23.5	66	93	45	61	48	14.3	7.2	4.4	1.6	0.6	26	22	19	149	209	81	76	116.8	5.4	32	406	12.9	38.3	14.5	250	13.6	0.61	142	4.1	107	9.5	4.2	113	8	7.9	0.5	0.7
59	M	161.3	53.6	20.6	18.9	76	139	83	101.7	52	11.4	7	4.3	1.5	0.9	21	16	23	238	205	119	51	130.2	5.3	51.4	425	13.8	40.6	27.8	315	20.4	0.67	143	4	107	9	7.4	73	10.1	9.4	0.8	0.8
66	M	165.5	47.5	17.34	9.8	66	128	78	94.67	52	22.8	6.7	4.4	1.9	0.9	21	14	13	204	163	55	85	67	6.4	46	421	14.6	42.9	20.2	239	13	0.65	145	4.1	107	9	3	135	11.9	11.8	0.6	0.6
63	F	154.1	57.7	24.3	30.1	73.5	142	72	95.33	63	20.6	7.3	4.1	1.3	0.6	23	15	17	225	228	137	74	126.6	4.9	50	425	13.6	40.5	19.5	296	18.8	0.76	144	4	107	9.4	4.1	50	9.9	12.4	0.5	0.7
62	M	164.4	51.6	19.09	18.6	75	133	75	94.33	57	45	7.7	4.5	1.4	0.5	29	27	16	285	129	89	35	76.2	5.6	57.1	417	13.5	39.2	19.7	283	21.7	0.77	142	4.1	106	9.4	6.3	216	11.1	10.2	0.6	0.6
57	M	166.4	55.3	19.97	16.1	70	131	80	97	55	9.6	7.1	4.6	1.8	0.8	18	16	21	115	212	138	66	118.4	5.1	62.2	449	13.9	40.6	23.1	253	17.9	0.67	145	4.2	107	9.1	3.2	63	9.5	17.1	0.7	0.7
68	F	150	45.8	20.36	25	67	117	50	72.33	80	3.9	7.4	4.6	1.6	0.5	21	13	18	235	211	151	44	136.8	5.5	56.6	429	13.3	39.7	27.8	343	9.7	0.7	144	4	104	9.1	4.9	83	13.4	14.2	0.6	0.6
58	M	170.4	81.2	27.97	25.2	95	127	79	95	57	5.6	7.2	4.6	1.8	1.2	29	19	102	239	213	253	68	94.4	5.9	49.6	445	16.1	45.5	17.8	326	11.2	0.9	145	4.3	107	9.1	5.9	61	10.2	14.6	0.4	0.4
53	M	161	54.5	21.03	19.8	72	120	69	86	47	15	7	4.3	1.6	0.8	18	27	42	260	166	136	46	92.8	5.5	68.1	485	15.4	45.4	22.8	279	19.6	0.84	144	4.7	108	9.1	4.8	64	10.6	9.5	0.6	0.7
61	M	168.5	60.5	21.31	17.2	81	165	81	109	55	21.9	7.1	4.3	1.5	0.5	35	32	77	316	197	114	63	111.2	7.5	63.7	429	13	39	30	477	25.4	1.17	144	3.8	107	9.5	7.9	48	8	10.8	0.6	0.6
58	M	169.3	71.8	25.05	25.7	86	138	88	104.7	43	7	7.5	4.6	1.6	0.6	21	19	43	233	240	198	57	143.4	5.8	68.8	508	15.3	44.8	26.3	310	17.8	0.88	144	4.1	105	9.3	3.7	66	13.1	10.4	0.7	0.7
54	M	162.2	81.8	31.09	30.9	95	153	100	117.7	65	9.5	7.3	4.4	1.5	0.7	56	64	72	172	165	101	59	85.8	7.1	72.8	498	16.4	46.2	15.2	244	14	0.61	143	4	105	8.7	5.2	42	14.2	16.1	0.5	0.5
54	F	150.5	55.1	24.33	30.8	71	107	63	77.67	60	8.2	8	4.7	1.4	0.5	23	25	22	155	279	132	104	148.6	6	63.4	428	12.8	38	27.5	279	25	0.71	143	3.9	104	9.3	5.1	95	12.1	11.3	0.5	0.6
65	F	144.7	54.2	25.89	38.4	74.5	114	57	76	59	20.5	7.8	4.4	1.3	0.9	19	13	22	210	259	118	69	166.4	5.2	59.2	438	13.8	41.6	24.7	349	15.8	0.55	142	4	104	9.4	4.3	90	11	10.6	0.6	0.6
57	M	158.1	58.5	23.4	16.9	77	158	88	111.3	67	21.5	7.2	4.7	1.9	1.4	26	18	71	216	174	168	69	71.4	4.9	56.2	455	14.7	42	25.2	239	10.5	0.89	144	3.9	105	9.5	5.9	41	13.2	16.2	0.7	0.6
53	M	174	61.5	20.31	16.3	71	112	70	84	64	4.8	7.3	4.7	1.8	0.9	24	35	80	242	247	84	95	135.2	5	41.8	462	14.9	43.3	20.4	200	21.3	1.03	143	4	104	9.4	5.5	67	10	14.4	0.7	0.8
60	M	173.4	87.4	29.07	34.6	93	144	82	102.7	76	223	7.7	4.6	1.5	1.3	29	25	66	190	241	188	53	150.4	5.2	65	532	16.6	47.4	21.5	296	17.1	0.91	143	4.2	104	9.7	8.2	105	14.5	11.5	0.8	0.8
65	F	150.3	50.4	22.31	27	67	114	56	75.33	68	43.9	7.3	4.4	1.5	0.9	22	9	24	253	241	83	92	132.4	5	57.3	368	11.9	36.1	22.9	296	15.3	0.67	146	4	105	9.3	3.8	89	9.6	11.4	0.6	0.6
55	M	166.5	68.4	24.67	24.3	85	112	61	78	58	36.8	7.2	4.3	1.5	0.7	20	22	34	165	204	127	47	131.6	4.8	60.7	458	14.8	43.1	19.1	233	18.5	0.73	144	4.1	105	9	5	86	8.9	9.1	0.8	0.6
49	F	156.3	62.4	25.54	30.9	73	111	59	76.33	56	17.2	6.8	4.2	1.6	0.6	19	25	43	155	161	56	51	98.8	4.8	55.8	406	12.8	36.7	23.7	260	10.5	0.59	142	4.2	109	9.2	4.1	57	9.9	9.8	0.5	0.5
59	M	177.7	59.8	18.94	13.7	69	130	74	92.67	98	8.7	7.5	4.8	1.8	2.2	18	12	17	206	201	55	84	106	4.7	45.9	505	15.7	46.2	27	279	15	0.99	144	4.5	104	9.9	3.3	100	10	12.3	0.5	0.6
57	M	160.5	58.1	22.55	24.2	78	147	94	111.7	66	6.3	8.1	4.8	1.5	0.9	30	43	90	202	173	71	53	105.8	4.8	56.4	492	16.2	46.6	26.1	287	22.3	0.71	141	4.1	100	9.2	5.3	62	12.6	11.9	0.6	0.8
59	F	145.9	45.6	21.42	26.8	64	108	50	69.33	64	16.6	7.4	4.3	1.4	0.8	22	15	13	193	260	88	74	168.4	5.9	49	441	13	39.1	28.6	283	13	0.65	143	3.8	103	9.3	4	110	11.7	8.9	0.5	0.5
58	M	170.5	68.1	23.43	19.5	77	160	78	105.3	60	8.8	7.2	4.5	1.7	0.6	19	27	83	244	211	169	35	142.2	5.2	58.4	490	15.8	46.9	17.9	315	11.7	0.83	145	3.7	106	9.1	6.4	62	9.7	9.3	0.5	0.9
57	F	163.5	55.2	20.65	26.1	69	124	63	83.33	62	69.9	8	4.8	1.5	1.1	17	15	49	192	218	88	57	143.4	4.6	39.1	450	13.6	40.5	25.9	226	12.3	0.52	143	4	106	9.5	6.1	69	17.4	18.2	0.6	0.7
68	F	155.7	52.4	21.61	26.9	69	132	73	92.67	62	42.9	7.7	4	1.1	0.6	21	14	17	274	214	72	62	137.6	4.7	36.8	407	12.8	38.2	23.2	337	14.7	0.72	142	4.1	104	9.4	4.6	53	9.5	12.2	0.7	0.7
66	M	166.1	69.5	25.19	28.2	83	150	82	104.7	64	26.4	7.3	4.4	1.5	0.7	32	36	31	207	196	98	69	107.4	5.3	47.3	430	14.6	42.3	14	296	16.1	1.07	141	3.8	104	9	6.4	86	13.9	15.3	0.6	0.7
62	F	151.3	52.2	22.8	28.8	72	136	73	94	62	22	7.4	4.4	1.5	0.9	20	15	16	186	243	123	73	145.4	5.2	54.5	437	13.9	41.6	28.7	326	10.1	0.61	143	4.1	105	9.2	4.6	99	14.4	14.2	0.6	0.6
54	F	156.3	61	24.97	30.8	77	132	59	83.33	52	44.2	7.5	4.6	1.6	1	17	13	21	215	192	96	71	101.8	4.4	47.8	440	13.8	39	22.3	247	14.5	0.57	143	4.6	105	9.9	2.7	55	10.2	13.5	0.5	0.8
67	F	151.5	48.9	21.31	27.1	69	107	60	75.67	47	13.9	8	4.4	1.2	0.7	23	24	33	128	233	72	63	155.6	5.2	61.5	478	14.8	45.2	24.6	326	19.6	0.78	143	4.2	105	10.1	4.6	118	15.4	13	0.6	0.5
65	F	159.7	68.2	26.74	33.3	81	132	74	93.33	69	7.9	8.3	4.7	1.3	0.5	32	19	20	168	215	124	68	122.2	4.8	56.1	459	14.2	42.4	33.1	305	11.2	0.55	143	4.5	107	9.6	4.6	71	15.4	13.1	0.5	0.6
63	F	160.5	48.8	18.94	20.9	66	108	68	81.33	57	27	7.4	4.5	1.6	0.9	20	14	11	249	173	98	47	106.4	4.9	51.6	417	13.1	39.9	24.1	283	14.5	0.67	142	4.2	106	9.6	4.2	75	10.3	10.6	0.8	0.8
63	M	165.3	61.8	22.62	18.1	82	114	70	84.67	63	32.8	6.8	4.2	1.6	1	35	26	26	116	186	36	65	113.8	5.1	56.2	456	14.1	42.7	19	337	15.1	0.97	141	4.2	106	9	6.5	80	10.5	7.9	0.8	0.6
53	F	147.3	44.4	20.46	25.3	62	127	80	95.67	65	4.4	8	4.4	1.2	0.6	24	22	18	155	205	47	60	135.6	4.7	56.6	454	14	39.9	27.7	375	15.3	0.61	142	4.2	105	9.6	5.5	70	15.9	16.3	0.6	0.6
83	M	160.5	52.5	20.38	16.6	73	131	71	91	59	38.2	7.1	4.4	1.6	0.6	23	10	13	290	195	37	73	114.6	5.4	50.2	420	13.9	41.1	23.5	257	9.6	0.89	140	4.4	101	9.5	6.3	103	8.3	9.3	0.8	0.7

62	M	157	52.4	21.26	15.3	69	104	62	76	45	11.3	6.9	4.1	1.5	0.6	24	23	15	214	197	73	75	107.4	6	48.8	388	12.8	37.5	14.6	223	21.7	0.58	142	4	107	8.9	3.7	105	11.4	10.7	0.6	0.7
67	F	147.8	58.3	26.69	35.4	84	157	101	119.7	92	43.5	8.3	4.6	1.2	0.5	22	21	38	236	281	136	60	193.8	5.1	100.2	499	15.4	46.4	30.1	337	16.7	0.79	145	4.2	104	9.8	6.3	64	8	9	1	0.7
68	M	158.9	67.8	26.85	29.7	90	128	74	92	52	35.7	7.4	4.5	1.6	0.9	22	28	29	255	202	110	59	121	4.5	65.2	508	16.1	47.1	16.5	267	14.5	0.68	143	3.9	107	9.3	5.7	89	10.7	10.6	0.7	0.9
63	M	167.3	64.1	22.9	21.1	80	147	84	105	55	22.6	7.3	4.5	1.6	0.8	19	12	50	248	224	100	70	134	5.5	44.9	412	14.9	42.3	20.8	271	11.3	0.76	141	4.4	102	9.4	5.1	71	13.7	13.4	0.5	0.7
54	F	151.8	53.3	23.13	28.8	73	135	83	100.3	54	27.6	6.8	3.9	1.3	0.6	19	16	30	224	189	64	63	113.2	4.9	61.2	426	13.3	40.2	16.9	343	15	0.69	143	3.7	107	8.7	4.6	73	9.2	9.2	0.5	0.6
65	F	156.5	44.5	18.17	19.6	63	107	61	76.33	61	29.4	6.9	4.5	1.9	1.1	22	16	12	169	206	53	78	117.4	5.1	38.7	415	12.9	38.1	17.6	223	15.7	0.71	144	4	106	9.3	4.9	66	11.4	10.6	0.7	0.6
69	F	155.1	54.5	22.66	31.4	77	135	63	87	61	36	7.8	4.4	1.3	0.8	26	19	25	202	239	127	62	151.6	5.9	49.6	457	14.4	42.9	21.7	267	15.3	0.62	141	4.2	101	10	5.4	61	16.7	14.9	1.2	1.1
63	M	172.4	85.4	28.73	29.3	98	125	61	82.33	48	5.9	7.7	4.6	1.5	0.5	17	21	26	195	177	100	53	104	6	62.8	471	14.9	43.7	25.2	361	15.2	0.67	142	3.9	105	9.4	5	85	12.1	14.9	0.9	0.7
67	F	153.1	59.9	25.56	31	73	131	57	81.67	54	51.1	7.2	4.4	1.6	0.5	25	16	29	268	270	50	86	174	5.2	45.5	407	13	39.3	17.5	279	12.9	0.66	144	4.4	107	9.3	3.7	119	14.7	12.7	0.9	1.1
65	F	149	56.7	25.54	34.1	79	120	63	82	50	17.1	8.3	4.4	1.1	0.8	21	19	16	287	214	167	38	142.6	5.2	54	437	13.9	41.2	24.1	305	9.2	0.57	142	4	105	9.6	5.4	58	10	9.7	0.7	0.9
67	M	164.6	60.5	22.33	24.8	81	173	74	107	53	20.4	7.4	4.6	1.6	0.6	19	17	66	250	222	238	57	117.4	7.1	58.2	448	14.2	41.5	23.8	279	15.8	0.92	141	4.4	103	9.7	5.6	52	13.5	13.1	0.5	1
64	F	150.1	57.1	25.34	32.7	78	121	71	87.67	65	21.5	7.4	4.5	1.6	1.4	19	24	50	205	258	317	47	147.6	5.5	57	426	13.6	39.2	33.8	250	13.2	0.69	141	4.3	104	9.7	6.1	55	22.2	23.6	0.9	1.2
67	F	143.9	44.5	21.49	28.1	70	129	61	83.67	66	11.8	7.6	4.5	1.5	0.9	22	12	25	301	312	66	55	243.8	5.4	59.1	431	12.9	38	27.7	337	14.5	0.53	141	3.9	109	9.4	5.2	80	10.8	9.9	0.6	0.7
63	F	147.7	39.9	18.29	19.7	63	125	67	86.33	58	34.6	7	3.7	1.1	0.7	30	13	18	349	187	98	58	109.4	5.3	78.6	386	11	33.6	27.1	337	20.2	0.78	141	4.1	106	8.5	3.7	134	15.2	15.3	0.6	0.7
57	M	165	57.9	21.27	15	80	124	69	87.33	65	7.8	6.8	4.3	1.7	0.6	24	20	87	162	174	41	70	95.8	5.6	48.6	420	15.3	44.5	15.2	375	11.9	0.83	143	4.3	108	9.1	6	95	10.9	10.9	0.5	0.7
54	F	147.3	45.6	21.02	25.1	64	104	57	72.67	63	22.7	6.7	4.2	1.7	0.6	20	18	16	182	196	93	76	101.4	4.9	49.4	401	13.3	39.3	28	305	12	0.63	144	4	108	8.9	3.4	157	13.9	13.1	0.4	0.4
54	M	172.5	66	22.18	17.5	77	120	66	84	62	9.7	6.7	4.2	1.7	0.8	12	12	28	213	220	86	63	139.8	5.3	66.4	460	14.5	43.2	27.4	264	14.5	1.05	143	4.3	109	9.4	4	106	12.6	10.1	0.4	0.4
50	F	158.7	54.9	21.8	25.9	68.5	98	53	68	63	10.5	6.8	4.1	1.5	0.4	18	12	13	242	211	67	65	132.6	5.2	39.7	376	10.6	33	25.4	287	16.1	0.6	142	4	105	9.1	4.4	105	9.3	9.6	0.7	0.6
60	M	164.1	66.4	24.66	24.6	84	142	77	98.67	54	7.5	7.3	4.5	1.6	1.7	23	23	25	203	227	88	47	162.4	5.1	45.9	520	15.3	45.5	18.4	247	17.2	0.93	144	3.8	106	9.2	6	85	10.8	11.1	0.6	0.5
61	F	149.5	45.9	20.54	25.6	66	104	66	78.67	69	8.8	7.6	4.4	1.4	0.8	20	12	20	133	223	101	51	151.8	5.4	44.4	413	13.1	38.8	23.2	292	15.3	0.64	143	4.4	105	9.6	5.1	123	14.4	10.3	0.5	0.5
63	M	164.3	62.2	23.04	23.9	85	117	67	83.67	70	26.2	7.3	4.1	1.3	0.4	22	15	22	220	246	109	45	179.2	5.4	137.1	439	15.4	44	22.2	331	10.3	0.81	141	4.5	107	8.9	5.8	49	8.2	10.2	1.5	1.6
60	F	148.1	51.3	23.39	31.2	74	133	61	85	57	12.2	8.1	4.3	1.1	0.4	18	14	19	235	303	295	56	188	5.5	61.3	367	12.3	36.5	26.7	337	15.1	0.66	142	4	105	9.4	4.6	92	14.1	11.5	0.6	0.7
56	F	148.1	46.4	21.15	23.9	69	98	41	60	58	22.9	7	4.3	1.6	1	24	19	19	226	174	68	65	95.4	5.3	50.8	350	11.7	34.9	22.7	247	12.9	0.64	143	4.1	107	9.3	3.5	50	13.3	11.2	0.4	0.5
69	F	153.3	44.6	18.98	23.8	68	114	73	86.67	66	48	7.3	4.4	1.5	0.6	29	19	22	280	249	102	83	145.6	5.4	75.3	439	13.9	42.6	28.8	287	14	0.6	143	4.3	106	9.6	3.5	105	12.2	12.4	0.7	0.6
63	F	156.4	60.8	24.86	31.6	77	108	64	78.67	71	8.3	8	4.7	1.4	0.8	24	31	31	251	214	164	67	114.2	8	67.6	494	15	43.2	21.5	331	20.8	0.67	141	3.8	104	9.4	3.9	69	8.3	8	0.8	0.7
62	M	165.7	72.3	26.33	21.4	83	127	82	97	58	11.4	7.4	4.7	1.7	1	20	25	35	207	187	93	76	92.4	4.6	44	433	15	42.8	16	287	12.8	0.74	145	4.2	105	9.6	6.9	65	12.5	20.3	0.7	0.8
66	F	152.3	45.5	19.62	24.4	65	131	62	85	63	23.6	7.8	4.4	1.3	0.6	21	13	16	276	223	92	72	132.6	5.3	56.6	453	14.2	42.7	23.3	403	13.9	0.66	142	4.4	101	9.8	3.9	126	17.9	15.8	0.9	1.2
63	F	155.3	42.8	17.75	19.9	58	96	42	60	71	35.3	7.4	4.7	1.7	1	23	15	15	190	230	57	117	101.6	5	37.5	404	13	38.5	18.1	283	10.1	0.61	143	4.9	105	9.8	4.2	63	14.2	14.4	0.6	0.7
50	M	172.4	57.7	19.41	19.2	72	138	68	91.33	63	14.9	7.8	4.7	1.5	0.6	32	22	129	278	167	66	69	84.8	5	77.5	479	17.8	50.1	31.3	271	9.4	0.82	144	5	102	10	7.4	67	11.8	12.8	0.5	0.6
52	M	160.5	69.1	26.82	24.1	88	111	60	77	58	22.7	7.4	4.1	1.2	1.1	22	22	29	220	252	200	45	167	4.7	80.7	529	16	46.7	24	260	11.4	0.92	143	4	103	9.3	6.2	65	11.3	11.4	0.6	0.7
51	M	156.1	66.8	27.41	32.1	95	137	78	97.67	61	19.8	7.3	4.7	1.8	0.4	29	34	371	221	199	80	77	106	4.4	64.9	448	15.1	43.8	20.5	301	14.6	0.59	147	4.1	107	9.3	6.9	82	11.4	10.7	0.7	0.6
53	M	167.5	70.2	25.02	23.6	82	136	88	104	59	5.7	7.9	4.4	1.3	1	22	27	31	204	204	130	79	99	5.1	97.3	491	15.7	45.7	33.1	283	16.3	0.88	146	4.1	105	9.7	5.9	84	13.1	12.1	0.5	0.5
67	M	166.7	52	18.71	19.2	68	129	67	87.67	62	13.5	7.3	4.4	1.5	1.6	36	21	25	238	218	86	64	136.8	4.9	73.9	456	14.4	43.2	26.7	315	19.8	0.8	146	4.6	104	9.7	5.8	110	14.7	14.6	0.7	0.9
49	M	172.9	84	28.1	25.3	91	104	56	72	69	11.9	7.8	4.8	1.6	1.2	31	41	62	136	276	162	56	187.6	4.8	60	490	15.8	45.3	27.3	301	14.1	0.93	145	3.6	107	9.8	6.6	50	16.8	16.9	0.6	0.5
60	M	163.7	62.2	23.21	23.1	82	115	69	84.33	61	38.1	7.2	4.4	1.6	0.6	32	26	52	190	191	47	84	97.6	4.7	54.2	459	14.6	42.8	25	368	12.5	0.96	143	3.8	106	9.7	6.4	80	14.6	15.3	0.7	0.7
60	F	149.1	50.3	22.63	32	68	128	73	91.33	58	17.5	7.6	4.4	1.4	0.6	27	19	21	202	213	104	53	139.2	5.5	60.5	391	12.8	37.9	15.6	296	13.1	0.56	145	3.7	104	9.5	3.6	66	12.8	13.8	0.6	0.7
64	F	155.7	50	20.62	27	70	109	65	79.67	61	8.5	7.4	4.2	1.3	0.6	19	11	21	190	197	76	85	96.8	5.2	67.9	443	12.9	39.5	23.9	305	14.9	0.53	143	3.7	103	9.5	3.3	85	12.4	11.9	0.5	0.7
67	F	150.5	51.5	22.74	29.7	69	110	74	86	68	11	8	4.7	1.4	0.6	22	12	15	185	217	147	52	135.6	4.9	52.9	418	12.9	38.3	25.2	337	12.7	0.64	141	3.8	103	9.3	4.7	95	10.7	10.6	0.5	0.6
47	F	159.2	54.6	21.54	27	69	107	49	68.33	55	21.6	7.1	4.3	1.5	0.8	13	8	10	159	233	75	64	154	4.9	86.3	399	12.3	36.6	29.5	326	9.6	0.55	140	3.7	103	9.4	4.7	66	9.2	10.4	0.7	0.7
63	F	149.9	57.9	25.77	40	79	132	77	95.33	62	3.8	6.7	4.3	1.8	0.8	18	19	21	261	211	159	49	130.2	4.9	54.1	434	14	41.4	20.2	320	16	0.63	144	4.5	105	9.1	6	62	12.6	14.6	0.5	0.6
65	M	166.1	76.9	27.87	32.2	95	131	79	96.33	74	2.5	7.4	4.8	1.8	0.5	36	63	72	138	221	160	49	140	5.4	55.3	433	13.5	39.4	29.9	253	23	0.73	142	4.2	103	9.4	5.7	76	15.1	12.2	0.8	0.8
57	F	147.5	43.6	20.04	24	63	108	60	76	69	13.3	7.6	4.5	1.5	0.6	24	15	19	211	264	98	84	160.4	5.4	54.7	496	13.7	42.6	28.8	226	17.6	0.58	143	3.8	105	9	4.1	80	13.1	11.1	0.5	0.6
63	M	165.6	68	24.8	21.6	80	143	84	103.7	61	7.8	7.8	4.5	1.4	0.8	24	21	57	156	177	203	60	76.4	5.1	64.1	480	15.3	45.6	23.1	264	14.9	0.83	142	4.1	101	9.6	5.9	73	10.6	10.4	0.5	0.7
51	M	167.7	69.1	24.57	23.7	83	117	61	79.67	57	5.2	8	4.7	1.4	0.7	26	49	58	222	194	99	47	127.2	6.1	54	442	14.1	42.4	24.2	257	12.8	0.93	142	4.2	103	9.6	6.8	87	12.9	12.7	0.5	0.6
52	M	167.6	58.4	20.79	18.4	70	117	73	87.67	61	8.6	7.3	4.4	1.5	0.8	26	25	75	266	191	87	90	83.6	4.6	55	442	14.7	42.2	26.8	253	11.8	0.85	142	4	102	9.4	4.6	89	10.3	10.9	0.5	0.6
56	M	158.3	63.7	25.42	24.7	85	139	79	99	55	11.5	7.5	4.5	1.5	1.1	27	37	58	285	222	159	60	130.2	5.4	83.2	512	15.6	46.7	21.7	349	15	0.83	143	4.2	105	9.9	6.6	79	11	9.6	0.7	1.1
50	F	158.1	52.8	21.12	25.5	68	150	78	102	78	15.9	7.2	4.2	1.4	0.9	16	10	14	246	199	54	114	74.2	5.1	55.1	445	13.7	41.8	26.2	320	16.2	0.68	141	3.8	102	9.4	5.3	63	17.8	16	0.5	0.7
62	M	179.6	76	23.56	27.3	85	154	78	103.3	65	11.1	8.2	4.5	1.2	1.2	30	30	26	384	177	145	58	90	5	74.5	494	16.2	47.9	24.9	310	14.7	0.82	140	4.1	102	10.6	4.9	109	15.4	15.5	0.8	6.1
64	F	154.5	47.9	20.07	24.6	63	146	93	110.7	75	37.5	7.4	4.5	1.6	0.7	30	17	13	290	248	48	119	119.4	4.5	51.6	400	12.3	37.2	30.7	292	16.2	0.56	144	3.7	105	9	4.8	90	11	11.5	0.5	0.6
69	F	148.7	57.3	25.91	37.5	77	160	89	112.7	59	29.9	7.3	4.5	1.6	0.6	20	19	21	209	260	67	90	156.6	5.2	71	439	13.9	41.2	21.2	305	14.7	0.58	144	4.2	106	9.3	4.8	90	12.1	10.2	0.8	0.9
63	F	150.1	48.9	21.7	25.6	68	106	62	76.67	63	60.6	6.5	4.3	2	0.7	26	18	20	212	197	62	51	133.6	4.9	54.8	347	11.9	34.9	20.7	264	15.1	0.58	143	4.4	104	9	4	42	8.9	7.7	0.9	0.7
67	F	152.6	47.1	20.23	27.3	68	139	75	96.33	73	10.5	8	4.5	1.3	1	28	18	13	183	273	161	57	183.8	5.4	52.7	393	12.3	37.5	18.2	368	12.8	0.6	143	3.8	103	9.5	4.1	89	10.4	11	0.6	0.6
61	M	169.7	71.6	24.86	29.1	90	143	73	96.33	63	7.6	7.6	4.6	1.5	0.7	31	37	82	208	222	107	58	142.6	5.5	75.4	513	17.9	52.9	19	331	10.1	0.67	142	4.3	103	9.1	5.1	45	15.3	13.8	0.7	0.8
57	M	165	67.8	24.9	32	90	189	96	127	88	8	7.6	4.6	1.5	0.7	25	32	57	207	226	142	49	148.6	5.9	61.5	548	16.7	49.1	17.3	403	13.8	0.62	142	4.6	101	9.5	6.3	47	16.1	12.9	0.7	0.6
58	F	157.7	50.7	20.39	29.8	70	150	85	106.7	77	6.4	8	4.6	1.4	0.7	27	21	26	196	245	129	57	162.2	5	39.8	474	14.8	43.2	20.8	326	14.6	0.71	143	4.2	103	9.7	3.6	128	19.4	15.9	0.7	0.6
64	F	153.4	57.6	24.48	35.1	81	119	63	81.67	56	25.1	7.2	4.3	1.5	0.9	28	44	32	536	214	65	59	142	5.4	58.1	488	14.6	43.2	23.8	271	19.2	0.61	145	3.6	105	9.1	4.4	85	9.3	8.5	0.7	0.8
55	F	158.9	59.2	23.45	31.1	74	105	68	80.33	67	7.8	7.4	4.2	1.3	0.7	21	16	18	226	222	65	95	114	5.3	58.8	412	12.9	38.7	37.4	275	16.6	0.58	143	4.5	103	9.3	5	76	9.6	11.8	0.6	0.7
63	M	157.4	53.7	21.68	18.3	77	167	90	115.7	66	22.6	6.9	4.2	1.6	0.3	21	27	38	552	147	103	49	77.4	6	63.9	294	10.2	30.2	29.8	301	60.4	3.58	139	5.5	106	9	7.6	188	10.9	13.5	0.7	0.6
66	F	149.7	41.8	18.65	20.1	64	145	81	102.3	66	58.2	7.2	4.5	1.7	0.8	38	28	12	319	205	42	105	91.6	4.6	40.8	412	12.3	37	23.9	368	17.5	0.53	144	4.2	104	9.4	3.3	98	8.9	9.6	0.9	0.7

68 M	161.2	68.7	26.44	26.4	84.5	139	76	97	55	6.4	7.3	4.4	1.5	0.9	25	21	140	265	195	89	59	118.2	5.3	59.8	451	15.3	44.6	23.7	271	16.3	0.89	141	4	98	9.4	5.9	95	10.6	11.3	0.7	0.6
69 F	148.5	54.3	24.62	32.9	73	98	57	70.67	48	21.3	7.5	4.4	1.4	0.8	21	7	13	287	197	157	61	104.6	5.5	35.5	416	13.8	41.2	26.5	326	11.8	0.52	142	5.2	103	9.2	3.7	87	9.2	11.7	0.6	0.7
68 F	146.3	41.7	19.48	22.7	61	103	53	69.67	76	32.2	7.4	4.4	1.5	0.9	25	18	20	171	225	67	90	121.6	5.2	51	429	13.7	41.5	17.8	264	16.8	0.66	142	4.1	101	9.2	4.2	77	14.5	10.4	0.7	1
54 M	166.5	71	25.61	26.2	85	118	73	88	58	7.6	7.4	4.5	1.6	0.6	21	27	57	268	232	291	39	134.8	5.2	92.1	516	16.1	48.5	26.3	296	11.3	0.59	142	4	103	8.8	6.3	55	8.3	9.1	0.8	0.9
57 M	172.1	85	28.7	27.9	94	139	76	97	72	3.3	7.6	5	1.9	0.6	34	56	61	177	274	235	46	181	5.7	61	504	16.5	45.9	21.7	267	17.7	0.91	144	5	104	9.9	7.6	46	14.1	15.7	1.2	0.8
52 M	157.7	60.9	24.49	20	82	136	85	102	46	13.7	7.4	4.7	1.7	0.7	23	22	26	225	191	107	50	119.6	4.4	55.8	527	16.6	47.1	21.5	271	16.3	0.93	142	3.6	103	9.1	4.9	88	7.9	9.6	0.7	0.7
68 F	153.3	54.2	23.06	30.8	76	111	58	75.67	77	14.4	7.8	4.7	1.5	0.9	18	14	29	462	249	130	46	177	5.6	63.4	399	13	38.4	28.9	301	11.8	0.45	142	4	101	9.5	3.7	63	13.4	9.6	0.7	0.6
67 M	166.1	68.8	24.94	21.4	87	153	80	104.3	52	23.9	7.2	4.4	1.6	0.7	31	47	34	244	170	69	40	116.2	6.5	61.2	468	15.4	45.5	17.1	424	14.2	0.73	143	4.4	102	9.1	4.5	101	14.6	13.2	0.7	2.1
56 F	151.1	47.6	20.85	29.8	69	125	80	95	70	4.6	8	4.7	1.4	0.7	18	17	15	347	211	80	41	154	5.2	58.7	464	14.1	42.1	26	337	11.8	0.63	143	4.2	103	9.8	4.9	55	16.5	14	0.6	1.6
52 M	161.1	74.5	28.71	29.9	90	136	77	96.67	59	4.4	7.2	4.3	1.5	0.3	23	56	48	319	249	182	45	167.6	6.6	108	510	16.4	48.7	41.6	283	11.8	0.95	143	4.2	107	9	6.2	54	15.8	15.7	0.9	0.9
65 F	156.5	56.1	22.91	29.8	73	109	59	75.67	60	43.8	7.1	4.4	1.6	0.8	22	19	19	242	287	68	78	195.4	5.5	58.6	423	13	39.7	30.1	349	15.1	0.5	145	4.5	105	9.5	5	63	10.5	7.6	1.5	0.7
69 F	140.4	56.3	28.56	34.9	79	145	83	103.7	97	11	7.6	4.7	1.6	1.1	35	31	23	396	180	103	68	91.4	5.8	82.8	431	13.7	40.8	28.8	271	11	0.54	145	4	101	9.7	3.9	70	14.8	11.5	0.7	0.5
67 F	146.1	50.6	23.71	30.1	70	155	96	115.7	61	28.1	6.4	4	1.7	0.6	17	23	32	290	203	74	92	96.2	5.6	93.7	389	12	35.3	43.5	414	18.3	0.49	145	4	105	9.3	3.8	45	15.5	15.3	0.5	0.7
64 M	174.1	82.6	27.25	27.1	94	122	77	92	56	4.8	7.8	4.6	1.4	0.8	32	34	27	208	206	106	49	135.8	5.8	79.7	470	15.6	46.9	27.1	394	14.3	1.14	144	4.5	106	9.1	8.1	98	12.1	12.5	0.7	0.9
66 F	148.5	52.6	23.85	31.2	74.5	125	79	94.33	56	10.3	7.3	4.6	1.7	0.7	29	16	18	185	203	68	68	121.4	5.4	39.4	425	15	43.2	20.5	279	13.6	0.64	145	3.9	105	9.4	5.1	48	14.9	16.3	0.8	0.8
57 F	156.7	55	22.4	29.5	74	141	59	86.33	68	19	7.4	4.4	1.5	0.6	34	40	22	535	192	405	36	75	5.4	50.7	492	15.1	43.9	20.1	271	10.1	0.53	144	4.6	104	9.4	3.1	78	12.6	15.4	0.9	0.8
66 M	162.5	62.2	23.56	24.3	84	148	68	94.67	55	12.5	8	4.6	1.4	0.6	29	23	47	290	198	108	66	110.4	5.3	62.4	482	16.2	46.8	31	310	13	0.69	144	4.5	104	9.4	5	82	12.4	14.2	0.8	0.6
68 F	150.9	55.9	24.55	32.4	79	165	100	121.7	67	49.6	8.5	4.4	1.1	0.7	25	18	22	310	187	371	38	74.8	5.6	75.4	491	14	42.9	27.8	361	12	0.76	144	4.3	103	9.7	7.1	73	12.8	12.2	1.2	1
41 M	175.3	87.6	28.51	34.5	89	102	50	67.33	57	2.7	7.6	4.7	1.6	1.4	53	96	64	184	287	122	50	212.6	5.2	54.6	559	17.5	50.4	20.9	253	16.6	0.75	142	4	102	9.4	7.3	67	11.6	12.7	0.5	1
58 M	174.7	70.3	23.03	23.5	86	107	58	74.33	62	10.1	7.3	4.3	1.4	0.8	25	19	22	281	192	112	57	112.6	5.4	71	495	16.1	47.2	27.8	287	15.7	0.93	143	4.1	102	9	4.4	65	10.4	8.1	0.7	0.8
66 F	147.6	45.2	20.75	26.8	71	118	66	83.33	56	12.4	7.5	4.5	1.5	0.5	22	16	40	299	241	153	63	147.4	5.2	48.7	366	12	36.4	32.6	315	14.4	0.4	144	4.2	102	9.4	3.8	58	13.6	13.6	0.8	0.8
67 F	144.1	45	21.67	24.9	63	123	76	91.67	52	14.5	7	4.5	1.8	0.7	25	16	21	195	220	111	51	146.8	5.4	68.3	439	13.3	39.9	25.1	247	12.1	0.67	147	4.1	106	9.5	4.8	60	12.9	15	0.7	0.6
68 F	149.9	46.7	20.78	25.9	63	133	63	86.33	61	33.4	7	4.2	1.5	0.7	21	16	18	173	224	71	71	138.8	5.4	45.2	392	13.3	39.6	18.9	271	11.1	0.63	143	4	103	8.8	4	71	14.2	13.9	0.7	0.8
58 M	168.1	60.7	21.48	18.7	73	131	81	97.67	63	7.4	7	4.4	1.7	0.8	26	24	21	191	191	102	70	100.6	5.3	37.2	454	15	43.5	21.4	244	14.7	0.85	144	4.3	102	8.9	4.3	100	17.8	17.3	0.7	0.8
57 F	156.8	51.4	20.91	25.5	65	103	68	79.67	49	11.9	7.5	4.3	1.3	0.7	22	15	12	206	218	67	96	108.6	5.1	38.1	425	13.2	39.6	21.1	320	15	0.54	144	4.1	103	8.9	3.3	119	11.4	14.9	0.7	0.7
66 F	154.5	58.8	24.63	30.6	72	128	53	78	58	31.6	7.7	4.5	1.4	0.5	23	22	14	159	223	79	55	152.2	5.6	41.1	419	13.5	41.1	30.3	287	14.6	0.56	148	5.4	106	9.6	5.2	129	7.7	8.7	1.1	1
57 F	147.3	52.9	24.38	32.2	71	85	51	62.33	61	4.5	7.1	4.2	1.4	0.6	79	121	97	210	200	58	62	126.4	5	37.5	413	13.2	40.8	27.5	275	15.2	0.6	144	4	103	8.9	3.5	98	12.3	11.6	0.7	0.6
51 F	156	71.9	29.54	34.4	85	179	115	136.3	60	3.2	7.8	4.7	1.5	0.5	19	14	23	192	286	90	62	206	5.4	52	425	12.6	38.6	37.9	271	16.4	0.58	142	4.2	101	9.3	4.4	71	17.5	16.2	0.8	0.7
61 F	163.9	69.8	25.98	38.9	85	107	57	73.67	63	6.4	7.5	4.3	1.3	0.8	21	18	14	228	184	95	60	105	4.9	42.4	423	13.5	39.5	26.8	239	12.2	0.67	143	4.2	103	9.3	4.2	97	11.5	11.1	0.7	0.8
67 M	153.3	59.8	25.45	29.5	86	154	91	112	70	36.8	8.1	4.5	1.3	0.5	25	15	49	155	183	184	48	98.2	5	49.4	504	14.5	43.6	31.1	301	13.5	0.65	143	4.4	105	9.2	5.2	81	12.9	9.5	0.9	1
60 M	163.6	70.8	26.45	29.5	89	125	87	99.67	65	180	7.7	4.5	1.4	0.8	42	46	83	237	234	289	51	125.2	5.5	65.4	490	15.5	45.1	18.3	287	13.8	0.97	142	4.5	103	9.9	6.1	77	15.5	14.4	0.6	0.8
56 M	168.3	85.6	30.22	32.8	97	138	73	94.67	66	14.3	8	5	1.7	1.7	37	47	22	219	157	118	53	80.4	4.9	60.2	521	17.5	49.8	18.4	315	16.6	0.86	146	4.6	102	9.9	6.4	141	13.2	14	0.8	0.5
66 M	172.1	73.7	24.88	29.5	89	134	80	98	65	3.1	8	4.8	1.5	1.4	30	27	29	224	230	83	56	157.4	4.8	59.6	459	16	46.4	19.1	326	18.2	0.86	144	4.4	101	9.5	6.9	40	10.6	9.7	1.2	1.2
63 M	165.9	70.8	25.72	27.4	82.5	116	63	80.67	49	17.2	7.8	4.4	1.3	0.8	21	15	21	205	236	83	59	160.4	5.5	65.8	451	14.5	42.3	29.6	436	12.9	1.15	144	4.4	105	9.5	6.1	101	15.9	16.5	0.8	0.7
62 F	144.1	46.5	22.39	29.3	67.5	121	75	90.33	67	9.4	7.5	4.6	1.6	1.1	35	33	21	178	231	80	85	130	5.7	58.2	450	13.4	41.1	39.5	355	14	0.72	144	4.8	100	9.9	5.6	98	12.1	11.9	0.8	0.7
42 F	148.6	59.9	27.13	39	75	119	75	89.67	64	7.2	7.6	4.6	1.5	0.8	34	42	37	157	222	86	85	119.8	7.2	70.2	490	15.8	47	32.3	219	11.9	0.56	143	3.7	101	9.8	3.7	50	17.7	17.6	0.5	0.6
66 F	150.3	66.9	29.61	38.9	85	171	98	122.3	70	9.3	7.4	4.5	1.6	0.6	50	53	69	338	261	141	69	163.8	7.5	41.9	480	14.3	42.9	16.8	326	8.5	0.63	142	4.6	104	9.4	4.9	59	13.6	14.2	0.7	0.9
68 M	161.6	56.2	21.52	21.5	76	141	73	95.67	67	2.3	6.9	4.6	2	0.8	22	31	50	265	185	164	45	107.2	7.4	60.8	546	16.5	48.7	23.4	296	9.3	0.86	146	3.8	104	9.4	3.6	107	12.9	10.6	1.1	1.3
66 F	144.5	46.5	22.27	27.9	66	140	76	97.33	72	23.5	7.3	4.4	1.5	0.8	24	27	32	218	192	108	51	119.4	4.9	49.3	444	13.7	42	36.7	271	14.5	0.55	146	4.2	106	9.2	4.4	78	12.8	13	0.7	0.6
65 M	163.3	66.3	24.86	25.3	80	145	67	93	63	174	7.4	4.6	1.6	0.7	18	17	23	149	165	79	45	104.2	5.4	47.5	510	16.7	48.7	17.5	310	16.9	0.98	142	4.4	99	9.6	6.1	63	9	8.8	0.8	1.2
64 M	163.6	65.4	24.43	24.5	85	127	71	89.67	61	59.2	7.7	4.9	1.8	2.2	24	26	44	187	216	67	66	136.6	4.8	54.5	467	15.3	43.8	20.7	257	14.7	0.87	147	3.7	104	9.5	7.5	106	10.4	8	0.8	0.4
68 F	148.1	52.5	23.94	33.8	76	148	71	96.67	81	9	7.7	4.6	1.5	0.8	27	20	27	282	199	95	44	136	5.6	33.9	427	13.6	40.3	25.3	253	17.3	0.59	145	4.6	103	9.4	4.4	78	18.8	15.2	0.8	0.8
63 M	159.6	55.5	21.79	25.3	73	127	74	91.67	66	7	7.8	4.8	1.6	1.1	34	23	15	259	178	68	53	111.4	4.3	35.3	481	15.3	44.6	19.2	239	14.9	0.68	145	3.9	104	9.4	3.9	93	12.7	15.2	0.6	0.6
53 F	164.6	53.1	19.6	23	65	116	71	86	62	20.2	7.8	4.9	1.7	0.5	20	16	43	309	206	77	84	106.6	5.4	44.8	446	14.4	42.5	17.9	414	11.1	0.53	146	4.4	103	9.9	4.3	88	12.5	9.9	0.5	0.5
48 F	153.5	55.8	23.68	33.6	67.5	107	66	79.67	69	5.2	7.8	4.5	1.4	1.9	22	22	26	239	208	68	56	138.4	4.6	82	494	14.3	42.2	27.1	331	17.7	0.78	141	4	102	9.3	6.4	68	12.9	12	0.7	0.7
63 M	163.7	72.3	26.98	36.9	89	139	83	101.7	67	15.2	7.5	4.6	1.6	0.7	27	26	58	249	206	113	61	122.4	5.4	85.2	469	16.5	47.3	23	279	11.5	0.93	144	3.7	102	9.5	5.4	90	14.2	15.1	0.5	0.5
57 F	149.3	52.2	23.42	28.5	70	151	81	104.3	60	2.1	8	4.7	1.4	0.6	28	34	42	278	191	129	53	112.2	6.8	51.8	464	14.2	41.3	28.2	260	15.2	0.68	142	4	101	9.5	6	67	10.5	10.8	1.1	1.2
54 M	160.4	56.3	21.88	25	76	112	61	78	63	8.9	7.3	4.4	1.5	0.5	17	12	30	154	186	70	64	108	5.4	110.6	442	14.3	42.4	30.4	301	13.8	0.86	144	4.1	103	9.7	7.4	122	15.2	15.5	0.7	0.7
65 F	155.3	52.7	21.85	29.8	70	105	54	71	67	22.7	7.5	4.5	1.5	0.5	26	27	30	271	157	69	52	91.2	5.8	71.2	437	13.7	40.9	29.8	292	12.9	0.58	146	4.1	106	9	4.3	81	15.1	16.7	0.7	0.9
54 M	160.9	57.1	22.06	22.8	75	106	58	74	70	21.7	7.6	4.6	1.5	1.1	23	18	21	144	184	40	61	115	5.5	36.1	448	14.6	43.7	14.9	331	19.6	0.78	143	3.9	102	9.5	4.2	117	12.2	12.3	0.7	0.6
72 F	149.3	38.2	17.14	19.6	60	124	74	90.67	65	8.8	7.1	4.3	1.5	1.3	23	14	31	145	200	72	93	92.6	5.1	57.3	407	12.7	37	26.7	241	15.8	0.65	143	3.9	105	9.2	2.4	93	7.9	7.4	0.8	0.6
64 M	165.4	64.4	23.54	21	78	145	76	99	53	14.5	7.2	4.2	1.4	0.8	21	12	20	232	187	154	59	97.2	4.9	41.4	422	14.8	43.9	21.7	244	14.2	0.83	145	4.4	104	8.8	5	94	7.5	7.6	0.7	0.7
67 M	168.4	60	21.16	19.9	75.5	141	74	96.33	68	6.5	7.4	4.3	1.4	0.9	21	15	24	215	213	79	82	115.2	4.9	50.1	482	15.1	44.9	30.4	250	13.2	0.88	144	3.9	103	9.1	3.9	117	28.9	28	0.9	0.8
65 F	154	54.8	23.11	31.7	70	135	66	89	44	18.9	6.9	4.2	1.6	0.5	21	15	22	270	204	238	65	91.4	5.1	61.6	435	13.9	41.4	24.2	271	10.6	0.7	147	4.4	107	9.1	4.3	82	13.1	15.1	0.8	0.8
57 M	158.5	71	28.26	25.5	89	140	89	106	60	4.9	7.8	4.9	1.7	0.5	20	30	40	275	214	537	38	68.6	7.9	66.1	513	15.9	46.9	24.4	296	14.9	0.79	143	4	101	9.4	4.1	84	18.4	18.1	1.1	1.1
64 M	166	61.7	22.39	16.4	82	114	65	81.33	55	11.7	7.3	4.3	1.4	1.1	27	18	33	170	210	73	69	126.4	5	41.7	433	14.3	42.5	27.1	267	16.4	0.79	144	3.8	105	9.3	5.8	130	13.1	14.4	0.8	0.7
66 F	144	57.2	27.58	39.8	80	132	54	80	81	12.3	8.1	4.4	1.2	0.4	19	16	30	185	158	53	63	84.4	4.9	64.8	411	12.8	39.4	23.8	436	8.9	0.52	144	3.7	102	9.2	4.5	43	10	8.4	1.5	1.2
62 M	169.6	62.8	21.83	14.4	78	148	80	102.7	81	4.1	7.4	4.4	1.5	0.8	21	15	19	308	242	84	65	160.2	5.3	62.6	459	15.3	45.4	32.6	337	14.3	1.02	147	4.3	104	9.6	6.5	73	12	10.8	0.8	0.7
70 M	154.1	56.1	23.62	18.5	77	144	69	94	63	12.9	7.3	4.6	1.7	0.8	22	18	30	156	241	71	79	147.8	4.9	52.7	453	15.4	43.9	22.9	244	18.3	0.95	144	4.1	106	9.1	6.7	101	13.8	13.2	0.6	0.5

66	F	152.1	55.6	24.03	28.8	73	121	70	87	57	14.7	7.5	4.2	1.3	1.2	25	16	27	332	217	81	60	140.8	4.7	54.7	320	11.5	33.2	16.4	375	12.6	0.53	143	4.5	104	9.2	3.3	64	10.3	10.6	0.7	0.6
60	M	163.7	59.3	22.13	22.6	80	127	68	87.67	72	6.2	6.9	4.3	1.7	0.8	34	50	73	295	231	67	66	151.6	4.7	40.5	460	15.4	44.5	18.3	275	16.4	0.84	143	4.3	103	9	5.4	112	18	15.4	0.7	0.5
54	F	157.5	58.5	23.58	33.2	77	115	72	86.33	71	18	7.8	4.3	1.2	0.5	12	9	11	237	184	107	47	115.6	5.3	49.9	476	13.8	42.3	25.7	331	8.9	0.7	143	3.9	103	9	4.6	90	14.6	17.4	0.8	1
59	F	162.9	61.4	23.14	29.4	75	108	64	78.67	68	15.6	6.9	4.4	1.8	0.7	24	22	27	338	196	261	62	81.8	5.3	50.4	463	13.9	43.1	23.4	275	9.9	0.58	144	3.7	103	9.4	4.1	131	11.9	13.3	0.8	1
55	M	180.1	83.2	25.65	36.9	101	138	76	96.67	82	9.7	7.8	4.4	1.3	1	32	53	101	313	202	353	40	91.4	6.3	96.7	538	16.9	49.9	29.7	292	17.4	0.82	142	4.2	100	9.4	7	47	11.9	9.5	0.8	0.6
51	F	150.6	59.9	26.41	34.9	79	134	63	86.67	91	13.6	8.6	4.4	1	0.6	20	20	13	248	170	119	51	95.2	5.4	56.6	468	13	39.7	33.3	414	11.4	0.44	143	3.9	104	9.6	4	85	16.6	13.5	0.7	0.5
67	F	157.3	72	29.1	39.4	97	133	58	83	93	9	7.6	4.7	1.6	0.8	19	22	20	232	181	141	42	110.8	7.4	73	472	14.9	43.6	26	403	22.8	0.61	142	4.7	104	10.1	5.7	94	17.5	19.5	0.8	0.8
62	M	158.5	58.4	23.25	26.3	82	105	59	74.33	60	22.2	7.6	4.4	1.4	0.7	43	55	59	284	202	118	55	123.4	7.5	48.5	442	15	44.3	17.2	250	16.7	0.53	143	3.9	104	9.3	6.2	72	14.6	15.6	0.5	0.6
58	F	155.7	44.7	18.44	22.2	62	155	89	111	91	4.7	7.8	4.8	1.6	0.8	25	29	23	231	237	108	64	151.4	5.5	48.9	422	13.8	40.9	23.6	305	14.8	0.55	142	4	102	9.6	4.7	88	10.6	12.4	0.9	1.5
71	M	148.9	55.4	24.99	22.8	77	132	69	90	51	23	7.5	4.3	1.3	0.8	31	11	29	190	218	83	69	132.4	6	49.1	460	17.2	49.7	18.8	264	7.5	0.88	145	4	103	9.1	5	84	12.7	14.7	0.9	1
74	F	151.2	43.8	19.16	20.7	60	154	70	98	76	69	7.4	4.6	1.6	0.7	31	18	15	148	258	96	108	130.8	5.3	59	443	13.8	41.5	20.2	301	7.4	0.71	146	3.9	103	9.5	3.7	91	11.8	12.3	0.8	0.6
82	M	154.3	50.1	21.04	15.6	76	156	85	108.7	66	97.8	6.9	4.1	1.5	0.7	22	12	34	261	132	62	49	70.6	6	66.7	471	14.5	43.2	20.7	368	6.9	0.78	142	5.1	104	9.1	4.9	61	11.9	12.2	0.7	1.5
78	F	135.9	40.3	21.82	28.8	69	116	62	80	66	30.3	8	4.8	1.5	0.6	31	16	22	242	163	93	56	88.4	4.9	62.3	461	14.9	45.6	20.6	331	8	0.49	147	4.2	105	9.5	4.3	80	14.2	14.4	0.7	1
58	M	163.7	60.5	22.58	20.7	82	100	59	72.67	77	9.9	7.3	4	1.2	0.3	20	23	75	360	117	157	28	57.6	6.1	63.8	492	15.1	44.7	26.7	283	7.3	1	145	3.7	107	8.8	6.7	121	9.1	8	0.6	0.7
64	M	165.6	79	28.81	33.4	94	143	83	103	61	7.8	7.3	4.2	1.4	1	51	70	105	190	158	123	39	94.4	5.5	54.1	459	15.3	43.6	26.2	244	7.3	0.76	143	4.2	102	9.1	5.8	65	11.1	13.5	0.7	1
64	F	148.1	52.6	23.98	30.5	74	124	77	92.67	66	5.8	7.2	4.3	1.5	0.5	20	14	20	235	232	83	58	157.4	5.7	38.6	410	13	38.4	26.6	292	7.2	0.67	144	4.9	104	9.4	3.8	113	9.3	11	1.6	1
58	F	141.7	53.6	26.69	32.4	75	121	69	86.33	92	4.1	7.6	4.9	1.8	0.6	27	26	36	483	242	108	73	147.4	5.2	51.3	457	14	41.4	28.6	305	8	0.5	144	3.7	102	9.5	4.5	62	14.3	14.3	0.8	0.8
49	F	158.4	53.5	21.32	28	66	152	80	104	76	33.9	7.8	4.2	1.2	0.9	20	17	15	156	243	77	102	125.6	5.1	51.8	471	14.7	43.8	25.8	305	17.1	0.83	141	3.9	101	9.1	4.6	76	16.3	13.2	0.8	0.7
69	M	160.1	54.3	21.18	16.2	75.5	128	68	88	61	2.5	7.2	4.5	1.7	0.5	26	32	32	295	271	197	72	159.6	7.8	60.8	454	15.1	44.2	37.8	326	13.9	0.91	139	4.7	98	9.4	5	86	15.7	14.1	0.9	0.6
65	M	166.9	67.4	24.2	22.5	85	126	66	86	51	20.9	8	4.3	1.2	0.8	29	22	48	208	168	65	71	84	5.4	60.6	477	16.2	47	24.8	368	11.7	0.68	142	4.7	103	8.8	6.5	60	10.6	11.4	0.8	0.9
70	F	148.5	49.6	22.49	29.3	66	102	51	68	84	14.8	8	4.1	1.1	0.6	45	37	23	383	152	85	49	86	4.9	44.3	398	14.1	39.2	19.9	228	17.9	0.56	144	4	106	9.6	5.4	115	13.4	13.8	0.9	0.7
72	M	161.8	67.1	25.63	25.1	91	157	87	110.3	74	7.6	7.2	4.4	1.6	0.6	26	29	48	273	165	100	42	103	5.5	88.2	487	15.2	45.1	31.3	326	14.4	0.92	145	4.3	104	9.4	5.2	79	10.8	10.6	0.9	0.9
66	M	158.5	50.1	19.94	20.6	70.5	103	60	74.33	72	5.8	7.1	4.4	1.6	1.1	37	29	33	166	222	63	96	113.4	5.7	53.9	482	15.4	45.7	23.6	257	13.1	0.64	145	4	104	9	4.9	89	13.3	13.1	0.7	0.7
67	F	142.5	41.3	20.34	26	64.5	130	62	84.67	65	21.6	6.8	4.3	1.7	0.5	36	42	24	243	173	102	69	83.6	6.6	54.7	344	11.3	32.7	25.2	384	12.5	0.52	145	3.6	104	8.9	2.8	72	12.9	10.5	1.2	1.4
69	M	163.3	69.3	25.99	24.9	86	179	87	117.7	67	15.1	7.4	4.4	1.5	0.7	26	21	29	212	234	192	44	151.6	4.5	79.8	477	15.2	43.8	33.1	331	13.6	1.02	146	4.7	104	9.1	5	59	9.9	11.2	1.3	1.5
61	F	152.5	42.8	18.4	20	61	129	79	95.67	66	46.7	7.3	3.8	1.1	1.2	27	23	15	247	230	69	130	86.2	4.8	51.9	461	13.9	42	25.1	315	18.9	0.57	144	3.5	102	8.7	4.4	135	13.1	13.1	1	1.1
59	F	155.6	70.2	28.99	41.9	80	123	66	85	72	8.7	7.4	4.3	1.4	0.8	24	22	27	282	211	40	77	126	5.2	53.9	481	14.9	43.5	25.3	331	16.7	0.63	142	4.1	100	9.3	5.9	79	17.3	17.6	0.8	0.7
69	F	157.7	62.9	25.29	34	85	120	55	76.67	66	10.3	8	4	1	0.5	19	17	18	222	169	106	36	111.8	5.3	60.1	408	13	38.2	33.5	550	9.3	0.69	142	3.6	100	9.3	5.3	67	16.1	15.1	0.9	0.9
67	F	148.1	58	26.44	40.1	91	132	88	102.7	82	2.2	8.1	4.7	1.4	0.8	31	30	34	262	167	228	35	86.4	5	72	430	13.6	40.6	14.6	236	16	0.69	143	4	103	9.3	4.9	89	13.8	13.7	0.7	0.7
50	F	160.6	55	21.32	27.9	63	155	92	113	82	12.4	7.7	4.8	1.7	0.8	17	10	21	195	240	61	98	129.8	5	41.2	477	14.9	44.1	21	267	16.7	0.57	143	4.2	102	9.5	3.9	83	14.7	17.2	0.6	0.8
66	M	171.3	75.2	25.63	27.2	99.5	128	72	90.67	72	4.6	8.1	4.6	1.3	0.8	29	32	64	187	196	131	50	119.8	5.2	79.8	468	15.8	44.7	31.8	275	15.3	0.8	143	4.1	103	9.3	7.2	65	13	13.3	0.8	0.8
67	M	164.6	77.8	28.72	28.3	88	136	69	91.33	67	30	7.7	4.6	1.5	0.8	26	32	56	166	168	358	34	62.4	4.9	48.6	444	15.4	42.8	19.7	260	15.1	0.93	142	4.3	102	10	8.9	104	13.7	16.4	0.8	0.8
72	F	145.7	47.5	22.38	35.2	69	130	79	96	74	24.4	8.3	4.4	1.1	0.4	24	15	56	381	196	196	59	97.8	5.2	67.8	379	12.5	37.4	34.4	264	21.6	1.11	142	4.4	103	9.8	5.7	116	11.3	10.2	1.1	1
70	M	176.1	69.1	22.28	18.1	83	154	71	98.67	64	14.8	7.8	4	1.1	1.2	16	10	61	311	213	106	53	138.8	6	110.9	406	13.8	41	22.3	663	16.8	0.93	141	4.5	101	8.9	6.1	47	7.8	7.6	1	1.1
59	M	161.3	59.9	23.02	21.2	85	150	90	110	67	5.9	7	4.3	1.6	0.9	22	17	28	178	211	105	55	135	5.4	64.2	489	16.4	48.1	28.3	349	11.8	0.67	143	4.7	103	9.7	5.3	76	14.2	15.9	0.8	1.1
66	F	159.1	59.9	23.66	34.6	75	123	65	84.33	68	22.8	7.3	4.3	1.4	0.6	17	16	13	312	181	119	58	99.2	5.3	59	440	13.7	40.9	19	375	13.6	0.54	143	3.9	103	9.2	3.9	65	13.4	14.2	0.6	0.7
58	F	155.7	47.5	19.59	23.4	62.5	96	51	66	76	25.7	7.1	4.4	1.6	0.5	20	21	21	263	251	226	45	160.8	4.7	78.5	394	13	37.5	26.5	296	14	0.57	145	3.7	104	9.4	3.3	156	13.8	13.5	0.5	0.7
65	M	162.3	58.1	22.06	18.2	76	113	53	73	74	85.9	7.4	4.5	1.6	0.7	15	13	24	124	181	86	54	109.8	5.7	64.4	511	15.9	46.1	29.6	250	11.4	0.69	143	3.9	103	9	5.5	83	13	16.1	1.3	1.2
63	M	161.9	60.2	22.97	20.7	78	139	82	101	86	3.1	7.3	4.2	1.4	0.5	24	16	32	212	209	98	95	94.4	5.3	43.1	503	16.3	48.2	23.5	264	14.5	0.89	142	4	103	8.9	6.1	154	12.4	11.2	0.7	0.5
63	F	143.7	48.5	23.49	33.9	71	120	76	90.67	77	14.5	7.3	4.6	1.7	0.5	28	28	17	227	210	131	45	138.8	4.8	48	471	14.4	42.4	22.2	320	16.5	0.73	144	5.2	106	9.3	4.9	59	16.1	14.5	0.9	0.7
51	F	152.5	47.4	20.38	23.8	62.5	103	59	73.67	79	3.3	7	4.4	1.7	0.7	19	12	13	124	215	48	117	88.4	4.8	32.7	442	13.4	40	23.5	337	11.4	0.61	145	3.7	104	9.4	3.5	79	11.7	12.6	0.7	0.6
71	F	149.3	66.5	29.83	37.8	80.5	134	65	88	62	11.9	7.7	4.5	1.4	0.7	18	18	37	229	191	306	41	88.8	7.6	69.9	401	12.9	37.7	29.8	343	8.3	0.47	142	4.1	101	9.5	4.6	66	18.2	19.8	0.9	1.5
65	F	150.2	44.9	19.9	25.3	65	105	57	73	58	7.2	7.8	4.4	1.3	0.5	25	16	20	215	218	170	71	113	5.4	60	418	13.4	41.2	37.4	315	15	0.67	144	4.3	103	9.4	3.8	127	10.2	11.6	0.8	0.8
46	F	150.8	49.1	21.59	27.6	64	104	50	68	77	18.2	7.6	4.7	1.6	0.7	17	23	20	186	183	60	65	106	4.9	57.5	451	14	41.7	33.7	228	14.9	0.48	143	3.9	103	9.2	4.1	34	15	14.8	0.6	0.6
69	F	148.9	48.8	22.01	18.6	63	111	58	75.67	54	20.1	7.3	4.4	1.5	0.7	19	15	19	262	211	70	70	127	5.5	55.5	468	14.7	44	24.5	337	12.2	0.57	146	3.9	105	8.5	3.7	115	11.7	11.9	0.8	0.8
65	M	166.9	74.4	26.71	24.5	90	137	54	81.67	53	7.5	7	4.5	1.8	0.8	20	21	33	213	204	109	44	138.2	6.3	60	466	14.9	44.8	18.7	337	16.6	0.82	144	4.2	103	8.8	5.5	69	12.9	15.3	0.9	1
64	F	160.9	49.6	19.16	26	69	130	57	81.33	89	8.7	6.9	4.4	1.8	0.8	26	23	19	157	236	115	66	147	5.6	41.5	422	13.6	40.9	18.1	331	15.1	0.63	145	4	104	9.2	4.3	80	14.3	12	0.6	0.8
67	F	156.7	64.7	26.35	33	88	140	83	102	63	83.7	7.3	4.1	1.3	0.7	21	16	16	251	208	83	71	120.4	5.5	43.6	446	13.6	41.8	27.2	361	13.9	0.58	143	3.6	102	8.8	5.6	84	14.6	11.4	1.1	0.8
57	F	155.8	58.2	23.98	31.3	72	137	81	99.67	58	3.3	7.2	4.3	1.5	0.5	16	9	22	163	219	153	56	132.4	5.5	37.6	388	12.7	37	29.6	236	15.2	0.56	143	4.1	104	9.3	5.6	81	9.4	8	0.8	0.7
68	F	149.3	59.3	26.6	37.2	77.5	109	56	73.67	58	6.7	6.7	4.3	1.8	0.7	23	19	28	192	240	158	70	138.4	5.2	44.2	405	13.1	38.8	27.9	292	19.9	0.62	144	4.5	105	8.8	4.6	89	13.8	12.5	0.5	0.7
68	M	170.1	64.8	22.4	19.1	81	121	76	91	58	7.2	6.8	4.1	1.5	0.5	17	14	22	176	195	80	68	111	5.3	60.4	469	14.8	44.2	22	271	17.2	0.84	144	4.5	105	8.8	7.1	72	14.7	12.3	0.9	0.8
64	F	150.1	47.8	21.22	30.8	65.5	111	75	87	64	10.2	7.5	4.3	1.3	0.5	31	19	15	334	181	163	51	97.4	5.5	48.5	517	12.6	39.9	19.8	247	20.4	0.53	143	4.3	103	9	4.6	71	12	10.7	0.5	0.6
53	M	163.4	59.9	22.43	20.5	75	133	78	96.33	66	6.9	6.6	3.9	1.4	0.4	21	9	27	181	231	248	49	132.4	5.2	70.5	444	14.6	42.9	36.5	414	11.3	0.68	142	4.6	105	9.1	6.6	78	7.8	8.2	0.7	0.8
67	F	160.5	58.8	22.83	34.1	72	101	64	76.33	66	40.4	7.5	4.6	1.6	0.8	26	20	25	168	205	89	76	111.2	5	52.7	423	13.5	40.7	23.3	287	9.2	0.67	145	4.2	104	9.4	4	98	7.2	7.3	0.7	0.6
68	M	166.9	76.3	27.39	28.8	93.5	133	84	100.3	55	28.1	7	4.3	1.6	0.8	18	14	29	173	213	132	53	133.6	4.6	39.2	505	16.9	48.7	18.3	275	18.2	0.76	141	4.2	103	8.8	6	102	13.6	13.5	0.7	0.7
52	F	152.5	43.9	18.88	22.5	63.5	109	50	69.67	56	10.8	7.5	4.4	1.4	0.6	19	18	19	200	217	142	48	140.6	5.4	47.5	438	13.5	41	21.7	267	15.5	0.61	144	3.5	104	9	3.2	63	12.7	12.6	0.8	0.7
66	M	168.1	66.9	23.68	26.3	81	117	75	89	65	4.6	7.3	4.7	1.8	0.7	19	22	35	170	229	97	59	150.6	5.2	63.9	528	16.1	48.1	22.2	244	15.3	0.93	142	4.1	102	9.4	4.4	92	11.5	14.1	0.7	0.8
64	F	147.7	44.1	20.22	23.2	63	121	61	81	69	24.3	7.3	4.4	1.5	0.5	17	8	8	96	163	58	62	89.4	5.2	49.5	406	12.9	38.1	25.9	231	10.1	0.5	140	3.9	103	9.1	4.1	103	12.8	11.5	0.7	0.7
66	M	165.7	57.6	20.98	21.4	78	117	65	82.33	66	65.7	6.9	4.1	1.5	0.7	19	14	29	195	266	214	64	159.2	5.4	70	438	14.5	43.4	30.4	320	10.9	0.94	145	4.2	107	8.9	5	99	14	16.4	1	0.9

64	F	152.9	51.5	22.03	29.1	70	135	71	92.33	72	5.9	8.1	4.7	1.4	0.5	25	17	30	218	199	160	58	109	5.1	57.2	452	14.9	43.2	20.3	279	11.5	0.56	145	4.8	105	9.6	3.6	74	13.8	12.5	0.7	0.8
62	F	154	46.9	19.78	23.9	60	154	78	103.3	107	27.1	7.2	4	1.3	0.6	26	17	17	191	196	66	50	132.8	5.4	111.3	390	12.6	38.7	22.7	513	9.3	0.55	141	3.9	100	9	3.8	107	13.6	12.8	0.7	0.8
61	F	158.9	57.5	22.77	32.6	70.5	152	101	118	60	14.9	7.6	4.5	1.5	1	28	21	20	312	244	95	64	161	4.9	44.5	477	13.6	43.1	27.9	461	13.1	0.6	144	3.7	104	9.2	4	113	9	11.2	0.9	0.9
61	F	154.7	49	20.47	23.2	73	141	80	100.3	71	18	7.8	4.7	1.5	0.6	20	13	23	183	269	155	55	183	5.1	44.4	393	11.6	35.4	23.6	250	15.7	0.59	144	4.3	105	9.7	3.5	70	12.6	14.8	0.6	0.9
62	F	142	55	27.28	38.5	71	149	81	103.7	67	11.3	6.4	4.2	1.9	0.4	20	15	18	207	198	127	47	125.6	5	73.6	421	14.1	42.3	19.2	283	20.6	0.58	144	4	109	8.7	4	96	17.3	11.6	0.8	0.8
62	F	147.7	57.6	26.4	36.2	76.5	117	46	69.67	71	13.9	7.9	4.7	1.5	0.6	24	25	47	149	245	126	64	155.8	5.6	61.7	434	13.1	40.4	20.9	331	16	0.71	143	4.4	103	10.2	7.8	113	14.2	11.6	0.6	0.6
66	M	172.5	77.9	26.18	28.3	95	125	60	81.67	58	12.7	7.1	4.6	1.8	0.9	28	26	87	236	239	160	57	150	5.6	51.7	521	16.3	50.9	18.6	244	14	0.87	144	4.2	106	9	7	53	9.4	9.2	0.7	0.6
55	M	164.9	68.8	25.3	23.1	83	124	68	86.67	65	4.6	7.3	4.7	1.8	0.8	16	18	56	198	252	104	66	165.2	5.8	39.7	496	16	48.5	22.1	257	11.5	0.73	144	4.3	105	9	2.9	121	15.1	13.4	0.7	0.6
67	F	150.5	57.9	25.56	39.8	79	113	59	77	51	33.4	7.2	4.3	1.5	0.5	17	13	24	167	234	175	60	139	6.2	68.5	498	14.7	43.9	30	239	17	0.62	143	4.5	105	8.9	6	100	13.3	14	1	0.8
62	M	165.5	60.8	22.2	19.8	72.5	131	72	91.67	59	2.2	7.9	4.6	1.4	0.6	32	31	34	177	244	283	48	139.4	5.1	44.9	440	14.3	42.5	20.3	375	13.5	0.61	143	4	102	9.4	5.2	68	8.4	10.3	0.7	0.7
72	F	151.3	45.2	19.75	24.4	70.5	133	75	94.33	80	50.9	6.9	4.1	1.5	0.4	23	23	14	386	223	96	77	126.8	4.9	67.2	406	12.1	38.1	19.8	292	18.1	0.75	142	3.9	106	9	3.3	147	10.6	9	0.8	0.9
74	F	152.8	55	23.56	27.6	73	148	81	103.3	69	40.5	7.2	4.1	1.3	0.6	20	14	22	205	225	94	56	150.2	5.3	76.3	482	14.5	45.3	19.7	337	14.6	0.68	144	4.1	106	8.7	5.3	73	12.1	10.7	0.8	1.1
58	M	155.9	50.9	20.94	20.5	76	141	78	99	59	24	7.3	4.4	1.5	1.5	20	27	38	265	225	101	63	141.8	8.8	54.1	498	15.7	45.3	18.5	175	15.2	0.77	142	3.4	98	9.1	5	45	13.2	15.9	0.8	0.7
69	F	151.8	46.5	20.18	25.9	66	113	58	76.33	95	23.8	7.6	4.6	1.5	1	18	9	23	245	239	68	57	168.4	5.3	151.9	412	12.3	38.6	19.3	483	11.3	0.69	140	3.8	100	9.3	3.4	51	12.4	12.9	0.8	1.2
58	F	159.9	57.8	22.61	29.3	73	120	61	80.67	57	5.3	8.6	4.6	1.2	0.6	17	12	12	229	193	120	45	124	5.1	78.7	479	13.9	42.5	21	226	15.8	0.74	142	3.9	103	9.7	7.3	63	12.1	11.9	1	1
63	F	147.9	46.6	21.3	25.2	69.5	103	72	82.33	72	26.7	7.3	4.5	1.6	0.9	21	4	16	190	195	89	50	127.2	5.4	46.7	462	12.7	41	18.9	216	11.8	0.6	143	4	104	9.2	4.6	58	12.8	13	0.6	0.9
63	F	154.9	49.9	20.8	26.5	69	128	75	92.67	59	8.5	7.6	4.9	1.8	0.7	24	23	24	220	226	109	66	138.2	5.6	50.3	465	13.7	43.4	18.3	260	15.1	0.59	142	3.4	102	9.4	5.8	104	12.7	13.7	0.6	1.1
69	F	154.1	56.1	23.62	31.7	64.5	127	64	85	91	28.8	7.5	4.7	1.7	0.8	19	10	13	182	180	75	66	99	5.2	44.5	435	13.3	41.5	25.7	283	13.4	0.56	144	4.2	104	9.2	4.6	91	17.1	14.7	0.7	0.8
61	M	171.2	71.6	24.43	20.2	88	137	84	101.7	63	17.2	8.1	4.6	1.3	0.9	42	37	22	224	264	104	69	174.2	4.9	70.6	488	15.8	48.6	19.1	305	16.3	0.87	142	4	103	9.5	6.4	192	15.8	14.4	0.9	0.8
62	M	170.4	74.5	25.66	25.1	88	130	69	89.33	67	3.7	7.9	4.8	1.5	0.5	29	40	28	265	220	130	40	154	5.3	62.9	557	15.8	48.4	15.9	305	11.4	0.76	143	4	101	9.3	6	76	13.4	13.8	0.5	0.8
63	F	153.4	53.2	22.61	27.2	73	146	92	110	69	6	7.4	4.7	1.7	0.6	23	19	22	236	216	117	62	130.6	5.4	59.4	480	14	44	24	271	10.9	0.49	143	4.1	102	9	3.2	86	9.5	12.1	0.7	0.8
67	M	155.4	60.9	25.22	21.9	85.5	109	57	74.33	53	6.4	7.4	4.4	1.5	0.5	17	18	29	169	196	405	36	79	5.3	72.7	473	15	45	20.2	310	16.3	0.8	144	4.4	105	9	7	90	15.7	14.2	0.7	0.9
64	M	155.6	59.2	24.45	26	75	179	100	126.3	59	15.3	7.1	4.3	1.5	1.3	21	17	21	240	168	112	53	92.6	5	75.7	502	14.7	46.5	22.2	287	12.9	0.72	141	4.5	101	9	6.5	90	11.9	18.6	0.8	1.1
67	F	155.7	51	21.04	28.1	71	124	71	88.67	68	11.1	7.6	4.6	1.5	0.7	18	14	18	220	214	73	68	131.4	5.8	52.6	449	13.4	41.8	24.8	301	21.1	0.6	142	4	102	9.9	5.4	67	10.2	10.2	0.7	0.8
57	F	153.3	44.2	18.81	24.1	58	138	69	92	89	14.4	8.3	4.7	1.3	0.5	20	21	18	308	197	111	52	122.8	5.2	51.5	498	14.2	43.6	20.2	305	16.8	0.63	144	3.7	105	9.4	4.6	100	13.8	12.4	2.1	1.2
63	M	169	68.5	23.98	24	81.5	131	76	94.33	52	16	7.4	4.5	1.6	0.8	19	15	12	173	202	173	43	124.4	5	55.5	487	13.9	42.9	23.1	301	14.1	0.87	141	3.8	103	9	6.1	68	17.5	14.2	0.6	0.7
62	F	155.6	53.2	21.97	26.7	67	138	92	107.3	60	23.9	7.1	4.5	1.7	0.8	25	22	29	204	253	104	63	169.2	5.2	50.2	441	13.6	42.9	24.2	337	15.4	0.71	145	4	105	9.1	4.5	102	12.9	13.2	0.7	0.8
60	M	168.1	54.6	19.32	17	72.5	151	83	105.7	77	4.6	7.7	4.3	1.3	0.7	35	29	123	274	210	592	41	50.6	5	57.1	468	15.5	47.1	24.9	275	10.7	0.72	146	3.8	104	9	7.6	78	15.7	14.6	0.9	0.7
62	F	154.6	49.5	20.71	27.3	66	131	71	91	69	9.5	7.3	4.5	1.6	0.9	14	12	15	228	229	71	69	145.8	5.3	56.8	485	13.8	44.2	20.9	239	16.2	0.6	145	4.4	107	8.8	4.2	58	12.7	13.6	0.8	0.6
67	F	148.2	62.8	28.59	32.7	86	128	64	85.33	62	9.5	7	4	1.3	0.6	19	17	11	188	215	114	50	142.2	5.3	51.6	376	12.3	38.9	16.2	384	14.2	0.61	144	3.8	106	8.5	5.2	60	8.3	13.4	0.5	0.7
71	M	165.6	52.2	19.03	15.4	71	114	75	88	79	20.5	7.4	4.3	1.4	0.8	14	14	26	200	169	83	48	104.4	5.5	45.6	460	14.3	42	18.1	349	15.2	0.75	143	4.2	105	9.1	4.7	92	13.8	15.5	0.9	0.9
69	F	154.9	46.9	19.55	21.8	65	97	54	68.33	66	18.1	6.9	4.3	1.7	0.6	21	16	17	185	225	70	92	119	5.5	49.1	431	13.8	42.3	21.4	228	14.7	0.65	144	4.2	108	9.7	5	89	7.6	7.9	0.6	0.7
65	M	162.6	73.1	27.65	23.9	97	149	89	109	63	3.8	7.7	4.5	1.4	1	43	45	39	184	172	158	56	84.4	4.9	52.7	446	14.5	43.6	17.7	264	10.5	0.81	143	4.3	104	9	6.1	98	7.8	9.3	1.2	1.6
60	M	173.3	71.4	23.77	22.6	85	103	58	73	48	22.6	7.1	4.4	1.6	1.5	24	19	18	165	205	63	55	137.4	5.2	37.1	504	15.9	49.1	14.9	267	14.8	0.9	145	4.6	108	8.8	6.2	77	15.2	12.2	1.2	0.6
68	M	162.7	52	19.64	13.7	66	121	64	83	63	16.7	7.8	5	1.8	1.4	26	18	26	214	257	76	110	131.8	5.3	38.7	491	15.1	46.4	17.7	212	20.5	0.77	145	4.6	104	9.3	4.8	108	12.7	12.6	0.6	0.7
48	F	160.9	71.2	27.5	30.7	78.5	94	52	66	54	43.1	7.3	4.2	1.4	0.5	15	12	15	122	197	77	50	131.6	5.4	36.5	392	9.6	31.7	25.7	231	14.8	0.69	144	4	109	8.5	2.9	53	11.3	12.6	0.6	0.5
61	F	156.6	58	23.65	36.8	81	125	69	87.67	62	8.3	7.5	4.3	1.3	0.8	19	15	16	190	239	128	56	157.4	5.3	56.7	473	14.2	43.9	23.3	343	12.1	0.58	145	4.3	105	9.3	4.7	90	13.4	12.3	0.6	0.8
64	M	174.9	69.4	22.69	21.5	86	186	91	122.7	63	40.9	6.9	4.3	1.7	1.1	25	14	30	186	169	189	50	81.2	5.2	60.2	463	14.7	44.9	19.3	315	13.8	0.79	144	4.2	105	9.1	4.7	98	18	33.8	0.6	0.7
70	F	150.9	53.9	23.67	32.1	74	107	54	71.67	60	7.2	7.6	4.6	1.5	0.5	32	32	41	238	191	229	41	104.2	5.7	75.7	429	13.1	40.1	27.4	271	10.4	0.59	141	4.2	104	9.4	5.9	52	12.7	12.3	0.6	0.8
74	M	161.4	66.2	25.41	27.4	87	118	63	81.33	63	12.3	7.1	4.4	1.6	0.8	20	14	42	176	193	144	47	117.2	5.5	38.6	500	14.5	43.5	14.9	264	11.3	0.79	143	4	106	9	5.8	121	10.1	9.8	1.2	1.2
73	F	148.1	48.3	22.02	28.2	72	168	85	112.7	73	47	7.5	4.6	1.6	0.8	20	16	10	203	195	84	81	97.2	5.1	63.3	385	12.3	37.5	22.8	247	12.4	0.63	144	4	105	9.6	4	58	9.2	14.1	0.8	0.7
71	M	169.3	64	22.33	22.2	79	98	64	75.33	60	34.2	7	4.3	1.6	1.1	21	11	19	181	235	79	59	160.2	5.3	44.4	489	14.1	43.7	35.4	310	12.2	0.77	141	4.3	104	9	5.2	121	12.5	13.1	0.9	0.7
69	F	151.3	53.2	23.24	27.9	68	121	65	83.67	66	4.7	7.7	4.5	1.4	0.5	20	9	12	224	264	124	48	191.2	5.7	58.9	430	11.3	35.5	26	384	16.6	0.79	141	4	102	9.1	4.7	87	7.6	7.7	1.1	1.2
51	M	172.1	62.7	21.17	17.8	78	124	74	90.67	62	2.1	7.9	4.8	1.5	0.6	31	31	39	263	203	101	51	131.8	5.8	64	598	19.3	58.2	17.9	447	16.1	0.63	141	5	104	9.1	4.8	72	9.2	11	0.5	0.7
57	F	152.5	63.5	27.3	33.1	79	157	71	99.67	56	5.2	7.6	4.6	1.5	0.6	18	17	16	144	259	145	50	180	5.4	49.6	422	12.7	39.1	21.2	226	11.5	0.74	145	4.2	106	9.6	4.8	93	16.6	16.9	0.7	0.7
74	M	169.3	56.6	19.75	20.3	79.5	128	62	84	75	4.6	7.3	4.4	1.5	0.8	19	14	26	176	146	83	43	86.4	7.8	89.9	476	14.4	43.9	21.1	253	14.1	1.01	146	3.4	106	9.4	6.2	68	12.2	9.6	0.8	1.8
72	F	147.7	52.9	24.25	29.6	76	140	77	98	62	45.7	7	3.8	1.2	0.6	18	13	18	291	243	106	66	155.8	6.2	63.6	381	10.4	32.4	20	320	10.1	0.48	145	3.9	110	8.5	3.9	69	13.8	15.2	0.7	0.9
73	M	158.2	64.1	25.61	20.4	83	126	66	86	57	14.2	8	4.6	1.4	0.8	34	42	70	224	252	107	70	160.6	6.9	55.4	463	14.1	42.6	19.1	292	10.3	0.78	144	3.7	104	9.2	7.5	51	10.5	11.6	0.9	0.9
53	F	161.1	62.1	23.93	29.9	67	137	72	93.67	49	18.7	6.9	4.3	1.7	0.7	15	10	16	159	234	61	79	142.8	5.3	51.2	426	13.5	40.8	19.6	275	10.5	0.59	144	3.8	104	9.5	4.2	81	14.2	14.5	0.7	1
80	M	155.3	57.3	23.76	14.1	76	137	84	101.7	71	197	7.7	4.4	1.3	0.6	37	25	88	179	177	66	86	77.8	5.6	52	423	13	40	22.7	296	15.1	0.98	143	4.1	104	9.8	6.7	153	7.9	7.7	0.9	0.7
50	M	171.7	67.6	22.93	19.3	75	133	86	101.7	50	50.9	6.7	4.1	1.6	0.7	18	13	22	214	176	78	37	123.4	5.4	59.5	454	13.9	41.7	19.7	343	17	0.88	144	4.1	105	8.8	5.5	63	16.6	16.1	0.7	0.8
51	M	171.9	53.6	18.14	12.8	66	113	64	80.33	58	14.1	7.5	4.6	1.6	0.9	21	19	55	228	231	59	102	117.2	5.4	49.8	499	14.3	42.6	25.1	236	9.2	0.75	142	4	102	9.2	5.1	99	11	8.6	0.6	0.7
65	M	163.8	70	26.09	27.6	84	126	64	84.67	59	13.4	7.9	4.1	1.1	0.5	29	27	68	231	171	166	47	90.8	5.3	44.4	491	15.5	46.3	24.3	403	14	1.02	144	4.2	105	9.4	5.8	87	7.8	8.9	0.9	1
70	M	150.8	52.8	23.22	22	74	158	109	125.3	65	13.1	7	4.5	1.8	1.1	17	11	24	190	189	87	62	109.6	5	46.8	521	16.1	46.8	20	292	13.2	0.71	145	3.9	106	9.2	6.1	99	17.2	16	1.1	0.7
55	F	158.7	48.9	19.42	22.4	63	104	64	77.33	53	26.8	7.1	4.6	1.8	1.7	18	13	14	153	173	63	67	93.4	5.2	52.2	400	12.4	37.4	18.8	305	10.4	0.56	145	4.1	106	9.5	3.2	99	9.4	9.1	0.8	0.7
66	F	146.3	52.8	24.67	35.2	77	115	59	77.67	69	11.6	7.1	4.4	1.6	0.7	32	28	32	219	204	106	48	134.8	5.5	45.1	428	13	38.9	20.2	337	18.3	0.77	144	4.7	108	9.4	5.2	77	16.2	14	0.7	0.7
64	M	164.9	68.6	25.23	25.6	92	141	68	92.33	54	66.4	6.9	4	1.4	0.5	23	22	25	170	210	100	49	141	5.3	82.3	459	15.5	45.1	20.5	292	10.4	0.77	142	4.4	106	8.6	3.9	62	7.6	7.7	1.3	1.7
65	M	182.4	63.2	19	14.6	68	124	67	86	70	7.4	7.2	4.5	1.7	0.7	19	15	30	218	206	72	87	104.6	5.5	61.4	455	13.9	41.9	25.4	355	17.6	0.78	142	4.4	103	9.5	5.3	117	14.6	14.2	0.7	0.6
63	M	163.4	63.9	23.93	25.1	84	148	76	100	60	40.9	6.7	4.1	1.6	0.4	25	17	35	234	182	52	67	104.6	5.9	46.4	429	13.8	41	23.6	305	13.7	0.71	142	4.6	105	9.1	6.5	74	10.2	11	1.2	1.5

63	M	168.1	49.6	17.55	15.1	68	148	72	97.33	50	28.6	6.9	4.5	1.9	1	36	28	69	186	186	69	102	70.2	4.7	50.3	475	16.1	47	20.8	219	14.8	0.62	144	4.7	104	9.3	6.1	51	14.7	16.1	0.8	0.7
65	F	152.9	52.4	22.41	29.8	77	118	59	78.67	63	11	7	4.4	1.7	0.7	16	11	13	215	266	199	58	168.2	5.8	53	444	13.6	40.9	20.1	315	12.9	0.7	144	4.3	105	9.1	4.6	76	10.2	10.5	0.9	1.1
65	F	143.9	51.5	24.87	27.5	72	124	69	87.33	58	48.7	7	4.2	1.5	0.5	20	14	14	266	182	143	47	106.4	5.4	54.3	429	12.7	38.8	15.6	271	10.5	0.6	145	3.8	106	8.8	5	79	16.5	14.5	1.2	0.7
55	F	147.9	50.3	22.99	28.8	64.5	116	63	80.67	63	6.6	7.3	4.2	1.4	0.6	19	15	10	160	166	112	56	87.6	5.1	54.8	393	12.6	38.3	22.8	279	17.2	0.46	142	4.1	103	9.3	3.8	98	19.6	18	0.7	0.7
51	M	165.7	53.5	19.49	16.6	71.5	148	80	102.7	61	29.3	7.3	4.5	1.6	0.5	29	30	75	199	161	58	91	58.4	5.1	60.3	454	14.1	42.6	26.5	260	15.1	0.75	141	4.2	102	9	7.1	80	12.2	12.7	0.8	0.6
53	M	170.5	73.5	25.28	25.2	86	115	72	86.33	60	4.1	7.5	4.4	1.4	0.7	20	16	22	158	218	74	71	132.2	4.4	72.8	475	14.9	44.8	25.9	326	11.8	1.1	143	4.4	106	9.3	6.6	73	10.6	11.5	0.7	0.7
52	M	167.8	71.3	25.32	25.1	82	129	76	93.67	49	9.1	7.2	4.7	1.9	1.1	25	20	109	230	178	137	62	88.6	5.7	62.6	464	14.7	44.7	25.7	331	19.2	1.13	143	4.3	106	9.6	7.6	55	11.5	13	0.7	0.5
53	M	150.5	40.9	18.06	14.5	63	103	52	69	45	30.5	7.2	4.5	1.7	0.5	38	35	28	218	193	104	59	113.2	5.5	73	489	15.9	47.2	22.7	221	21.8	0.78	145	5.3	106	9.3	5.3	96	11.9	10.3	0.6	0.6
50	M	174.9	61.6	20.14	19.7	77	136	74	94.67	70	2	7.5	4.9	1.9	0.7	20	19	27	232	229	66	98	117.8	4.8	44.9	516	16.6	50.2	26.1	315	7.2	0.84	142	4.1	102	9.5	4.7	88	13.9	15.5	0.8	0.8
58	M	167.9	61.9	21.96	24.3	83	152	91	111.3	77	2	7.8	4.8	1.6	0.5	24	25	59	241	252	98	89	143.4	5.3	59.4	482	15.3	45.4	23.8	315	14.2	1.09	143	4.4	104	9.6	6.9	107	20.5	19.6	0.7	0.9
54	F	166.1	58.4	21.17	24.4	68	120	64	82.67	65	17.6	7.5	4.5	1.5	0.8	27	27	31	161	181	49	76	95.2	4.8	34.6	422	13.4	40.2	11.4	247	14.7	0.79	143	3.7	104	9.7	4.5	124	10.4	9.8	0.6	0.7
57	M	174	77	25.43	24.7	86.5	167	93	117.7	63	21.6	7.2	4.5	1.7	1.4	32	26	65	150	188	287	44	86.6	4.6	59.5	505	16	48.2	18.8	283	14.9	0.88	146	3.9	107	9.3	6.5	44	10.2	13.9	0.5	0.8
68	M	172.6	73.7	24.74	19.9	90	128	80	96	59	49.6	6.9	4.2	1.6	1	30	22	101	282	192	52	70	111.6	4.3	49.6	454	15	43.2	22.6	242	11.7	0.84	143	4.1	106	8.8	5.3	73	8.6	11	0.7	0.8
55	F	150.4	39.1	17.29	19.3	64.8	112	62	78.67	88	14.4	7.5	4.5	1.5	0.5	22	14	15	197	238	45	117	112	4.8	47.3	458	13.9	41.4	21.3	317	11.2	0.7	142	4.3	104	9.6	4.1	170	12.4	13.1	0.6	0.6
57	F	150.5	62.6	27.64	40.3	80.5	137	81	99.67	62	4.3	7.4	4.5	1.6	0.9	25	32	20	146	209	78	70	123.4	5.2	61.5	435	13.9	41.3	18.4	328	17.1	0.61	141	3.7	102	9.4	9	48	12.6	18.5	0.6	0.6
67	F	143.9	53.9	26.03	36.3	80	125	64	84.33	80	3.2	8.1	4.5	1.3	0.7	19	19	22	212	193	86	56	119.8	6.6	65.1	446	13.6	41.7	28.4	439	24.4	0.63	143	4.5	107	9.5	5	75	10.7	12.6	0.8	0.8
64	F	154.7	56.1	23.44	29.1	73.5	146	81	102.7	70	64.8	7.7	4.2	1.2	0.8	19	13	15	214	186	72	64	107.6	5.5	47.9	420	12.8	38.8	19.9	250	14.4	0.74	143	3.9	106	9.1	4.4	37	8.5	11.1	0.5	0.7
66	F	153.7	59.4	25.14	31.8	77	126	69	88	64	8.5	7.4	4.9	2	0.8	27	29	28	207	199	137	71	100.6	6.6	57.5	472	14	43.1	17.4	269	15.1	0.82	145	4.2	106	9.4	5.7	96	23.9	24.5	1.3	0.8
63	F	150.5	58.4	25.78	33.6	72.5	124	75	91.33	74	4.2	7.4	4.4	1.5	0.7	17	14	17	189	248	76	57	175.8	5.1	51.9	445	13.5	40.3	22.1	360	22.5	0.68	143	4	106	9	4.6	54	13	12.6	0.8	0.7
65	M	168.1	65.5	23.18	21.6	80	148	78	101.3	56	10.8	7.2	4.5	1.7	0.4	21	19	30	386	134	92	49	66.6	6.4	65.9	474	14.9	44.6	22.6	353	14.2	0.93	141	4.3	101	9.3	5.2	66	18.1	13.9	0.8	0.7
46	F	157.3	62.9	25.42	34.3	80.5	109	65	79.67	62	5.9	7.4	4.2	1.3	0.8	14	11	32	169	170	111	80	67.8	4.8	61	476	14.3	43.6	8.6	254	9.3	0.67	141	3.7	103	9	5.1	43	13	12	0.5	0.5
80	M	163.1	73.3	27.55	23.4	101	133	72	92.33	74	48.2	7.5	4.4	1.4	1.1	29	26	44	161	211	160	53	126	4.8	55.7	449	14.3	43.3	15.6	242	16.2	0.58	143	4.1	102	9	5.6	39	9.1	9.5	0.5	1.2
52	M	172.1	57.5	19.41	14.3	67.5	112	75	87.33	51	7	7.4	4.8	1.8	1	18	11	35	195	238	62	78	147.6	5	41.5	482	14.9	45.4	18.5	286	12.5	0.77	144	4.6	103	9.9	6.1	94	10.8	8.7	0.3	0.4
54	F	154.4	51.1	21.44	26.4	70	102	55	70.67	51	7.2	7.1	4.5	1.7	0.9	13	12	18	253	261	59	75	174.2	5.6	49.7	441	13.4	40.1	30.7	257	14.4	0.62	143	3.9	106	9.4	4.1	62	10	8.9	0.4	0.5
64	F	163.1	68.5	25.75	30.5	86	128	79	95.33	60	32	7.2	4.3	1.5	0.6	25	23	17	238	226	110	57	147	5.2	50.5	395	12.1	36.2	20.8	322	14.6	0.57	144	3.9	106	9	4.8	46	11.5	10.8	0.6	0.7
55	M	183.3	80	23.81	24.1	87	145	83	103.7	71	3.5	7.6	4.7	1.6	1.1	19	21	30	241	207	206	44	121.8	5.4	61.6	558	16.6	47.5	22.7	273	14.4	1.01	144	4	105	9.3	6.8	75	12	10.6	0.6	0.7
68	F	149.7	54.2	24.19	28.6	78.5	143	83	103	72	13.5	7	4.3	1.6	0.8	19	17	17	239	227	72	60	152.6	5.3	71.1	434	12.8	38.8	19.4	239	16.7	0.63	142	4.4	107	9	3.8	77	16	16.8	0.7	0.9
67	F	155.6	59.9	24.74	31.5	77	149	89	109	56	46.9	7.3	4.6	1.7	0.6	16	11	15	226	195	125	52	118	5.7	64.9	443	13.3	40.5	28.9	328	11.2	0.73	143	4.1	104	9.6	5.1	64	16.6	13	0.5	0.8
57	F	155.9	49.9	20.53	23.3	61	183	89	120.3	62	91.2	7.4	4.5	1.6	0.6	17	13	13	174	196	59	63	121.2	5.2	62.1	456	13.9	41.5	23.2	311	13	0.7	144	4.6	106	9.3	4.2	66	16.6	19.3	0.7	0.9
54	M	166.3	62.1	22.45	20.2	79.5	133	87	102.3	48	25.7	7.5	4.4	1.4	1.1	27	29	63	225	183	112	47	113.6	5.3	41.7	477	15.5	43.4	20.8	209	12.1	0.72	140	4.4	103	9.3	6.4	51	9.7	12.8	0.6	0.9
49	M	169.3	63	21.98	17.5	71	136	74	94.67	45	27.3	6.8	4.5	2	1.2	19	16	21	179	179	58	52	115.4	5.1	75.3	436	15.6	46.9	20.5	311	9.8	0.82	142	4.5	105	9.4	5.9	74	13.9	11.7	0.6	0.7
62	F	161.3	42.7	16.41	15.5	61.5	107	60	75.67	57	15.1	7.4	4.4	1.5	0.8	24	22	41	228	226	90	71	137	6.3	54.5	469	14.1	42.6	23.3	301	12.4	0.51	141	4.7	103	9.6	4.9	80	13.4	12.5	0.8	0.9
56	F	151.3	54.7	23.9	27.9	79	119	78	91.67	70	11.4	7.5	4.6	1.6	1.1	19	17	15	250	200	55	64	125	4.6	40.8	416	13	38.5	21.6	301	17.9	0.67	143	4.1	103	9.3	3.8	46	12.1	13	0.7	0.6
66	M	166.9	52.6	18.88	16.6	72.5	113	55	74.33	55	12	6.3	4.2	2	1.1	25	16	64	262	201	98	78	103.4	4.8	47.1	438	14.7	44.3	25.2	286	13.1	0.65	141	4.7	105	8.9	5.4	53	7.3	8.1	0.6	0.6
61	F	149.4	44.8	20.07	25.8	66	132	78	96	92	4.5	7.6	4.6	1.5	1.1	28	26	29	231	206	80	48	142	5.1	41.1	485	14.3	43.1	19.3	317	11.2	0.63	143	3.6	104	9.4	4.7	63	13.8	14.6	0.6	0.6
57	M	171.7	67.9	23.03	24.1	83	159	87	111	80	12.9	7.3	4.2	1.4	0.8	15	14	35	171	245	160	55	158	6.3	67.6	448	14	42	24.7	306	15.6	0.75	142	4.2	106	9.8	4.6	60	11	12.3	0.6	1.1
58	F	154.9	60.8	25.34	33.4	78	156	82	106.7	71	8.8	7.1	4.5	1.7	0.6	18	14	21	301	210	187	37	135.6	5.5	45	490	14.5	43.5	25.4	273	11.3	0.55	143	3.8	107	9.5	5.3	60	13.1	13.2	0.7	0.7
56	F	145	45.5	21.64	33.7	66.5	120	70	86.67	70	19	8	4.7	1.4	1.1	29	21	24	143	182	89	87	77.2	4.9	48.5	437	13.6	41.1	26.8	306	11.9	0.48	141	4.2	103	9.7	4.2	101	16	14.9	0.6	0.8
65	M	172.8	64.8	21.7	15	83	146	78	100.7	69	22.3	7.5	4.5	1.5	1.1	25	19	32	146	173	119	54	95.2	6.3	72	399	13.7	40.5	30	282	15.5	0.81	142	4.1	103	9.4	7.5	75	14.8	20.3	0.8	0.7
66	F	160.1	61.5	23.99	29.7	78	174	104	127.3	75	32.6	7.3	4.2	1.4	0.6	24	18	28	228	179	107	63	94.6	5.5	57.2	489	14.5	43.6	23.3	347	15.4	0.56	143	3.9	104	9.3	3.9	70	9.4	11.3	0.6	0.7
67	M	161.3	52.6	20.22	18.2	76.5	143	80	101	58	25.5	7.9	4.7	1.5	0.9	23	18	32	166	259	86	107	134.8	5.9	73.7	483	14.7	45.2	20.9	273	12.2	0.7	142	4.1	101	9.7	6.5	69	13.1	13.7	0.7	0.5
62	M	169.3	71.7	25.02	24.8	90.5	102	60	74	55	4.2	7.7	4.5	1.4	0.7	29	43	46	384	215	225	40	130	5.4	63.8	540	16.4	48.3	33.2	250	12.4	0.83	142	4.5	107	9.7	7.5	71	14.6	12.5	0.6	0.6
63	M	163.6	64.5	24.1	18.6	83	120	65	83.33	60	30.4	6.6	4.2	1.8	0.6	21	22	90	142	151	92	72	60.6	4.9	66	362	12.7	36.8	24.4	203	14	0.75	142	4.1	108	8.7	7.8	59	10.2	14.4	0.7	0.7
64	F	163.3	58.7	22.01	21.9	81	134	71	92	75	21.2	7.8	4.5	1.4	0.7	24	19	20	358	222	277	46	120.6	6.9	51.7	470	14.2	42.2	22	250	12.9	0.63	141	4.2	101	9.7	6.7	56	13	14.5	0.9	0.7
51	M	167.1	58.8	21.06	16.7	77.5	101	56	71	41	14.8	6.7	4.4	1.9	0.8	19	16	42	190	164	79	60	88.2	5	56.1	500	13.9	43.3	22	306	11.3	0.84	142	4.3	105	8.9	5.1	96	11.5	10.1	0.6	0.6
53	M	160	43.6	17.03	12.9	60	101	64	76.33	65	8.1	7.2	4.1	1.3	0.9	23	24	36	163	183	104	83	79.2	5	68.4	487	15.7	47	22.3	192	8.7	0.77	143	4.1	105	9	6.3	75	12.8	12	0.7	0.7
54	M	169.3	74.9	26.13	22.4	85	133	93	106.3	75	14	7.1	4.5	1.7	0.6	31	42	257	230	172	269	43	75.2	4.9	62.7	429	15	43.7	19.6	265	11.8	0.94	144	4	107	9.2	7.7	35	11.5	11.3	0.6	0.6
57	F	146.2	62.3	29.15	42.8	101	112	74	86.67	65	24.5	7.1	4.3	1.5	0.5	15	15	16	173	216	75	54	147	5	49.4	451	13.8	41	25.1	286	11.4	0.61	143	4.4	108	9.2	4.4	41	16.3	14.6	0.7	0.7
55	M	171.2	79.8	27.23	34.1	90.5	123	63	83	63	4.1	7.5	4.7	1.7	0.6	24	29	46	215	243	199	51	152.2	5.6	77.2	506	15.8	46	27.4	239	14.7	0.7	140	4.8	104	9.8	7.6	77	10.5	9.8	0.6	0.6
69	M	164.1	56.7	21.06	13.3	70	117	60	79	46	31.7	7.8	4.7	1.5	0.6	27	23	20	203	165	89	52	95.2	6	41	489	14.4	43.4	17	178	15.5	0.7	140	4.2	105	9.5	6.4	72	14.6	15.3	0.7	0.7
64	F	146.9	50.7	23.49	34.9	85.5	130	66	87.33	53	7.7	7	4.4	1.7	0.9	23	29	41	184	210	109	64	124.2	5.9	85.3	430	14.1	41	21.9	334	15.3	0.69	141	4.3	104	9.4	6.2	118	14.7	16.7	0.6	1.2
48	M	169.9	68.6	23.76	25.9	83	165	92	116.3	41	8.1	5.9	3.9	2	0.6	32	28	223	178	191	153	67	93.4	6	59.7	531	16.7	49.2	22.2	301	22.2	0.7	144	4.2	110	8.9	6.2	31	19.1	15.6	0.7	0.7
58	F	151.8	54.6	23.69	33.2	80	129	72	91	62	18.3	7.4	4.4	1.5	1	20	14	16	246	197	92	61	117.6	5.3	64	472	14	41.4	19.2	311	12.6	0.63	143	4	106	9.2	5.2	42	14.3	13.6	0.6	0.7
63	F	156.3	61.6	25.22	35.1	91	117	64	81.67	75	3.6	7.8	4.6	1.4	1.3	28	29	54	235	230	92	83	128.6	5.3	54.7	456	14.5	43.9	38.9	296	15.3	0.82	143	4.2	106	9.6	3.7	66	14.9	12.7	0.5	0.5
67	M	168.5	59.3	20.89	17.3	77	134	80	98	66	58.1	7	4.3	1.6	1.1	22	15	26	186	173	63	51	109.4	5.1	44.7	447	14.1	41.7	20.4	353	17.7	0.91	140	4.1	105	8.8	5.7	53	13.7	15.7	0.6	0.7
48	F	163.3	59.8	22.42	25.7	77	96	59	71.33	55	42.5	6.7	4.5	2	0.7	17	14	16	126	212	74	58	139.2	5.1	51.1	444	13.5	40.8	25.1	205	9.6	0.52	144	3.9	109	9.4	4.2	70	9.5	12.5	0.7	0.7
63	F	156.5	63	25.72	34.2	85	132	80	97.33	95	2.4	8.2	4.8	1.4	0.5	29	38	96	248	190	271	51	84.8	5.7	93.7	500	15.4	46.3	37.3	317	13	0.77	143	4.4	105	10	7.3	92	15.2	13.7	0.5	0.5
63	F	157.3	64.1	25.91	32.6	89	117	56	76.33	57	2.8	7.1	4.3	1.5	0.4	16	12	13	211	245	148	44	171.4	5.2	66.5	464	14.2	42.6	23.5	269	13.6	0.63	144	4.3	107	9.1	3.6	56	8	7.5	0.6	0.6
55	M	162.5	70.2	26.58	22	84.5	104	58	73.33	64	7.4	7.9	5.2	1.9	0.7	28	31	22	150	214	70	54	146	5	56.2	507	15.5	44.3	17.7	291	14.7	0.95	144	4.7	105	9.9	6.5	46	17.1	16.4	0.8	0.6

55	M	163.3	66.8	25.05	24.3	85	120	71	87.33	69	3.2	7.6	4.5	1.5	0.7	30	25	276	303	190	77	64	110.6	5.1	49.4	469	14.8	44.9	21.9	317	13	0.73	143	5	106	9.2	6.2	48	8.8	9.7	0.6	0.6
61	M	168.2	56.8	20.08	18.9	79.5	129	73	91.67	67	28.3	7.1	4.6	1.8	0.7	17	11	27	216	184	70	64	106	8.7	74.5	459	15	43.9	26.4	296	21.2	0.75	142	4.8	102	9.4	5	58	9.6	11.5	0.6	0.7
51	M	165.3	63.8	23.35	21.5	78	113	64	80.33	63	2.7	7.4	4.8	1.8	1.1	20	24	30	206	229	181	53	139.8	5.3	100.5	524	16.1	45.9	19.5	328	12.8	0.79	143	4	106	9.5	5.4	52	11.9	9.9	0.8	0.7
72	F	159.4	49.2	19.36	19.1	66	141	80	100.3	61	25	7	4.2	1.5	0.8	32	18	14	220	207	65	81	113	5.2	43.8	398	14.2	39.2	15.3	334	27.6	0.83	144	5.2	106	9.5	6.6	79	9.9	9.9	0.8	0.8
65	F	154.9	52.4	21.84	25.1	83	133	67	89	69	13.7	6.9	4.1	1.5	1.2	19	37	25	94	206	130	72	108	5.3	69.7	416	13.1	38.7	26.4	306	19.6	0.77	144	4.5	105	9.2	3.3	162	12.9	12	1.1	0.9
59	M	156.5	57.4	23.44	25.7	86	118	72	87.33	38	5.5	7.4	4.6	1.6	0.6	29	35	59	373	170	87	56	96.6	6.3	48.3	494	14.5	43.9	22.9	296	16.2	0.75	144	4.4	107	9.4	6.9	67	8.3	9.1	0.7	0.9
62	F	154.2	53	22.29	26	75.5	114	66	82	69	34.5	7.2	4.4	1.6	1.1	30	27	17	244	179	96	49	110.8	4.6	49.9	452	13.8	40.1	7.3	224	11.1	0.7	144	3.9	108	9	5.7	56	19.1	16.2	0.7	0.8
65	M	157.1	58.6	23.74	24.3	86	126	65	85.33	61	11.2	7.2	4.4	1.6	1.1	25	18	91	180	165	112	59	83.6	5.2	56.8	390	12.2	36.5	19.5	340	25.7	0.9	140	4.5	108	9.3	6.6	77	8.6	10.2	1	2.1
62	M	164.4	66.3	24.53	21.1	84.5	151	70	97	54	20.6	8.2	4.9	1.5	0.7	27	35	30	125	211	181	41	133.8	5.6	42	456	14.8	44.2	17.8	233	16.3	0.71	145	3.8	105	9.7	6.2	67	14.5	15.4	0.5	0.6
69	M	175.5	68.1	22.11	17.4	84	157	89	111.7	80	31.8	7.1	4	1.3	0.8	22	14	51	175	285	383	49	159.4	8.4	48.5	465	14.7	43.3	12.5	286	14.5	0.86	141	4.6	104	9	5.7	112	9.4	11.4	0.7	0.6
50	M	163.5	69.6	26.04	25.2	87	120	63	82	54	6.7	7.4	4.2	1.3	0.8	37	43	259	164	215	219	48	123.2	5.2	74.6	504	16.7	49.6	16.5	360	12.6	0.93	143	3.9	107	9.7	7.8	95	8	8.6	0.6	0.6
66	F	141	49.9	25.1	30.9	76	102	60	74	71	30.4	7.4	4.5	1.6	1	20	9	17	168	211	109	71	118.2	5.5	54.9	370	11.3	34	23.4	317	14.6	0.75	146	4.8	110	8.7	5.4	105	12	10.5	0.5	0.6
66	F	142.5	43.4	21.37	37.2	76	175	72	106.3	92	23.3	8.3	4.6	1.2	0.5	22	16	31	238	220	99	61	139.2	5.3	90.4	417	12.8	39	29.4	233	13.8	0.67	146	4.7	111	9.5	5.7	105	16.5	15.4	0.7	0.6
68	M	167	75.6	27.11	19.9	87	129	61	83.67	47	55.8	7.1	4.4	1.6	1.6	21	12	36	260	158	143	41	88.4	5.1	62.2	512	16	46	20.5	277	13.9	1.26	144	4.4	108	9.4	7.8	97	14.6	15.1	0.7	0.6
65	M	162.2	55.6	21.13	19.9	79	141	88	105.7	58	27.1	7	4.5	1.8	1.3	22	16	16	212	240	97	68	152.6	5.4	50.6	514	15.6	47.8	15.3	247	13.8	0.87	146	4	104	9.6	5.9	91	12.1	11.9	0.6	0.7
64	F	151.3	45.5	19.88	20.8	63	109	65	79.67	56	31.8	7.3	4	1.2	0.8	23	17	32	238	150	34	58	85.2	5.4	40.4	397	11.7	35.9	13.6	282	12.3	0.54	143	4.1	104	9.1	4.2	55	11.3	12	0.5	0.6
52	M	177.3	89.4	28.44	27.2	95	112	64	80	62	13.2	7.4	4.3	1.4	0.8	37	48	32	297	173	98	57	96.4	5.4	63.1	531	16.4	49.9	15.8	171	13.6	0.81	144	4	106	9.3	7.3	53	9.7	10.7	0.7	0.6
50	M	160.3	62.9	24.48	21.1	77.5	119	76	90.33	54	23.3	7	4.3	1.6	0.5	17	14	39	185	159	56	55	92.8	5.1	51.1	469	13.8	41.9	26.1	306	15.1	0.85	145	3.8	105	9	5.8	121	9.5	14.7	0.8	0.8
55	M	167.3	69.6	24.87	24.8	87	122	74	90	68	17.8	7	4.3	1.6	0.9	27	56	33	161	184	206	36	106.8	5.6	61.8	481	14.9	44.7	17	241	13.9	0.93	144	4	107	9.5	6.3	81	14.5	13.5	0.6	0.6
54	M	166.5	90.4	32.61	36.7	107	117	60	79	83	4.3	7.8	4.6	1.4	0.8	32	49	105	223	205	212	50	112.6	5.9	55.2	472	15.3	46.4	16.2	228	12.5	0.66	141	3.9	105	9.2	6.8	67	16.3	19.5	0.6	0.7
68	F	151.3	46.6	20.36	19.1	68	108	60	76	65	118	6.4	3.9	1.5	0.5	27	17	12	176	175	70	74	87	4.3	28.2	372	12.4	37.9	17.5	273	14.9	0.6	144	4	108	8.7	4.1	76	13.5	12.9	0.7	0.7
65	F	148.9	48.2	21.74	25.4	80	129	57	81	58	74.1	7.5	4.7	1.6	2.1	24	20	46	198	187	87	74	95.6	5.2	65.9	384	12.3	37.7	23.9	233	13.8	0.58	142	3.9	104	9.6	5.2	39	14.6	13.3	0.7	0.7
68	F	146.1	47.5	22.25	26.9	74	123	62	82.33	69	33.7	7.3	4.4	1.5	0.9	21	15	32	196	222	130	57	139	5.8	66.7	425	12.9	40.8	23	340	15.1	0.61	144	4	104	9.9	5.8	71	10	10.2	1	0.9
51	M	171.3	60.6	20.65	14.7	73.5	151	89	109.7	47	11.7	6.7	4.4	1.9	1	15	10	39	196	193	141	73	91.8	4.6	48.9	433	14.3	43.7	20.2	265	12.9	0.87	142	4.6	108	9	5.5	48	16	15.2	0.6	0.6
51	F	156.1	45.8	18.8	21.7	66.5	111	76	87.67	55	22.4	7.7	4.5	1.4	0.9	14	11	14	221	162	51	68	83.8	5	37.9	396	11.7	37.1	11.5	221	9.4	0.66	142	4.4	108	9.1	2.8	82	11.2	13.5	0.5	0.6
47	M	167.3	66.8	23.87	24	85	145	85	105	61	7.3	7.4	4.6	1.6	0.5	42	36	311	154	214	196	50	124.8	5.1	77.5	473	15.4	46.1	20.8	250	10.8	0.67	143	4.3	105	9.7	8.3	45	12.5	14.5	0.5	0.5
48	F	155.6	51.9	21.44	29.6	71	105	50	68.33	63	17.4	8	4.4	1.2	0.6	15	14	18	152	204	145	45	130	5.3	81.3	414	10.9	34.8	39.9	286	9.6	0.63	141	4.7	107	9.4	3.6	60	10.5	9.2	0.6	0.6
63	F	152.2	53.2	22.97	26.6	79	120	61	80.67	60	23.4	7.3	4.2	1.4	0.8	18	17	21	239	231	165	48	150	5.8	64	426	12.9	40.5	30.1	347	11.8	0.65	144	4.1	107	9.3	3.9	75	13.2	13.8	0.6	0.5
66	M	166.4	55.3	19.97	16.3	68	126	71	89.33	51	10.8	7	4.4	1.7	0.5	22	14	47	268	207	88	57	132.4	4.7	45.1	428	13.6	40.7	21.7	322	15.7	0.77	141	4.4	102	9.3	5.3	88	7.9	8.3	0.7	1.2
65	F	154.2	45.7	19.22	20.9	69	109	54	72.33	59	34.4	7.3	4.4	1.5	0.4	28	13	18	143	216	73	74	127.4	5.2	49.8	437	13.1	39.4	22.1	239	15.8	0.64	144	4.5	108	9.4	4.7	70	11.2	15.7	0.6	0.7
64	M	154.6	57.5	24.06	24.5	78	156	56	89.33	56	15.2	7.1	4.6	1.8	0.5	21	20	30	175	200	88	46	136.4	6.3	60.3	407	13	39.6	18.1	273	20	0.86	144	4.1	106	9.1	5.2	71	11.9	13	2.1	1.6
63	M	161.3	69	26.52	22.7	88	156	78	104	54	30.1	7.4	4.4	1.5	0.7	20	13	56	86	199	172	57	107.6	5.3	65.1	468	13.9	42.1	19.3	347	16.7	0.79	145	3.9	110	9.1	7	62	17	16.8	0.5	0.7
66	M	171	64.5	22.06	17.7	79	120	54	76	43	23.9	7.1	4.2	1.4	0.9	30	28	26	137	201	108	90	89.4	6.5	61.7	447	14.7	45	22.1	212	13	0.73	140	4.8	104	8.7	5.1	137	14.4	14.4	0.8	1.1
62	F	162.5	56.9	21.55	27.5	71	131	81	97.67	59	14.8	7.9	4.5	1.3	0.8	22	15	15	316	287	47	93	184.6	5.5	54.1	456	13.4	42.3	21	347	12.8	0.63	142	3.8	103	9.5	4.1	90	15.7	15.3	0.7	0.8
67	M	168.9	63.1	22.12	22	84	117	58	77.67	49	9.7	6.8	4.1	1.5	0.8	21	15	20	168	178	63	57	108.4	5.2	60.1	508	13.7	43.1	22.5	226	14.4	0.85	144	4	109	8.6	5.6	125	7.6	7.4	0.6	0.9
68	F	157.5	52.2	21.04	23.7	73	135	85	101.7	78	34.1	7.5	4.3	1.3	1.1	18	12	19	218	196	168	54	108.4	5.3	64.2	390	12	38.5	22	265	14.7	0.63	141	4.3	107	9.1	4.3	60	14	14.8	0.7	1.1
49	F	159	50.9	20.13	21.6	67	116	68	84	57	77.7	7	4.2	1.5	1.2	17	14	20	135	221	48	71	140.4	5.4	40.7	468	10.9	36.1	26	250	13.8	0.63	141	3.6	106	8.5	3	46	7.7	7.1	0.7	0.8
36	M	161.3	55	21.14	23.3	75	144	88	106.7	57	3.7	7.1	4.8	2.1	0.7	17	13	15	175	185	105	55	109	4.9	87.9	511	16.3	48.9	25.1	334	16.6	0.73	141	4.3	105	9.7	5.1	61	11.4	10.9	0.7	0.6
60	M	171.4	76.9	26.18	20.4	87	136	79	98	50	26.7	7.7	4.5	1.4	1.6	27	29	22	160	232	134	58	147.2	5.1	56.1	455	14.2	43.8	16.8	273	10.8	1.06	145	3.8	107	9.2	6.1	57	15.1	11.6	0.7	0.8
66	F	153.2	62.5	26.63	33.7	92	151	80	103.7	61	28	7.3	4.5	1.6	0.7	18	12	15	301	202	135	53	122	5.5	58.6	466	14	42.6	24.9	250	11.5	0.53	143	4.2	106	9.4	4.5	76	16.3	13.6	1.1	1
54	M	166.5	68.2	24.6	23.5	82	137	81	99.67	69	15.8	7.2	4.6	1.8	0.8	19	24	33	174	227	331	30	130.8	5	99.1	516	15.5	46.5	22.8	282	12.4	0.84	142	4.1	105	9.4	5.5	51	14.6	14.8	1.1	0.8
57	F	151.9	40.2	17.42	18	67	95	46	62.33	60	66.7	7.6	4.5	1.5	0.7	18	13	16	290	236	72	75	146.6	4.9	44.1	407	12	36.8	19	286	12.5	0.52	144	3.7	105	9.2	3.4	72	12.3	13.3	0.7	0.7
57	M	162	68.1	25.95	25.6	84	141	75	97	57	6.3	6.7	4.4	1.9	0.3	22	28	118	217	203	243	50	104.4	5.5	54.5	467	14.6	44.9	32.8	265	16.9	0.79	143	4.3	104	9.3	6.1	88	11.1	12.1	0.9	0.8
54	F	146.9	49.5	22.94	28.3	74	107	58	74.33	57	4.7	7.5	4.6	1.6	0.9	16	13	17	217	263	89	97	148.2	5.1	49.5	464	14.6	43.7	29	296	12.7	0.57	144	4.4	104	9.4	5	71	8	8	1	0.8
51	M	168.1	56.3	19.92	17.1	76	138	79	98.67	73	14.3	7.2	4.5	1.7	1.6	25	22	92	166	222	285	92	73	4.6	58.3	515	15.6	47.3	25.1	206	13.4	0.79	145	4.2	104	9.2	7.4	46	12.1	9.7	0.7	0.6
50	M	166.9	73.8	26.49	22.8	86	116	67	83.33	61	5.2	7.3	4.7	1.8	0.7	22	31	32	755	180	93	57	104.4	5	35.2	516	16.9	50.1	23.4	223	12.5	0.85	144	4.4	104	9.5	6	96	14.3	13.3	0.8	0.7
53	M	158.1	49.1	19.64	16.9	74	120	78	92	61	31.7	7.3	4.4	1.5	1	22	20	30	206	179	82	73	89.6	5	86.4	456	15.1	44.4	27.2	328	11.6	0.79	143	4.1	105	9.3	5.6	83	17.5	17.2	0.6	0.7
57	F	155.4	62.1	25.72	37.6	76	128	56	80	61	4.6	7.9	4.5	1.3	0.9	19	18	18	310	220	117	59	137.6	7.1	61	433	13.1	41.1	36.3	282	14	0.76	144	4.5	103	9.5	5.6	106	15.5	11.6	0.9	0.9
56	M	166.4	75.7	27.34	30	97	113	65	81	60	7	7.5	4	1.1	0.8	32	47	79	179	214	146	54	130.8	4.9	55.3	448	15.1	44.3	24.2	261	10.1	0.96	141	4.2	105	9	7	108	10.5	11.7	0.9	0.7
52	M	163.9	57.8	21.52	18.4	74	137	78	97.67	63	2.7	7.7	4.7	1.6	0.4	16	12	28	244	167	91	56	92.8	5.3	71.4	500	15.1	44.5	22.6	233	13.4	0.83	144	3.6	105	9.3	5.2	77	8.7	7.9	0.5	0.8
56	F	151.7	53.5	23.25	28.5	81	96	58	70.67	67	5.4	7.2	4.3	1.5	0.4	28	39	69	223	197	94	49	129.2	5.2	57	447	13.3	41.5	24.4	236	12.3	0.54	144	4.2	106	9.2	4.1	56	12	12	0.5	0.8
69	M	167	64.6	23.16	20	83	147	82	103.7	56	10.3	7.8	4.5	1.4	0.9	16	16	25	196	279	126	75	178.8	5.1	71.1	533	15.9	48.1	21.4	301	16.1	0.89	141	4.7	102	9.7	6.1	111	10.6	11.7	2.5	2.3
67	F	154.1	52.5	22.11	32.8	83	114	63	80	56	18.3	8	4.4	1.2	0.8	20	11	35	170	215	101	65	129.8	5.2	71.8	386	12.4	38.6	23.8	322	13.8	0.66	141	4.2	101	9.7	5.3	169	11.5	11	0.8	0.5
56	M	164.9	67.9	24.97	27.6	83	129	81	97	51	21.5	7.7	4.7	1.6	0.7	18	18	20	407	234	88	50	166.4	5	68.6	505	15.2	46.2	17.9	239	15.4	0.83	144	4	104	9.7	5.9	81	7.6	7.7	0.5	0.7
57	F	150.8	39.7	17.46	20.3	64	116	64	81.33	66	20.5	7.8	4.6	1.4	0.6	19	11	12	167	192	29	77	109.2	4.8	61.1	429	13.3	40.4	17.3	241	8.6	0.54	143	3.4	104	8.9	3.5	82	10.9	9.1	0.7	0.8
63	M	162.4	51.6	19.56	18.7	72	135	67	89.67	69	6.6	7.8	4.8	1.6	0.5	32	33	69	203	139	90	56	65	5	78.5	452	14.8	44.3	19.2	301	14.5	1.09	140	4.4	102	9.6	6.5	124	16.4	13.4	1.6	2.3
40	F	166.7	59	21.23	28.9	77	119	56	77	58	17.9	7.2	4.3	1.5	0.4	16	10	14	127	235	56	89	134.8	4.6	41.7	417	11.6	36.4	21.7	216	6.9	0.54	142	3.5	106	8.9	3.5	64	17.8	17.2	0.6	0.5
67	M	167.9	67.7	24.02	23.5	83	123	80	94.33	60	7.7	7.7	4.5	1.4	0.7	30	37	50	228	191	157	43	116.6	5.3	75.5	496	16.6	49.4	29.5	360	17.4	0.93	142	4.5	105	10	7	78	18.6	16.2	0.8	0.7
61	F	161.3	44.5	17.1	17.1	63	109	64	79	62	36.2	6.6	4	1.5	0.6	19	13	20	202	234	93	58	157.4	5.1	52.4	399	12.5	38.9	21.2	273	15.7	0.66	144	4	106	9.1	3.9	87	12	12.3	0.7	0.8

66	F	156.9	66.5	27.01	34.1	90	155	81	105.7	75	11	7.4	4.2	1.3	1.2	20	14	15	251	191	70	48	129	5.3	68.6	459	13.7	41.9	23	437	15.9	0.64	143	3.9	105	9.5	6.1	86	12.3	10.7	1.2	1.2
57	M	159.3	55.5	21.87	26.7	81.5	115	62	79.67	67	8.8	7.1	4.4	1.6	1	33	52	48	219	219	135	55	137	6.3	116.2	467	15.3	45.4	25.4	269	13.6	0.76	140	4.3	105	9	4.6	71	14.3	15.2	0.6	0.8
63	F	143.2	47.7	23.26	30.4	73	122	61	81.33	86	12.2	7.6	4.4	1.4	0.6	29	31	24	255	204	48	63	131.4	5.5	60	436	13	39.4	29.2	296	15.1	0.65	141	4.1	105	9.3	4.4	47	10.3	9.1	0.8	0.9
60	F	147.3	45.1	20.79	22.7	60	106	50	68.67	54	23.6	7.2	4.3	1.5	0.8	27	17	20	205	249	42	87	153.6	5	50.7	474	14.8	44.5	25.9	226	18.8	0.61	142	4	106	9.6	4.2	58	13.1	11.9	0.7	0.7
68	F	154.9	48.7	20.3	24.6	70	110	56	74	55	14.8	7.2	4.3	1.5	1.6	18	16	38	140	257	106	77	158.8	5.3	56.8	406	12.9	39.9	23.1	254	12.9	0.55	143	3.9	103	9.2	4.1	75	14.9	19.4	0.7	0.8
55	F	155.5	68.3	28.25	36.9	85	119	74	89	71	25.1	7	4.5	1.8	0.5	19	33	27	180	231	120	63	144	5.3	60.2	479	13.2	40.6	32.8	257	10.1	0.59	143	4.2	107	9.5	5.7	97	10.6	12.7	0.6	0.6
52	F	159.3	51.1	20.14	24.3	68	120	70	86.67	53	17.8	7.9	4.8	1.5	1.6	24	15	19	154	270	71	93	162.8	5.4	26.6	408	12.7	38.5	19.1	277	12.1	0.68	142	3.9	103	9.7	4.6	108	14.4	12	0.5	0.7
57	M	163.4	69.4	25.99	28.2	86.5	148	89	108.7	69	4	8.1	5.1	1.7	0.9	28	37	21	282	240	151	49	160.8	5.3	42.9	518	15.2	46.4	22.2	221	19.8	0.83	143	4.5	104	9.6	5.7	76	12	9.8	0.6	0.8
65	F	142.8	51.4	25.21	36.5	75	154	79	104	71	7.9	8	4.3	1.2	0.6	37	66	46	244	229	102	59	149.6	5.5	45.3	477	14.2	43.1	19.1	301	18.6	0.6	144	4.5	107	9	5.2	94	15.2	14.6	0.5	0.7
58	M	160.5	71.6	27.79	31.8	86.5	130	79	96	78	32.7	7.8	4.1	1.1	0.8	26	27	60	255	172	126	47	99.8	5.4	66.6	472	15.2	45.9	16.1	273	11.7	0.91	142	4.2	105	9	7.2	133	14	13.6	0.5	0.7
57	F	158.1	51.4	20.56	24	71	100	48	65.33	62	9.9	7.4	4.5	1.6	0.8	21	16	27	236	217	87	90	109.6	5.2	46.3	417	12.5	39.2	29.3	265	16.7	0.66	144	4	103	9.4	4.7	64	7.9	8.5	0.6	0.5
66	M	162.6	49.7	18.8	16.4	73.5	122	59	80	52	7.5	7.3	4.5	1.6	1	24	17	28	211	221	99	63	138.2	5.1	53.9	456	14.1	43.7	21.6	265	8.8	0.78	145	4.6	105	9.5	6.5	76	11.9	14.6	1.1	1.1
57	M	166.1	74.3	26.93	30.1	91	118	81	93.33	59	11.7	7.6	4.5	1.5	0.5	58	79	107	191	161	195	33	89	5.5	62.9	496	15.6	47	27.9	317	12	0.74	144	4.2	106	9.7	6.4	71	12.8	13.6	0.6	0.8
59	M	167.2	64.5	23.07	21.3	80.5	128	66	86.67	65	9.2	7.6	4.6	1.5	1	23	32	27	273	207	139	51	128.2	5.2	73.2	540	16.1	48.1	20.2	334	9.8	0.79	141	4.3	103	9.6	5.3	87	9.8	10.3	0.7	0.8
54	M	166.5	84.6	30.52	29.1	93	127	67	87	71	36.3	7	4.4	1.7	0.8	32	55	105	146	173	83	55	101.4	6.8	66.9	490	15.8	46.9	15.2	265	28.7	0.76	142	4.1	104	9.2	5.9	91	17.3	17.1	0.8	0.8
58	M	169.2	85	29.69	31.6	97	120	66	84	70	11	6.8	4.3	1.7	0.7	31	50	72	182	187	156	66	89.8	5.7	59.6	547	15.7	48.2	18.8	291	14.2	0.93	143	4	104	8.8	7.3	58	13.6	14.4	0.6	0.7
69	M	161	57.2	22.07	22.6	83.5	107	56	73	66	8.2	7.2	4.1	1.3	0.4	22	19	26	197	204	143	62	113.4	5.2	66.7	462	13.8	42.4	24.8	301	11.4	0.66	141	4.3	102	9	5	73	18.2	17	0.8	0.9
69	F	148	50.1	22.87	28.2	81	123	61	81.67	80	8	7.4	4.6	1.6	0.5	20	23	51	317	187	96	58	109.8	5.6	35	433	13	40.3	19.5	219	12.2	0.58	143	4.4	104	9.2	6.2	70	17	17.1	0.7	0.8
52	F	153.9	63.1	26.64	21	70	98	53	68	71	4.9	6.6	3.9	1.4	1	14	10	13	132	192	88	65	109.4	5.3	65.8	458	13.4	41.3	25	286	10.1	0.62	139	3.9	105	8.7	4.8	54	9.4	13.4	0.8	0.8
69	M	159.5	49	19.26	15.9	64	127	81	96.33	72	8.9	7.4	4.5	1.6	1.1	22	15	13	319	235	65	65	157	5.4	83.7	483	14.4	44.4	17.4	311	17.6	0.75	143	4.1	103	9.4	4.3	76	10.9	9.7	1.1	0.9
51	F	154.2	57.3	24.1	34.6	88	117	71	86.33	66	4.1	7.2	4.4	1.6	0.6	18	14	15	201	153	63	57	83.4	4.9	47.6	432	12.1	38.3	29.6	241	9.6	0.46	141	3.9	104	9	4.5	76	17.6	17.2	0.7	0.7
60	M	172	76.3	25.79	25.8	87	137	80	99	57	8.7	7.3	4.5	1.6	0.7	22	17	41	157	175	175	66	74	5	52.4	532	16.9	50.3	22.9	291	16.6	0.8	143	4.4	105	10.3	6.1	60	8.8	8.6	0.8	0.9
54	F	151	49.1	21.53	28.6	69	100	70	80	54	19.6	7.9	4.5	1.3	0.6	34	23	53	209	263	59	98	153.2	5.3	52.4	459	12.4	39	35.2	398	17.9	0.66	142	4.5	103	9.8	5.2	119	11.4	12.6	0.6	0.7
57	M	173.5	83.4	27.71	24.6	91	115	68	83.67	54	4.3	7.2	4.6	1.8	1	22	24	56	297	222	127	70	126.6	4.9	53.5	476	14.3	43.8	19.7	198	11.9	0.77	144	4	108	9.4	5.4	46	7.8	7.6	0.7	0.7
52	F	162.5	43.7	16.55	14.6	60	120	60	80	78	22.5	7.4	4.6	1.6	1.3	24	21	21	189	195	40	88	99	5.1	37.4	437	13.4	41.2	11.3	219	12.3	0.6	144	4.3	106	9.5	3.1	72	14.3	14.3	0.5	0.7
65	M	158.9	44.8	17.74	13.4	62	92	53	66	60	18.9	6.9	4.3	1.7	0.6	26	15	19	266	227	76	68	143.8	5.2	99.5	477	14.3	43.5	15.1	328	19.5	0.68	143	4.3	104	9.4	4.2	94	15	11	0.7	0.7
68	M	159.6	57.3	22.5	24.3	86	127	82	97	80	2	7.5	4.5	1.5	0.9	26	25	42	465	210	135	71	112	5.1	39.9	496	15.2	47.3	20	375	17.1	0.81	142	4.4	102	9.8	3.2	91	13.1	13.1	0.7	1.6
67	F	146.1	39.2	18.36	21.8	60	117	63	81	66	4.1	7.5	4.5	1.5	0.8	24	12	10	226	220	68	91	115.4	5.7	37.8	418	13.1	39.9	23	334	18.5	0.76	144	5.2	104	9.7	5.2	127	14.6	15.4	0.5	0.9
69	M	162.1	51	19.41	14.3	73.5	107	57	73.67	59	21	6.9	4.3	1.7	0.3	15	10	12	214	219	106	63	134.8	5.5	66.5	426	11.6	36.1	37.1	368	13.4	0.75	142	4.1	103	9.3	4	92	8	8.6	0.6	0.8
57	M	163.9	63.1	23.49	22.6	87	132	80	97.33	62	3.5	6.9	4.5	1.9	0.6	16	19	131	219	222	79	61	145.2	5.2	51.4	450	15	44.6	20.3	286	13.5	0.77	144	4.2	106	9.1	5.3	98	14.2	13.1	0.7	1.1
51	M	170.4	81.6	28.1	35.2	89	144	75	98	72	4.8	8.1	4.5	1.3	1	36	47	20	206	165	76	42	107.8	5.4	66.4	536	16.4	49.5	26.3	269	12	0.81	140	4.3	102	9.8	7	109	8	9.9	0.7	0.8
63	M	170.5	64.3	22.12	18.4	78	141	71	94.33	61	5.2	7.5	4.4	1.4	1.1	30	23	24	151	212	107	71	119.6	5.2	62.2	482	16.4	49	18.2	286	15.8	0.8	141	4.4	103	9.1	5.5	101	22.5	19.4	0.8	0.8
59	F	156.8	51.1	20.78	23.9	67.5	115	70	85	79	16.4	8	4.7	1.4	1.2	21	25	23	232	236	90	56	162	6	66.7	525	15.6	46.7	21.7	291	16.5	0.7	141	4.3	101	9.7	4.5	116	17.2	16.9	0.8	0.8
69	M	147.7	61.8	28.33	28.2	87.5	108	69	82	75	11.7	7.3	4.2	1.4	0.8	22	26	26	186	194	87	50	126.6	5.7	51.1	464	14.5	44.3	19.2	286	14.4	0.92	143	4.7	105	9.8	7.3	77	9.2	11	0.9	0.8
64	M	169.5	53.8	18.73	14.3	72.5	112	64	80	49	15.8	7.3	4.6	1.7	0.6	38	21	15	203	191	63	75	103.4	5	41.4	482	15.5	46	16.5	328	20.2	0.96	144	4.2	104	9.3	4.9	123	14.9	11	0.9	0.8
66	F	148.2	43.2	19.67	26	72	152	83	106	67	18.1	7.4	4.4	1.5	0.5	26	17	16	234	208	156	72	104.8	5.4	54.9	405	13	40.1	17.1	340	15.2	0.55	142	3.8	101	9.1	4.7	91	11.5	12.7	0.8	1.1
64	M	167.3	75.1	26.83	27.2	94	137	85	102.3	66	5.6	7.8	4.8	1.6	0.5	31	39	40	192	192	184	47	108.2	6.5	70.5	490	14.3	44.6	30.9	269	19.6	1.19	143	4.5	105	10.3	5.9	72	14.6	15.4	0.9	1.2
61	F	154.5	64.9	27.19	27.9	84	100	55	70	66	11.5	7.5	4.2	1.3	0.9	31	44	24	273	220	173	57	128.4	5.3	47.3	482	12.8	41	27.9	347	12.4	0.6	144	4.2	104	9.3	5.4	58	15.6	12.6	0.8	0.8
50	M	167.1	65.4	23.42	25.8	77	152	93	112.7	61	8.3	6.9	4.7	2.1	0.6	16	13	40	150	213	191	51	123.8	5.9	81.8	479	14.9	44.7	29.5	247	16.8	1.02	147	4.6	108	9.5	3.4	41	13.7	17	0.7	0.7
50	M	160.5	68.5	26.59	26	88.5	127	70	89	66	3.2	7.1	4.5	1.7	0.5	31	28	42	164	213	230	46	121	9	54.4	483	15.7	45.7	18.3	322	21.4	0.63	141	4.5	101	9.5	3.1	46	10.7	11.9	0.8	0.8
56	M	165.4	66.7	24.38	24.8	81	131	70	90.33	49	5.4	8	4.7	1.4	2.1	35	54	91	248	214	192	57	118.6	5.1	57.4	556	17.5	51.7	19.4	261	9.6	1.02	143	3.7	103	9.5	4.9	49	9.7	11.6	0.8	0.7
57	F	152.5	47.7	20.51	25.6	72	108	52	70.67	62	14.4	7.1	4.4	1.6	0.7	18	11	11	189	236	58	82	142.4	5	50	421	12.4	38.5	21.4	241	12.4	0.72	144	4	105	9.5	5.3	79	11.5	13.3	0.7	0.7
58	M	169.6	70.8	24.61	25.7	85	149	85	106.3	46	11	7.9	4.6	1.4	1.2	25	25	21	179	221	136	70	123.8	5	59.6	466	14.8	44.8	30.5	269	16.3	0.73	141	4.7	102	9.6	5.1	52	12.5	12.4	0.5	0.9
61	F	150.5	36.8	16.25	15.5	59	123	70	87.67	74	30.2	7.1	4.1	1.4	0.5	21	22	33	167	182	86	83	81.8	5.1	60.1	447	13.6	42.8	24.7	334	13.8	0.72	144	3.6	106	9.1	5.2	64	17.6	15.5	0.7	0.8
55	M	176.5	81.4	26.13	28.3	87	158	83	108	65	8.1	8	4.4	1.2	0.4	22	31	84	299	181	570	36	31	7.3	87.8	519	16.7	50.1	21.5	301	14.2	0.76	141	4.2	103	9.1	5.4	59	12.8	14	0.8	0.7
64	M	167.5	57.5	20.49	19.3	80	117	67	83.67	51	8.7	7.7	4.5	1.4	1	22	16	18	171	197	55	64	122	5.4	49.2	522	15.6	46.7	22.4	334	11.5	0.83	141	4	101	9.2	3.8	104	7.8	8.3	0.9	0.7
65	F	147.2	52.2	24.09	31.3	81	128	65	86	70	54.2	7	4.1	1.4	0.5	18	17	13	248	226	150	61	135	5.8	74.2	409	12.4	38.3	29.2	353	11.6	0.66	144	3.9	105	9.2	4.2	76	10.5	10.8	0.8	1
66	M	163.4	56	20.97	16.5	81	154	93	113.3	49	20.7	7.7	4.5	1.4	1	44	26	25	255	178	86	45	115.8	5.3	61.3	469	14.5	44.5	20.9	353	15.3	0.73	143	4.2	102	9.3	4.6	135	8.1	7.9	0.9	1.3
67	M	158.3	51.9	20.71	17.2	72.5	138	70	92.67	62	44.4	7.3	4.3	1.4	1.6	25	22	38	259	177	87	63	96.6	5.1	43.9	437	13.5	39.9	18.5	301	10	0.67	143	3.9	104	9.2	4.7	94	8	11.5	0.7	0.7
67	F	145.8	45.1	21.22	27.9	71	133	70	91	56	104	7.6	4.1	1.2	1	16	13	21	245	210	118	62	124.4	4.9	63.3	443	14.1	42.2	20	261	9.1	0.54	142	3.8	102	9.1	4.2	93	10.6	11.2	0.5	0.8
68	M	166.1	63	22.84	21.9	80.5	131	76	94.33	63	2.1	7.3	4.7	1.8	0.8	17	14	26	107	202	90	64	120	6.9	110.1	493	15.4	46.1	22.4	277	21.3	1.08	143	4.3	102	11	6.6	44	14.5	15.9	0.9	0.9
65	F	146.3	48.7	22.75	36.1	83	126	61	82.67	91	7.4	8.5	4.9	1.4	0.6	36	55	28	172	236	130	48	162	6	70.4	473	14.5	44.5	27.4	317	12.9	0.44	142	4.2	102	10	5.9	92	14	16.8	0.8	1.2
64	F	149	47.7	21.49	34.1	83	127	70	89	67	4.4	7.5	4.4	1.4	0.7	22	17	22	215	235	123	63	147.4	4.9	61.3	427	13.7	41.5	24.4	273	14.7	0.43	145	3.6	104	9.3	3.7	61	12.9	13.2	0.7	0.7
46	F	156.4	93.6	38.27	51.7	109	125	76	92.33	69	5.7	7.2	4.1	1.3	0.6	33	39	31	190	220	261	53	114.8	8.5	96.4	440	13.1	39.4	20.8	286	10.8	0.49	140	4.1	99	9.2	6.1	54	16.5	18.2	0.6	0.7
60	M	166.9	83.2	29.87	30.5	93	123	67	85.67	52	4.7	7.8	4.4	1.3	0.6	23	25	38	196	249	148	60	159.4	5.4	65.8	482	14.9	45.2	25.7	286	12.9	0.86	143	4.3	104	9.5	7.3	103	10.5	12.3	0.7	0.7
60	M	167.9	60.3	21.39	20	76	133	70	91	50	16.6	6.9	4.6	2	0.6	17	13	24	173	188	86	71	99.8	5	48.1	418	13.6	40.7	21.4	273	19.4	0.84	146	4.3	106	9.5	7.2	67	14.5	14	0.7	0.7
56	M	170.9	74.3	25.44	24.3	86.5	148	73	98	65	14.8	6.7	4.4	1.9	1.4	25	23	177	139	180	241	58	73.8	5.4	70.9	392	13.2	39.4	24.6	219	16.3	0.94	146	4.2	108	9.2	7.9	43	12	11.2	0.7	0.7
63	M	163	67.2	25.29	21.6	80	161	88	112.3	64	16.9	7.4	4.2	1.3	0.8	20	22	63	228	233	119	67	142.2	5.5	60.6	467	15.2	44.9	21.7	265	9.5	0.87	142	3.9	103	9.5	8	101	10	9.7	0.8	0.8
68	F	150.6	57.3	25.26	32.8	87	127	79	95	64	102	7.9	4.7	1.5	0.7	21	20	17	316	210	102	61	128.6	5.1	102.2	429	12.9	40.2	24.9	328	14	0.63	144	4.8	105	9.5	4.6	70	13.7	12.8	0.9	0.9

50	M	165.7	55.5	20.21	17.7	71.5	108	68	81.33	49	24.1	7.3	4.5	1.6	0.8	16	12	45	199	206	89	82	106.2	5.4	64.3	465	15.6	44.3	20	269	11.2	0.75	145	4	104	9.4	7.2	49	11	14.2	0.7	0.5
65	F	146.1	55.6	26.05	42.9	86.5	138	65	89.33	54	28.2	8.1	4.7	1.4	0.8	33	43	24	307	160	49	49	101.2	5.3	68.1	447	13.4	41.2	27.6	328	11.8	0.56	144	4	102	9.8	4.9	86	13.3	14.4	0.8	0.9
62	M	162.5	59.7	22.61	21	78	146	76	99.33	85	6.2	8.1	4.3	1.1	0.6	40	52	129	230	209	245	52	108	10	102	462	15.2	43.7	27	296	20.6	0.96	143	3.5	101	9.4	5.4	46	16.7	17.9	0.5	0.8
65	F	141.9	46.3	22.99	33.6	71	154	77	102.7	63	15	8.2	4.4	1.2	0.6	23	23	35	248	225	113	76	126.4	5.7	62.5	488	13.4	41.3	21	340	14.7	0.68	142	3.7	102	9.2	5.7	87	12.9	10.6	0.8	0.7
64	F	156.2	52.7	21.6	27.8	72	115	52	73	76	16.2	7.4	4.5	1.6	0.7	22	13	15	245	248	105	60	167	5.3	58.7	415	12.4	39.1	18.3	411	11	0.63	143	3.9	102	9.3	4.4	69	14.8	13	0.5	0.8
59	F	153.2	54	23.01	26.9	74	118	59	78.67	56	35.2	7.1	4	1.3	0.6	31	23	27	187	173	182	42	94.6	5.5	65.9	467	13	40.7	28.1	216	8.8	0.7	144	4	108	9.1	4.5	87	10.6	12.8	0.7	0.8
61	M	161.3	57.5	22.1	23.2	80.5	130	60	83.33	68	154	7.5	4.5	1.5	0.9	41	34	83	168	211	129	42	143.2	5.5	59	464	15	45.5	23.3	360	9.9	0.64	142	4.1	103	9.4	5	73	13.1	10.6	0.5	0.5
65	M	162	57.6	21.95	18.4	73	142	64	90	66	82.9	7.4	3.9	1.1	0.3	39	19	47	286	132	87	82	32.6	4.7	62.6	375	11.9	35.2	29	429	14.9	0.82	137	4.6	98	8.4	6.1	126	12.8	16.3	0.7	0.8
65	F	149.7	44.3	19.77	27.6	76	133	74	93.67	73	15.1	7.6	4.4	1.4	0.6	22	15	19	233	258	107	89	147.6	5.2	43.5	445	14.1	40.8	18.5	257	11.3	0.64	143	4.2	104	9.3	5.1	79	9.3	9	0.8	0.9
66	M	162.5	49	18.56	15	68	128	77	94	61	19.1	7.4	4.4	1.5	1.1	28	21	109	254	219	55	99	109	5.2	64.9	488	16.2	46.6	17.8	282	16.6	0.81	145	4.6	106	9.2	6	68	15.6	12.7	1.214	0.7
64	M	168.1	66.5	23.53	25.1	83	109	76	87	69	55.8	6.9	4.5	1.9	1.3	21	30	29	272	213	145	56	128	5.2	73.8	510	15.8	47.6	17.4	254	11.7	0.64	143	4.2	104	9.2	5.4	77	15.3	15.5	0.7	1.3
69	M	164	59.9	22.27	18.9	77.5	115	65	81.67	49	39.5	6.4	3.9	1.6	1.4	27	20	67	287	171	104	76	74.2	4.9	47	416	14.8	42.1	24.2	244	14.7	0.86	143	4.1	104	8.9	5.4	117	10.4	9.4	0.8	0.8
67	F	141.7	39.3	19.57	27	66	130	58	82	66	97.6	7.6	4.2	1.2	0.9	30	28	72	228	206	63	66	127.4	5.7	60.5	448	13.6	41.2	27.2	433	10.4	0.61	142	4.5	100	9.5	4.7	72	11.3	13.3	0.8	1.8
59	M	166.8	55.5	19.95	18.2	78	146	85	105.3	66	40.6	7	4.2	1.5	0.5	18	14	30	175	185	65	52	120	5.3	64	469	15.4	47.1	22.5	368	10.4	0.82	144	4.1	104	9.1	6.2	56	12.9	12.3	0.5	0.7
59	M	154.1	55.7	23.46	21	80	115	67	83	64	12	7.4	4.2	1.3	0.3	23	18	14	278	150	95	41	90	6	48.8	466	13.4	40.4	36.8	442	12.6	0.69	144	4.3	102	9.4	5.3	48	12	10.3	0.7	1.1
59	F	151.3	55.7	24.33	27.7	78	120	63	82	61	26.8	7	4.4	1.7	1.1	18	14	25	294	282	82	118	147.6	5.1	75.2	443	13.7	42.2	22.4	311	21.1	0.64	144	4.3	104	9.4	4.5	201	12.9	13.3	0.7	0.7
68	F	149.6	47.6	21.27	27.7	79.5	122	68	86	74	28.8	7.1	4.4	1.6	0.8	25	23	25	246	192	59	68	112.2	5.1	33.9	404	13.8	40.9	13.4	411	18.5	0.63	144	4.3	105	9.4	4.5	96	12.5	12	0.7	0.8
68	F	142.6	33.5	16.47	15.1	69	138	62	87.33	65	42.5	7.7	4.6	1.5	0.6	28	17	28	266	223	84	78	128.2	5.1	33.3	421	13.5	41	16.1	244	10.1	0.57	146	3.7	105	9.1	3.2	74	10.8	11.2	0.7	0.8
66	F	151.3	65.5	28.61	40	83	131	67	88.33	70	13.8	7.4	4.4	1.5	0.9	23	16	30	320	228	209	52	134.2	5.6	56.1	402	12.3	37.3	32.6	340	13.1	0.53	145	3.8	104	8.9	4.1	86	11.3	9.8	0.7	0.7
67	F	147.7	43.5	19.94	25.5	79.5	150	75	100	70	33.4	7.8	4.3	1.2	0.6	29	19	28	301	239	93	68	152.4	5.7	72.7	459	13.9	43.1	39.4	375	7.4	0.6	142	4.2	102	9.6	3.6	137	15.8	15.5	0.5	2
65	F	147	46.5	21.52	22.5	72	128	68	88	63	15.6	7.9	4.8	1.5	0.8	30	23	15	358	219	91	72	128.8	5.4	52.6	434	14.5	43.9	20.2	328	15.4	0.6	144	4.7	103	9.9	4.5	76	13.5	12.1	0.8	1.5
67	M	153.7	45.7	19.34	11.9	65	122	67	85.33	51	59.8	6.7	4.2	1.7	0.5	22	14	20	217	195	92	73	103.6	5	38.7	421	12.8	39.9	24.7	223	13	0.89	142	4.5	105	8.8	4.8	124	12.3	12.6	1.2	0.7
64	F	145	54.7	26.02	35.2	91	144	78	100	77	3.7	7.7	4.6	1.5	0.5	21	25	121	348	220	81	89	114.8	5.3	69.1	436	14.3	43.4	20.8	291	15.6	0.79	142	4.4	103	9.7	4.2	106	14.3	15.9	0.7	0.7
66	F	155.2	52.9	21.96	28.2	85	101	54	69.67	72	5.7	7.2	4.4	1.6	0.9	26	24	20	262	221	147	67	124.6	5.2	54.7	456	13.5	40.8	29.2	250	20.9	0.65	143	4.5	104	9.6	4.9	131	14.1	13.2	0.8	0.8
68	M	164.7	62.6	23.08	24.1	81.5	138	86	103.3	87	9	7.7	4.4	1.3	0.8	29	32	63	196	218	124	82	111.2	5.4	80	510	15.6	47.2	26	291	14.2	0.79	143	5.1	102	9.5	7.2	95	16.3	13.7	0.7	0.8
64	F	152.9	58	24.81	30.4	84	135	71	92.33	66	16.9	6.9	4.5	1.9	0.6	21	29	33	216	204	138	52	124.4	5.4	67.7	428	13.1	39.7	22.3	296	18.6	0.6	143	4.1	105	9.4	5.5	69	11.3	12.9	0.7	0.8
67	M	166.4	63	22.75	23.2	81	156	72	100	58	6.6	7.2	4.3	1.5	0.6	26	17	36	254	196	100	65	111	5.7	64.3	514	15.2	47.4	18.7	269	19.8	0.89	141	4.4	104	9.1	6	101	13.3	15.3	1.2	3.3
68	M	167.2	54.2	19.39	19.3	73	134	82	99.33	58	51.4	7.2	3.9	1.2	0.6	23	11	63	176	173	106	51	100.8	5.3	70.4	398	12.8	39.4	26.5	334	12.7	0.82	143	4.6	105	9.1	7.4	73	12.2	10.8	0.5	0.7
66	F	152.9	55.9	23.91	33.2	91.5	149	91	110.3	67	14.4	7.2	4.3	1.5	0.8	31	28	19	224	195	107	71	102.6	5	61.9	430	14.1	42.3	28.6	317	10.4	0.66	144	4.5	105	9.4	3.7	73	14.6	16.8	0.7	0.9
55	M	174.5	81.6	26.8	26	84	130	74	92.67	58	63.4	7.6	4.3	1.3	1.6	33	23	32	136	158	33	54	97.4	5.1	63.3	485	15.5	46.1	18.6	250	16.1	0.97	142	4	101	9.4	6.6	98	13.3	13.7	0.7	0.7
54	F	147.8	38.5	17.62	19.9	57	103	52	69	76	9.5	6.9	4.1	1.5	0.4	24	17	32	140	227	39	105	114.2	5	45.2	370	9.6	32.4	49.9	375	10.2	0.69	140	3.3	98	9.4	5.9	78	7.9	7.9	0.5	0.5
57	M	159.1	50.5	19.95	18.3	76	146	90	108.7	64	28.9	7.3	4.4	1.5	0.5	26	20	36	243	194	78	70	108.4	4.8	70	428	14.8	45.4	24.5	340	15.9	0.98	142	4.2	103	9.5	7.9	108	14.3	14.1	0.5	0.7
69	M	170.9	72	24.65	21.1	87	126	78	94	70	22.4	7.1	4.2	1.4	0.7	22	17	21	203	195	84	51	127.2	5	44.3	473	14.7	45.2	20	257	15.5	0.68	141	4.1	102	8.6	2.8	56	11.9	12.9	0.7	0.7
59	F	146.5	40.7	18.96	25.7	61	174	98	123.3	60	87.4	7.4	4.5	1.6	0.8	30	17	70	212	233	85	84	132	5.1	33.7	424	13.1	40.7	20.5	241	13.8	0.52	143	3.8	104	9.3	3.8	82	13	11.5	0.7	0.7
65	F	147.2	39.7	18.32	20.7	62	158	77	104	82	19.6	7.3	4.4	1.5	0.7	26	20	15	306	219	114	76	120.2	4.9	65.2	568	15.4	48.7	23.6	247	12.2	0.48	144	4.4	104	9.1	3.3	69	12.9	12.3	0.8	0.9
63	M	163	60.8	22.88	22	79	120	61	80.67	83	5.5	7.4	4.5	1.6	0.8	39	79	120	286	254	171	66	153.8	5.3	66.5	485	16.1	48.4	19.5	261	22.6	0.74	142	4.8	103	9.6	3.8	114	11.7	10.4	0.8	0.7
59	F	151.6	56.1	24.41	38.4	76	132	76	94.67	82	2.9	7.7	4.3	1.3	1.5	16	15	24	217	220	94	64	137.2	5.6	69.8	485	14.6	45.8	27.6	317	17.1	0.47	142	4	102	9.5	5.7	67	13.6	14.6	0.7	0.7
56	M	163.7	63.6	23.73	29.3	84	124	72	89.33	72	13	7.3	4.5	1.6	1.5	19	22	193	279	186	100	83	83	5.5	60.8	562	9.2	35.2	41.7	202	17	0.73	139	4.5	101	9.1	6.6	70	18.2	14.4	0.5	0.7
54	M	173.3	78.8	26.24	27.5	90	151	85	107	75	11.1	7.5	4.6	1.6	1	30	19	80	156	209	288	47	104.4	5.3	133.4	441	15.3	44.6	17.7	375	12.6	0.83	144	3.8	104	9.7	8	64	12.9	11.9	0.7	0.7
57	M	170	63.7	22.04	18.8	80	126	80	95.33	52	4.2	7.6	4.7	1.6	1.2	17	15	45	243	202	80	101	85	5.3	49.1	479	15.1	45.9	21.2	261	14.1	0.98	145	4.3	102	9.9	7.2	89	9.2	9.2	0.4	0.5
56	M	163.3	62.5	23.44	26.5	82	143	77	99	74	5.6	8.1	4.7	1.4	0.3	19	13	30	321	188	150	46	112	5.1	90.6	489	15.9	45.9	21.1	398	14.4	0.79	144	4.1	102	9.7	7.4	64	17.4	18.9	0.7	0.7
66	F	150.9	48.6	21.34	27.5	80	90	54	66	69	17.7	7.2	4.3	1.5	0.4	47	73	91	333	205	99	52	133.2	6	93.1	452	13.3	41.3	35.3	433	14.5	0.59	143	4.4	103	9.4	4.9	80	12.6	12.1	0.7	0.8
61	F	144.5	51.7	24.76	27.6	73	108	61	76.67	68	34.6	7.9	4.3	1.2	0.7	48	42	87	186	173	28	79	88.4	5.2	57.4	428	13.6	41.8	21.2	269	12.2	0.54	143	3.5	103	9	4	61	19.1	19.1	0.7	0.5
62	F	152.3	53.7	23.15	31.6	75	121	66	84.33	68	8.1	7.7	4.8	1.7	0.6	19	18	30	234	146	51	69	66.8	5.8	96.6	433	12.6	38.9	28.2	411	16.7	0.64	143	4	103	9.7	5.8	97	13.4	15.6	0.5	0.5
66	F	146.4	53	24.73	38.4	92	117	71	86.33	69	11.7	7.4	4.3	1.4	1	19	13	18	150	216	134	53	136.2	5.3	56	406	12.8	39.2	16.5	282	12.8	0.6	146	3.8	104	9.3	4.4	88	13.8	12.4	0.7	0.5
71	M	157.8	47.1	18.92	10.2	65	85	50	61.67	67	37.8	7.2	4.5	1.7	0.9	43	20	24	279	289	35	177	105	7.3	33.4	357	12.7	36.6	23.2	273	16.5	0.58	130	4.6	89	9.1	3.2	89	8.6	10.9	0.8	0.8
69	F	150.9	56.3	24.72	35	87	129	76	93.67	86	14.3	7.5	4.6	1.6	1.8	19	22	31	264	230	221	56	129.8	5.7	43.3	470	14.9	42.9	21.8	273	10.9	0.54	143	3.8	105	9.6	3.9	90	24.2	20.1	0.7	0.7
57	M	159.7	66	25.88	37.1	86	125	72	89.67	81	3.7	7.4	4.5	1.6	0.5	42	60	174	249	222	139	78	116.2	5.1	71.7	510	17.3	50.8	24.6	191	13.1	0.73	144	4.1	100	9.5	6.5	49	16.2	14.6	0.5	0.7
52	M	167.8	70.9	25.18	25	87	130	87	101.3	76	16.5	7	4.4	1.7	1.1	28	20	65	308	236	241	51	136.8	5	52.4	476	15.3	45.2	26.4	221	9.7	0.68	142	4.5	105	9.2	7.2	66	14.6	12.2	0.7	0.7
65	F	152.7	45.6	19.56	27.1	73	117	60	79	76	21.2	7.4	4.6	1.6	1	16	11	14	211	245	66	95	136.8	5.1	46.7	441	13.7	41.4	22.4	291	11.7	0.63	140	4.2	98	9.9	3.5	104	11.5	9.4	0.8	0.7
64	F	154.9	64.7	26.97	34.1	86.5	164	77	106	80	6.3	7.9	4.4	1.3	1.4	18	15	27	261	207	104	70	116.2	5	46.8	432	14.5	42.8	32.3	322	15.7	0.46	142	4.1	104	9.6	4.8	78	11.5	10.9	0.7	0.8
67	M	177.3	72.5	23.06	24.1	85	123	77	92.33	65	174	7	4.5	1.8	1	18	17	16	229	177	95	43	115	4.8	41.1	424	14.1	41.6	23.6	254	17.7	0.81	144	4	104	9	5	79	12.7	12.9	0.9	1.2
68	M	165.2	47.5	17.4	10.8	69	134	71	92	57	13.1	6.8	4.3	1.7	1	25	21	14	229	158	57	79	67.6	7	46	431	14.9	44.3	18	241	12.3	0.64	143	4	102	9	3.4	139	14.8	12.4	0.7	0.7
63	M	156.3	54.8	22.43	23.7	78	176	92	120	72	9.8	7.2	4.3	1.5	1.3	19	21	22	221	176	126	57	93.8	7.9	51.5	496	16.1	45.3	16	296	18.3	0.53	139	4.4	101	9.2	5.7	116	15.3	15.4	0.715	0.8
64	F	144.2	62.1	29.86	36.5	81	144	75	98	57	44.5	7.1	4.3	1.5	0.5	23	18	15	169	211	107	55	134.6	5.4	77.9	433	13	40.7	20.3	334	14	0.85	143	4.9	103	9.2	6.6	65	12	12.3	0.5	0.8
67	F	153.3	49.1	20.89	30.1	88.5	144	80	101.3	77	5.7	7.3	4.4	1.5	0.5	19	21	52	182	233	107	56	155.6	6.1	63.3	480	14.4	44.1	19	265	17.2	0.62	142	3.7	102	9.4	4.7	73	15.2	16.5	0.8	0.7
59	M	159.7	60	23.53	25.9	78.5	120	72	88	65	29.4	7.5	4.5	1.5	0.6	26	32	25	247	181	120	55	102	5.3	61.8	501	14.9	46.2	24.6	334	16	0.67	142	4.3	103	9.1	5.7	55	20.5	18.9	0.7	0.9
55	F	150.7	60.9	26.82	36.8	92	124	80	94.67	71	6.7	7.4	4.4	1.5	0.5	25	28	45	245	183	100	52	111	5.6	68.5	444	13.2	40.1	21.7	334	12.8	0.7	143	4.5	107	9.3	5	50	16.3	17	0.7	0.9
57	F	159.4	46.4	18.26	22.6	74	110	52	71.33	53	14.1	7.6	4.6	1.5	0.9	27	17	13	315	218	61	88	117.8	4.9	45.7	451	13.9	42.7	22.1	311	14	0.6	143	3.7	102	9.4	4.3	117	12.8	12.7	0.7	0.5

63	F	153.4	45.2	19.21	22.9	61	101	61	74.33	65	12	7.4	4.5	1.6	0.6	18	10	16	177	199	44	46	144.2	5.4	31.4	327	10.6	32.8	22.9	494	19.2	0.73	141	4.1	103	9.7	2.1	119	11.7	8.9	0.5	0.8
52	M	170.9	75.3	25.78	23.3	81.5	118	56	76.67	53	7	7.5	4.6	1.6	1.2	31	53	185	239	235	129	55	154.2	6	68.8	492	15.4	45.2	21.4	228	15.7	0.96	142	4.3	103	9.6	5.9	85	17	14.6	0.7	0.7
65	M	165.8	58.8	21.39	15.4	73	139	73	95	48	12.3	6.9	4.3	1.7	0.8	22	21	31	143	146	45	53	84	6	49.3	467	14.8	45.6	20	257	20.8	0.79	142	4	103	9.1	4.1	89	12.5	15.3	0.7	0.7
62	F	149.3	45.8	20.55	27.5	73	113	70	84.33	81	8	7.6	4.4	1.4	1	22	15	20	124	239	70	58	167	5.6	38.3	424	13.3	40.3	22.5	317	15	0.63	146	4.7	103	9.3	4.7	107	14.1	11.7	0.7	0.5
66	M	168.1	72.5	25.66	23.2	83.5	123	85	97.67	55	6.8	7.6	4.4	1.4	1.1	29	36	32	261	171	95	55	97	4.8	54.6	502	16.1	46.2	15.1	277	15.3	0.75	145	4.6	105	9.4	5.8	69	10	9.8	1	0.9
61	M	170.1	57.6	19.91	14.3	69	137	85	102.3	73	11.8	7.3	4.7	1.8	1	20	18	30	202	218	121	70	123.8	5.4	54.3	504	15	46.5	22.9	236	15.4	0.88	147	4.4	104	9.4	5.6	78	13.5	10.2	0.5	0.5
71	M	156.9	70.9	28.8	29.4	93	148	83	104.7	70	8.5	7.5	4.3	1.3	0.6	20	21	23	199	209	61	56	140.8	6.6	77.8	443	13.2	40	21.3	340	21.6	0.87	145	4.3	104	9	6.1	90	11.4	12.4	0.7	0.9
55	M	177.5	80.4	25.52	28.3	90	129	75	93	74	15	8	4.6	1.4	0.4	38	31	50	205	251	779	64	31.2	4.9	42	444	15.3	43.8	15.8	221	16.8	0.89	144	4.5	103	9	8.9	74	13.4	13.7	0.7	0.5
51	M	170.2	77.5	26.75	30.7	88	158	86	110	72	3.2	7.7	4.3	1.3	0.6	34	30	213	255	203	259	49	102.2	6.7	79.2	494	15.6	45.5	29.5	386	13.5	0.68	142	4.2	101	9	5.9	80	12.5	15.7	0.7	0.7
56	M	171.4	81.2	27.64	30	89	133	86	101.7	76	3.6	7.8	4.6	1.4	0.6	49	95	367	220	209	284	41	111.2	5.5	89.5	470	15.1	45.1	20.5	317	18.7	0.84	145	4.6	102	9.4	6.6	58	8.9	8.7	0.7	0.8
88	M	164.4	45.9	16.98	11.2	61	113	59	77	67	106	7.3	4.5	1.6	0.8	43	26	35	231	176	84	54	105.2	5.2	37.2	395	12.4	38.1	13.3	286	19.2	0.96	143	4.8	99	8.9	5.4	145	8.3	8.6	0.8	0.9
50	M	163.7	70.2	26.2	22.9	82	136	82	100	40	2.3	7.3	4.5	1.6	0.7	20	19	25	203	180	214	47	90.2	5.8	91.8	502	15.5	45.9	18.6	277	17.1	0.77	145	3.8	103	9.3	5	56	9	8	0.5	0.8
65	F	149.9	51.2	22.79	31.2	85	135	70	91.67	62	16.6	7.3	4.4	1.5	1.1	16	14	11	311	209	95	61	129	5.4	56.3	486	14.4	44	17.8	311	20	0.63	144	4.3	101	9.4	6.7	81	10	9.7	0.7	0.8
57	F	154.5	53.3	22.33	34.6	89	106	54	71.33	73	14.2	7.5	4.5	1.5	1	20	15	13	353	228	96	69	139.8	5.3	60.8	497	13.8	41.9	20.1	360	19.3	0.89	145	4.3	103	9.5	5.5	81	15.4	16.3	0.7	0.7
69	F	146.3	40.1	18.74	22.8	66.5	115	58	77	55	22.6	7	4.3	1.6	0.6	27	21	16	180	237	88	87	132.4	5.2	43.7	416	12.5	38.8	24.6	261	15.8	0.47	145	3.7	105	8.7	3.2	73	16.5	16.1	0.9	0.8
67	F	151.5	61.5	26.79	40.6	83.5	134	75	94.67	76	29	7.2	4.4	1.6	0.5	29	28	108	277	200	213	50	107.4	5.4	79.7	416	13	37.8	25	347	18	0.67	144	4	103	9.2	4.8	62	16.3	16.1	0.5	0.7
51	M	161.8	53.4	20.4	22.4	71.5	118	81	93.33	70	3.5	7.4	4.6	1.6	0.8	25	21	24	135	197	94	77	101.2	5.1	49.7	490	15.3	45.2	16.2	286	19	0.93	145	4	102	9.6	3.7	74	10.1	8.3	0.7	0.5
69	F	150.5	50.9	22.47	30	82	120	77	91.33	69	5.4	7.8	4.5	1.4	0.6	22	15	14	158	218	76	57	145.8	5.2	45.9	424	12.6	38.6	21.5	317	13.9	0.61	144	3.9	103	9.3	5.5	100	11	11	0.8	1
48	M	165	73.3	26.92	30.7	91.5	140	86	104	68	7.5	7.2	4.5	1.7	0.9	15	18	23	358	209	118	54	131.4	4.9	58.4	509	15.9	46.7	29.3	296	11.4	0.76	142	4.5	101	9.5	5.5	70	12.5	14.1	0.7	0.5
47	F	161.3	64.5	24.79	35.5	85	134	80	98	71	3.2	7.2	4.1	1.3	0.5	21	34	80	153	227	109	57	148.2	5.1	75.2	471	13.9	43.7	35.1	311	8.2	0.62	143	4.2	102	9.1	4.8	73	12	11.9	1.1	0.8
69	M	163.5	64.2	24.02	21.1	74.5	151	80	103.7	63	4.1	7.1	4.3	1.5	0.5	18	15	26	173	152	62	52	87.6	5.3	73.5	481	14.3	43.3	22.7	347	22.9	0.79	144	4.2	103	9	5.2	90	11.7	10.7	0.9	0.8
62	F	157.6	44.5	17.92	21.7	70	139	75	96.33	81	44.3	7.5	4.6	1.6	0.8	23	13	12	285	212	54	67	134.2	5	41.1	461	13.8	43.8	19.2	265	12.8	0.74	144	4	100	9.3	4.4	108	13.6	15.5	1.1	1.1
83	M	152.7	52.2	22.39	19.1	79	133	81	98.33	67	122	7.5	4.3	1.3	0.7	21	13	34	254	151	122	48	78.6	5.7	82.6	503	15.2	46.4	21.4	368	14	0.97	145	4.9	103	9.2	7	80	14.3	11.7	1.1	1.6
79	F	134.1	42.8	23.8	31.7	84	131	75	93.67	95	17.4	8.1	4.7	1.4	0.6	40	16	41	273	204	128	71	107.4	4.6	74.9	463	14.6	45.7	24.4	360	12.3	0.4	144	4.1	102	8.8	3.8	72	13.6	15.1	0.8	0.7
57	M	161.1	51.6	19.88	17.5	69	120	77	91.33	52	2.1	7.2	4.4	1.6	1.1	14	13	31	189	196	134	52	117.2	5	41.7	455	14.5	44	23.1	334	14.8	0.89	143	4	101	9.1	5.2	72	11.5	11.9	0.5	0.7
53	F	159.3	51.9	20.45	25.9	69	121	67	85	75	2.5	7.7	4.5	1.4	1.2	25	19	12	244	206	40	80	118	4.9	75.6	453	13.7	42.2	22.4	368	26.8	0.71	144	4.1	103	9.6	3.6	97	10.5	9.3	0.8	0.8
56	M	168.5	68.7	24.2	22.9	84	119	68	85	65	9	7.6	4.4	1.4	0.8	19	23	37	290	217	97	71	126.6	5.2	59.1	502	16.3	46.7	22.8	231	11.6	0.84	143	4.2	102	9.3	3.6	79	13.8	9.6	0.7	0.7
59	M	168.3	51.1	18.04	18.9	71	126	80	95.33	85	8.1	7.8	4.8	1.6	1	35	39	264	354	268	194	81	148.2	4.9	49.2	464	14.7	44.4	20.1	296	11.8	0.71	145	3.9	101	9.3	6.4	110	15.7	14.9	0.5	0.5
53	M	171.2	67.9	23.17	23.7	77.5	111	72	85	62	13.2	6.9	4.3	1.7	1	22	18	159	221	259	226	73	140.8	5.1	59.8	509	16.6	49	18.2	317	14.8	0.86	145	4.3	104	8.9	5.9	55	14.1	14.7	0.8	0.8
67	F	148.5	38.6	17.5	21.7	71	138	76	96.67	66	26.3	8	4.5	1.3	0.4	21	18	17	214	244	81	82	145.8	7.7	69.9	405	11.8	35.8	30.3	317	16.7	0.69	140	4.6	103	9.5	3.8	72	12.6	15.8	0.9	1.2
58	M	159.1	72.1	28.48	24.3	86	161	88	112.3	58	3.8	7.4	4.6	1.6	0.5	21	31	35	256	202	288	37	107.4	8.2	58.2	516	15.7	46.7	21.3	277	15	0.85	143	3.9	101	9.4	4.4	76	15.7	15.9	0.9	0.8
61	F	154.9	62.2	25.92	33.7	89	107	61	76.33	67	5.5	7.7	4.6	1.5	0.8	24	32	73	335	218	59	67	139.2	5.8	65.9	453	13.8	42.5	20.8	301	17	0.53	144	4	103	9.2	4.3	60	10.8	11	0.5	0.5
65	F	151.7	53.8	23.38	33.5	82.5	127	75	92.33	78	16.8	7.6	4.7	1.6	0.5	22	16	28	220	178	72	87	76.6	5.3	49.2	454	13.7	41.1	25.3	375	12.5	0.52	145	4.3	106	9.4	4.1	87	8.3	15.5	0.5	0.7
64	F	150.1	47.7	21.17	26.2	79	129	63	85	72	4.7	7.2	4.5	1.7	0.7	20	17	23	186	206	150	55	121	5.9	59.7	450	13.3	40.3	22.7	219	15.4	0.65	143	4.1	100	9.5	5.1	67	15.3	17.5	0.8	1.7
62	F	146	51.6	24.21	33	86	147	88	107.7	78	39.2	9	4.2	0.9	0.6	103	125	65	341	208	91	49	140.8	5.3	59.8	443	13.4	41.9	19.1	291	15.3	0.57	142	3.7	103	9.2	5.3	84	17.7	20.4	0.5	0.7
62	M	162.4	69.2	26.24	24.8	83	145	72	96.33	74	19.3	7.5	4.7	1.7	0.9	35	39	58	333	197	90	58	121	5.1	64.6	490	15.7	46.6	17.8	239	17.6	0.74	144	3.9	104	9.2	7.3	85	33.1	22.8	0.7	0.9
71	M	173	64.1	21.42	18.4	75	126	66	86	62	64.7	7.6	4.6	1.5	0.9	24	15	30	206	179	62	64	102.6	5.3	52.6	430	14.2	42.4	20.4	317	19.1	0.75	141	3.8	99	9.5	6.7	85	14.7	14.5	0.8	0.7
62	F	157.4	52.6	21.23	25.6	84	132	79	96.67	80	77.5	7.3	4.5	1.6	0.8	19	11	20	219	209	50	75	124	5.1	37.1	415	12.9	39.4	18.4	353	15.5	0.55	144	3.7	103	9.3	2.5	69	11.4	10.8	0.5	0.7
68	F	150.9	51.7	22.7	34.4	77	153	81	105	64	20.8	7	4.3	1.6	0.6	20	21	72	184	208	89	66	124.2	5.5	53.9	496	14.5	45.2	26.5	269	17.1	0.68	145	4.4	103	9.2	5.5	89	8.7	9.5	0.7	0.7
66	M	170.5	75	25.8	31.8	90	151	81	104.3	79	37.3	7.6	4.6	1.5	0.6	27	16	40	253	229	139	54	147.2	5.5	89.5	550	13.3	42.6	31.2	398	19.6	1.01	143	4.3	102	9.8	7.3	126	13.4	14.7	1.1	0.9
65	M	170.9	72	24.65	26.1	81	123	65	84.33	60	9.2	7.6	4.2	1.2	0.5	44	37	348	286	210	420	47	79	6.3	60.2	510	16.7	49.1	18.2	277	13.2	0.78	139	4.2	99	9.1	6	82	10.2	11.3	0.7	0.7
60	M	162.1	64.3	24.47	26.2	81	133	73	93	64	10.9	7.5	4.7	1.7	1	28	22	38	215	180	188	39	103.4	5.2	64.1	552	17.2	52.3	16.3	353	15.5	0.87	142	4.4	102	9.4	6.4	71	14.4	14.9	0.7	0.6
63	M	167.7	65.1	23.15	24.2	84	146	77	100	57	10.9	7.4	4.3	1.4	1.1	33	38	143	258	246	142	49	168.6	5.3	92.8	487	16.2	48.8	16.1	269	15.3	0.78	141	4.3	100	9.7	6.1	62	13.3	11.8	0.7	0.9
67	M	168.5	50.5	17.79	13.2	71.5	135	74	94.33	60	14.8	7	4.3	1.6	0.5	30	24	17	188	226	91	112	95.8	5.3	84.9	467	14.2	43.3	25.4	291	15.3	1	144	4.3	104	9.1	7.4	121	13.4	11.9	0.9	0.8
59	M	166.8	71.7	25.77	29.2	88	115	69	84.33	68	10	8.1	4.5	1.3	1.2	32	40	67	170	204	249	42	112.2	5.2	44.4	496	15.6	46.6	23.1	296	16.8	0.82	141	4.2	98	9.6	6.3	112	19.9	18.5	0.8	0.7
56	F	152.7	56.8	24.36	32	84	134	72	92.67	64	16.5	7.4	4.3	1.4	0.7	24	22	48	243	201	78	70	115.4	5.6	37.5	437	14.2	40.6	18.2	261	13.7	0.58	142	4.1	103	9.3	5.4	96	17.4	18.1	0.5	0.7
68	F	155.2	47.5	19.72	23.4	81	128	68	88	71	33.6	7.4	4.4	1.5	0.6	26	20	19	164	196	116	56	116.8	4.9	44.1	442	13.3	40.3	12.9	328	14	0.64	145	4.1	105	9.1	4.6	94	12.9	12.7	0.7	1
68	F	149.7	37.9	16.91	16.2	59	144	85	104.7	65	64.4	6.6	3.9	1.4	0.5	23	16	26	195	208	119	81	103.2	5	48.7	396	11.9	36.4	22.4	437	17.1	0.98	142	4.9	103	9.8	7	115	14.2	14.9	0.7	0.8
55	M	152.5	44.4	19.09	17.3	69	134	78	96.67	69	3.6	7.6	4.8	1.7	0.9	22	18	78	287	232	67	75	143.6	5	50.6	487	16.1	48.8	23	261	15.7	0.67	143	4.4	102	9.6	5.8	84	11.3	10.9	0.8	0.7
66	M	157.3	60	24.25	24.8	78	126	68	87.33	54	16.9	7.1	4.4	1.6	0.8	23	22	24	189	165	82	69	79.6	5.7	50.7	497	17.1	49.2	19.6	212	12.3	0.8	144	4.2	104	8.9	3.5	147	16.9	16.5	1.4	0.8
67	F	150.8	47.1	20.71	25.4	70	136	68	90.67	63	16.1	7	4.2	1.5	0.5	22	17	13	246	233	88	87	128.4	5.1	64.8	478	13.8	43	20.8	289	14.8	0.6	145	3.9	104	9.2	4.6	89	17.4	18.8	0.8	0.7
57	M	166.7	64.6	23.25	22.5	78	125	80	95	77	4.1	7.4	4.6	1.6	0.9	22	30	32	217	202	98	66	116.4	6.6	59.2	539	15.9	49.1	18.6	264	20.1	0.7	143	4.7	102	9.9	5.9	73	8.3	8.8	0.8	0.7
68	F	154.9	49.5	20.63	25.4	82	113	60	77.67	65	29.8	7.6	4.3	1.3	0.7	23	14	15	305	229	182	42	150.6	5	55.5	459	14	42.4	20.3	348	12.2	0.59	145	4.4	103	9.9	4	76	15.1	13.6	0.8	0.8
70	M	167.7	58.5	20.8	14.8	74	152	89	110	65	16.1	7.6	4.4	1.4	0.8	25	16	34	242	176	171	50	91.8	6	60.1	468	15	44.7	22.4	313	14.2	0.8	143	4	102	9.2	6.5	50	14.5	13.7	0.7	1.1
56	F	141.7	51.6	25.7	36.4	77	108	55	72.67	60	12	7.6	4.3	1.3	0.7	24	25	30	229	206	150	53	123	6.2	62.8	421	13.3	41.2	24.9	361	11.1	0.54	145	4.2	107	9.4	4.9	82	12.9	11.6	0.5	0.8
53	M	170.2	61	21.06	21.9	75	108	73	84.67	62	3.7	7.1	4.2	1.4	0.9	22	22	36	262	191	107	71	98.6	5.4	121.7	504	15.7	48.5	28.7	431	14.3	0.73	145	4.3	106	9.5	4.8	68	8	7.7	0.7	0.8
74	M	158.9	59.3	23.49	22.5	75.5	119	66	83.67	69	34.6	7.2	4.3	1.5	0.9	29	17	22	223	181	61	64	104.8	5.3	73.1	471	15.3	47.2	15.1	236	23.2	0.89	144	4.4	103	9.3	3.9	55	12.6	19.5	0.8	0.7
53	M	175.5	65.1	21.14	17.5	73	131	76	94.33	67	9.3	6.7	4.3	1.8	0.6	15	13	35	219	201	74	64	122.2	5	73.6	467	15.9	46.4	29.2	226	12.6	0.75	144	4.9	105	9.4	3.8	73	12.9	11.8	0.5	0.8
52	M	168.2	79.8	28.21	28.8	95	141	78	99	69	8	7.4	4.4	1.5	1.1	43	64	90	167	187	100	58	109	4.7	46.3	509	16	48.1	17.5	239	10.3	0.66	145	4	104	9.1	6.9	50	15.4	17.3	0.8	0.8

65	F	153.3	57	24.25	33.2	72	117	65	82.33	65	19.1	7.4	4.1	1.2	0.5	20	16	17	230	228	200	73	115	5.2	39.7	450	14	43.1	19.1	253	13.6	0.68	145	4	104	9.3	3.9	59	10.8	13.3	0.7	0.8
65	F	158.5	55.3	22.01	27.7	82	138	76	96.67	74	10.5	7.2	4.5	1.7	0.5	33	29	60	191	210	56	73	125.8	4.7	38.7	447	14	42.9	14.8	318	10.3	0.56	146	4.5	104	9.7	3.3	73	14.8	11.5	0.7	0.7
52	F	160.4	52	20.21	27.3	74	138	68	91.33	62	11.9	7.3	4.8	1.9	0.5	29	44	78	254	253	92	93	141.6	6	53.6	452	13.1	40.9	32.4	285	12	0.51	142	4	101	9.5	4.9	80	14.1	14.4	0.5	0.7
60	M	176.8	78	24.95	22.7	86	135	72	93	63	18.7	7.3	4.3	1.4	0.8	26	20	32	184	246	166	64	148.8	6	63.7	473	14.4	43.8	20.6	264	14.5	1.18	142	4.2	103	9.6	8.5	92	12.5	12.9	0.7	0.7
66	F	152.6	50.1	21.51	28.9	77	130	67	88	81	9.7	6.7	4.2	1.7	0.7	20	21	16	233	216	112	63	130.6	5.1	57.7	457	13.9	43.4	21.7	261	11.7	0.81	142	4.4	102	9	3.5	110	12.8	11.4	0.7	0.7
41	F	153	55.4	23.67	36.9	75	128	81	96.67	70	4.2	7.7	4.6	1.5	0.7	25	24	17	166	191	84	82	92.2	4.9	62.9	476	14.7	44.7	24.5	261	16.2	0.63	142	3.9	103	9.6	4.6	79	17.4	14.1	0.7	0.7
58	M	161	58.9	22.72	20.5	81	135	70	91.67	62	22.1	6.9	4.5	1.8	0.6	22	21	23	159	215	98	73	122.4	8	57.5	521	15.6	46.5	16.3	298	13	0.7	144	4.9	104	9.5	4.3	82	14.6	16.4	1.8	0.8
57	F	155.4	63.8	26.42	39.2	99	122	71	88	86	2.8	7.7	4.2	1.2	0.8	19	20	66	929	228	119	78	126.2	5.3	48	492	15	44	27.9	348	11.2	0.52	146	3.9	104	9.6	4.8	84	17.3	18.2	0.5	0.7
62	F	149.4	48.8	21.86	28.3	77	108	60	76	57	16.5	7.6	4.5	1.4	0.8	36	48	34	227	253	66	84	155.8	5.6	53.3	459	14.6	43.8	15.3	264	15.3	0.81	143	4.3	103	9.5	3.1	87	7.9	9.1	0.7	0.7
55	F	159.4	77.6	30.54	41.2	91	145	84	104.3	81	40.3	7.6	4.5	1.4	0.6	22	24	46	320	205	192	45	121.6	6	54.7	420	12.7	39.5	19.7	324	13.3	0.53	143	4	105	9.2	5.2	58	17.9	15.1	0.7	0.7
65	F	154.1	52.2	21.98	29.7	73	119	69	85.67	54	19.5	7.8	4.4	1.3	0.5	20	17	14	175	226	57	63	151.6	5.5	57.4	439	12.9	40.6	20.4	329	15.2	0.59	144	3.9	102	9.5	2.7	118	14.1	15.1	0.7	0.8
73	F	150.8	51.5	22.65	27.1	75	110	67	81.33	120	77	7.6	4.3	1.3	0.9	30	32	34	341	133	62	62	58.6	9.8	63.9	493	15.2	45.1	14.1	285	17.7	0.62	140	4.4	99	9.4	2.9	78	13.6	12.1	0.8	0.9
45	F	159.7	52.8	20.7	26.6	73	104	55	71.33	60	5.4	7.2	4.3	1.5	0.6	21	16	13	165	208	85	66	125	5.1	62.8	485	13.5	41.8	25.3	231	9.6	0.61	142	3.9	104	9.1	3.7	100	10.2	12.5	0.8	0.5
59	F	156.9	53.8	21.85	28.9	69	150	84	106	68	13.5	7.4	4.7	1.7	0.8	15	12	15	235	221	154	66	124.2	5.7	58.1	442	13.7	41.5	21.1	308	11.2	0.67	144	3.9	105	9.5	3.9	63	13.4	11.7	0.5	0.7
55	F	153.9	51.5	21.74	26.9	66	118	76	90	69	5.4	7.1	4.6	1.8	0.9	18	15	21	268	182	60	78	92	5.7	67.8	490	13.7	43.7	21.8	264	19.6	0.58	146	5.1	107	9.6	3.8	93	13.1	12	0.5	0.7
63	M	166.2	67.8	24.55	21	83	110	55	73.33	68	9	7.6	4.4	1.4	1.1	21	17	29	198	223	81	52	154.8	5	56.1	462	16	47.6	23.1	216	13	0.77	144	3.6	102	9.1	4.8	113	8.9	8.3	0.7	0.7
43	M	166.3	68.7	24.84	31	86	121	71	87.67	62	3.7	7.3	4.2	1.4	1.2	37	104	146	259	254	72	70	169.6	5	61	514	15.1	45	25.1	244	20.6	0.91	143	4.2	105	9.4	7.8	98	9.5	9.8	0.7	0.9
62	F	149.3	52.1	23.37	34	82	112	63	79.33	50	49.9	7.4	4.5	1.6	1.1	33	32	30	212	229	105	67	141	6.3	65.8	455	13.7	41.5	24.8	272	15.2	0.68	141	4.5	103	9.6	3.9	123	13.6	15.2	0.5	0.7
68	M	162.5	55.3	20.94	17.5	76	127	60	82.33	91	8.1	7.3	4	1.2	0.4	20	23	37	200	184	157	49	103.6	4.8	31.9	410	12.2	38.1	23.2	348	13.3	0.69	140	4.5	102	9.3	6	96	10	10.4	0.7	0.8
68	M	167.9	68.2	24.19	20.8	85	117	63	81	65	150	7.5	4	1.1	0.8	26	15	35	166	167	54	65	91.2	5	49	473	14.7	45.4	15	264	14.2	0.81	143	4	103	9	6.1	66	16.3	10.3	0.7	0.5
68	M	164.7	65.7	24.22	25.3	77	121	64	83	65	31.7	7.5	4.5	1.5	0.7	16	15	56	231	196	111	49	124.8	4.8	66.1	470	14.4	43	26.8	298	17.7	0.69	141	4.6	102	9.5	4.7	62	10.3	11.1	0.7	0.8
69	M	162.1	66.3	25.23	30	83	123	74	90.33	62	26.7	7.1	4.2	1.4	0.9	24	21	58	156	195	131	57	111.8	5	76.3	465	14.9	43.7	27.1	341	9.1	0.68	139	4.3	97	9.5	5.9	75	16.3	17	0.7	0.7
57	F	153.9	48.9	20.65	27.3	83.5	110	67	81.33	85	7.1	7.6	4.4	1.4	0.5	24	27	20	242	221	89	61	142.2	5.2	49.3	426	12.8	38.4	22.3	276	16.9	0.62	140	4.6	102	9.4	5.7	65	7.9	9.2	0.7	0.7
59	F	159.1	60.5	23.9	35.2	91	122	68	86	57	41	8.2	4.4	1.2	0.4	15	14	16	317	225	116	56	145.8	6.2	71.5	511	14.7	44.9	26.1	341	12.7	0.72	143	4.5	104	9.8	6.6	77	19.1	13.9	0.7	0.7
67	F	148.2	55	25.04	38.7	90	153	82	105.7	54	33.7	7.7	4.4	1.3	1	22	18	16	323	241	114	55	163.2	5.2	71	487	14.9	45.9	30.6	285	14.3	0.45	143	3.8	101	9.2	4.7	87	15.8	11.2	1.3	1.5
79	M	160.5	65.1	25.27	18.6	83	138	76	96.67	57	91.2	7.3	4.2	1.4	0.6	31	27	32	142	188	77	68	104.6	6.1	47.5	452	15.2	45.6	16.6	298	20	0.85	142	4.8	101	9	7.6	67	17.2	17.1	0.8	0.9
66	F	153.3	50.2	21.36	27.6	86	137	74	95	71	9.3	7.9	4.3	1.2	0.5	25	24	17	264	232	106	55	155.8	5.4	46.6	431	13.3	41.4	19.7	285	14.1	0.73	142	4.1	101	9.4	5.3	84	12.8	15	0.7	0.8
64	F	150.1	54.7	24.28	32.4	87	160	85	110	64	4.7	7.4	4.4	1.5	0.7	21	24	42	220	251	161	62	156.8	5.5	59.1	469	14.6	44.2	25	313	16	0.57	144	3.8	103	9.4	4.6	79	17.4	13.6	1.3	1.4
57	M	164.1	62.2	23.1	23.2	79.5	145	77	99.67	60	24	7.6	4.4	1.4	1	23	18	27	232	137	58	58	67.4	6.1	69.8	434	13.3	40.8	27.5	388	13.6	0.74	145	4.4	104	9.5	4.9	86	17.8	17.6	0.9	0.9
52	F	152.1	51.6	22.3	31.1	77	112	65	80.67	69	3.3	7.9	4.5	1.3	0.6	23	17	23	123	254	144	53	172.2	5.5	58.5	427	13.5	41.8	37.3	329	12.9	0.74	144	4.2	102	9.9	5.1	83	14.3	15.1	0.5	0.8
57	F	156.6	42.3	17.25	21.5	70	150	68	95.33	76	12.1	7.4	4.5	1.6	0.8	21	13	13	188	233	73	90	128.4	5.5	61.3	486	14.5	44.4	28.6	368	18.7	0.63	143	4.7	101	9.9	4.3	57	12.9	13.5	0.7	0.5
66	F	159.5	49.9	19.61	23	62.5	148	81	103.3	70	15.5	7.9	4.5	1.3	0.8	23	15	12	342	292	111	82	187.8	5.6	53.3	476	14	44.4	31.8	261	15.1	0.6	143	4.5	102	9.6	3.7	107	16.3	12.9	1.2	0.9
69	F	149.5	50.2	22.46	28.8	85	118	57	77.33	72	28.4	7.3	4.3	1.4	0.6	30	18	13	187	248	38	89	151.4	5.8	46.4	396	12.1	37.7	14.2	289	17.4	0.48	144	3.8	103	9.2	3.6	45	13.5	11.3	0.8	0.8
56	F	158.8	54.5	21.61	26.7	70.5	159	80	106.3	63	9.5	7.6	4.7	1.6	0.9	25	33	45	163	226	212	67	116.6	6.7	59.2	446	13.6	41.9	33.5	261	14.8	0.59	144	4.2	101	9.8	6.5	77	14.8	15.1	1.3	1.4
68	F	151.8	52.4	22.74	26.8	87	147	77	100.3	101	4.8	7.6	4.3	1.3	0.5	28	25	134	289	178	92	78	81.6	5.5	68.3	420	13.1	39.9	23.2	324	12.9	0.62	144	4	102	9.3	5.4	65	18.2	20.8	0.7	0.9
62	F	147.7	52.1	23.88	29.2	83	128	75	92.67	61	9.9	7.8	4.4	1.3	0.6	18	14	20	205	232	169	71	127.2	5.7	55.2	380	12.5	37.6	24.7	341	16.3	0.64	144	4.1	104	9.1	4.4	89	15	12.7	0.8	0.7
64	F	156.9	50.9	20.68	27	74.5	151	74	99.67	65	10.6	7.4	4.4	1.5	0.6	20	15	22	277	253	94	72	162.2	5.5	40.3	494	14	43.7	22	341	10.4	0.49	145	4.2	102	9.5	3.5	95	14.7	14	0.7	0.8
52	F	153.5	54.1	22.96	31.9	78	130	78	95.33	70	2.3	7.6	4.3	1.3	0.5	22	30	134	224	226	109	66	138.2	5.4	49.1	458	13.7	42.2	17.4	289	19.1	0.66	144	3.4	101	9.8	5.1	75	13.9	14.1	0.5	0.5
50	M	178.5	100.2	31.45	32	100	124	61	82	55	2	7.4	4.5	1.6	0.9	43	75	84	177	228	148	61	137.4	5.7	59.4	592	18.2	54.8	19.1	219	13.6	1.06	142	4.2	103	9.1	5.5	74	13	11.4	0.5	0.5
68	M	162.1	71.5	27.21	20.7	89	161	81	107.7	75	17.4	7.3	4.4	1.5	0.4	29	41	103	174	233	217	67	122.6	6.5	60.1	422	14.1	42	15.6	276	21	0.99	141	4.4	105	9.2	7.2	53	12.1	12.3	0.7	1.1
59	F	169.3	58.8	20.51	26.6	77	100	61	74	60	21	7.3	4.6	1.7	0.9	15	12	27	261	242	98	72	150.4	5.7	85.7	514	16.2	49.3	18.9	318	15	0.74	142	4.5	103	9.5	5.1	71	16.2	15.7	0.8	0.7
56	F	147.3	52.3	24.1	34.4	76.5	130	71	90.67	66	4.1	7.4	4.5	1.6	0.4	22	16	41	333	181	118	54	103.4	5.1	61.7	477	14.6	43.8	22.6	324	14.1	0.46	141	4.3	103	9.6	4	107	11.4	12.5	0.7	0.7
58	M	156.1	49.9	20.48	12.7	57	114	60	78	46	67.8	7.3	4.4	1.5	0.8	21	13	16	178	223	84	87	119.2	5.4	59.7	463	14.8	45.7	19.8	289	16.4	0.75	146	4.8	107	9.4	5.7	70	16.5	17.1	0.7	0.7
58	F	150.1	44.4	19.71	24.6	64	105	50	68.33	63	6.7	7.2	4	1.3	0.7	14	13	14	198	245	107	57	166.6	5.7	80.3	413	12.1	37.8	28.5	313	14.6	0.51	142	4.1	107	9	4	54	7.7	8.1	0.9	1.1
62	M	174.5	84.6	27.78	36.4	96	154	75	101.3	74	2.4	7.7	4.6	1.5	0.6	25	34	150	219	228	441	53	86.8	6.2	60.7	517	16	48.2	21.7	206	15.7	0.85	140	4.3	104	9.8	9.3	61	12.6	13.4	0.7	0.7
67	M	162.5	61.8	23.4	24.5	80.5	147	64	91.67	83	14.4	7.8	4.7	1.5	1.7	34	43	205	231	245	119	100	121.2	5.2	46.9	403	14.6	43.6	17.5	268	14	0.65	139	4.1	101	9.6	4.1	43	9.7	9.9	0.8	1.6
69	M	170.1	66.6	23.02	21.6	86	151	73	99	60	4.7	7.2	4.5	1.7	0.5	26	32	89	119	208	234	58	103.2	7	48.7	498	15.8	48.7	17.6	276	16.6	1.01	145	3.8	102	9.4	7.3	66	16.4	15.1	0.8	0.7
68	F	145.3	52	24.63	32.3	84	114	65	81.33	59	46.5	6.6	4	1.5	0.5	15	11	19	277	203	87	100	85.6	5.5	70.6	407	12.1	37.7	33.3	361	16.4	0.47	145	4.3	107	9	3.8	54	14	12.3	0.7	0.7
66	F	156.3	53	21.69	30.3	83	121	62	81.67	63	18.7	7.4	4.6	1.6	0.6	23	12	15	92	247	137	81	138.6	5.7	43	412	13	40.2	20.8	348	14.4	0.55	144	5	105	9.6	4.2	76	9	8.4	0.8	1.1
58	F	150.9	64.5	28.33	42.8	90.5	136	69	91.33	92	94.3	8.3	4.5	1.2	0.3	19	14	17	248	208	67	60	134.6	5.5	34.2	426	12.3	37.7	14.3	341	18.5	0.53	143	3.8	106	9.1	5.3	53	15.9	13.8	0.5	0.5
42	F	152.5	57.3	24.64	35.2	77	116	77	90	64	21.7	6.9	4.3	1.7	0.6	33	40	122	171	225	155	102	92	5.1	86.3	416	13.5	41.1	36.3	236	11	0.42	141	3.9	104	8.9	5.9	42	14.4	13.4	0.5	0.5
69	M	174.8	71.2	23.3	22.8	83.5	138	78	98	62	9.8	6.9	4.5	1.9	0.9	19	15	23	198	178	70	80	84	5	34.7	506	15.6	46.5	14.2	180	17.4	0.81	144	4.5	107	9	6.7	104	10.4	7.9	0.8	1
62	M	172.3	62.5	21.05	18.8	81	124	62	82.67	54	5.6	7.1	4.3	1.5	0.6	32	21	40	195	228	60	93	123	5.4	40.3	452	14.7	45.1	24.9	401	19.2	0.75	142	4.1	104	9.2	5.7	90	10.8	9.2	0.7	0.8
66	F	147.7	69.4	31.81	41.3	98	121	72	88.33	84	21.7	8.1	4.5	1.3	0.6	26	21	9	176	168	102	53	94.6	5.9	90.6	422	12.9	40.2	26.5	293	13.1	0.56	144	4.5	104	9.8	4.6	122	17.5	17.3	0.6	0.6
56	F	159.6	56.1	22.02	30.2	75.5	129	67	87.67	72	4.3	7.5	4.4	1.4	0.9	22	32	30	194	250	323	56	129.4	4.9	49.7	462	14.6	42.4	21.5	268	12.7	0.53	141	3.6	101	9.7	2.3	63	11.8	16.2	0.8	0.7
68	F	145.7	42.9	20.21	23.2	69	168	79	108.7	111	13.3	8	4.6	1.4	0.6	31	16	21	452	219	71	91	113.8	5.3	40.1	495	14.4	45.3	23	276	7.8	0.61	142	3.4	101	9.5	3.9	87	18.5	20.4	0.7	0.7
60	F	152.6	50.9	21.86	27.9	73	140	83	102	57	7.4	7.3	4.4	1.5	0.4	13	8	19	228	213	132	47	139.6	5.7	57.4	465	12.8	40.5	27.2	298	22.8	0.7	144	4.1	108	9.2	4.2	218	14.8	14.6	1.2	1.2
59	M	178.1	83.6	26.36	29.3	92	132	75	94	61	10.3	7.4	4.6	1.6	1.4	35	25	97	179	152	54	98	43.2	5.6	62.2	493	16.4	49.2	29.5	233	12.5	0.81	143	4	106	8.7	6.6	65	11	13.6	0.8	0.7
69	M	167.7	67.1	23.86	23	83	142	77	98.67	80	22.2	6.9	4.3	1.7	0.8	24	19	34	150	204	125	55	124	5.2	53.2	437	14.3	44.1	26.2	298	15.3	0.9	146	3.9	107	8.9	5.7	160	17.7	18.4	0.7	0.7

66	F	151	55.4	24.3	31.2	82	117	64	81.67	69	3.5	8.1	4.7	1.4	0.7	20	13	20	297	221	92	68	134.6	5.6	78.2	475	14.1	42.5	22.1	335	11.4	0.64	142	3.6	100	9.3	4.4	93	12.4	10.7	0.7	0.7
69	M	163.3	51.9	19.46	16.8	74	145	76	99	61	6.9	7.3	4.2	1.4	1.2	24	17	24	163	174	90	54	102	4.7	49.5	421	13.6	42.1	19.7	341	16.4	0.88	142	4.1	103	9.1	5.9	87	15.9	10.8	0.8	1.5
63	F	153.9	46.6	19.67	23.6	65	114	65	81.33	81	7.9	7.3	4.8	1.9	0.9	24	26	21	213	212	48	70	132.4	5	51.1	456	14.4	44	19.9	261	15.4	0.7	145	4.4	106	9.8	4.9	103	8.3	10	0.5	0.7
71	F	150.5	42.9	18.94	21.4	64	95	55	68.33	67	15.4	7.8	4.6	1.4	0.6	23	15	20	128	231	63	68	150.4	6	37.5	440	13.3	41.2	16.4	261	12.3	0.7	142	4.6	101	9.9	4.5	139	10.7	10.7	0.7	0.8
64	F	153.3	47.5	20.21	22.9	73	145	70	95	98	139	7.8	4.6	1.4	1	25	22	21	183	158	63	83	62.4	5.5	37	484	14.3	44.4	13.9	293	13.5	0.88	142	4.4	103	9.4	5.1	228	14.3	18.1	0.7	0.7
63	M	166.5	62.8	22.65	20.7	86	120	72	88	62	12.3	7.5	4.6	1.6	0.7	17	13	15	229	200	91	51	130.8	5.3	65.2	440	14.3	43.3	19.2	431	10.7	0.88	142	4.3	103	9.2	6.4	112	12.3	11.1	1.1	0.7
60	F	165.7	70.9	25.82	34.1	88	138	71	93.33	51	10	7.4	4.3	1.4	0.5	22	27	44	268	189	86	56	115.8	5.5	35.8	440	13.2	40.7	26.2	272	9.8	0.49	142	4.2	106	9	4.5	54	9.3	11.3	0.7	0.8
64	M	159.3	67.8	26.72	26.4	86	156	90	112	59	13.1	7.4	4.6	1.6	1	28	28	44	169	247	118	66	157.4	5.5	83.9	524	16.4	48.7	20	289	12.8	0.74	141	3.9	103	9.2	4.6	56	15.9	19.1	0.8	0.7
66	F	150.5	48.6	21.46	30.8	71	152	77	102	71	17.5	8.1	4.4	1.2	0.6	26	19	20	272	228	130	68	134	5.3	53.7	456	13.6	42.4	20.8	298	18	0.74	141	3.9	104	9.3	4.5	69	15.7	15.5	0.7	0.5
69	F	149.3	48.1	21.58	26.7	70	117	58	77.67	65	11.3	7.6	4.6	1.5	0.9	26	23	17	224	188	49	67	111.2	5	36.1	431	13.7	41.7	18	272	14.9	0.66	143	4.6	105	9.4	3.8	115	12.2	16.8	0.8	0.7
66	F	154.3	58.5	24.57	34.6	96	163	65	97.67	99	13.7	7.8	4.7	1.5	0.6	24	32	46	342	215	193	70	106.4	6.2	81.9	511	15.2	46.8	25.2	375	10.8	0.61	142	3.9	104	9.6	4.6	82	19.1	17.7	1.2	1.1
54	F	160.5	66.3	25.74	41.6	86	115	67	83	72	15.9	7.9	4.6	1.4	0.7	29	47	98	261	245	262	51	141.6	5.3	60.8	531	15.7	46.6	24	250	9.3	0.46	144	3.9	101	9.8	4.8	54	15.4	15.9	0.5	0.7
67	F	155.7	65.3	26.94	27.9	80	131	89	103	141	467	7.7	4	1.1	1.3	21	25	204	342	112	105	31	60	8.2	68.1	459	13.2	41.4	18.1	368	21.8	0.82	144	4.3	104	9.6	8.2	71	15.8	15.5	0.8	1.316
64	M	159	51.7	20.45	15.4	74.5	139	75	96.33	49	22.1	6.9	4.2	1.6	0.8	29	18	124	124	207	153	76	100.4	4.9	51.6	399	14.3	42.8	22.1	348	19.1	0.71	144	3.9	104	8.9	6.2	66	14	13.9	0.7	0.7
80	M	173.1	74.9	25	24.8	93	138	68	91.33	91	7.3	7.7	4.1	1.1	0.6	28	27	27	264	196	73	62	119.4	5.8	70.1	465	15.4	45.9	21.1	529	17.2	0.73	142	4.4	101	9.4	5.9	140	11.2	10.8	0.8	1.2
73	F	151.4	51.5	22.47	32.3	87	138	65	89.33	64	46.5	7.3	4.4	1.5	0.8	19	15	16	173	215	57	69	134.6	5.4	65.2	481	14.7	44.9	23.2	261	15	0.54	144	4	105	9.6	4.2	51	18.6	16.9	0.8	1.2
69	M	161.7	60.7	23.21	19.2	80	155	68	97	54	90.6	7.2	4.2	1.4	0.4	16	15	36	302	179	183	41	101.4	8.9	104.3	497	15	45.8	23.9	375	18	1.06	142	4.5	102	9.4	4.3	50	9.3	10	1.2	1.6
68	M	168.1	69.1	24.45	22.8	88.5	128	78	94.67	47	27.1	7.4	4.3	1.4	1	29	39	16	276	212	125	50	137	5.4	70.5	578	17.2	50.5	21.3	303	14.2	1.14	143	4.3	104	9.6	6.3	75	15.7	16.2	0.8	0.7
60	F	149.7	54.7	24.41	31.5	83	121	70	87	61	9	7.1	4	1.3	0.5	18	15	16	172	205	67	51	140.6	5.7	64.5	440	13	41.2	24.6	368	13.1	0.63	143	4.1	105	9.1	5.7	50	11.9	11.5	0.7	0.8
76	F	150	60.3	26.8	35.2	76	129	75	93	58	28	8.1	4.4	1.2	0.6	23	14	16	380	222	94	76	127.2	5.4	50.4	506	13.5	43.3	30.9	308	12.9	0.66	140	4.9	103	9.6	5.7	101	14.6	15.8	0.7	0.8
77	M	159.3	50.1	19.74	13.6	73	137	78	97.67	68	36.1	7	3.7	1.1	0.7	22	10	13	300	166	61	64	89.8	6.2	63.8	330	11.8	36.9	26.8	494	14.9	0.54	139	4.5	99	8.6	2.7	126	7.9	9.1	1.7	0.9
70	F	148.3	57.2	26.01	35.1	81	156	92	113.3	91	4.5	7.5	4.6	1.6	0.7	24	26	43	188	195	172	63	97.6	5.2	46.2	439	14.4	43.5	26.1	324	10.7	0.8	142	4.3	101	9.2	6.2	63	12	12.6	0.5	1.3
72	M	161.7	73.4	28.07	22.1	95	123	77	92.33	73	11.9	7.5	4.3	1.3	0.6	36	36	39	316	183	215	39	101	5.3	46.3	479	14.5	44.4	20.8	341	11.8	0.77	140	4.3	102	9.3	5.3	105	7.9	8.7	0.9	0.7
51	M	169.9	86.8	30.07	38.1	105	142	88	106	57	5	7.4	4.5	1.6	0.7	54	48	51	272	219	196	45	134.8	4.9	93.1	463	15.7	45.5	19.6	335	10.9	0.87	141	4.5	105	9.5	4.6	54	14.9	13.1	0.5	0.7
29	M	176.5	76.6	24.59	22.4	79.5	119	70	86.33	64	2	7.7	4.6	1.5	0.9	28	21	16	210	178	54	40	127.2	4.9	52.9	514	15.8	47.9	24.5	239	15.8	0.86	141	4.3	101	9.4	5.8	76	15.8	17	0.5	0.7
71	F	146.2	40.3	18.85	26.9	68	168	92	117.3	67	54.4	7.9	4.5	1.3	0.6	30	23	22	200	215	140	68	119	5.4	62.8	455	12.9	41	32.5	268	14.2	0.59	139	4.1	100	9.7	4.9	99	10.8	10.8	0.7	0.7
62	M	165.7	75.9	27.64	31.7	98	144	79	100.7	57	44.6	6.5	4.1	1.7	0.8	16	21	30	317	173	130	40	107	5.6	81.9	494	15.2	46.4	20.8	253	16.8	0.77	143	4.3	106	8.8	4.8	78	8	7.9	0.9	0.7
53	M	174.5	89.2	29.29	28.4	100	137	75	95.67	58	5.2	7.4	4.6	1.6	0.7	54	98	94	281	185	241	33	103.8	5.8	73.4	550	17.1	50.8	15.1	228	10.9	0.98	140	4	102	9.1	3.7	202	15.9	15.9	0.7	0.7
71	F	135.3	41.9	22.89	29.6	75	103	57	72.33	75	29.5	7.4	4.3	1.4	0.8	40	44	23	199	207	70	68	125	4.9	37.3	448	14.4	42.1	9.6	231	20.3	0.5	141	4.2	105	9.1	2.7	87	9.5	10.7	0.7	0.7
78	F	149.2	52.3	23.49	27.9	89	107	53	71	53	43.3	6.9	4	1.4	0.5	39	20	32	169	218	103	64	133.4	5.4	74.9	454	13.4	40.7	19.5	298	21.6	0.48	144	4.8	108	8.6	4.4	52	9.7	10.1	1.3	1.3
71	F	155.9	69.1	28.43	37.1	85.5	150	82	104.7	58	46.6	7.4	4.5	1.6	1.2	20	20	18	192	196	117	67	105.6	5.4	58.1	443	13.4	40.8	19.3	253	15.6	0.56	143	4.3	105	9.7	4.1	48	12.8	14	0.9	0.9
66	M	168.5	65.8	23.18	24.5	80.5	151	86	107.7	54	26.2	7.7	4.7	1.5	1.4	24	17	32	203	233	101	68	144.8	6.7	64	482	15.1	46	20.9	361	20.7	0.88	141	4.7	105	9.6	7	49	12.9	10.8	0.9	0.8
48	M	164.1	64	23.77	27.2	82.6	117	66	83	57	8.1	6.6	4.2	1.7	0.7	21	27	71	173	214	232	51	116.6	4.8	101.8	521	16.9	46.5	24.1	313	13.8	1.02	143	4.7	105	9	8.8	74	21.3	21.4	0.8	0.7
46	M	163.6	68.3	25.52	23.2	85	129	84	99	90	2.6	7.6	4.7	1.7	0.7	28	50	111	242	220	330	60	94	5.1	71.1	492	14.7	43.1	18	280	13.4	0.87	144	4	106	9.3	5.1	63	14.4	13.2	0.5	0.5
75	M	168.9	58.9	20.65	19.1	78	131	71	91	71	12.9	7.1	4.1	1.3	0.6	28	20	24	174	165	66	41	110.8	8	56.9	460	13.8	42.5	18.7	253	18.8	1.05	145	4.5	110	9.1	6.7	68	12.5	8.2	0.6	1.6
59	F	155.4	64.5	26.71	41.9	92.5	157	99	118.3	75	10.3	7.8	4.5	1.3	1.3	27	27	118	316	248	101	87	140.8	4.8	54	469	14.2	42.9	36.4	206	14.4	0.57	142	4.5	106	9.2	6	75	11.8	12.5	0.6	0.7
58	F	152	48.8	21.12	27.4	66.5	124	62	82.67	65	15.9	7.5	4.3	1.3	0.4	20	13	12	216	198	37	73	117.6	5.5	33	414	12.2	38.1	16.8	276	11.1	0.62	144	4.4	106	9.4	4.5	128	7.6	7.6	0.7	0.7
44	M	180.3	60.4	18.58	12.5	71.5	103	63	76.33	61	10.3	6.9	3.9	1.3	0.6	30	27	33	219	203	67	53	136.6	5	41.8	446	13	41.4	17	354	13	0.83	144	4.2	106	9	5	133	12.5	13.4	0.5	0.6
54	F	161.7	62	23.71	28.8	76.5	122	63	82.67	60	17.3	6.9	4.2	1.5	0.6	19	17	16	163	228	86	84	126.8	5.3	51	429	13.4	42	19.1	289	9.7	0.59	143	3.9	106	9.1	4.8	79	13.7	13.8	0.7	0.8
63	F	165.7	61	22.22	24.4	82	117	63	81	71	48.1	7.1	4	1.3	1.2	23	16	12	258	216	71	74	127.8	5	35.4	425	12.4	39.1	20.8	236	12.4	0.59	143	4.7	105	9.3	3.4	57	9.1	11.6	0.9	0.7
66	F	149.3	49.3	22.12	28.3	78	99	66	77	66	10.1	7.3	4.1	1.3	0.6	25	17	43	242	191	81	43	131.8	5.2	53.7	455	13.4	41.1	22.9	298	13.9	0.54	143	4.4	107	9.2	4.3	73	13.7	9.9	0.5	0.7
64	M	157.1	63.9	25.89	30.2	92	164	94	117.3	56	33.2	7.2	4.3	1.5	0.9	23	25	34	146	194	87	63	113.6	5.5	53.1	457	13.8	43.4	16.2	261	16.5	0.96	143	4.6	106	9.3	8.2	48	11	10	0.9	0.8
66	F	149.9	57.4	25.55	32.7	84.5	124	73	90	78	6.1	7.4	4.4	1.5	0.9	31	35	51	249	275	146	74	171.8	6.7	60.3	437	13.3	39.9	25	288	10.7	0.55	140	4.2	103	9.3	4.5	92	10	10.4	0.9	0.9
68	M	164.1	69.7	25.88	27.3	88.5	126	76	92.67	58	14.8	7	4.2	1.5	1.3	26	27	76	199	231	235	47	137	5	44.6	507	15.4	45.8	19.5	239	12.8	0.6	140	4.4	103	8.9	5.8	72	12	13.2	0.9	0.9
49	M	170.9	71.9	24.62	21.8	81.5	129	85	99.67	64	5.9	7.7	4.4	1.3	0.7	23	17	56	165	188	73	65	108.4	5.2	53.4	482	14.4	44.4	22.1	293	13.3	1.03	140	4.9	104	9	6.4	62	12.3	11.6	0.7	0.7
50	M	171.7	84	28.49	23.8	84	144	94	110.7	76	6.4	6.9	4.2	1.6	1	16	15	35	169	182	98	43	119.4	5.2	47.8	447	14	42.8	15.2	228	19.3	1.01	143	4.1	108	9.2	5.7	65	12.2	13.4	0.5	0.5
68	M	172.7	77.4	25.95	28.7	95	150	80	103.3	54	39.1	7.6	4.2	1.2	1.5	19	16	35	161	231	83	52	162.6	5.2	50.1	469	14.9	45.4	26.7	339	14.9	0.9	141	4.2	104	9.1	6.4	86	12.8	11.1	0.7	0.9
66	F	152.5	40.9	17.59	22.7	76.5	118	65	82.67	81	17.5	7.4	4.2	1.3	0.7	24	17	26	300	218	82	79	122.6	6.1	58.5	461	13.3	41.2	25.7	266	19.1	0.41	144	4	106	9.1	3.3	69	14.2	15.1	0.9	0.7
62	M	162.9	50.7	19.11	17.3	65	118	69	85.33	68	44.2	7.3	4.2	1.4	1.1	24	21	48	154	208	77	81	111.6	5.1	64.4	465	14.4	44	19.5	298	13.1	0.93	143	4.1	106	8.7	5.9	72	12.9	12.2	0.6	0.7
54	F	150.3	51.6	22.84	33.5	71.5	125	76	92.33	73	26.8	7.2	4.3	1.5	0.5	20	14	19	248	198	61	63	122.8	5.5	51.6	450	13	40	20.5	353	16.4	0.65	144	4.2	104	9.2	6	69	10.4	9.7	0.5	0.6
74	M	170.8	78.4	26.87	25.6	98.5	138	70	92.67	50	146	8	4.7	1.4	0.9	36	48	75	122	170	110	69	79	5.4	73.1	543	17.1	50.7	15.8	239	13.1	0.77	144	3.9	106	9.5	8.1	71	16.9	9.8	0.7	0.8
66	M	170.5	62	21.33	15.3	81	141	84	103	60	34.1	7.2	4.4	1.6	1.3	37	21	54	194	161	161	37	91.8	4.9	66.1	395	13.4	40.5	25.5	293	17.3	0.77	143	4.3	107	9	8.3	51	11	12.1	0.7	0.7
65	F	152.2	51.7	22.32	28.5	74.5	177	102	127	78	42.1	7.3	4.5	1.6	0.5	22	11	15	154	217	87	61	138.6	5.3	36.3	398	11.6	35.9	28.5	241	17.8	0.7	144	4	108	9.7	5.4	70	12.9	11.8	0.8	0.7
66	M	162.6	67.9	25.68	26.5	89	150	88	108.7	64	9.4	7	4.5	1.8	1.3	35	57	50	220	200	197	44	116.6	5.3	78.1	526	16	47.3	18.8	266	20.5	1.15	143	3.9	109	9	5.8	56	9.8	12.1	0.7	0.8
68	M	164.5	76.5	28.27	27	87	136	79	98	72	33.4	7.4	4.3	1.4	0.7	22	24	67	198	190	410	40	68	5.4	41.9	437	14.7	41.3	15.9	284	20.7	0.99	139	4.4	103	9.5	6.9	91	14.5	14	0.8	0.9
61	F	158.2	72.4	28.93	38.7	91.5	149	94	112.3	66	53.9	7.1	4.3	1.6	0.6	21	16	27	515	246	63	75	158.4	5.5	40.4	446	13.2	40	16.3	309	20	0.7	144	4.4	107	9.2	5.4	76	14.8	13.6	0.8	0.7
65	F	150.5	53.1	23.44	29.7	72	133	73	93	69	14.7	7.1	4.5	1.7	0.9	34	36	42	343	174	64	96	65.2	5.3	62.3	470	14.2	44.1	22.4	239	10.2	0.62	142	4.6	104	9.2	4.7	99	16	15.3	0.7	0.7
62	F	155.3	46.6	19.32	23.9	66.5	127	74	91.67	71	11.3	7.7	4.3	1.2	0.5	21	9	14	218	258	58	69	177.4	5.1	36.2	428	12.5	39.2	22	411	10.6	0.46	143	4.1	107	9.1	4.3	77	14	11.2	0.7	0.8
66	M	172.7	64.6	21.66	19.2	76	141	90	107	59	9.6	6.7	4.2	1.7	0.6	25	17	119	197	217	108	60	135.4	5.1	53.3	437	14	42	25.5	437	9.1	0.8	139	4.1	103	9.2	5	72	12.3	12.5	0.7	1.2

81	M	162.6	71.9	27.19	24.4	101	134	78	96.67	95	27.6	7.6	4.3	1.3	1.1	27	24	48	158	214	99	74	120.2	4.9	59.4	458	14.7	44.1	13.8	247	18.5	0.6	143	4	103	9.3	7.8	48	9.3	8.9	0.6	1
52	M	172.1	73.7	24.88	19.8	86	138	87	104	58	6.1	6.6	4.3	1.9	0.7	20	14	20	169	174	50	55	109	5.8	66	446	15.4	45.2	22.4	353	13.3	0.84	144	4.2	105	9.2	7	52	15.5	17.7	0.5	0.7
56	M	168.1	66.5	23.53	21.3	83	143	95	111	58	22.6	7.5	4.4	1.4	1.9	24	18	139	314	202	52	76	115.6	5.1	44.1	494	14.4	43.4	24.4	208	15.2	0.78	143	4	105	8.8	4.6	91	13.4	11.5	0.6	0.6
65	M	169.5	67.4	23.46	21.2	87	135	78	97	63	14.6	7.2	4.2	1.4	0.7	40	25	53	114	200	135	51	122	5.8	71	445	13.9	41.8	25.3	262	26.4	1.28	141	4.8	106	9.2	6.4	52	15.9	12.9	0.5	0.8
59	M	162.9	63.7	24	24.9	81.5	137	86	103	99	3.2	8.3	4.7	1.3	0.9	31	39	26	235	231	81	61	153.8	5.7	74.9	537	16.7	49	17.3	279	15.9	0.8	141	4.1	100	9.9	6.9	97	16.5	16.9	0.5	0.6
67	M	163.5	65.9	24.65	23.4	87	156	96	116	97	2	7.6	4.6	1.5	0.7	21	15	16	141	190	63	71	106.4	5.2	77.9	443	14.4	42.9	16.6	472	13.8	1.02	142	3.9	103	9.6	5.9	51	22	18.3	0.7	0.6
52	M	168.9	67.8	23.77	20.7	84	121	81	94.33	62	5.6	7.2	4	1.3	0.5	21	14	22	147	161	183	44	80.4	5.4	86.9	501	15.4	47.7	25.2	339	10.8	0.74	143	4.6	105	9.1	6.6	50	12.5	11.7	0.6	0.7
51	M	164	56.5	21.01	23.5	84	127	89	101.7	69	2	7.3	4.1	1.3	0.6	32	35	29	212	231	222	47	139.6	5.5	59.9	501	15.4	46.6	20.6	262	11	0.95	141	4.9	101	9.1	6.8	66	9.7	9.6	0.5	0.7
65	F	156.2	58.3	23.89	34.3	85	139	77	97.67	66	18.8	7.6	4.1	1.2	0.7	28	44	40	180	194	114	50	121.2	4.7	46.1	436	14.6	40.4	11.4	274	18.7	0.51	144	4.5	108	9.2	4.7	91	13.4	13.6	0.7	0.7
67	F	150.7	43.6	19.2	24.4	69.5	137	84	101.7	67	17.4	7	4.2	1.5	0.5	20	15	12	397	180	47	80	90.6	5.9	37.2	418	12.2	37.5	22	309	14.9	0.48	142	4.3	106	9	4.2	68	15.7	12	0.7	0.7
68	M	174.1	67.9	22.4	20.3	84	149	92	111	70	10	7	4.1	1.4	0.8	23	20	24	215	179	165	41	105	5.7	51.8	414	13.7	41.3	13	226	21.4	1.02	141	4.4	105	8.9	7.9	93	11.9	10.6	0.8	0.7
63	F	146.7	55.5	25.79	35.6	88	148	86	106.7	78	10.5	7.2	4.5	1.7	0.9	27	42	26	293	261	126	68	167.8	5.6	48.7	466	15.2	44.4	27.4	288	16.8	0.52	144	4.4	105	9.9	5	57	19.1	17.5	0.7	0.9
65	F	149.7	60.5	27	38.1	91	137	74	95	69	5.5	7.8	4.5	1.4	0.9	41	60	44	515	252	124	72	155.2	5.7	54.4	464	14.6	43.9	16	274	12	0.49	142	4.2	104	9.4	4.8	101	21.9	20.3	0.8	0.7
57	M	166.3	67.4	24.37	26.1	86	119	75	89.67	61	4.3	7.3	4.7	1.8	0.9	19	16	18	142	185	86	51	116.8	4.9	47.6	454	14.2	41.8	17.7	212	19.9	0.87	143	4.3	105	9.3	5.1	85	9.2	10	0.8	0.7
53	M	160.4	70.6	27.44	25.6	87	165	100	121.7	53	6.4	7.5	4.6	1.6	1	26	33	40	273	209	128	40	143.4	5	67.8	437	14.8	43	22.7	253	13.7	0.84	142	4.3	103	9	7.5	62	13.6	10.1	0.7	0.7
61	F	157	59.3	24.06	31	83	125	76	92.33	69	13.1	7.6	4.8	1.7	0.9	18	17	30	205	205	90	55	132	5.5	52	453	12.9	40.3	23.4	298	11.1	0.61	144	4	105	9.7	5.7	77	15.2	14.2	0.8	0.7
64	F	137.3	39.8	21.11	25.9	67.5	116	66	82.67	54	32.4	7.2	4.4	1.5	0.9	19	9	15	239	236	107	55	159.6	5.2	47.9	371	11.3	34.8	22	313	14.6	0.49	144	4.1	108	9.2	4	68	14.3	9.5	0.8	1.1
50	M	177.7	71.2	22.55	18.2	79	128	75	92.67	59	7.8	6.6	4.2	1.8	0.6	21	33	13	149	190	169	41	115.2	5.2	38.4	475	13.6	42.3	26.1	241	20.5	0.82	145	4	107	9	5.2	86	7.8	8.9	0.5	0.7
59	M	167.7	73	25.96	26.2	89.5	132	91	104.7	68	4.9	7.4	4.5	1.6	0.5	51	57	175	200	230	466	62	74.8	5.4	112.6	456	15.1	45.8	29	313	14.3	0.88	141	4.2	102	9.8	8.8	73	12.4	12.4	0.7	0.7
72	M	167.3	71.3	25.47	24.9	88.5	140	77	98	72	82.4	7.4	4.7	1.7	0.8	23	20	24	195	169	161	44	92.8	5.2	66.7	469	15.4	45.4	22.1	341	12.1	0.87	143	4.3	107	9.4	6.8	78	15.7	15	0.8	1.3
77	F	144.4	61.5	29.49	35.4	94	119	66	83.67	57	20.7	6.9	4.4	1.8	0.5	21	17	17	183	195	102	48	126.6	5.7	47.4	375	11	33.4	22.5	204	22.8	0.55	141	4.4	104	9.3	5.1	60	7.1	7.1	0.9	0.9
83	F	148.7	71.4	32.29	33.8	100	128	66	86.67	70	16	7.6	4.3	1.3	0.7	23	17	23	212	237	120	66	147	5.6	54.3	379	11.3	33.8	23.6	348	12.4	0.57	144	3.7	102	8.9	5.8	75	12	9	0.8	0.7
86	F	131.3	35.1	20.36	23.1	64.5	162	80	107.3	86	29.2	7.5	4.2	1.3	0.4	36	14	16	239	190	33	60	123.4	5.5	64.3	305	10.9	30.3	26.9	361	15	0.56	137	4.3	102	9.5	6.9	115	9	12.1	0.9	0.8
63	F	158.2	56.5	22.58	29.9	76	141	88	105.7	70	31.5	7.5	4.5	1.5	0.7	22	20	20	334	199	60	62	125	5.3	47.4	505	14.4	44.2	22.8	308	15.4	0.49	143	4.2	105	9.4	4.3	88	13.8	12.7	0.9	0.7
63	M	168.5	74.2	26.13	27.3	86	123	75	91	72	4.3	7.3	4.1	1.3	0.4	47	36	37	192	185	120	68	93	5.4	55.5	481	14.8	46.1	18	318	21.2	0.88	141	4.6	102	9.2	6.5	33	8.7	8	0.7	0.7
60	F	160.7	48.1	18.63	26	76.6	121	62	81.67	50	49.3	7.8	4.4	1.3	1	44	36	30	396	195	150	54	111	5.5	58.1	419	13.6	40.4	16.8	298	15.4	0.65	142	4.4	102	9.6	4	78	12.2	14.2	0.8	0.7
81	M	154.9	56.9	23.71	16.1	74.5	109	55	73	60	162	7.6	4.2	1.2	0.7	35	16	63	146	173	41	89	75.8	5.4	57	394	12.3	37.5	23.2	272	18.6	1.1	140	4.6	104	9.3	7.4	145	7.4	7	0.7	0.7
60	F	154.9	52.4	21.84	29.8	75	127	70	89	69	2.8	7.3	4.4	1.5	0.6	17	22	18	308	254	312	46	145.6	5.5	60.3	503	15.2	44.6	30.7	354	12.1	0.58	142	4.4	104	10.1	5	90	13.9	16.2	0.7	0.7
64	F	161.3	51.8	19.91	26.4	71.5	108	54	72	63	31.3	7.8	4.3	1.2	0.8	20	11	26	219	266	118	73	169.4	5.3	66.1	416	12.5	39.3	21.1	298	12	0.62	141	4.3	103	9.3	4.8	108	8.7	8.9	0.7	0.7
53	M	176.9	68.6	21.92	20.1	80	130	80	96.67	72	2	6.7	4.3	1.8	0.4	18	18	31	277	265	228	43	176.4	5.6	139.9	513	16.3	49.2	31.4	452	11.8	0.79	143	4.5	103	9.5	6.3	79	13.8	12	0.6	0.6
59	M	170.4	67.4	23.21	19.7	80.5	152	90	110.7	45	5.9	7.2	4.4	1.6	0.8	19	19	26	176	171	88	46	107.4	5.2	65.8	478	15.4	45.9	20.8	261	14.1	0.97	143	4.3	104	8.9	7	74	17.4	15.2	0.8	0.7
57	M	165.9	64.4	23.4	21.7	79.5	143	83	103	67	10.4	7.6	4.7	1.6	0.8	22	17	38	248	212	66	70	128.8	5.6	47.3	504	16.3	48.5	22.7	293	15.7	0.96	144	4.8	104	9.5	5.1	56	15	13	0.7	0.8
66	M	174.1	80.6	26.59	25.4	92.5	122	77	92	46	5.2	7.3	4.3	1.4	0.8	25	23	19	207	200	61	50	137.8	5.8	64.2	456	15.1	44.5	25.5	361	13.4	1.15	144	4.1	105	9.3	8.1	89	11.4	12.1	0.7	0.8
64	F	158.9	58	22.97	29.9	83.5	195	113	140.3	82	5.6	8.1	4.9	1.5	0.8	18	20	24	315	308	158	68	208.4	5.7	45.5	491	15.1	44.1	26.8	313	11.8	0.51	144	3.4	103	9.6	3.6	67	9.6	8.3	1.1	1.3
64	M	170.5	74.4	25.59	22.8	95	157	87	110.3	52	22.9	7.4	4.3	1.4	0.9	20	13	41	220	222	64	63	146.2	5	60.2	484	15.5	45.4	23.9	354	11.6	1.11	142	4.3	103	9.4	6.1	86	10.7	11.1	1.2	1.1
67	F	156.1	69.9	28.69	40.8	92.5	135	72	93	70	14.3	7.5	4.3	1.3	0.7	25	20	19	180	222	159	57	133.2	4.9	48.1	456	13.5	40.7	21.4	318	10.9	0.69	143	4.3	104	9.3	6.1	163	16.8	16.4	0.6	0.5
67	M	164.9	61.7	22.69	23.6	77.5	130	80	96.67	52	24.1	7	4.1	1.4	0.7	20	21	79	221	207	82	66	124.6	5.5	67.6	439	14.3	42.5	17	239	11.8	0.66	140	4.6	104	9.1	6.5	94	16.5	16.7	0.6	0.8
64	F	147.9	44	20.11	26.4	71.5	128	79	95.33	78	5.1	7.5	4.5	1.5	1.5	21	16	23	204	245	67	86	145.6	5.1	49.6	453	14.1	41.6	24.2	329	14.7	0.6	142	4.4	104	9.5	4.6	72	12.4	12.1	0.5	0.7
80	M	161.6	54.1	20.72	16.6	72	147	88	107.7	66	314	7.7	4.1	1.1	0.8	29	19	24	195	216	75	92	109	5.5	75.6	444	14.4	44.3	17.6	318	14.6	0.77	142	5	101	9.7	5.4	143	12.4	14.5	0.9	0.9
63	F	150.4	55.3	24.45	36.5	84.5	151	87	108.3	63	16.3	7.5	4.5	1.5	0.8	23	21	57	362	221	109	55	144.2	5.4	45	468	13.9	41.7	19.3	298	18	0.53	144	4.1	108	9.5	4.5	105	14.3	13.4	0.7	0.7
56	F	155.8	64.1	26.41	36.7	84.5	139	73	95	51	19.8	7.2	4.4	1.5	1.1	19	15	24	209	198	86	64	116.8	4.9	47.5	458	13.7	40.5	18.1	250	16.5	0.67	143	4.4	104	9.6	4	59	15.1	14.3	0.8	0.9
65	F	157.3	47.9	19.36	21.7	67.5	141	71	94.33	86	8.3	7.4	4.4	1.5	1.2	17	12	13	144	171	50	65	96	3.8	46.9	410	13.3	35.6	19.5	226	13.2	0.66	145	3.6	106	8.9	4.3	126	12.6	13.3	0.8	0.8
57	M	165.6	84	30.63	28.9	100	128	68	88	52	28.2	7.2	4.3	1.5	1	44	85	51	211	236	304	42	133.2	5.1	48.6	484	15.3	46.3	17.1	272	11.8	0.89	144	3.9	105	9	5.2	68	13.8	17.8	0.7	0.8
65	M	150.8	56.9	25.02	25.9	81	152	95	114	68	48.7	7.2	4.5	1.7	0.7	22	13	25	122	209	202	50	118.6	4.8	68	525	15.9	46.6	22.8	184	11.1	0.97	142	4.3	105	9.6	5.9	104	13	11.2	0.7	0.7
61	F	151.7	50.4	21.9	30.4	77.5	173	95	121	75	17.1	7.5	4.6	1.6	0.6	15	12	41	265	209	184	45	127.2	5.1	63.4	451	13.1	39.8	26.5	219	10.7	0.56	144	4.7	106	9.7	4	82	19	16.3	0.7	1.2
68	M	166.4	62.5	22.57	21.1	83	147	75	99	52	5.8	7.3	4.3	1.5	0.5	21	14	32	244	216	127	64	126.6	5.9	73.1	540	16	48.5	18	285	12.6	0.88	143	4.8	104	9.4	5.2	82	13.8	11.1	1.2	2.8
51	M	171.3	71.9	24.5	24.7	89.5	159	100	119.7	72	4.7	7.1	4.3	1.5	0.6	50	70	432	368	222	737	56	18.6	7.8	60.9	501	16	47.2	52.6	167	13.4	0.59	139	4.7	100	9.3	5.7	44	18.2	16.7	0.5	0.7
59	M	173.6	68.7	22.8	22.4	81.5	153	98	116.3	72	3.3	7	4.4	1.7	1.6	27	24	43	165	193	264	56	84.2	5.5	51.1	508	15.7	45.8	15.8	106	12.2	1.09	142	4	104	8.9	6.6	108	12.9	12.3	0.7	0.8
55	M	162.1	56	21.31	16.6	75.5	117	73	87.67	58	7.7	6.8	4.2	1.6	1.1	23	21	32	127	230	93	67	144.4	5.1	62.3	452	14.2	41.5	21.5	171	14.8	0.97	143	3.8	105	9.3	6.2	108	11.2	10.1	0.7	0.7
56	F	150.5	44.3	19.56	25.6	66.5	107	58	74.33	71	10.3	7.5	4.2	1.3	0.6	19	12	19	228	180	58	64	104.4	5.5	55.8	444	12.9	39.2	23.5	318	12.8	0.63	141	4	104	9.1	4.5	147	9.8	12.1	0.7	0.6
60	M	163.8	63.4	23.63	26	80	153	86	108.3	63	4.8	7	4.4	1.7	0.5	20	13	48	279	188	170	38	116	6.4	89	443	14.5	42.6	17.5	289	11.8	0.8	143	4	104	9.3	6.2	69	11.4	12.5	0.6	0.8
61	M	168.3	66.4	23.44	21.7	84	127	76	93	70	21.3	7.3	4.3	1.4	0.9	19	21	56	206	161	84	60	84.2	5.8	50.4	467	14.8	44	21.5	261	13.2	0.66	139	4.1	103	9.2	3.6	85	18.1	15.5	0.6	0.6
63	F	161.6	51.2	19.61	24.9	73	106	73	84	66	16.1	7.1	4.3	1.5	0.7	17	13	33	219	215	73	77	123.4	5.5	51	437	13.4	40.1	17.7	298	12.7	0.53	140	3.9	100	9.5	4.9	86	9	8.5	0.5	0.6
60	F	159.7	60.1	23.56	28.7	85.5	134	78	96.67	68	13.8	7.9	4.6	1.4	0.9	18	13	20	254	236	214	50	143.2	5.4	56.7	470	14.4	41.7	21.6	272	19.5	0.61	143	4.6	103	9.5	3.4	73	11.9	11.7	0.8	0.8
54	M	171.8	67.8	22.97	25.1	83	164	102	122.7	67	2	7.7	4.7	1.6	0.8	28	38	141	181	195	140	50	117	5.1	83.1	480	15	44.7	24.8	298	17.9	0.67	141	4.3	101	9.1	6.5	55	15	15.9	1.1	0.7
68	M	170.5	76.5	26.32	22.7	90	144	91	108.7	51	16	7.2	4.4	1.6	0.8	33	37	34	214	160	103	63	76.4	5.2	54.7	514	15.1	45.2	15.6	241	13.1	0.96	143	4.2	105	9.1	6	96	10.4	11.6	0.8	1.1
49	F	158.5	56.2	22.37	28.6	74.5	111	62	78.33	64	4.3	7.5	4.5	1.5	0.9	15	6	8	152	169	71	64	90.8	4.7	54.7	437	13	39.5	23.2	285	12	0.54	141	4	103	9.4	3.8	57	15.6	12.3	0.8	0.7
67	M	162.7	79.2	29.92	29.3	98.5	173	96	121.7	61	11.3	7.3	4.4	1.5	0.6	27	34	68	200	211	120	46	141	5.7	63.3	505	15.9	46.5	23	233	14.6	0.67	141	4.3	106	9.3	8.2	59	14.8	16.3	0.8	0.8
59	M	162.5	71.2	26.96	28.8	88	132	78	96	71	3.5	7.1	4.2	1.4	1.1	24	29	40	266	255	201	51	163.8	5.7	96.2	530	16.4	49.1	20.1	335	22.6	1	145	4.3	107	9.6	7.6	78	10.2	9.2	0.6	0.8

77	M	160.1	58	22.63	18.1	72.5	136	71	92.67	54	59	7.2	4.3	1.5	1	20	17	26	176	220	103	90	109.4	5.3	47.2	475	15.1	45	16.6	231	14.9	0.98	140	4.4	102	8.9	7.2	89	12.7	11.5	0.7	0.6
63	M	167.8	53.5	19	14.4	70.5	145	83	103.7	66	23.4	7.5	4.5	1.5	0.8	20	30	16	245	166	68	71	81.4	5.8	74.5	446	14.3	42.5	17.3	272	21.2	0.81	141	4.1	103	8.9	3.6	57	9	9.8	0.5	0.6
59	M	170.1	77.1	26.65	25	91	149	84	105.7	60	3.6	7.8	4.4	1.3	0.5	16	20	44	215	260	327	53	141.6	6.6	85.7	490	13.9	42.7	26.2	340	15.5	0.69	141	4.7	103	9.8	8.7	42	9.8	11.2	0.8	0.9
50	M	177.3	89.6	28.5	27.8	94	113	70	84.33	60	2	6.9	4.3	1.7	1.2	18	32	88	190	213	381	40	96.8	4.5	50.7	468	14.7	41.6	22.6	244	14	0.78	143	3.8	105	9.1	5.9	38	13.7	13.3	0.7	0.8
52	M	162.9	71.6	26.98	30.7	90	165	109	127.7	64	4	7.2	4.4	1.5	0.8	14	19	32	215	216	112	58	135.6	5.3	58.3	536	15.5	46.2	24.8	257	13.5	0.95	142	4	106	9.2	6.3	101	10.8	11.6	0.7	0.9
58	M	161.4	60	23.03	17.4	75	129	79	95.67	53	9.9	6.7	4.1	1.6	0.7	16	13	23	217	192	111	52	117.8	6.4	64.3	485	15.9	47.5	23.3	282	11.3	0.7	144	4.2	105	8.9	5.6	57	10.1	12.5	0.9	0.8
57	F	157.9	52.7	21.14	25.6	79.5	106	55	72	59	6.6	7.2	4.4	1.6	0.7	24	25	15	146	221	88	77	126.4	5.3	37.9	437	13.4	40.3	14.4	300	15.5	0.6	141	4.3	106	9.5	4.1	113	8.9	7.8	0.5	0.7
57	M	167.7	69.4	24.68	25.6	82	138	87	104	66	4.5	7.5	4.6	1.6	1.1	22	17	22	154	205	74	74	116.2	5	50.6	453	14.5	43.8	21	286	15.9	0.8	142	4.1	105	9.6	7.1	61	16.8	16.2	0.7	0.7
55	F	156.5	52.8	21.56	28.5	75.5	159	98	118.3	62	22.1	8	4.6	1.4	1.3	16	14	23	259	247	105	59	167	5.7	62.8	510	14.8	43.7	27.5	291	8.4	0.51	143	3.9	103	9.7	5.2	70	16.7	18.5	0.8	0.7
68	M	160.9	62	23.95	19.3	82	133	79	97	63	10.9	7.1	4.3	1.5	0.9	17	18	25	205	183	64	56	114.2	5	61.9	505	15.4	47.3	22.3	218	17	0.9	143	4.3	106	9	3.8	73	9.3	10	0.8	1.1
68	F	150.9	45.9	20.16	20	64	156	93	114	71	99.7	7.4	4.6	1.6	0.9	32	22	18	384	208	61	68	127.8	6.2	46.2	445	12.2	38	20.5	257	13	0.7	143	3.6	103	9.4	6.1	73	13.2	12.7	0.9	1.1
57	F	153.9	46.7	19.72	23.6	72	113	61	78.33	76	8.1	7.7	4.6	1.5	0.7	23	15	13	145	190	75	64	111	4.9	51.7	413	12.5	38.2	20.7	347	20.7	0.52	143	4.5	107	9.5	3.8	68	15.3	13.8	0.9	1.1
63	F	150.6	48.9	21.56	29.7	79	129	87	101	69	20.5	6.9	4.4	1.8	0.7	20	15	23	199	258	162	96	129.6	5.4	54	464	13.1	40.9	23.2	282	12.6	0.65	141	4.1	105	9	4.3	106	10.6	11.1	0.7	0.8
59	M	156.7	69.3	28.22	28	90.5	177	97	123.7	72	24.2	7.4	4.4	1.5	1.2	114	90	60	215	180	100	52	108	5.7	76.5	512	15.3	45.5	22.2	296	14.5	0.87	142	4.4	105	9.3	7.1	62	15.1	14.2	2.8	0.8
54	F	149.3	51.5	23.1	28.7	77.5	100	57	71.33	69	17	7.5	4.6	1.6	1	26	18	20	219	252	93	68	165.4	5	49.6	442	13.6	41.2	20.8	184	12.3	0.74	144	4	104	9.7	5.4	128	14.1	15.3	0.8	0.5
55	M	166.7	70.3	25.3	18	83	142	85	104	55	3.9	7.6	4.8	1.7	1	26	34	23	153	207	60	56	139	5.4	73.1	447	14.6	43	25.9	247	19.5	0.96	142	4.2	103	9.7	6.3	129	10.8	10.6	0.8	0.9
52	F	157.2	42.8	17.32	18	58.5	101	65	77	67	5.4	7.3	4.3	1.4	0.8	19	14	14	185	212	53	82	119.4	4.9	53.2	458	12.6	39.1	20	257	11	0.68	144	4.5	107	9.5	3.6	57	14.1	13.8	0.5	0.5
57	M	170.7	67.5	23.17	22.5	84.5	132	83	99.33	61	5.9	7.2	4.5	1.7	1.1	31	34	80	254	218	188	53	127.4	5.6	68.8	549	16.2	49.1	21.1	238	16.9	1.01	144	4.7	106	9.5	8.5	63	12	11.2	0.5	0.6
53	M	165.1	81.6	29.94	25.2	89.5	132	82	98.67	63	2.7	7.8	4.5	1.4	1	51	86	205	187	204	200	56	108	5.1	60.7	472	15.1	44.1	23.3	233	13.6	0.97	141	3.8	104	9.3	6.1	43	13.4	12.5	0.7	0.8
58	M	165.9	78.7	28.59	24.7	91	141	89	106.3	61	12.9	7.6	4.6	1.5	0.4	51	62	164	144	186	219	43	99.2	5.1	40.3	505	15.8	47	15.3	265	17.1	0.78	141	4.1	103	9.3	4.4	69	13.6	14.1	0.6	0.6
70	M	164.5	63.5	23.47	22.3	80	162	71	101.3	58	26	7.5	4.1	1.2	1	22	15	26	208	163	55	67	85	5.2	46.9	450	13.9	42.3	17.5	233	12.6	0.83	140	4.9	104	8.7	5.2	59	7.8	7.9	0.6	0.7
47	M	180.5	73.1	22.44	25.6	84.5	132	90	104	74	2	8.2	4.8	1.4	1.3	40	57	160	181	218	228	55	117.4	4.8	62	557	15.5	46.9	24.2	197	16.8	0.8	142	4	104	9.7	6.8	46	10.7	13.8	0.7	0.5
67	M	167.9	67.9	24.09	20.9	87	128	82	97.33	56	7.9	7.1	4.3	1.5	1.5	31	31	47	172	178	92	46	113.6	5.3	64	479	16	47	23.7	250	15.1	0.81	140	4.2	105	9.2	6.8	60	16.7	14.5	0.8	0.8
68	F	155.2	53.9	22.38	28.7	77	183	112	135.7	67	32.1	7.5	4.6	1.6	0.8	26	40	96	235	199	124	74	100.2	5.1	46.1	424	13	39.6	17	328	15.6	0.76	143	4.2	104	9.3	5.8	60	13	11.3	1.1	1.1
60	F	160.1	45.7	17.83	18.1	66	139	79	99	65	25.6	7	4.3	1.6	1.3	23	13	13	279	235	84	92	126.2	5	49.3	437	13	40.7	20.5	269	15	0.8	142	3.9	100	9.6	5.3	128	10.2	13	0.7	0.5
65	F	147.1	47.4	21.91	29.7	77.5	133	68	89.67	69	3.3	7.8	4.7	1.5	0.7	22	17	12	212	205	78	89	100.4	5	32.3	359	11.6	35.6	17.8	291	15.9	0.66	143	4.3	105	9.7	3.5	102	10.5	11.2	0.8	0.9
65	M	163.3	65.8	24.67	24.4	89	139	92	107.7	80	2	7.2	3.9	1.2	0.8	19	16	27	197	192	142	42	121.6	5.3	97.6	437	14	42.4	35	322	16.9	0.79	140	4.8	104	9.1	7.6	123	10.4	10.9	0.8	0.9
66	M	164.1	67	24.88	24.2	84.5	145	91	109	73	96.7	7.5	4.5	1.5	0.9	20	23	27	242	200	133	58	115.4	5.1	52.6	515	16.6	48.3	17.3	311	17.3	0.92	141	4.1	100	9.5	5.9	61	7.5	7.5	1.1	0.9
65	F	150.9	50.8	22.31	29.7	80	123	69	87	57	21.9	7.1	4.5	1.7	0.7	23	18	13	152	228	57	74	142.6	5.2	35	433	13.5	40.2	20.7	261	14.2	0.63	144	4.1	105	9.1	5.2	80	10.8	11.3	0.7	0.9
60	M	167.7	63	22.4	25.2	76.5	109	66	80.33	56	8.3	6.9	4.3	1.7	0.7	23	15	16	229	181	58	68	101.4	7.7	46.8	422	12.6	39.3	21.5	250	14	0.76	141	4.5	102	9.6	5.1	47	11.6	11.4	0.8	0.8
66	F	151.3	52	22.72	27.5	73	147	80	102.3	65	10	7.4	4.5	1.6	0.8	15	9	13	327	208	50	64	134	5.4	44.2	401	12	37	23.2	300	13.7	0.5	142	4.1	106	9	3.9	52	15	13.2	0.6	0.7
58	M	169.9	62.7	21.72	14.6	74	111	69	83	59	7.5	7	4.5	1.8	0.8	17	15	22	172	185	79	78	91.2	6.2	54.3	477	14.5	42.3	18.8	286	14.4	0.88	143	4	104	9.2	5.6	85	11.9	17.4	0.7	0.7
54	F	157.4	72.2	29.14	41.3	93.5	116	76	89.33	61	28.5	6.6	4.2	1.8	0.5	18	19	18	193	206	50	56	140	5.7	61	493	14.5	43.5	23.1	375	22.1	0.72	144	4.3	106	9.5	5.6	51	19	16.4	0.5	0.5
65	F	149.6	46.4	20.73	25.5	69.5	95	46	62.33	59	17.2	7.4	4.3	1.4	0.7	29	35	35	221	216	77	82	118.6	5.1	43.1	407	12.6	38.8	20	254	14.5	0.65	141	4.3	104	9.3	5	90	11.6	10.7	0.7	0.7
63	M	160.4	51.6	20.06	17.2	76	121	77	91.67	82	2.8	7.5	4.5	1.5	0.9	27	36	40	166	247	143	69	149.4	5.3	87.5	479	14.5	43.7	22	213	11.3	0.84	142	4.3	102	9.3	6	103	15.5	17.7	0.7	0.7
55	F	154.1	54.5	22.95	29.2	72.5	106	65	78.67	64	24.2	7.2	4.1	1.3	0.8	20	18	16	289	160	71	61	84.8	5.1	43.4	450	13.4	39.6	22.4	209	12	0.49	143	4	103	9.1	4.3	99	16.5	16.5	0.7	0.8
51	M	160.9	56.1	21.67	20.2	77.5	110	58	75.33	70	9.5	6.7	4.4	1.9	0.8	22	23	41	269	204	107	61	121.6	5.1	66.2	468	15.8	47.6	26.9	215	14.3	0.88	144	4.3	106	9.1	5.9	68	10.6	11.8	0.5	0.5
61	F	152.9	59.7	25.54	34.7	87.5	123	62	82.33	69	20.4	7.3	4.3	1.4	0.9	18	17	19	257	275	140	54	193	6	62.6	511	14.3	43.7	23.2	375	11.8	0.7	144	4	104	9.5	6.6	122	9.7	11	0.5	0.6
57	M	168.5	82	28.88	29.3	99	131	78	95.67	48	14.3	7.5	4.7	1.7	2.5	32	35	21	193	176	117	56	96.6	5.2	61	517	16.5	48.9	16.1	261	17.4	0.85	142	4.6	101	9.7	7.6	129	14.8	16.4	0.8	0.7
61	F	153.1	57.9	24.7	35.1	89.5	170	89	116	63	5.7	7.3	4.6	1.7	0.7	24	13	98	222	253	91	68	166.8	5.6	62.4	482	14.7	44.8	23.6	306	12.4	0.55	143	4	104	9.7	4.7	24	14	12.5	0.8	0.8
62	F	166.1	68.8	24.94	32.1	88.5	125	82	96.33	74	19.3	8.1	4.4	1.2	0.9	37	64	97	280	217	153	50	136.4	5.6	51.6	415	13.1	41	15.3	311	15.4	0.68	141	3.8	105	9.6	5.7	65	12.5	11.3	1.3	0.8
44	F	161.3	48.5	18.64	21.5	68.5	103	55	71	70	12.5	7.3	4.6	1.7	0.5	19	15	12	172	180	48	86	84.4	4.6	41.7	470	13.8	42.8	15.7	265	11.5	0.8	140	4	104	9.2	3.8	174	11.5	12.5	0.5	0.7
65	F	153.3	50	21.28	25.7	83	131	71	91	73	12.7	7.1	4.6	1.8	0.7	19	15	18	288	218	48	99	109.4	5.1	49.4	419	12.5	39.4	21.1	225	15.7	0.67	143	4.4	106	9.5	4.9	75	14.6	14.5	0.7	0.7
69	M	156.1	57.4	23.56	18.4	72	158	87	110.7	53	18.3	7.3	4.4	1.5	0.6	17	9	21	218	170	74	58	97.2	4.6	49.7	405	13.4	40.9	20	334	14.2	0.96	143	4.6	104	9.5	5.3	72	9.8	12.9	0.9	1.1
58	F	159	67	26.5	34.5	88	140	77	98	54	13.6	7.8	4.5	1.4	0.8	16	18	20	280	215	234	54	114.2	6.2	48.6	464	14.1	41.8	25.2	247	8.3	0.63	141	4.2	101	10	5.4	70	15.3	13	0.8	1.1
62	M	163	61.3	23.07	22.5	85.5	126	78	94	72	9.2	8.1	4.5	1.3	0.8	25	32	67	409	189	109	37	130.2	5.3	59.6	522	15.6	47	18.1	254	13.6	0.88	141	4.9	100	9.5	6.9	80	12.5	11.3	0.5	0.8
63	M	161	54.2	20.91	17.3	76.5	121	78	92.33	61	11.4	7.5	4.5	1.5	0.9	24	21	22	211	196	54	65	120.2	5	67.3	484	14.8	44.9	19.7	296	20.2	0.98	143	3.8	104	9	5.7	96	13.5	12	0.6	0.6
67	F	153	61	26.06	34	84.5	133	77	95.67	58	26.6	7.2	4.4	1.6	0.9	35	49	29	265	192	90	66	108	5.3	53.2	441	13.3	40.1	16.4	296	15.3	0.69	142	4.1	105	9.1	6.1	75	12.8	12.9	0.6	0.6
56	M	165.7	71	25.86	22.3	87	119	82	94.33	50	10.4	7	4.3	1.6	0.6	27	26	50	142	212	99	52	140.2	5.1	51.6	470	15.3	45	21.8	296	12.9	0.93	142	4.4	106	9.6	6.6	74	10.2	9.3	0.7	0.5
51	M	171.2	54.9	18.73	17.4	74	121	75	90.33	51	4.2	7.3	4.5	1.6	0.7	18	15	28	197	239	90	62	159	5.1	45.8	477	14.6	44.6	16.4	328	11.3	0.79	145	4.7	105	9.6	5.7	61	9.9	7.7	0.8	0.7
60	F	158.1	53.9	21.56	27.9	77	130	81	97.33	58	7.8	7.9	4.6	1.4	0.8	18	9	11	296	257	106	61	174.8	5.1	52.9	439	13.1	39.8	24.5	265	10	0.69	145	4.9	108	10.7	4.5	42	12.2	10.8	0.8	0.9
55	M	174.5	72.6	23.84	24.8	88.5	141	90	107	68	6.4	7	4.6	1.9	0.9	24	27	110	237	239	124	58	156.2	5.1	78.6	535	16.7	49.2	34.5	273	13.8	0.71	142	4.5	105	9.6	5.9	54	12.1	12.1	0.8	0.8
60	M	162.9	70.7	26.64	25.9	91	134	84	100.7	78	9.2	8.1	4.9	1.5	1.1	28	36	114	222	218	80	58	144	6.6	54.7	610	18.8	55.8	16.3	247	12.7	0.76	143	3.8	103	9.6	5.5	69	13.2	15.3	0.8	0.8
67	M	172.1	73.8	24.92	24.5	88	136	89	104.7	58	10.6	7.6	4.5	1.5	1.2	28	23	23	267	201	51	55	135.8	4.9	56.4	421	14.1	41.9	15.3	286	15.9	0.78	142	4	104	9.3	6.4	29	9.5	8.5	1.1	0.8
60	F	156.9	47.9	19.46	22.1	68	117	67	83.67	70	20	7.1	4.6	1.8	0.7	31	24	13	124	204	68	76	114.4	5.4	38.2	460	14.2	41.6	24.9	282	11.8	0.47	137	4.4	97	9.8	2.3	84	15.7	14.1	0.5	0.5
64	M	162	64.3	24.5	19.9	83.5	137	95	109	61	30.8	7	4.5	1.8	1	19	19	47	258	193	149	38	125.2	4.9	57.4	526	16.2	46.9	21	261	16.4	0.8	144	3.8	105	9.4	7	93	11.4	13.4	0.7	0.8
42	M	165.7	55.8	20.32	16.6	68	114	74	87.33	55	7.3	7.7	4.7	1.6	0.8	20	14	15	321	219	77	57	146.6	5.3	61.4	482	13.3	40.6	27.7	235	12.2	0.69	142	4	102	9.9	4.4	80	8.7	7.9	0.5	0.7
68	F	149.5	40.1	17.94	17.7	62	136	81	99.33	63	46.1	6.9	4.4	1.8	0.7	35	25	17	309	223	58	113	98.4	4.7	27.7	420	10.6	33.8	22.2	254	12.8	0.57	142	4	105	9.2	2.9	114	8.2	7.9	0.7	0.7
60	M	166.1	58.2	21.1	21.1	78	127	79	95	66	24	7.4	4.5	1.6	1.9	24	21	34	281	170	71	52	103.8	5	54.6	521	15.9	47.9	25.9	273	15.8	0.89	141	4	101	9	5	73	14.1	11.7	0.5	0.7

67	M	167.4	68.2	24.34	21.2	75.5	127	82	97	73	4.8	7.8	4.4	1.3	0.9	24	31	33	249	213	172	48	130.6	5.2	76.1	483	15	45.2	22	265	14.2	0.8	141	3.8	100	9.6	4.9	86	9.3	10.1	0.8	0.8
64	F	154.5	54.9	23	31.1	84	119	68	85	69	15.1	7.6	4.6	1.5	0.9	28	31	103	220	210	141	59	122.8	5.2	49.5	421	13	37.4	18	198	14.3	0.71	142	4.4	106	9.6	5.2	38	14.5	18.2	0.7	0.7
67	F	145.3	53.1	25.15	31.5	84.5	144	81	102	59	30.7	7.2	4.1	1.3	0.5	25	23	26	262	214	98	53	141.4	5.1	32.9	411	12.3	37.6	18.1	247	9.9	0.59	143	4	106	9.2	5.1	73	10	11.4	0.7	0.8
61	F	151.2	56.8	24.85	30.8	80	136	85	102	71	6.6	7.3	4.5	1.6	0.7	38	31	20	210	229	136	51	150.8	4.8	59.1	474	14.3	43.4	27.1	306	13.2	0.49	142	4.4	104	9.4	4.8	64	8.6	10.6	1.1	1
60	M	167.7	68	24.18	24.8	81	138	82	100.7	59	5.4	7.6	4.5	1.5	0.6	22	23	54	169	160	122	49	86.6	5	52.4	462	15.1	44.1	20.8	213	13.6	0.75	141	4	104	9.2	7.6	64	10.9	10	0.9	0.7
61	M	162.5	64.3	24.35	24.8	82	155	92	113	61	14	8.1	4.7	1.4	0.8	35	28	51	228	278	88	65	195.4	4.9	75.2	462	14.9	43.7	19.9	211	14.5	0.91	142	3.9	102	9.7	3.9	89	11.6	9	1.8	1.3
50	M	170.5	80.6	27.73	27	91.5	137	77	97	59	5.2	7.7	4.5	1.4	0.9	18	17	28	206	198	273	40	103.4	5.2	54.2	539	14.3	44.5	21	353	14	0.75	142	4.3	102	9.6	5.6	40	9.8	10.8	0.7	0.5
64	F	148.1	58.1	26.49	33	85.5	149	86	107	77	9.7	7.3	5	2.2	0.7	22	22	13	231	268	85	87	164	5.5	62.6	447	13.5	40.8	30.5	316	13.4	0.56	143	3.8	104	9.3	3.9	40	9	9.4	0.7	0.7
50	M	167.3	60.3	21.54	24.5	80	132	83	99.33	45	18.1	7	4.3	1.6	0.7	24	19	43	225	178	60	67	99	5.3	58.1	484	15.3	46	18.9	220	16.3	0.69	142	4.2	104	9.5	5.6	53	11.9	11.9	0.5	0.7
56	F	156.1	44	18.06	19.1	64	150	97	114.7	69	11.5	7.3	4.4	1.5	1.2	15	10	13	303	241	42	113	119.6	4.9	33	417	13.5	42.6	19.8	238	11.7	0.52	145	3.6	104	9.7	4.7	92	9.1	10.2	0.8	0.7
52	M	171.6	78	26.49	26	88	138	87	104	83	13.1	7.3	4.5	1.6	0.8	60	72	269	316	188	375	44	69	5.1	46.6	489	14.8	43.8	17.5	257	13.6	0.74	140	4.2	104	9.3	6.1	96	10.2	12.6	0.8	0.9
53	M	164.6	73	26.94	24.5	86	126	86	99.33	58	2	7	4.2	1.5	1.2	22	19	138	205	153	109	58	73.2	5	69	478	15.4	45.9	19.5	322	11.1	0.71	140	4.1	101	9.2	5.1	53	13.6	11.7	0.8	0.9
63	F	147.9	48	21.94	29	80	109	71	83.67	61	17	7.8	4.9	1.7	1	24	27	25	202	118	147	38	50.6	5.6	85.3	533	14.3	45.1	24.7	273	19.8	0.71	143	4.2	105	9.7	6.3	78	12.3	10.5	0.5	0.7
63	F	156.2	48	19.67	23.3	69	122	79	93.33	73	28.1	7.4	4.8	1.8	1.2	26	16	21	212	255	57	113	130.6	5.2	49.6	483	14.8	45.3	15.9	306	13.9	0.73	142	4.5	101	9.5	2.9	94	12	14.9	0.7	0.8
62	F	166.6	64.1	23.09	31.3	89	111	63	79	63	24.9	7.1	4.2	1.4	0.6	16	15	15	194	201	80	58	127	5.6	56.4	454	13.7	43.3	19.8	353	10.8	0.51	145	3.8	107	9.5	4.9	57	9.3	8.2	0.8	0.8
69	M	164.3	65.3	24.19	29.6	87.5	121	77	91.67	72	10.6	7.5	4.4	1.4	0.9	32	22	95	155	206	70	95	97	5.4	47.1	475	14.4	44.1	18.2	265	15	0.85	140	4.4	100	9.5	7.9	100	14.2	13.8	0.7	0.7
65	M	160.3	63.4	24.67	26.3	84	121	72	88.33	90	29.8	6.1	4.2	2.2	0.7	16	14	17	138	198	57	56	130.6	5.2	95.7	483	14.7	43.6	18.5	311	13.3	0.8	145	4.1	108	9.1	5.5	81	10.9	13.1	0.5	0.7
58	M	164.5	72	26.61	24.8	86	118	71	86.67	53	17	7.1	4.5	1.7	0.7	22	21	33	217	219	143	37	153.4	5.9	70.5	468	15.4	45.2	19	291	16.1	0.81	142	4.6	103	9.4	5	60	12.9	14.3	0.5	0.5
57	F	152.9	54.7	23.4	27.3	78	151	86	107.7	48	33.5	7.2	4.3	1.5	0.7	20	16	22	227	190	77	64	110.6	5.1	52.8	466	13.8	41.9	15.6	306	13.1	0.55	143	4.1	105	9.2	4.6	63	11.7	12.5	0.5	0.8
64	M	162.2	84	31.93	34	103.5	169	97	121	68	10.6	7.5	4.2	1.3	1	23	10	54	246	196	141	50	117.8	5.4	67.9	561	16.1	48.8	23.3	393	13.4	0.85	140	3.9	101	9.4	6.1	78	15.3	14.1	0.8	1
57	M	171.6	57.2	19.43	14.2	71	117	68	84.33	52	25.3	6.9	4.3	1.7	0.8	19	11	23	162	190	55	76	103	5	47.1	413	13.5	41.2	27.2	300	17.2	0.88	143	4.6	103	9.4	6.3	72	11.9	13.2	0.7	0.7
66	F	150.1	46	20.42	23.2	67	112	61	78	74	24.8	7.9	4.8	1.5	1	26	28	17	360	196	92	72	105.6	5.3	77.2	418	13.2	39.5	24.7	269	11.6	0.68	143	4.6	104	9.7	5.1	78	11.9	11.6	0.7	2.1
65	F	154	52.9	22.31	31.2	83.5	133	78	96.33	65	20.6	7.2	4.5	1.7	0.9	19	16	16	269	207	148	44	133.4	4.8	63.8	492	14.9	43.9	22.5	261	11.7	0.7	144	3.9	105	9.3	1	61	12.1	15.5	0.6	0.6
60	F	156.3	47.6	19.48	22.4	65.5	121	79	93	64	25.4	7.8	4.3	1.2	0.7	23	19	14	232	151	97	57	74.6	4.8	36.7	414	11.9	37.7	22.2	222	16.1	0.5	143	4.1	104	9.5	3.9	89	8.5	8.9	0.7	0.7
60	M	175.1	90.6	29.55	24.3	104.5	145	88	107	62	209	6.8	4.2	1.6	0.8	32	31	63	366	159	72	70	74.6	4.8	58	491	15.4	46.1	13.5	211	18.7	0.79	144	4.2	106	9	6.8	57	16.2	16.1	0.7	0.8
65	M	165.3	70	25.62	29.7	85.5	168	98	121.3	63	16.3	7.7	4.6	1.5	0.7	34	37	50	311	207	154	47	129.2	5.4	79.5	482	15.5	45.4	23.3	300	21.2	0.84	140	4.9	102	9.6	7.7	95	11.9	13.9	0.9	0.8
80	M	165.3	66.4	24.3	20.6	83.5	171	94	119.7	67	44.5	7.8	4.6	1.4	2.1	21	22	20	197	245	52	80	154.6	4.7	41.9	516	17.1	49.9	22.9	178	11.8	0.78	141	4.2	102	9.5	4.5	110	11.3	11.3	0.9	0.8
58	F	156.9	50.1	20.35	24.2	79.5	114	57	76	66	4.4	6.1	3.9	1.8	1.2	19	17	10	163	243	88	58	167.4	5.5	58.8	414	12.6	38.3	15	328	14.1	0.49	144	4.7	106	9.2	4.3	99	12.9	9.2	0.7	0.7
60	M	160.9	49.1	18.97	14.7	64.5	127	73	91	63	7.9	7.2	4.3	1.5	0.8	31	21	25	273	254	164	77	144.2	6.1	93.2	482	14.8	45.1	21.9	300	14	0.82	142	4.1	102	9.4	5	89	7.7	7.3	0.8	0.8
56	M	172.5	75.9	25.51	21.2	91	133	79	97	54	9.7	6.7	4.4	1.9	1	19	23	25	296	176	155	53	92	8.2	73.5	488	15.4	47.2	20.5	353	8.7	0.8	143	4.6	102	9.7	5	52	16.6	14.9	0.9	0.9
52	F	160	51.4	20.08	22.6	69.5	121	74	89.67	60	94.5	6.7	4.2	1.7	0.6	15	11	13	146	176	96	79	77.8	4.8	64.1	431	12.8	38.7	22.2	257	13.7	0.57	140	3.4	103	9.3	3.3	58	14.5	15.2	0.7	0.7
58	M	171	58.9	20.14	15.6	76	118	80	92.67	78	6.4	7	4.3	1.6	0.7	100	114	875	411	193	473	50	48.4	5.5	49.7	460	14.5	43.4	33.4	172	12.7	0.75	141	4.9	104	9.2	5.6	73	12.7	14.6	0.7	0.7
66	F	150.7	46.6	20.52	34.8	77	144	97	112.7	98	4.4	8.1	4.9	1.5	0.8	25	36	27	181	279	56	98	169.8	5	50.5	494	15.1	46.2	23.7	282	13.5	0.58	141	5.5	100	10.3	4.1	68	13.7	12	0.9	0.9
68	M	179.3	69.8	21.71	17.5	82	143	93	109.7	65	15.9	6.6	3.9	1.4	0.6	18	19	25	139	182	80	64	102	5.9	48.5	452	14.5	43.9	16.4	241	14.3	0.98	143	4.1	106	8.9	6.6	61	15.4	14.3	0.9	0.7
65	M	156.6	55.4	22.59	23.9	80	141	87	105	63	7.6	8.5	4.4	1.1	1.3	32	32	46	224	176	127	48	102.6	7.1	59.9	456	15.4	45.7	20.2	282	13.4	0.72	141	4.3	101	9.4	4.9	52	10.3	8.2	0.9	0.8
67	M	172.5	80.4	27.02	28.8	95	131	82	98.33	56	16.9	7.1	4.4	1.6	1	28	29	73	257	234	169	54	146.2	5.8	62.6	519	16.5	49.6	18.1	230	13.3	0.9	142	4.1	104	9.2	6.8	50	10.4	10.6	0.8	0.8
64	F	147.7	38.9	17.83	20.6	71.5	125	76	92.33	71	11.7	7.2	4.6	1.8	0.5	28	19	58	256	169	93	72	78.4	5.4	110.8	475	13.9	42.9	33.7	435	12.1	0.47	142	4.1	102	9.7	5.3	94	11.8	14.2	0.7	1.4
64	M	167.7	61.9	22.01	18	83	116	70	85.33	52	6.7	7.4	4.5	1.6	0.9	25	19	20	179	172	47	68	94.6	4.6	58.5	466	14.8	43.8	17.7	316	10.8	0.65	142	3.9	102	9.5	6.3	68	13.3	15.2	0.8	0.9
56	M	173.3	71.5	23.81	22.7	87	158	94	115.3	65	26	7.3	4.4	1.5	0.4	21	22	19	339	229	215	37	149	5.9	60	534	15.7	47.3	22.2	291	22.1	0.97	143	4.2	104	9.2	7.1	61	19	18.1	0.9	0.9
61	F	159.7	61.1	23.96	28.8	83.5	116	71	86	69	6.7	7.3	4.6	1.7	0.6	29	35	26	199	206	59	78	116.2	5.2	48.1	419	12.8	38.9	26	300	14.3	0.69	144	4.1	103	10.1	5.7	55	12.3	13.4	0.8	0.7
51	M	163.7	67.2	25.08	28.7	87	138	95	109.3	57	17	6.8	3.9	1.3	1	35	39	244	179	222	348	62	90.4	4.8	76.1	534	18.2	54.1	12.2	265	12.2	0.89	141	3.6	102	9	7.4	80	8.2	9.9	0.7	0.8
39	F	153.7	46	19.47	23.7	72	131	81	97.67	104	11	7.5	4.5	1.5	0.5	23	34	21	140	176	170	52	90	5.1	62.9	493	14.2	43.5	21.5	244	9.6	0.55	142	4.1	102	9.2	5.7	111	17.3	17.3	0.5	0.5
50	M	170.5	69.8	24.01	21	86.5	150	83	105.3	74	2	7.5	4.6	1.6	0.5	23	26	95	233	197	141	49	119.8	4.7	45	519	15.8	47	19.8	282	9.1	0.83	143	4.2	105	9.4	6.3	62	9.6	10.3	0.7	0.5
68	F	154.1	57.1	24.05	34.4	90.5	111	64	79.67	72	12.4	7	4.2	1.5	1	24	27	20	237	171	112	60	88.6	5.2	55.2	412	12.5	38.8	20.9	235	10.2	0.55	144	4.6	104	9.5	4	82	13.2	11.6	0.7	0.8
55	M	165.3	56.3	20.6	18.1	73.5	141	94	109.7	60	4.8	7.1	3.8	1.2	0.7	37	31	124	302	127	67	54	59.6	4.7	52.9	434	13.9	42.6	26.8	367	11.5	0.69	144	4	105	8.8	7.8	50	14.2	14.4	0.5	0.7
65	F	154.5	58	24.3	33.5	90.5	132	63	86	75	10.3	7	4.2	1.5	0.9	22	22	16	258	206	121	42	139.8	5.1	47.1	480	14.5	43.1	21.8	347	19	0.62	142	4	103	9.3	5.2	49	15.7	12.7	0.9	0.9
68	F	143.6	57.3	27.79	37.8	91.5	132	74	93.33	58	29.6	7.9	4.3	1.2	0.6	23	25	36	222	205	108	72	111.4	4.9	52.7	406	12.4	38.5	23.2	340	10.1	0.46	144	3.9	102	9.6	4.8	67	11.3	7.9	0.9	1.3
62	F	157.3	63	25.46	36.7	89	149	80	103	66	11.3	8	4.8	1.5	0.6	21	28	30	399	205	90	60	127	5.8	56.6	489	14.1	41.8	19.6	347	17.8	0.53	144	4.3	105	9.6	4.5	71	11.6	10.9	1.1	0.9
67	M	165.7	69.7	25.39	23.7	88.5	134	73	93.33	63	10.5	7.5	4.3	1.3	0.8	46	43	107	161	168	303	49	58.4	5.1	63.9	431	14	41.4	18.2	300	13.2	0.82	141	4	103	9.4	5.4	74	10.3	12	0.7	0.5
58	F	152.6	49.5	21.26	29.1	76.5	110	76	87.33	72	7	7.6	4.4	1.4	0.5	20	7	23	239	265	75	89	161	5.2	75.9	451	13.4	40.5	22.2	291	14.7	0.53	143	4.5	106	9.4	4.4	106	13.1	12.7	0.5	0.5
55	M	163.7	60.1	22.43	22.4	76.5	121	80	93.67	64	7.2	6.7	4	1.5	0.6	26	11	27	200	218	201	50	127.8	5.2	82	473	14.7	43.5	33.5	393	11.3	0.7	142	4.6	105	9.2	7.3	99	7.7	7.8	0.7	0.8
57	F	149.6	52.4	23.41	33	81	131	72	91.67	65	14.2	7.4	4.8	1.8	0.5	27	18	20	211	264	82	98	149.6	5	65.5	425	13.3	40.5	30.1	241	11.1	0.56	144	3.9	103	9.4	6	102	10.8	11	0.7	0.8
64	M	168.7	62.7	22.03	18.9	85	172	109	130	85	6.9	7.6	5	1.9	0.5	43	29	90	154	214	108	70	122.4	4.8	53.2	402	14	41.2	25.5	296	13.5	0.91	146	4.4	102	10.2	6.4	90	12.6	13.6	0.5	0.5
59	M	157.3	54.6	22.07	18.7	76.5	145	89	107.7	63	4.2	7.7	4.6	1.5	1.2	28	28	48	142	240	152	64	145.6	5	58.7	517	16	48.3	22.6	269	13.7	0.86	143	4.4	102	9.5	4.4	129	15.5	14.2	0.5	0.5
68	M	167.1	64.6	23.14	17.9	79	153	82	105.7	59	3.4	7.6	4.7	1.6	1	32	31	28	232	214	110	65	127	5.3	68.6	545	17	50.8	21.3	360	14.3	0.98	141	5.6	99	9.7	7.5	89	13.6	13.4	0.7	0.7
59	M	163.5	62.8	23.49	31	82.5	112	72	85.33	66	6	7.5	4.3	1.3	1.2	16	13	35	286	185	170	57	94	5.2	55.3	545	16.1	48.9	18.9	202	16	1.12	140	4	103	9.4	7	95	10.9	11.3	0.7	0.7
63	F	147.8	67.8	31.04	36.5	101.5	143	81	101.7	77	16.4	6.6	4.2	1.8	0.8	26	28	19	249	237	113	56	158.4	5.9	82.5	473	14.1	43.3	27	353	11.8	0.4	145	4.8	107	9.5	4.7	44	16.9	15.1	0.7	0.8
54	F	158.3	53	21.15	25.7	77	128	78	94.67	62	6.5	8	5	1.7	0.7	27	19	15	201	212	64	80	119.2	5.2	44.4	478	14.1	43.4	22.9	286	13.2	0.62	143	4.9	105	9.6	4.6	41	13.3	11.4	0.5	0.5
63	M	156.5	57.9	23.64	25.1	80	151	92	111.7	76	3.6	8.1	4.1	1	0.7	27	29	70	163	237	414	44	110.2	7.4	55.8	437	14.7	43.6	20.6	273	11.4	0.81	136	4.1	99	8.8	4.4	43	14.7	15.5	0.8	0.8

67	M	161.5	52.6	20.17	23	75	134	89	104	54	8.7	7.6	4.4	1.4	2	22	11	22	166	192	170	49	109	5	70.9	462	15.1	45.2	20.9	347	16.8	0.73	142	4.3	101	9	6.5	86	14.6	13.4	0.8	0.7
62	M	154.9	53.3	22.21	21.5	79	137	78	97.67	73	3.5	8	4.6	1.4	0.5	21	24	198	299	165	240	48	69	7.6	83.2	397	12.9	39.1	23.7	411	23.4	1.21	138	5	101	10.7	5.2	102	15.3	14.4	0.6	0.7
52	F	151.3	52.1	22.76	26.2	70.5	125	67	86.33	61	7.9	7.1	4.3	1.5	0.8	20	25	28	241	195	70	67	114	5	42.1	507	14.6	44.1	17.5	227	16.9	0.67	143	4.2	102	9.2	5	111	14.6	13.2	0.8	0.6
64	M	160.5	52.6	20.42	19.2	74	140	78	98.67	59	10.6	6.9	4.3	1.7	0.6	33	17	15	186	184	72	73	96.6	6.3	47.7	440	14.2	44.1	22.5	300	14.1	0.83	140	4.6	102	9.2	4.1	89	14.8	18.2	0.6	0.7
62	F	147.3	54.6	25.16	28.9	76.5	124	69	87.33	64	9.9	6.6	4.1	1.6	0.6	22	30	131	327	222	66	76	132.8	5.2	54	441	12.7	39.6	17.9	282	15.2	0.71	143	3.9	104	9.4	6.6	70	13.7	13.4	0.7	0.9
84	M	154.5	51	21.37	17.5	80	135	77	96.33	56	118	7.1	4	1.3	0.7	22	20	38	221	149	79	53	80.2	5.7	73.9	476	14.1	43.6	23	367	12	0.86	140	5.2	102	9.7	5.4	81	13	12.5	1	1.2
80	F	135.3	41.1	22.45	30.1	74	132	85	100.7	93	7.8	8	4.7	1.4	0.6	31	15	28	276	244	76	90	138.8	4.6	78.5	484	15.1	46.5	24.3	334	13.3	0.41	143	4.1	101	9.3	4.4	69	12.8	13.1	0.6	0.8
63	M	172.5	60	20.16	26.5	78	127	81	96.33	58	38.1	7.4	4.5	1.6	0.8	18	17	24	165	250	81	71	162.8	5.1	77.5	489	15.3	46.5	21.9	296	15.5	0.86	142	4.1	104	9.7	7.2	116	11	12.1	0.7	0.7
54	M	166.4	88.8	32.07	34.1	108.5	136	80	98.67	119	3.7	8.1	4.5	1.3	1.7	51	28	93	426	171	103	67	83.4	7.5	50	512	15.7	47.2	12.4	188	12	0.96	142	3.6	102	9.4	5.4	62	15.5	13.6	0.7	0.7
54	M	174.2	73.6	24.25	21.9	84	123	78	93	53	11.4	7.1	4.4	1.6	0.7	19	20	51	179	229	251	46	132.8	5.4	69.6	527	15.7	47.3	21.8	233	12.8	0.79	142	4.3	105	9.3	6	72	9.8	9.7	0.7	0.7
53	M	167.5	55.9	19.92	17.5	75.2	115	74	87.67	54	2.5	7.2	4.2	1.4	0.5	25	23	42	142	202	58	81	109.4	5.2	51.2	472	14.6	42.9	24	225	20.4	0.81	139	4.2	102	8.9	5.2	80	12.3	15.3	0.5	0.5
51	M	164.1	54.3	20.16	18.6	71	113	70	84.33	57	5	7.4	4.5	1.6	1.3	33	25	33	163	211	74	95	101.2	5.1	47.8	451	14.9	44.4	13.1	186	22.3	0.8	143	4.4	103	9.2	5.1	105	13.5	15.5	0.5	0.5
67	F	151.1	51.1	22.38	27.4	77	132	72	92	82	6.5	7.4	4.5	1.6	0.7	19	10	15	199	243	99	106	117.2	5.2	34.9	412	12.1	38.2	29.1	340	13.3	0.55	141	3.9	102	9.4	4.3	297	12.9	12.7	0.7	0.8
59	F	153.7	48.3	20.45	27.3	76.5	114	61	78.67	69	9.5	7.2	4.2	1.4	0.4	17	9	15	280	218	89	53	147.2	5.1	62.4	390	11.3	35	32.3	328	15.9	0.56	142	3.9	105	9.1	3.7	129	12.9	11.4	0.8	0.7
56	F	155.9	49.6	20.41	26.1	71	94	44	60.67	59	27.1	6.9	4.4	1.8	0.6	26	16	14	171	207	58	80	115.4	5.4	50.1	417	12.5	38.6	16.4	306	19.5	0.89	142	4.4	102	9.2	4.8	123	9.5	9.1	0.7	0.5
56	F	157.3	51	20.61	26.8	75	125	84	97.67	90	3.2	7.7	4.8	1.7	1	22	28	41	308	282	266	54	174.8	5.4	57.2	425	13.1	40.3	21.4	277	13.2	0.86	142	4.5	101	10.1	4.9	117	14.7	14.3	0.5	0.8
63	F	149.2	59.7	26.82	35.6	83	142	84	103.3	59	40.5	7.3	4.6	1.7	0.6	17	12	17	162	192	100	63	109	7.1	63.5	428	13.2	40.2	29.3	353	14.1	0.58	141	4	102	9.5	3.3	91	19.4	17.7	0.5	1
61	M	169.3	84.4	29.45	36.3	106.5	127	83	97.67	83	47.8	7.6	4.6	1.5	0.6	47	49	166	220	213	302	49	103.6	6.5	67.9	510	17.6	51	10.9	269	14.2	0.85	139	4.6	101	9.4	6.7	46	14.2	16.6	0.9	1.7
58	F	153.3	62.6	26.64	35.4	84	129	82	97.67	48	7.8	7	4.3	1.6	0.9	23	14	24	181	191	51	119	61.8	5	27.6	380	12.5	38.2	16.7	286	13.8	0.59	144	4.1	104	9.3	5.9	56	15.2	15.5	0.8	0.7
51	F	156	65.4	26.87	46.2	90	115	79	91	69	4.1	7.3	4.5	1.6	1	17	19	17	174	277	112	87	167.6	4.8	55.9	492	13.2	41.7	29.3	273	13.6	0.53	141	4.3	103	9.3	3.7	85	14.5	15.7	0.7	0.7
66	F	153.5	80.4	34.12	27	108	134	79	97.33	63	7.6	7.1	4.1	1.4	0.7	53	68	26	215	180	132	53	100.6	6.1	68.2	469	14.1	42.9	17.5	241	12.8	0.51	141	4.4	103	8.9	5.9	57	8.9	7.9	0.9	0.9
42	M	167.7	60.3	21.44	16.5	68.5	138	72	94	49	2.1	7	4.2	1.5	1.2	28	18	25	253	186	84	66	103.2	4.7	55.7	513	15.3	44.3	23.3	273	12.8	0.84	142	4.3	102	9.2	6.1	104	14.4	11.7	0.7	0.7
67	F	155.9	54.3	22.34	29.8	79.5	121	74	89.67	60	26	7.1	4.5	1.7	0.7	25	20	16	242	209	86	71	120.8	5.1	54.7	442	13.1	41.1	27	306	11.1	0.54	144	4.2	105	9.6	4.2	61	10.9	7.7	1	0.8
67	M	160.3	75.6	29.42	28.6	97.5	134	82	99.33	71	8.6	6.8	4.5	2	1.4	31	7	16	186	224	128	51	147.4	5	44.2	503	16.2	48.2	16.9	273	11.7	0.93	144	4.9	104	9.2	5.3	117	14.4	15.6	0.6	0.7
64	M	162.1	53.2	20.25	20.1	72	146	89	108	72	2	7.5	4.6	1.6	0.5	84	86	116	260	133	154	51	51.2	5.2	64.6	484	16.2	47.1	19.1	254	14.8	0.93	140	4.2	99	9	6.3	136	14.2	16.3	1.4	1.4
64	M	158.9	61.9	24.52	27.6	83	134	86	102	67	3.3	7.6	4.6	1.5	0.8	27	26	92	170	221	164	69	119.2	5.1	49.6	478	14.2	42.6	27.6	261	18.8	0.8	142	3.8	100	9.4	5.9	63	20.9	21.1	0.8	0.8
49	F	148.1	56.4	25.71	41	89	135	85	101.7	73	2.3	7.8	4.6	1.4	0.8	26	44	33	201	230	130	60	144	5.9	58.5	483	13.5	40.4	25.2	215	12.8	0.5	141	3.9	103	9.3	5.4	59	11.9	13.2	0.6	0.7
60	M	164.6	76	28.05	32.4	92.5	127	77	93.67	51	27.7	6.7	4.2	1.7	1	40	60	47	203	186	80	45	125	7.1	71.1	560	16.1	48.7	16.2	244	14.2	0.84	143	4.2	106	8.8	5.2	117	11.8	9.1	1.1	1.1
65	M	171.7	84.2	28.56	31.3	108	149	79	102.3	64	2.1	7.5	4.7	1.7	0.5	18	33	40	184	206	89	53	135.2	5.9	63.9	505	15.2	46.4	23.9	353	13.8	0.63	142	4.3	105	9.5	5.7	89	13.8	14.2	0.8	1
60	F	154.9	59.4	24.76	29.3	82	161	88	112.3	56	21.2	7.5	4.3	1.3	0.9	17	14	18	190	197	95	86	92	5.1	74.6	438	14.4	42.2	30.1	296	10.9	0.63	141	3.8	100	9.4	4	73	9.7	10.4	0.8	0.9
64	M	163.1	54.5	20.49	17.9	75	132	90	104	70	12.1	7	3.7	1.1	0.9	20	18	39	257	216	96	78	118.8	5.3	54.1	455	14.4	44	20.4	296	14	0.97	140	3.7	103	8.7	6.7	74	11.1	13	0.7	0.7
67	M	159.3	62.6	24.67	26.3	86.5	142	72	95.33	54	14.4	6.7	4.1	1.6	0.6	34	51	38	242	190	94	46	125.2	5.5	67.3	492	15.1	46.5	26.1	291	16.3	0.77	142	4	102	9.1	6.7	94	8.2	12.7	1.1	0.8
57	F	152.4	52.4	22.56	29.7	73.5	158	89	112	75	7	7.7	4.7	1.6	1.1	20	13	14	289	209	81	66	126.8	4.7	59	464	14.5	42.4	24.3	230	17.4	0.59	142	4	102	9.6	4.6	71	11.4	14.9	0.8	0.8
56	M	164.1	69.2	25.7	25.6	86	131	93	105.7	71	3.1	7.4	4.6	1.6	0.7	16	27	74	199	179	141	35	115.8	5.1	55.8	555	16.1	48.9	21.5	250	13.7	1.05	143	4.1	103	9.3	6.4	80	8.3	11.1	0.6	0.6
58	F	145.3	48.6	23.02	29.4	79	136	67	90	40	10.8	7	4.4	1.7	0.5	15	12	20	207	208	77	82	110.6	5.2	47.3	401	12.5	38.6	17.5	233	18.2	0.61	141	4	102	9.1	4.6	42	15.5	14.9	0.7	0.7
53	F	158.2	70.6	28.21	37.4	95	132	77	95.33	75	18.2	7.4	4.1	1.2	0.6	23	19	20	208	179	73	59	105.4	5	72.8	467	12.6	38.4	25.3	347	16.5	0.71	139	4	104	9.1	5.8	85	11.6	12.9	0.5	0.6
64	M	165.8	58.6	21.32	18.6	73.5	136	85	102	61	2	7.6	4.4	1.4	0.8	29	23	36	203	240	89	64	158.2	5.2	31.5	445	13.7	41.6	19.9	431	10	0.63	140	4	99	9.4	4.8	57	12.5	11.4	0.6	0.7
62	M	168.2	63.8	22.55	19.1	83	138	87	104	55	6.6	7	4.3	1.6	0.8	18	12	15	203	154	39	62	84.2	5	58.2	465	15	45.2	22.4	222	16.6	0.8	141	4.1	102	9.1	4.9	73	12.1	11.6	0.7	0.7
69	F	148.7	47.4	21.44	26	74.5	143	79	100.3	61	30.7	7.3	4.5	1.6	0.5	24	18	28	195	198	122	64	109.6	5.8	44.9	408	13.2	39.8	21.9	273	11.7	0.53	142	4.4	102	9.2	2.8	64	12.2	18.8	1	1.1
65	M	162.5	60.2	22.8	22.2	80.5	153	92	112.3	51	37.3	7.5	4.3	1.3	1.5	23	15	29	141	233	37	95	130.6	5.1	50.3	473	15.4	45.2	17.6	238	14.8	0.68	139	4.4	99	9.3	5.2	82	13.9	13.9	1.1	0.8
57	F	159.1	46.9	18.53	22	69	94	48	63.33	61	11.8	7	4.5	1.8	1	29	21	12	263	211	49	76	125.2	4.9	35.6	377	13	39.4	16.4	291	17.1	0.53	142	4.9	103	9.8	3.3	114	9.9	7.8	0.5	0.5
72	M	163.5	68.8	25.74	28.8	89.5	137	83	101	54	22.9	7.1	4.4	1.6	0.9	23	23	43	254	200	177	47	117.6	7	66	531	18	53.7	14.1	328	14	0.69	142	4.2	101	9.7	5.2	60	19.9	18.8	0.8	1.4
71	F	138.1	46.7	24.49	35.2	76.5	148	76	100	59	35.9	7.8	4.5	1.4	0.6	19	14	18	383	151	71	43	93.8	4.8	44.6	442	13.6	40.7	19.2	375	14.5	0.66	145	4	107	9.3	5.1	92	13	15.3	0.8	0.9
66	F	141.1	43.8	22	25.5	73.5	139	84	102.3	105	10.6	7.3	4.5	1.6	0.8	22	21	22	239	199	150	67	102	5.8	68.5	445	14.1	42.5	22.5	340	14.2	0.67	142	4.6	103	9.2	5.8	86	13.2	14.2	0.8	0.9
67	M	164.8	58.2	21.43	19.6	79	136	82	100	66	7.1	7	4.3	1.6	0.8	27	21	38	294	195	112	67	105.6	5.3	97.6	468	15	47	24	316	13.8	0.8	142	4	102	9.4	4.8	48	15.8	14	0.6	0.8
53	F	154.4	53.8	22.57	31.5	80.5	138	80	99.33	59	23.9	7.5	4.2	1.3	0.6	18	13	13	240	232	190	48	146	5.4	54.6	546	14.1	43.2	20.8	286	15.8	0.61	141	3.7	103	8.7	4.5	82	15.2	13.2	0.7	0.6
64	F	154.8	51.1	21.32	29.3	82.5	119	74	89	61	4.3	7	4.2	1.5	0.5	38	35	101	207	238	121	80	133.8	5.3	70.9	441	13.3	40.8	25.5	261	21.2	0.45	138	3.9	102	9	5.2	65	11.4	12.6	0.7	0.7
53	F	163.9	70.1	26.1	36.7	90	113	69	83.67	82	2	7.4	4.3	1.4	0.8	27	36	34	368	203	252	50	102.6	5.1	68.7	504	15	44.5	23.3	257	14	0.65	142	4.1	104	9	5.4	56	10	8.4	0.6	0.6
50	M	166.4	75.8	27.38	28.3	88.5	132	83	99.33	59	15.6	6.9	4.2	1.6	1.1	25	19	33	184	197	84	63	117.2	5.3	39.7	487	15.5	46.6	22.2	306	9.7	0.88	141	4	103	9.1	6.1	78	10.5	12.3	0.6	0.8
65	F	152.1	53.6	23.17	34.5	80	115	63	80.33	68	17.5	7.6	4.4	1.4	0.6	26	21	77	254	292	164	68	191.2	5.1	36.3	447	13.5	41.7	20.8	347	13.2	0.81	142	3.8	102	9.3	4.7	122	10.2	12.6	0.7	0.8
66	F	154.1	67.8	28.55	42.6	99	179	103	128.3	68	25.2	7.3	4.4	1.5	0.8	24	21	40	171	214	114	64	127.2	5.2	72.1	455	13.8	41.1	22.2	316	17.6	0.74	142	3.5	100	9.5	5	54	13.5	14.6	0.8	1.4
89	M	165.1	47.1	17.28	11.6	65	122	66	84.67	67	90.8	7.4	4.5	1.6	0.6	45	26	35	238	177	102	54	102.6	5.3	41	430	12.9	41	13.7	322	18.5	0.9	140	4.8	96	8.9	4.9	160	8	8.4	0.8	0.9
68	M	163.7	62.6	23.36	23.2	80.5	135	86	102.3	58	12.4	8.2	4.1	1	0.8	38	35	23	204	201	105	40	140	5.2	71.8	458	15.4	45.6	15.1	334	22	1.04	142	4.9	102	9.2	7.8	133	10.7	9.7	0.7	0.9
60	M	163.7	61.4	22.91	24.9	79	114	74	87.33	58	7.4	7.3	4.5	1.6	0.4	19	18	24	245	209	198	44	125.4	5.4	72.5	506	15.9	48.1	32.5	296	8.8	0.89	141	4	100	9.6	6.6	87	13.8	12.2	0.7	0.7
60	F	147.3	49.4	22.77	25.2	74.5	151	82	105	69	27.7	6.6	4.2	1.8	0.5	21	17	17	237	188	53	70	107.4	5.1	56.6	427	12.8	38.9	23.2	220	18.5	0.5	143	4	104	9.2	3.6	75	11.9	11.3	0.9	0.7
60	M	174.1	74.4	24.55	27.1	83.5	126	78	94	67	8.5	7.5	4.4	1.4	1	16	17	31	237	258	99	60	178.2	4.9	51.9	483	15.7	48.2	24	488	16.9	0.68	141	4	102	9.1	5.1	78	13.6	13.1	0.7	0.5
72	M	153.7	56.5	23.92	17	80	158	95	116	87	38.5	6.7	4.2	1.7	1.1	28	22	26	159	242	70	78	150	4.9	48	447	14.6	42.1	18.4	220	18.6	1.04	142	4.2	105	9.3	7	103	15.7	15	0.6	2.6
66	F	150.2	44.1	19.55	24.3	68.5	134	68	90	86	18.1	7.6	4.3	1.3	0.8	22	22	20	309	194	156	43	119.8	5.8	91.8	502	14	44.4	18.9	367	17.2	0.64	141	4.2	101	9.1	5.3	67	14.2	16.8	0.7	0.9
54	M	160.5	71.1	27.6	23.8	91	147	95	112.3	62	24.8	7.4	4.1	1.2	1.2	29	36	35	230	264	225	42	177	5	92.6	555	16.1	48.5	23.5	311	11.2	0.87	142	4	102	8.8	6.2	66	12.4	14.8	0.9	2

64	F	156.9	49	19.9	25.9	74	143	81	101.7	60	13.9	7.4	4.5	1.6	0.7	19	13	17	283	191	71	59	117.8	6	41.5	425	12.7	39.3	16.4	261	13.4	0.57	142	4.1	101	9.5	3	58	14.7	13.1	0.8	0.8
44	M	173.3	76.7	25.54	25.9	87	140	84	102.7	65	5.8	7.5	4.3	1.3	0.7	35	53	101	176	209	470	40	75	5.3	60.7	470	14.1	42.2	27.3	197	12.8	0.86	141	4.2	103	9.2	7.2	139	14.6	15.3	0.6	0.6
52	F	154.6	68.7	28.74	47.7	96	108	74	85.33	72	2.5	7.1	4	1.3	0.5	83	163	39	297	243	131	52	164.8	6	85.2	435	14.1	41.6	22.4	334	8.9	0.53	142	4	105	9.2	4.7	44	14	15	0.7	0.6
55	M	166.3	71.3	25.78	31	90	139	91	107	69	12	7.2	4.3	1.5	1.1	16	18	73	123	255	123	83	147.4	5	97	436	14.4	42.6	26.9	222	14.2	0.66	142	3.9	100	8.9	4.5	50	13.9	12.5	0.8	0.6
65	F	153.9	53.1	22.42	33.4	74.5	121	61	81	57	6.6	6.9	4.1	1.5	0.8	21	15	13	189	240	93	60	161.4	5	51.8	428	13.6	41.4	20.1	265	13.3	0.68	143	4.1	103	9.3	4.6	53	7.8	9.1	0.8	0.9
58	M	168.3	84.4	29.8	31.9	98.5	125	73	90.33	48	14.5	7.6	4.7	1.6	2.1	29	28	22	199	178	73	63	100.4	5.2	59	516	16.7	49.5	16.1	241	15.2	0.94	144	4.1	101	9.8	7.9	138	13.4	12.6	0.7	0.6
55	F	158.6	64.8	25.76	35.9	83.5	166	91	116	63	11.9	7	4.3	1.6	0.7	18	16	40	129	229	80	76	137	5.2	47.8	492	14	43.6	31	230	15.3	0.54	143	4.3	104	9	5.1	89	16.7	15.4	0.7	0.7
61	F	156.1	53	21.75	23.4	71	130	68	88.67	55	12.2	7.7	4.2	1.2	0.9	18	13	17	172	239	79	84	139.2	5.3	91.7	447	13.5	42.4	21.9	282	15.3	0.66	142	3.9	103	9	4.7	107	13	11.9	0.6	0.6
53	M	164.1	72.5	26.92	28.2	92.5	127	79	95	77	2.8	7	4.2	1.5	0.5	27	41	45	397	220	531	34	79.8	6.1	70.7	551	16.2	48.5	21.7	254	11	1.05	142	4.1	104	9	6.9	65	11.1	10.8	0.7	0.5
65	F	142.2	45.9	22.7	31.5	78	149	95	113	82	11	8.5	4.4	1.1	0.7	19	14	18	176	219	159	49	138.2	5.2	48.8	429	12.6	39.3	18.7	273	22.4	0.6	143	3.5	103	9.4	5.6	149	15.4	13.3	1	1
68	M	161.3	57.3	22.02	21.7	76	160	85	110	87	6.7	7.7	4.5	1.4	0.7	28	34	33	283	219	130	49	144	5.4	61.4	554	16.4	50.2	16.7	411	19.6	0.93	143	4	102	9.3	6.5	114	18.5	19.7	1.4	1.2
66	F	150.7	59.7	26.29	42.8	95	117	64	81.67	71	8.7	7.6	4.4	1.4	0.8	17	13	24	255	324	204	65	218.2	5.5	52.9	463	14.1	44.4	26.8	353	16.8	0.47	142	3.9	102	9.4	4.6	84	15.8	13.2	0.5	0.7
67	M	175.2	74.5	24.27	29.1	97	143	95	111	57	4.2	7.1	4	1.3	0.9	19	12	33	159	192	174	43	114.2	5.6	73.8	470	15.2	46.7	16.7	347	17.7	0.88	140	4.1	101	8.8	5.8	80	7.3	7.8	0.8	2.1
55	F	153.5	68.1	28.9	47	93	131	71	91	68	18.2	7.5	4.4	1.4	0.5	48	61	47	287	165	121	58	82.8	7.2	42.6	433	13	40.6	15.9	273	10.3	0.53	144	3.9	104	9.4	3.3	61	12.8	11.7	0.6	0.7
68	F	146.5	45.1	21.01	28.4	74.5	154	90	111.3	69	11.4	7.2	4.2	1.4	1.1	24	10	10	245	198	49	67	121.2	5	59.2	421	12.6	40	22.6	291	15.6	0.59	141	3.4	101	8.9	3.8	78	13.3	13.3	0.7	0.7
57	F	156.6	59.1	24.1	30.4	83.5	150	85	106.7	74	10.4	7.7	4.7	1.6	1.2	13	14	21	264	256	108	63	171.4	5.3	34.1	483	14.1	43.2	23.6	311	13.9	0.54	144	4.5	103	9.9	4.3	78	19	15.6	0.7	0.7
57	F	152.1	53.8	23.26	32.7	79.5	130	77	94.67	52	9.8	8.5	4.3	1	0.7	21	15	18	326	225	137	42	155.6	5	51	414	13.2	40.1	19.5	334	11.8	0.53	142	4.3	103	9.3	5.5	96	15.7	11.5	0.9	0.8
67	F	147.7	46.5	21.32	29.7	69.5	121	71	87.67	72	37.8	6.7	4.1	1.6	0.7	21	17	26	336	215	72	104	96.6	5.8	57	431	13.4	41.6	17.1	273	10.4	0.54	143	4.3	100	9.3	4.2	106	9.4	11.3	0.7	0.6
65	F	151.7	50.4	21.9	27.6	74.5	130	79	96	67	21.1	7.5	4.3	1.3	0.7	20	16	17	264	257	178	54	167.4	5.7	63.6	447	13.7	42.7	27.4	340	11.5	0.61	143	4	104	9.6	3.6	82	13.8	12.6	0.6	0.6
64	M	172.5	65.3	21.94	23.1	81	147	98	114.3	83	4.7	7.9	4.7	1.5	0.7	44	35	158	199	217	162	59	125.6	5.2	61.6	545	17.2	50.4	17.9	316	14.2	0.77	142	3.9	102	9.7	6.2	122	15.2	16.3	0.8	0.8
61	F	153.5	50.1	21.26	31.6	76.5	150	82	104.7	75	6	7.9	4.4	1.3	0.8	19	20	30	240	210	60	55	143	5.3	50.3	537	15.8	47.6	26.3	316	10.2	0.58	143	4.2	104	9.6	5.4	138	11.8	13	1	1.2
63	M	172.9	63.7	21.31	16.8	78	151	86	107.7	53	21.4	7	4.2	1.5	0.7	21	15	30	178	231	88	79	134.4	5.3	47.5	440	14.1	43.5	21.1	311	20.3	0.73	142	3.9	105	8.9	5.6	94	11.9	9.5	0.7	0.8
64	F	148.3	66.8	30.37	41.5	92	123	81	95	61	26.4	7.5	4.4	1.4	0.9	23	20	17	214	220	99	66	134.2	5.2	50	485	14.8	45.8	22.7	257	11.3	0.5	141	3.8	99	9.5	3.8	98	9.5	11.4	0.7	0.8
51	M	166.1	67.4	24.43	26.4	83.5	139	84	102.3	48	4	7	4.6	1.9	0.7	26	19	34	145	172	60	71	89	5.2	53.7	481	14.9	44.8	20.4	235	18.9	1.03	143	4.1	104	9.5	7.8	49	19.6	14.7	0.8	0.8
66	M	166	63.6	23.08	17.6	85.5	153	94	113.7	55	18	7.2	4.5	1.7	0.8	21	16	19	190	220	93	48	153.4	6.2	61.2	430	13.1	40.5	21.5	273	21.6	1.07	140	4.3	103	8.9	6.1	67	8.5	9.5	0.7	0.8
64	F	157.7	64.5	25.94	36.2	94.5	147	83	104.3	70	42.4	6.7	4.2	1.7	0.5	15	11	14	125	250	163	46	171.4	5.8	51.7	522	13.2	42.1	21.5	269	12.2	0.56	144	4.5	104	8.8	4.5	74	12.7	10.1	0.6	0.8
65	F	154.5	49.7	20.82	24.6	76.5	114	73	86.67	61	23.1	6.9	3.9	1.3	0.7	25	25	19	173	172	87	73	81.6	5.6	49.4	435	12.8	39.5	25.2	282	19.8	0.68	145	4.2	106	9	5.5	68	10.5	9.2	0.8	0.8
65	M	162.5	58	21.96	17.2	78	164	105	124.7	71	10.8	7.4	4.4	1.5	1.4	21	17	23	238	199	88	86	95.4	5	48.7	511	15.9	47.6	18.1	176	17.4	0.87	142	3.8	102	9.1	0.8	85	11.8	12.6	0.7	0.8
66	M	159.6	64.1	25.16	28.5	82	154	87	109.3	79	6.4	7.5	4.3	1.3	0.5	22	18	91	159	202	153	94	77.4	5	44.9	457	14	42.6	25.6	213	23.8	0.92	141	4.6	101	9.2	7.3	87	11.2	13.2	1.3	0.9
51	M	164.9	56.7	20.85	17.7	71.5	154	110	124.7	69	10.5	8.2	4.1	1	0.7	22	15	29	266	192	127	49	117.6	5.2	84.5	549	14	43.4	30.7	431	15.7	1.17	138	4	99	9.4	5.6	146	15	16	0.7	0.8
62	F	151.3	38.2	16.69	16.1	62	124	77	92.67	72	15.5	7.3	4.6	1.7	0.7	24	13	19	258	173	66	70	89.8	5.4	35.5	477	13.2	40.6	26.7	291	10.6	0.45	142	3.7	102	8.9	3.3	90	11.7	9.8	0.6	0.7
70	M	161.7	68.4	26.16	22.2	87	154	87	109.3	71	6.1	8	4.3	1.2	0.9	24	37	33	209	215	149	49	136.2	5.3	72.7	475	14.6	45.2	25.3	261	13.2	0.81	141	4.2	103	9.1	6.1	69	15.5	13.3	1	0.7
62	F	155.3	57.3	23.76	31	80.5	116	61	79.33	61	7.3	6.9	4.3	1.7	0.7	26	18	16	264	234	86	73	143.8	4.9	57.1	462	14.3	46	14.7	209	17.1	0.57	143	3.9	101	9.2	5.6	87	15.3	12.2	0.7	0.9
59	M	165.8	66.3	24.12	22.7	82.5	126	81	96	62	8.5	7	4.2	1.5	0.9	23	17	67	230	220	316	51	105.8	5.1	77	470	16.4	47.6	21.8	311	11.6	0.71	145	3.9	105	9.3	5.7	66	11.3	11.2	0.7	0.5
64	M	160.9	57.6	22.25	22.8	75	150	81	104	52	8.5	8.1	4.9	1.5	0.8	26	26	53	259	225	125	54	146	5.5	72.7	530	15.4	46.9	23	277	12.4	0.69	142	4.1	101	9.5	5.9	151	13.3	14.1	1	0.8
81	M	165.7	62.3	22.69	26.3	81.5	132	79	96.67	61	25.2	7.5	4.2	1.3	0.6	27	17	15	277	175	81	46	112.8	5.5	59.7	508	16	50	23.6	360	13.8	0.9	144	3.7	103	8.9	5.4	144	7.8	10.5	1.6	1.7
80	F	154.1	52.3	22.02	26.8	81.5	134	75	94.67	63	40.7	7.4	4.5	1.6	1.1	21	13	12	185	208	142	48	131.6	5.4	27.2	391	12.1	37.3	11	265	14.3	0.71	143	3.8	104	9.4	6	84	10.7	10.7	0.6	0.9
55	F	156.5	68	27.76	42.5	96	114	67	82.67	61	9.3	7.6	4.3	1.3	1.1	20	18	28	297	265	326	37	162.8	5	69.9	460	14.4	42.5	29	254	11.5	0.55	139	3.5	101	9.6	4.3	51	12	11.7	0.7	0.8
60	F	159.7	63.5	24.9	36.4	85.5	144	84	104	56	7.1	7.7	4.5	1.4	0.7	21	19	23	215	228	108	42	164.4	5.4	52.8	435	13.6	42	25.1	450	13.8	0.68	141	3.7	100	9.5	5.8	63	18	18.5	0.6	0.9
51	F	171.5	69.4	23.6	29	80	144	77	99.33	48	21.2	7.5	4.5	1.5	0.6	22	27	14	177	218	54	84	123.2	5.2	32.7	499	14.5	44.2	18.5	241	12	0.62	145	4.1	106	9.2	3.6	29	14.5	14.4	2.1	2.5
67	M	166.2	70.1	25.38	27.1	88	125	73	90.33	81	31.1	7	4.2	1.5	0.6	22	25	86	328	211	95	74	118	8.7	65.5	481	16	47.7	13.1	204	22	0.62	139	4.2	98	9	5.3	101	16.2	13.4	0.8	1
67	F	153.7	50.3	21.29	31.6	83.5	142	83	102.7	63	10.7	7.4	4.5	1.6	1	26	19	16	184	219	36	76	135.8	4.9	51.4	466	13.5	42.1	18.1	296	17.7	0.58	142	4.4	102	9.5	4.1	115	11.3	11.4	0.9	0.8
64	M	160.2	44.9	17.5	12.4	65	147	80	102.3	57	60.4	7.3	4	1.2	0.9	37	28	61	199	152	112	80	49.6	6.7	68.4	398	14.1	42.7	20.6	235	11.5	0.68	140	5.1	98	9.1	4.2	31	16.8	17.2	0.7	0.7
65	M	163.7	53.8	20.08	18.8	69	116	74	88	62	10.2	7.3	4.3	1.4	0.8	23	21	15	173	239	108	72	145.4	6.7	67.7	550	15	46.8	16.2	286	20.3	0.94	142	5.1	102	9.7	5	133	15.8	14.8	0.6	0.7
62	F	153.7	51.8	21.93	29.9	79	156	83	107.3	69	7.6	7.1	4.4	1.6	0.7	19	12	26	305	280	124	49	206.2	5.4	64.3	433	13.7	41.1	19.4	300	13.7	0.57	143	3.8	103	9	4.7	75	10.6	10.4	0.7	1
59	F	149.7	51.1	22.8	31.7	75.5	131	70	90.33	56	27.8	7.2	4	1.3	0.5	29	28	51	478	239	70	58	167	5.7	59.2	495	13.7	42.5	17.5	230	18.4	0.56	143	4	103	9	4.3	112	14.8	11	1	0.7
63	M	159.3	52.8	20.81	23.1	75	123	75	91	67	8.5	8	4.9	1.6	0.5	22	30	76	362	217	217	40	133.6	5.8	75.9	455	14.6	43.7	37.9	296	12.4	0.68	142	4.2	102	9.9	6.5	67	14.4	15.7	0.6	0.6
67	M	175.7	72.4	23.45	25	88.5	129	81	97	80	6.4	7.8	4.7	1.5	1.3	22	24	60	191	188	185	37	114	5	56.6	559	16.6	48	26.3	244	10.8	0.88	141	3.8	102	9.1	5.4	70	14.5	11.8	0.6	0.8
68	F	152.5	46.2	19.87	17.1	65.5	115	61	79	62	17.2	7.2	4.8	2	1.1	24	16	25	216	236	78	88	132.4	5.6	45.7	445	14	43	21.5	296	14.6	0.66	144	4	104	9.4	3.8	115	14.2	13.1	0.7	0.7
60	F	164.5	53.8	19.88	20.5	69.5	130	75	93.33	69	17.1	7.5	4.1	1.2	0.7	27	12	38	194	243	80	83	144	5.2	30	421	13	39.8	20.8	300	13	0.74	142	3.9	104	9.4	5	113	14.2	15	1.3	0.6
77	M	164.1	65.2	24.21	21.5	87	148	92	110.7	74	10	6.9	4.3	1.7	0.9	16	15	24	253	195	101	57	117.8	6.4	42.8	445	13.3	41.3	16.7	261	17.4	0.84	142	3.8	106	9	3.4	54	11.7	10.2	0.9	0.8
58	M	169.5	77.7	27.04	35.2	93.5	141	87	105	84	7.1	7.2	4.3	1.5	0.4	24	25	74	195	249	296	70	119.8	5.6	55.5	495	15.3	46.8	24.8	222	13.7	0.78	142	4.2	104	9.1	5.6	70	18.6	13.4	1.1	0.8
69	F	147.6	58.7	26.94	33.2	84	145	68	93.67	53	34.1	7.4	3.9	1.1	0.8	14	20	24	358	220	122	47	148.6	7.8	48.9	505	14.8	46.6	20.7	286	14.9	0.62	142	4	106	8.8	4	55	11.2	12.7	0.7	0.8
68	M	162.7	60.5	22.85	21.8	81.5	139	84	102.3	76	15.2	7.3	4.5	1.6	1.3	28	34	98	200	200	84	87	96.2	5.1	43	384	14	41.5	16	250	11.5	0.66	141	3.8	104	9.3	4.2	64	10.1	10	0.7	1.6
68	M	164.9	63.3	23.28	26.1	80.5	122	76	91.33	65	5	7.6	4.5	1.5	0.9	21	20	22	277	169	125	34	110	9.3	54.4	504	15.4	46.7	16.4	340	18.1	0.54	139	4.2	102	9.2	5.3	81	10.7	8.5	0.7	0.6
62	M	153.7	56.3	23.83	22.4	83.5	136	71	92.67	49	40.7	7.2	4.3	1.5	1.3	16	17	26	167	207	54	97	99.2	4.8	50.3	460	14.6	45.8	21.5	182	14.4	0.75	141	4.4	104	9.4	4.7	81	8	9.6	0.9	0.9
60	F	146.5	43.9	20.45	26.6	74	137	84	101.7	59	8.4	7.2	4.4	1.6	0.9	22	17	16	266	203	49	71	122.2	5.2	44.8	438	12.9	40.4	20.5	300	16.9	0.56	141	3.9	105	9.1	4.4	72	14.8	14.1	0.6	0.7
52	F	162.5	80	30.3	44.5	95.5	166	111	129.3	68	9.6	7.7	4.5	1.4	1	23	42	23	238	271	151	63	177.8	5.8	50	484	14.5	44.7	27.4	291	18.1	0.67	144	3.8	105	10	6.3	40	10.6	11	0.8	0.9
61	F	150.1	54.2	24.06	30	79.5	125	70	88.33	68	9.3	6.8	4.3	1.7	0.7	23	26	37	243	226	157	50	144.6	5.7	76.1	473	13.9	43.4	25.1	311	15.3	0.61	144	3.7	105	9	4.6	74	15.7	16.7	0.6	0.7

80	M	154.8	57.5	24	27.2	85	133	80	97.67	68	12	7.3	4.1	1.3	1.2	18	13	19	217	262	116	63	175.8	5.9	55.9	456	13.8	42.5	20.3	306	16.2	0.9	141	3.9	103	8.8	4.6	72	13	12.8	1.4	1.1
76	F	142.8	49.7	24.37	38.3	75	119	79	92.33	81	24.9	7.1	4.2	1.4	0.9	25	11	15	197	182	101	64	97.8	5.1	41.9	451	13	40.6	14.5	247	17.9	0.57	143	4.1	105	9.2	4.2	90	10.6	10.5	0.8	0.6
70	F	145	51.1	24.3	31.3	71.3	130	65	86.67	59	15.4	7.8	4.6	1.4	0.6	23	16	15	169	212	89	61	133.2	5.3	53.1	388	12.6	38.8	16.4	269	11.6	0.56	144	4.1	105	9.2	4.9	117	10.7	10.8	0.8	0.7
71	M	165.3	69	25.25	23.8	86.5	146	82	103.3	57	19.2	7.7	4.2	1.2	0.8	21	18	50	258	176	109	48	106.2	5	69.5	483	14.9	45.2	23.4	311	15.7	0.92	141	4.1	102	9.4	5.6	130	11.4	12.2	1	0.8
60	M	171.7	67.9	23.03	23.9	80	135	87	103	64	17.8	7.7	4.4	1.3	1	25	27	61	132	236	111	54	159.8	5.3	71.6	525	16.5	50.3	20.5	347	15.2	0.93	142	4	103	9.4	5.9	92	10.5	10.3	0.8	0.8
64	F	158.3	63.2	25.22	31.3	92.5	185	86	119	67	43.5	7.2	4.4	1.6	0.6	21	15	17	152	171	112	47	101.6	5.6	60.2	462	13.9	43.5	18.8	235	12.8	0.44	145	3.6	107	9.2	5	56	9.3	7.9	0.9	0.8
69	M	164.5	68.8	25.42	23	88	139	92	107.7	82	4.7	7.5	4.4	1.4	0.8	21	38	35	164	183	139	40	115.2	5.4	70.1	501	14.6	43.9	23.5	265	11.9	0.76	141	4.3	105	9.1	5.2	80	14.8	12.8	0.9	0.8
66	F	153.3	52.4	22.3	29.1	78	123	73	89.67	63	36.5	7.2	4.5	1.7	1.1	24	16	19	332	135	35	55	73	5	42.3	474	14	42.9	18.2	218	15.5	0.5	141	4	104	9.3	4.4	48	12.8	13.1	0.8	1
29	M	183.2	171.6	51.13	40.2	146	142	97	112	67	8.1	7	4.3	1.6	0.5	33	62	29	243	150	122	44	81.6	5.6	74.1	512	15.1	45.6	26.6	306	11.3	0.64	140	3.9	105	9	6	54	15.7	14.6	0.7	0.6
58	M	167.9	68.4	24.26	22.6	81.5	120	74	89.33	65	9.8	7.7	4.4	1.3	0.9	19	32	117	245	174	104	42	111.2	5.8	56.8	496	15	45.3	27.2	375	15.6	0.85	140	4.5	103	9.4	5.7	104	7.8	9.4	0.7	0.5
76	M	161.7	59.7	22.83	21.1	77.5	116	65	82	67	28.4	7.1	4	1.3	0.6	20	14	14	216	192	66	44	134.8	5.2	68.5	464	14.5	44.2	18.7	316	17.1	1.04	140	4.6	106	8.6	5.1	87	9.4	9.7	1	1.1
60	M	161.3	66.2	25.44	26.2	85	133	81	98.33	67	6.3	7.8	4.5	1.4	0.9	54	51	133	220	239	237	53	138.6	5.7	75.4	473	15.3	46.2	20.1	296	14.2	0.75	142	4.2	105	9.6	7.4	47	14.2	14.2	1.3	1.5
55	M	169.7	84.4	29.31	35.2	99	128	85	99.33	64	12.1	7.3	4.6	1.7	0.9	22	25	183	309	293	235	67	179	5.3	93.7	511	14.9	45.1	23.8	257	13.2	0.86	141	3.9	105	9.5	7.7	40	17.6	14.4	0.9	0.7
58	F	155.5	71.4	29.53	35.6	93.5	142	88	106	67	49.8	7.6	4.8	1.7	1	18	15	21	216	231	88	70	143.4	4.9	53.7	478	14.2	41.9	26.3	257	15.2	0.68	142	4	105	9.7	5.2	78	10	12.7	0.7	1
57	F	149.9	53.4	23.77	31.7	79.5	152	76	101.3	89	98.3	7.4	4.6	1.6	1.6	13	19	49	336	209	70	87	108	5.2	100	584	17.9	53.4	14.4	316	11.1	0.91	141	4.4	103	9.5	6	78	16.5	16.6	0.7	0.5
72	M	162.8	59.8	22.56	25	79	150	94	112.7	72	54.5	7.2	4.1	1.3	0.9	20	22	37	219	213	63	70	120	5.1	57.5	514	15.4	46.3	21	296	16.6	1.13	141	3.9	105	9.2	5.1	109	14.4	14.2	0.8	1.2
44	F	154.5	47	19.69	20.3	67	103	54	70.33	62	14.7	7	4.3	1.6	0.6	29	22	16	110	190	52	85	80	4.9	62.2	385	11.8	35.7	27.1	225	19.5	0.57	144	3.3	107	8.9	2.7	81	11.9	11.5	0.6	0.6
75	M	153.1	53.5	22.82	16.1	81.5	138	78	98	52	23.7	6.9	4.1	1.5	0.6	28	23	72	397	249	81	116	110	5.6	41.2	435	14	43.2	28.3	306	13	0.66	142	4.4	102	9.1	3.5	110	7.6	9.4	0.7	0.8
64	M	170.5	61	20.98	21.5	82	132	73	92.67	63	50	7.5	4.4	1.4	1.9	112	74	548	235	227	126	61	153	4.9	74.3	493	16.8	50.6	24.6	322	7.7	0.73	142	4.3	103	9.7	6.8	94	20.3	17.5	0.7	0.8
57	M	167.9	58.8	20.86	14.3	69	129	75	93	43	19.6	7.5	4.8	1.8	0.6	30	19	17	203	196	37	93	78	5.1	42	451	13.6	41.8	15.7	225	18.2	0.93	139	4.3	101	9.4	5.9	116	8	7.7	0.6	0.6
57	F	157.3	59.4	24.01	32.3	85	120	69	86	59	10.4	7.5	4.3	1.3	0.5	26	24	76	190	260	87	54	80	5.3	45	439	12.5	38.6	30.2	269	14.4	0.62	140	4	103	9.2	4	78	8.8	7.9	0.7	0.9
52	M	165.2	57.1	20.92	16.3	72	161	105	123.7	65	9.9	6.4	4	1.7	0.7	16	23	47	177	209	62	48	153	4.9	70.7	539	16.5	49.4	25.7	277	15	0.72	143	4.2	107	9	5.2	102	10.1	11.3	0.7	0.7
82	F	137.7	42.9	22.63	32.6	89.5	154	84	107.3	67	15.1	7.8	4.3	1.2	0.8	23	16	19	275	216	103	64	117	5.4	54.3	466	14.5	43.9	23.3	340	16.6	0.61	142	4.6	104	10	5.1	92	10.5	12	0.7	0.6
58	M	159.9	71.7	28.04	29.4	99	149	98	115	98	147	6.7	4.1	1.6	0.4	44	61	592	290	244	274	52	124	6.8	91.5	517	16.8	49.7	21.4	296	15.1	0.57	137	4.1	102	9	7.2	60	15.1	15.1	1.2	0.8
54	M	162.5	52.5	19.88	14.4	71.5	144	91	108.7	66	6.8	7.2	4.4	1.6	0.9	16	25	34	204	191	76	67	109	5.3	40.5	505	15.1	45.2	5.2	291	13.3	1.03	141	3.8	100	9.4	5.9	74	9.9	11.3	0.6	0.5
63	M	163	50.2	18.89	18.6	68	110	69	82.67	53	34.1	7.1	4.2	1.4	1.5	29	26	67	169	207	54	79	108	5.1	60.5	462	14.2	44.1	18	265	14.6	0.92	143	4.1	105	8.8	5.6	64	12.5	13.2	0.6	1
52	M	169.7	87.4	30.35	35.2	104.5	126	76	92.67	72	7.1	7.3	4.3	1.4	0.6	73	84	53	264	207	166	41	156	4.9	91.3	458	15.3	44.7	22.8	340	12.3	0.8	141	4.3	103	9.5	4.8	63	16	11.5	0.6	0.6
50	M	164.5	60.8	22.47	24.5	80.5	123	67	85.67	55	15.9	6.7	4.1	1.6	0.8	18	18	28	189	191	122	56	123	4.7	98.3	520	16.2	45.8	23.6	360	14.2	0.96	145	4.1	105	9.3	8.6	82	20.3	20.6	0.6	0.7
65	M	170.9	76.1	26.06	24.7	87	165	88	113.7	57	33.8	7.3	4.3	1.4	1.2	25	19	42	203	226	101	58	147	5	60.8	484	15.8	46.4	22.3	282	16	1.08	139	3.9	100	9.3	5.4	82	8.2	10.2	1.2	0.8
41	M	166.5	60.1	21.68	21.3	76.5	113	75	87.67	66	8.1	6.7	4.4	1.9	0.4	12	15	14	198	173	102	53	101	5.2	74.3	533	15.7	46.5	29.1	261	13.9	0.85	142	4.1	104	9.5	6.2	56	13.1	12.3	0.6	0.7
81	M	170.5	64.9	22.33	17.9	78	137	75	95.67	49	96.7	7.6	4.3	1.3	0.9	31	23	15	233	180	79	44	116	4.8	49.6	418	13.2	40.4	19.7	277	19.5	0.99	138	4.3	104	8.9	6	74	7.6	7	1.2	0.7
46	F	149.7	62.8	28.02	40.4	87.5	108	62	77.33	59	5.2	7.5	4.6	1.6	0.6	19	23	31	139	245	103	69	157	5.2	56.4	506	14	41	35.2	200	10.5	0.41	139	4	105	9.6	4.4	68	14.6	15.1	0.5	0.5
44	M	168.8	69.3	24.32	27.4	83	127	71	89.67	54	5.5	7.6	4.7	1.6	0.8	46	86	252	195	286	122	63	203	5.4	63	450	14.3	43.3	35.6	202	20.4	0.76	139	4.5	105	9.6	7	80	16.5	14.3	0.6	0.8
47	M	163.7	67	25	21.8	82.5	135	85	101.7	55	13.9	7.4	4.6	1.7	1	24	32	85	227	212	387	62	105	5.1	75.4	485	14.6	42.8	16.9	265	10.3	0.83	143	3.7	105	9.3	5.6	57	17.2	15.8	0.5	0.6
56	M	167	65.2	23.38	25.5	79.5	138	92	107.3	68	10.6	7	4.1	1.4	0.9	24	23	125	213	228	172	98	109	5.2	56.5	501	15.5	46	23.9	282	13	0.67	140	4.4	105	9.1	8.7	57	13.7	10.7	0.7	0.6
53	F	153.7	55	23.28	30.6	82.5	102	61	74.67	57	8.5	6.9	4.3	1.7	0.5	38	33	16	360	228	41	74	134	5.1	52.3	460	13.4	42	27.1	241	13.2	0.5	141	4.1	106	9.4	5	76	9.3	11.4	0.6	0.6
55	F	160.4	61.8	24.02	33.7	85.5	174	93	120	89	21.8	7.6	4.6	1.5	0.6	19	18	12	287	169	59	57	92	5.2	78.3	561	15.5	47.7	18.4	393	10.4	0.53	143	3.6	107	9.6	2.8	51	15.3	13.9	0.7	0.7
47	M	180.5	74.8	22.96	26.7	91	134	84	100.7	77	15.2	8.2	4.7	1.3	1	17	22	101	177	215	362	49	114	5	65.3	588	16.1	49.3	24.5	198	11.6	0.74	139	4.1	101	9.8	7.1	58	10	10.6	0.6	0.7
32	M	170.5	74.7	25.7	23.7	86.5	129	75	93	76	4	7.9	4.7	1.5	0.5	16	22	29	198	241	220	49	173	5	66.8	551	15.1	45.8	23.4	322	14.8	0.72	141	4.5	103	9.6	7	49	12.4	12.1	0.6	0.6
32	F	148.9	44.2	19.94	24.1	71.5	112	60	77.33	63	4.5	7.6	4.6	1.5	0.6	14	12	10	185	218	45	76	123	4.9	87.2	452	13	39.7	33.6	247	14.8	0.62	141	3.9	107	9.2	5	128	16.7	16.9	0.6	0.6
82	M	162.9	71.2	26.83	22.3	97	144	93	110	94	27.3	7.6	4.6	1.5	1.1	21	20	39	179	216	82	70	139	4.8	71.7	458	14.6	44.2	15.1	254	17.8	0.56	140	3.7	101	9.2	6.4	48	9.9	9.6	0.7	1.2
72	F	153.3	45.7	19.45	25	79	127	78	94.33	67	11.2	7.5	4.3	1.3	0.7	21	15	19	225	165	115	59	83	5.4	72.7	410	12.8	40.9	22.6	431	14.2	0.78	140	4.9	104	9.4	5.7	88	12.5	12.9	0.7	0.7
73	M	167.1	70.7	25.32	24.8	90.5	122	69	86.67	64	48.1	7	4.3	1.6	0.7	24	20	24	177	151	140	43	82	5.1	56.4	457	15	44.4	21.1	286	15.8	0.81	139	4.3	106	8.9	6.1	79	11.5	10.4	0.7	1.1
65	F	153.4	43.8	18.61	18.1	60	108	61	76.67	71	24.5	6.9	4.2	1.6	1.1	29	21	18	204	223	45	79	129	5.3	42.7	398	11.8	36.8	21.5	393	17	0.57	139	3.9	101	9.3	3.9	111	12.1	14	1	0.7
59	M	181.1	97.8	29.82	30.1	103	125	74	91	59	10.8	7.4	4.1	1.2	0.5	18	18	35	173	216	166	44	155	5.5	70.4	489	10.9	36.5	39.5	238	13.9	1.09	141	4.2	107	9.2	8	88	17.2	22.5	0.8	0.8
59	M	167.7	62.5	22.22	22.4	84	115	71	85.67	86	18.5	7.4	4.4	1.5	0.9	18	15	26	228	203	223	39	119	5.4	49.2	463	14.4	42.6	21.6	257	14.9	0.73	141	3.9	106	9.2	5.5	92	13.9	11.4	0.6	0.5
70	M	166.7	89.2	32.1	35.2	107	146	89	108	56	10.2	7.7	4.6	1.5	0.8	24	35	57	155	215	143	44	116	5.2	55.6	548	17.1	50.4	15.4	269	13.7	0.95	137	4	101	9.2	5.6	68	15.6	13.7	0.9	1
59	F	159.7	55.4	21.72	30.3	79.5	125	79	94.33	62	12.2	6.9	4.2	1.6	1.5	15	12	12	275	196	95	60	112	5.4	48.8	480	13.6	41.8	15.6	220	13.3	0.56	141	3.9	105	9	4.4	57	16	15.1	0.8	0.7
60	M	162.5	64.5	24.43	21.7	87	148	90	109.3	58	7.5	7.3	4.3	1.4	1	25	14	80	157	218	183	60	131	5.4	87.1	481	14.5	43.3	28.2	233	13.7	0.87	142	4.2	108	9	4.8	90	16.5	15.1	0.9	0.9
35	M	172.1	58.5	19.75	21.7	70	120	76	90.67	53	5.9	7.4	4.7	1.7	1.2	21	25	64	240	186	65	47	130	5.1	60.4	522	15.7	46.6	17.4	184	12.4	0.95	140	3.5	103	9.2	5.8	51	12.3	12	0.4	0.6
61	M	155.2	58.5	24.29	24	83.5	111	69	83	48	5.9	7.1	4.2	1.4	0.7	17	17	23	238	246	72	47	189	4.9	66.6	478	14.5	44.8	27.5	179	18	0.96	143	4.2	108	9	6.5	97	11.3	12.4	0.8	1.1
65	F	152	44.4	19.22	18.3	73	97	52	67	54	39.8	7	4.2	1.5	0.6	24	18	14	255	225	59	83	123	5.3	31.1	410	11.9	36.9	21.8	300	12.3	0.55	144	4.3	105	9.2	3.6	95	8.1	8.7	0.8	0.7
74	M	161.1	62.6	24.12	18.8	87	131	78	95.67	70	31.1	7.6	4.2	1.2	1.3	19	16	14	197	232	160	45	158	5.1	56	458	15.4	44.8	17.7	316	15.7	1.07	142	4.6	109	8.9	6.7	57	13.6	12.5	0.8	0.8
62	M	162.9	66.3	24.98	26.7	83.5	125	79	94.33	49	15.4	7.4	4.3	1.3	0.8	24	20	63	239	216	119	60	117	5.4	48.7	524	15.7	46.8	18.9	269	14.4	0.7	141	4	102	8.8	4.5	66	8.3	9.8	0.8	0.7
65	F	154.5	50.4	21.11	24.3	69.5	116	69	84.67	73	15.5	7.4	4.3	1.4	0.6	22	15	18	177	245	107	73	148	5.5	52.8	430	12.6	39.2	19	311	13.9	0.7	142	4	103	9.5	5.1	113	9.5	9.2	0.7	0.7
61	F	146.9	53.1	24.61	36.2	83.5	138	77	97.33	62	51.7	7.3	4.5	1.7	0.9	17	16	22	142	244	99	57	166	5.4	54.7	423	12.8	37.6	20.6	465	13.1	0.57	142	3.7	108	9	6	27	14.1	13.3	1	1.2
83	M	151.7	60.3	26.2	17.4	83	160	80	106.7	49	153	7.1	3.9	1.2	0.6	20	15	56	300	239	199	37	141	5.7	55.4	440	13.2	41	16.5	316	33.1	1.66	139	4.7	105	8.8	6.7	90	10.6	10.8	0.6	1.1
72	M	168.6	59.7	21	16.2	78	148	82	104	57	33.7	7.2	4.4	1.5	1.2	20	15	32	187	248	125	60	171	5.7	54.2	488	15.5	45.4	18.5	230	15.1	0.83	138	4.1	101	9	4.7	80	7.5	8.2	0.7	0.8
69	F	159.5	54.1	21.27	24.4	65.5	122	75	90.67	67	30.6	7.5	4.7	1.6	0.7	29	16	16	203	265	85	115	107	5.4	35.8	473	14.8	44	18.5	316	12.9	0.66	140	4.6	101	10	4.8	72	13	12.9	0.7	0.8

61	F	147.1	55.4	25.6	37	75.5	148	91	110	69	36.2	8	4.9	1.6	1	22	19	29	176	199	90	63	109	5.4	49.9	524	15.5	46.6	16.5	296	17.2	0.69	143	4	102	9.5	6.3	80	14.8	13.9	0.7	0.7
37	M	173.7	76.4	25.32	30.4	86.5	143	85	104.3	57	6.9	7.2	4.7	1.9	0.4	21	22	60	207	200	208	47	121	5	71.5	514	15.5	45.5	20.8	286	9.2	0.77	143	3.9	102	9.6	8.8	55	11.8	9	0.7	0.7
64	M	167.1	62.5	22.38	17.1	85.2	132	86	101.3	67	30.9	7.2	4.2	1.4	0.6	27	28	48	333	238	90	57	163	7.3	67.7	482	13.7	41.1	23.6	393	27.4	1.43	140	4.1	104	9.4	9.4	53	8.5	8	1.3	0.7
56	M	160.9	54.5	21.05	19.8	72.5	118	64	82	62	19.9	7	4.2	1.5	0.9	20	27	34	252	167	103	43	104	5.4	64.2	494	15.5	45.6	18.9	316	19.6	0.82	143	4.3	109	8.7	5.1	66	12.2	10.5	0.6	0.6
64	F	140.5	43.4	21.99	26.7	80.5	140	87	104.7	94	20.5	8.2	4.5	1.2	0.5	32	28	33	233	208	135	50	128	5	69.9	450	13.9	41.5	22.5	286	12	0.64	143	4.2	106	9.3	4.9	91	15	12.4	0.6	0.5
71	M	159.5	62.7	24.65	22.4	83.5	161	94	116.3	53	53.4	6.9	3.9	1.3	0.8	22	18	30	232	161	88	54	88	5.1	58.2	447	14.5	42.8	22.6	306	12.2	0.89	144	4.1	108	8.9	6	66	12.7	12.7	0.9	0.8
65	M	159.7	59.1	23.17	25.1	83.5	141	77	98.33	61	22.3	7.4	4.2	1.3	1.5	25	31	50	327	196	87	63	111	7.7	64	603	19.2	56.6	21.2	250	14.9	0.79	141	4.1	103	9.1	5.2	77	15.4	13.4	0.6	0.6
78	F	146.9	52.8	24.47	30.3	83.5	194	97	129.3	67	17.8	7.4	4.3	1.4	1.1	25	18	23	436	220	163	55	127	4.7	64.9	457	14.5	41.7	23	328	15.6	0.9	141	4.5	105	9.4	4.7	89	7.9	7.6	1	0.9
82	M	155	55.4	23.06	16.8	74	101	66	77.67	59	239	7.9	4.4	1.3	0.7	34	20	71	156	180	33	80	84	5.5	52	393	12.4	37	20.3	273	18.3	1.2	138	3.6	101	9.4	8.1	155	7.8	7.6	0.7	0.7
69	M	165.9	54.9	19.95	13.6	67	127	72	90.33	53	52.9	7.1	4.2	1.5	0.7	33	42	34	223	176	62	61	100	5.2	46.2	464	14	41.5	21.5	241	13.1	0.77	142	4.2	104	8.7	4.2	116	9.8	10.7	0.7	0.6
63	F	156.9	56.1	22.79	29.4	84	136	72	93.33	74	8.5	7.8	4.5	1.4	0.6	20	17	18	251	202	126	55	124	5.3	62.3	482	14.2	41.8	28.5	353	13	0.71	143	4.4	104	9.5	5.3	52	11.9	11.8	0.9	0.8
79	M	170.4	58.2	20.04	13.9	76	145	85	105	70	13.1	7.1	4.3	1.5	0.8	15	15	26	179	181	46	79	79	6.7	43	461	12.9	39	15.7	227	21.1	0.85	143	4.4	105	9	5.3	52	9.4	9.6	0.6	0.6
52	M	154.5	49	20.53	15.6	62.5	114	72	86	94	12.7	7.1	4.3	1.5	0.7	15	16	129	249	243	158	57	151	5	72.7	466	14.5	44.1	25.4	282	12.9	0.71	144	4.2	106	9.6	5.3	61	15.9	12	0.6	0.8
48	F	156.9	65.4	26.57	33.9	87.5	106	59	74.67	63	12	7.6	4.5	1.5	0.7	16	15	15	163	248	81	56	174	5.5	59.1	431	12.3	37.2	31.8	296	14.4	0.61	141	4.2	105	9.5	4	56	13.8	10.2	0.7	0.8
66	M	162.1	83.8	31.89	29.9	107	127	88	101	73	15.7	7.1	4.4	1.6	0.5	39	51	115	146	185	88	63	99	5.1	65.5	498	15.9	46.7	21	230	21.6	1.04	142	4	106	8.8	6.2	60	14.5	11.7	1.3	0.7
59	M	160.7	75.1	29.08	36	90.5	145	85	105	85	17.1	7.3	4.6	1.7	1.1	27	36	144	173	240	165	48	161	5.8	66.7	502	16.6	47.9	20.5	222	12.6	0.75	143	3.9	106	9.4	5.2	62	15.7	17.9	0.7	0.6
69	F	153.7	54.2	22.94	30.9	76.5	124	70	88	56	20.4	7.1	4.2	1.4	1.7	34	21	15	180	183	118	47	117	5.9	59	459	13.3	39.7	25.9	254	12.3	0.61	143	4.2	109	9.1	4.5	71	10.9	10.9	0.7	0.9
69	F	151.3	51.7	22.58	29.6	83	111	67	81.67	60	15	7.2	4.6	1.8	0.8	21	18	13	225	232	87	73	115	5.7	44.2	437	13.3	38.9	17.7	300	14.9	0.69	142	3.9	104	9.7	4.8	98	12.1	12.5	0.6	0.8
70	M	169.2	73.2	25.57	22.7	90.5	162	90	114	59	29.2	7.1	4	1.3	1.4	21	17	32	244	204	81	53	133	4.8	94.3	494	14.9	44.6	17.1	244	20.3	1.01	143	4	105	8.8	8.3	87	11.1	12.4	0.8	0.8
79	M	165.2	50.3	18.43	9.5	64	117	63	81	68	61.9	7.2	4.4	1.6	0.9	23	19	31	209	199	76	71	97	5.7	38.6	438	13.3	40.5	16.7	265	20.5	0.96	138	3.7	100	9.1	5.3	96	12.7	13.4	0.7	1
75	F	154.3	49.6	20.83	25.4	65	120	62	81.33	47	156	7.9	4.4	1.3	0.8	27	38	46	220	174	144	34	103	5.4	58.1	385	12.5	37.2	13.5	233	19.2	0.84	139	4	100	9.2	8.4	97	13.1	12.5	0.6	0.8
63	F	160	59.8	23.36	31.7	85.5	136	90	105.3	82	16.1	7.7	4.7	1.6	0.7	21	24	16	267	221	83	48	156	5.5	46.7	440	13.1	39.7	26.3	291	11.8	0.53	140	4	105	9.5	4.9	54	14.3	13.3	0.8	1
80	M	150.9	61	26.79	20.3	88.5	139	83	101.7	52	43	6.6	4.1	1.6	0.8	17	15	15	246	141	55	52	77	5.2	43.3	470	14.7	43.2	17.4	254	17.9	0.82	141	4.1	107	8.9	6.9	63	11.2	9.7	1.2	1.4
63	F	160.5	51.4	19.95	22.8	71	130	80	96.67	60	22.5	6.7	4.4	1.9	0.8	17	13	16	168	195	52	69	98	5.9	56.9	366	11.5	34.9	21.2	282	22.2	0.69	139	4.2	103	9.3	3.5	75	15.6	13.2	0.8	0.8
66	M	168.1	57.4	20.31	8.2	72.5	120	70	86.67	61	32.3	4.6	2.6	1.3	0.6	29	25	30	181	163	59	50	94	4.3	59	455	14	40.8	24.8	277	21.6	0.77	139	4.2	105	7.9	5.8	124	11.5	11.7	0.9	1.3
61	F	154.9	57.7	24.05	21.7	93	143	80	101	65	32.7	7	4.4	1.7	0.8	17	16	29	382	227	247	39	145	5.4	47.9	422	12.7	37.8	20	235	8.1	0.61	141	4.3	103	9.3	4.3	87	10.2	10.1	1.3	0.8
61	M	171.9	64.1	21.69	20	76.5	137	74	95	60	22.7	7.5	4.5	1.5	0.6	23	21	230	353	218	206	42	121	7	66.8	505	15.6	45.7	24.7	334	13	0.73	138	4.4	99	9.9	7.5	72	11.7	13	3.2	1.6
81	F	153	56.6	24.18	31.7	95.5	131	66	87.67	63	75.2	7.3	4.1	1.3	0.7	25	19	24	155	201	121	76	86	5.5	74.3	469	14.3	43.1	24.1	334	21.6	0.75	142	3.7	104	9.4	3.4	66	14.4	15	0.7	0.8
57	M	172.1	72.5	24.48	22.1	90.5	107	63	77.67	58	12.5	7.2	4.7	1.9	1.4	42	85	106	237	212	142	57	132	5.2	73	472	14.8	44.4	21.5	220	11.8	0.76	141	4.3	103	9.6	6	78	10.8	13.3	0.8	0.7
74	M	167	56.5	20.26	14.3	73	127	80	95.67	65	17.2	7.2	4	1.3	0.6	19	10	13	224	218	111	50	132	5.6	61.5	442	13	40.3	20.5	269	25.9	0.77	141	4	103	8.9	5	99	10.1	11.2	2.2	1
82	F	145	37.6	17.88	19.1	56.5	140	66	90.67	65	46.6	7.4	4.5	1.5	1	28	20	17	315	209	60	81	105	5.3	48.2	424	13.3	39.3	20	316	14.5	0.58	144	3.8	101	9	3	89	11	11.7	1.1	1.1
59	M	169.6	61.1	21.24	19.1	73.5	146	92	110	66	10.4	6.3	4.1	1.8	0.5	16	13	23	307	236	143	56	152	5.2	66.2	496	15.8	47.1	21.4	286	12.8	0.98	139	4	100	9	5.2	88	7.9	8.1	0.8	0.6
57	F	153.9	55.7	23.52	30.4	81	105	57	73	60	11.4	7.5	4.2	1.3	0.7	20	23	22	203	204	157	43	127	4.9	66.3	410	12	35.3	20.9	322	11.6	0.54	141	3.9	102	9.4	5.2	119	10.4	12.4	0.5	0.6
66	M	161.7	52.6	20.12	16.9	68.5	116	70	85.33	63	16	7.7	4.5	1.4	0.8	27	19	63	172	201	58	81	95	5.5	41.6	424	14.7	44.3	22.2	286	16.3	0.72	141	4.3	99	9.7	6.7	95	10.7	10.5	1.8	2.8
51	M	170.3	68.5	23.62	20.6	81	145	93	110.3	66	8.5	7.6	4.6	1.5	0.7	43	56	132	203	263	346	64	135	5.7	42.1	535	16.7	48	14.4	193	17.5	0.8	140	4.2	100	9.6	5.7	67	9.8	9.3	0.6	0.6
52	M	166.9	60.4	21.68	22.2	74	122	77	92	72	13.3	8.1	4.7	1.4	0.7	33	35	26	210	299	106	58	201	5.1	56.3	480	16.1	44.4	18.6	347	12.3	0.82	137	4.3	101	9.7	6.9	171	13.3	12.8	0.5	0.5
54	F	157.3	43.4	17.54	19.6	57.5	93	52	65.67	57	31.6	7	4.5	1.8	0.9	21	16	28	151	221	71	63	135	5.1	52.2	392	12.2	36.6	26.7	291	10.6	0.52	140	4.2	104	9.7	4	87	17.2	14.5	0.8	0.6
80	M	162.4	55.8	21.16	21.5	72	121	67	85	70	119	8.1	4.4	1.2	0.9	32	23	15	184	180	62	43	111	6	69.9	441	13.8	41.1	19	347	21.8	1.1	136	4.2	99	9.4	7.6	59	9.1	9.3	1.1	1.2
70	M	169.5	74.1	25.79	33.6	96.5	126	95	105.3	107	87.7	8	4.4	1.2	0.9	35	38	105	256	229	200	70	124	6.1	89.6	548	17.1	49.2	22	230	13.6	0.9	139	4.4	101	9.4	6.2	125	7.9	9.1	0.6	0.8
64	M	164.6	61.5	22.7	18.7	68	109	67	81	65	40.7	7.3	4.3	1.4	1.2	86	45	21	263	214	63	58	135	5.6	70.3	501	14.8	43.6	22.4	286	7.8	0.71	142	3.6	103	9.2	6.1	70	7.8	7.5	0.7	0.7
74	M	172.5	65.9	22.15	23.5	83	152	80	104	70	17.4	7.5	4.3	1.3	0.7	20	14	45	266	167	87	74	71	5.2	69.8	479	14.8	44.4	23.9	296	6.4	0.65	139	4.1	99	9.3	6.5	57	12.6	9.3	0.8	0.7
61	M	168.9	72.3	25.34	24.3	85.5	155	87	109.7	62	22.7	6.5	4.5	2.3	0.9	28	20	149	215	212	145	77	92	5.7	51.5	475	16.6	46	25	257	12.5	0.71	137	4.3	102	9.4	5.8	81	14.4	14.6	0.7	0.8
58	M	176.9	74.7	23.87	23.3	82	121	79	93	56	31.3	7.9	4.7	1.5	0.9	32	35	62	152	217	121	64	125	5.9	61.5	537	15.8	46.5	26	238	13.4	0.7	139	4.4	102	9.7	5.7	70	11	9.9	0.7	0.6
57	F	158.5	51.7	20.58	29.1	82	125	71	89	72	11.6	6.8	4.4	1.8	1.2	23	22	14	217	257	72	67	157	5.2	59.4	449	13.4	40	17.8	347	17.1	0.52	142	3.8	102	9.5	4.3	82	13.6	12.6	1.1	0.9
65	F	150.9	46	20.2	22.8	64	128	78	94.67	58	87.6	7.5	4.5	1.5	0.7	24	22	11	202	210	199	55	107	5.8	43.6	445	13.1	40.2	29.1	334	19.1	0.6	140	4.3	99	9.4	4.6	113	10.5	14.6	0.9	0.9
69	M	158.5	64.3	25.59	24.3	87.5	104	64	77.33	53	15.4	7.4	4.3	1.4	0.9	33	31	22	275	235	100	52	148	5.5	48.3	485	15.5	46.6	16.6	233	18	0.77	140	4	103	9.6	5.2	86	7.8	9	0.7	0.7
50	M	179.7	66.9	20.72	17.5	79.5	106	66	79.33	55	8.1	7.3	4.5	1.6	0.8	24	22	21	149	234	164	65	134	5.5	62.2	518	14.6	43.9	27.8	209	15.7	0.91	140	4.5	103	9.6	6.5	93	10.7	9.6	0.7	0.6
68	F	159.3	52.4	20.65	28.3	80	123	75	91	77	38.2	7.2	4	1.3	0.6	22	17	32	250	233	99	73	121	5.3	47.3	400	12.5	38.8	27.1	367	14	0.55	141	3.4	99	8.9	3.9	107	7.6	7.5	0.9	0.8
75	F	152.6	51.7	22.2	27.9	86	154	81	105.3	62	38.5	8.3	4.8	1.4	1	28	22	20	242	247	167	63	153	5.8	63.7	446	13.7	41.1	26.8	334	19.6	0.81	141	4.1	96	9.9	7	69	11.2	10.5	1.1	1.1
59	F	160.1	55.9	21.81	31.1	79.5	126	77	93.33	71	14.1	7.3	4.4	1.5	0.8	17	10	15	238	203	79	75	91	5.5	32.4	498	13.2	40.5	13.6	238	16.6	0.54	142	3.7	102	8.8	1.9	149	12.1	10.4	0.6	0.5
67	F	150.5	46.3	20.44	22.7	62	92	54	66.67	72	86.3	6.9	4.1	1.5	1.1	31	18	93	249	236	80	80	113	6.2	61.3	376	11.1	34.5	25.6	296	17.1	0.63	141	3.7	102	8.8	5.9	72	13.3	12.1	0.6	0.7
50	M	170.8	69.9	23.96	26.4	77.5	124	79	94	61	9.3	8	4.8	1.5	1.2	21	19	38	118	257	109	57	166	5.2	61.6	481	16	47.1	22.9	244	15.1	0.92	138	4	97	9.6	5.4	64	7.4	7.5	0.6	1.5
74	M	159.7	48.1	18.86	15.1	72.5	140	85	103.3	83	48.8	7.5	4.3	1.3	1.2	51	27	157	163	159	240	62	55	6.3	54.9	382	13.1	39	12.8	207	13.6	0.53	139	4	100	8.7	4.4	83	11.5	8.5	1.2	1.8
65	F	145.6	43.2	20.38	24.3	64.5	132	78	96	53	20.5	7.3	4.4	1.5	0.5	25	27	23	379	180	67	63	87	5.6	34.3	438	13.4	41.6	17.2	262	14.3	0.62	142	4	104	11.6	6.1	53	12.4	14.8	0.7	0.8
79	M	159.3	71.9	28.33	27.5	92	123	69	87	60	663	7.8	4	1.1	0.8	32	35	67	356	179	94	40	115	5.6	63.3	422	12.8	39.3	23.9	318	29.2	1.63	142	4.5	103	9	6.3	140	7.4	8	0.8	1.2
78	M	167.6	61.5	21.89	23.5	84	123	77	92.33	57	12.6	7.7	4.3	1.3	0.9	24	24	55	192	225	128	51	146	5.6	70.7	522	16.2	48.3	33.6	235	13.6	0.85	139	4.2	100	9.5	6	127	8.7	9.5	0.9	0.7
56	F	162.1	46.8	17.81	18.6	65	112	73	86	61	15.4	7.1	4.6	1.8	0.8	30	22	101	176	237	40	124	82	4.9	43.9	457	14.9	43.6	25.7	213	10.4	0.62	141	4	99	9.2	5.1	52	10.8	11.5	0.7	0.6
61	F	158.1	62.2	24.88	32.3	85	132	70	90.67	69	50.1	7.3	4.5	1.6	0.6	38	62	27	212	255	96	54	170	6.7	43	437	14	42	24.8	254	13.1	0.44	142	4.5	103	9.7	6.5	45	16.8	15.2	0.7	1
69	M	167.3	70.6	25.22	23.9	93	137	91	106.3	72	23.6	7.8	4.7	1.5	1.3	21	17	37	287	185	78	63	98	5.9	60.7	524	15.8	46.9	19.6	276	11.2	0.73	141	4.2	100	9.6	4.4	55	12.2	13.9	1	0.9

73	F	158.4	72.9	29.05	44.5	98	141	74	96.33	72	11.2	7.8	4.4	1.3	0.6	23	26	31	309	270	130	63	186	5.5	102.8	419	12.9	39.2	38.1	318	17.2	0.56	141	4	102	9.5	6	87	13.5	12.5	0.8	0.8
72	F	146.6	58.6	27.27	36.7	86	124	49	74	85	146	7	3.9	1.3	0.5	21	17	26	271	223	41	62	134	5.7	52.4	392	13.1	38.7	20.9	324	17.5	0.38	142	3.9	105	8.9	3.5	71	12.1	11.5	0.8	1
61	M	161.7	61.9	23.67	23.4	80.5	123	76	91.67	56	12.7	7.2	4.4	1.6	0.5	24	23	71	246	207	100	55	124	5.3	61.5	491	15.9	46.6	19.1	244	16.1	0.82	140	4.2	99	9.6	6.3	46	12.2	11.7	0.6	0.7
63	M	161.7	60.4	23.1	21.6	78.5	155	85	108.3	60	18.2	7.5	4.8	1.8	0.6	22	20	77	149	219	93	64	123	5.9	93.9	474	15	45	30.5	318	15	0.65	138	4.5	99	9.6	5.8	64	13	12.5	0.8	1.8
69	M	162.1	49.2	18.72	10.8	63.5	126	69	88	61	43.3	7.3	4.7	1.8	1.7	65	47	24	202	247	115	76	129	6.2	45.8	439	14.3	42.1	14.4	345	13.7	0.72	137	4	96	9.4	3.1	100	14	18.1	0.6	1.6
70	F	142.9	46.4	22.72	28.2	72	147	75	99	59	339	8	4	1	1.3	52	46	122	165	189	104	59	110	5.5	75.8	516	15.8	48.1	16	254	16.1	0.77	142	4.2	104	9.4	5.9	118	15.8	16.8	0.9	1
76	M	159	51	20.17	20.1	68	125	67	86.33	83	17.2	7.8	4.5	1.4	0.7	37	34	25	273	203	125	60	117	4.8	54.6	478	14.6	44.2	20.9	258	16.4	0.37	140	4.2	99	9	5.6	110	12	11.5	1.1	1.7
72	F	150.8	36.9	16.23	16.4	59	126	67	86.67	91	62.6	7.7	4.5	1.4	1	20	21	36	293	220	94	79	116	9.9	79	497	15	43.5	24.4	290	24.3	0.45	139	4.5	96	9.7	3.2	88	15.7	14.8	1.7	1.9
72	M	158.9	49.9	19.76	19.6	74	156	90	112	72	11	7.3	4.2	1.4	1.1	26	38	40	174	187	80	53	110	5.6	58.3	479	15.7	46	20.9	285	14.6	0.68	139	4.3	102	9	5.3	128	9.2	10.6	0.8	1.3
37	M	168.4	60.2	21.23	21.4	75.5	108	65	79.33	61	11.4	7.5	4.5	1.5	0.7	25	34	32	150	212	225	45	140	5.2	60.4	462	14	41.4	26.1	224	17.6	0.88	140	3.9	103	9.4	6.2	54	12.1	14.7	0.6	0.7
57	F	162.3	56.7	21.53	28.8	84	120	75	90	78	32.7	7.8	4.5	1.4	0.7	19	16	13	252	229	140	60	138	5	54.1	477	14.3	42	19.7	345	11.8	0.62	141	4	100	9.4	4.6	116	12.6	8.9	0.6	0.6
56	M	171.3	71.8	24.47	21.9	85.5	131	88	102.3	77	11.1	7.3	4.5	1.6	0.8	29	25	57	287	210	88	71	118	5.3	71.4	539	16.1	49.8	21.5	285	11	0.61	139	3.8	98	9.2	5.2	101	10.5	8.5	0.7	0.5
53	F	156.7	49.9	20.32	25.9	66.5	137	87	103.7	63	11.7	7.6	4.3	1.3	0.7	22	16	18	213	250	88	66	152	5.2	66.7	460	13.7	42.7	25.4	280	13.1	0.57	140	3.8	102	9.4	3.4	63	16.5	15.6	1.1	0.9
45	M	170.1	73.4	25.37	27.2	85	115	71	85.67	61	5.3	7.6	4.4	1.4	0.7	21	42	76	257	252	282	49	181	5.5	94.9	473	14.6	43.9	36.7	276	10	0.74	138	4.2	99	9.3	4.2	43	14.7	16.4	0.8	0.6
43	F	155.9	56.1	23.08	35.4	70.5	102	62	75.33	65	11	7.6	4.6	1.5	0.5	12	8	12	223	200	52	88	89	4.7	49.9	489	9.6	32.7	37.8	267	13	0.51	138	4	102	9.6	3.2	63	13.7	12.7	0.6	0.7
78	F	147.5	50	22.98	29.3	78.5	131	76	94.33	64	56.3	7.6	4.8	1.7	0.5	36	44	52	332	180	84	80	67	5.4	45.1	441	14.5	42.6	20.6	250	12.7	0.52	143	3.8	103	9.4	5.1	97	12.2	10.1	1.1	1.1
69	F	146.1	48.2	22.58	26.5	85.5	131	73	92.33	69	17	7.2	4.3	1.5	0.5	14	10	37	177	204	83	61	121	5.2	51.8	446	12.7	38.6	21.9	307	15	0.48	143	4.1	104	9	6	56	15.3	12.9	0.6	1.3
62	F	144.5	40	19.16	22.3	62	156	80	105.3	59	102	7.6	4.6	1.5	0.7	26	21	27	178	269	47	81	152	5.4	37.6	465	14	42.6	20.3	238	18.1	0.66	142	3.6	101	9	5.3	70	13.5	16.3	1.1	1.5
61	F	156.5	56.5	23.07	30.1	79	128	73	91.33	58	34.4	7.1	4.6	1.8	0.5	19	18	33	152	242	115	65	141	5	64.2	428	13.9	42.4	29	307	14.1	0.58	143	4.7	103	9.6	5.8	66	17	17	0.9	1
57	F	148.6	41.3	18.7	22.5	59	147	87	107	62	16.1	7.3	4.1	1.3	1.4	32	46	60	273	206	65	80	104	4.9	43.3	422	13.2	39.3	22.3	285	15.9	0.61	139	3.7	100	9.1	5.2	119	12.8	12	0.7	0.7
66	F	152.1	41.8	18.07	21.9	61.5	128	81	96.67	63	95.9	7.5	4.6	1.6	0.6	20	14	20	276	264	104	66	171	5.4	53.7	467	14.2	44	21.8	390	15.5	0.49	145	4.3	102	9.4	4.7	110	14.9	16	1	0.8
55	M	164.5	70.6	26.09	28.6	90.5	109	70	83	72	17.5	7.6	4.5	1.5	0.5	39	67	79	355	259	241	58	171	5.7	64.7	517	16.2	46.1	21.9	262	18.8	0.68	140	4.2	101	9	6.4	62	16.3	15.6	0.5	0.7
82	F	146.5	42	19.57	20.9	69	121	63	82.33	82	63.21	7.3	4.1	1.3	0.5	24	15	17	220	180	81	53	102	7.3	55.9	357	10.3	33	21.7	440	13.4	0.6	143	5	104	9.2	3.9	88	9.1	8.5	3.7	1.1
71	F	150.1	46.8	20.77	26.2	69.5	112	71	84.67	70	13.5	6.9	4.2	1.6	0.9	27	18	12	238	247	52	82	138	5.2	52	409	13	38.6	22.8	375	13.7	0.49	142	3.9	104	9.1	3.5	84	17.4	18.4	0.8	0.9
90	M	164.8	48	17.67	14.4	64	127	67	87	65	112	7.1	4.3	1.5	0.7	44	24	32	201	155	79	52	82	5.4	43.1	410	12.2	38.3	12.8	301	20.4	0.89	140	4.8	96	8.8	4.9	154	7.8	8.1	1.3	0.9
60	F	146.8	47.1	21.86	28.4	73.5	115	72	86.33	63	10.3	6.9	4.2	1.6	0.5	23	13	17	328	214	86	70	107	5.5	58	443	13.7	41.8	20.3	312	16.7	0.63	144	3.8	102	9.1	4.2	88	14.9	12.9	0.6	0.7
68	M	156.9	74.4	30.22	31.3	92	138	83	101.3	53	13	7.7	4	1.1	0.8	51	20	27	347	170	48	49	97	5.4	75.4	485	15.2	45.3	27.1	324	15.1	0.68	143	4.3	103	9	4.9	150	13.4	18.5	0.9	1.1
64	F	154.2	56.8	23.89	34	85.5	127	75	92.33	69	15.8	7.7	4.3	1.3	0.7	73	85	30	337	185	62	57	97	5.2	75.4	460	13.4	41.3	14.9	250	20.2	0.63	146	5.6	106	9.6	6.8	80	13.3	14.3	0.8	0.7
66	M	158.8	65.7	26.05	24.9	79	122	71	88	64	13.5	7.8	4.5	1.4	0.5	31	24	154	169	208	103	64	123	5.7	73.9	490	14.7	44.9	21.4	312	15	0.95	143	4.4	102	9.5	6.7	49	14.7	14.9	1.7	1.5
56	F	153.7	38.7	16.38	16.7	72	94	61	72	61	15.6	7.9	4.4	1.3	0.5	33	24	12	288	187	45	82	88	5.8	72.9	443	12.6	39	35	359	13.3	0.52	142	4.8	101	9.4	4.7	131	13.5	12.3	0.7	0.7
77	M	163.5	56.7	21.21	18.9	79	135	70	91.67	61	17.8	7	4.2	1.5	0.8	17	8	13	218	118	56	43	57	5.7	51.5	426	13.1	40	17.9	318	14	0.7	143	3.7	100	8.8	4.9	119	9.8	11.9	1.4	0.7
64	F	146	56	26.27	33.4	83.5	136	87	103.3	117	18.1	7.7	4.6	1.5	0.7	26	18	17	352	218	84	73	114	5.5	52.1	459	13.3	41.2	13.8	312	11.2	0.55	142	3.9	102	9.6	3.3	90	14.5	14	0.6	0.7
47	M	168.9	64.3	22.54	20.8	79.5	121	77	91.67	41	7.6	7	4.3	1.6	0.9	18	16	25	164	183	55	85	80	4.7	29.2	456	14.4	42	20.1	218	9.7	0.6	139	4.1	99	9.3	4.4	78	8	10.2	0.6	0.7
55	M	176.4	73	23.46	25.6	87	115	77	89.67	61	9.2	7.1	4.1	1.4	0.6	28	36	64	237	199	55	60	13	5.1	43.4	466	14.8	44.9	17.6	301	18.6	0.93	142	3.4	100	9.3	8.4	132	14.3	13.9	0.6	0.7
54	F	141.3	41.7	20.89	29.1	71.5	97	57	70.33	65	5.6	7.3	4.3	1.4	0.8	31	33	12	348	297	77	83	176	4.8	46	420	13	39.5	25.4	367	13.6	0.57	140	3.9	99	9.2	3.9	148	15.2	12.1	0.5	0.7
60	M	163.4	78.8	29.51	35.6	101	146	81	102.7	72	76.5	7.1	4.5	1.7	0.8	25	23	60	183	228	249	50	144	6.2	79.5	517	15.4	46.5	33.6	290	17.5	0.81	139	4	99	9.3	5	47	17.7	14.3	0.6	0.6
58	F	147	42.5	19.67	20.3	58.5	106	73	84	57	19	6.9	4.5	1.9	0.4	15	10	12	157	218	61	78	113	5.3	42.2	365	11.9	36.1	24.1	271	23.6	0.51	142	4.2	104	9.4	3.9	73	14.6	13.7	0.6	0.7
62	M	178.8	59	18.46	14.5	69	156	89	111.3	69	16.8	7.2	4.4	1.6	1.1	15	5	22	183	218	89	55	137	5.3	40	487	14.9	45	20.4	296	17.8	1.06	143	4	102	9.4	7.9	122	10.5	10.7	0.8	1
73	F	155.5	49.8	20.6	25.3	78.5	124	76	92	59	44.3	7.1	4.2	1.4	0.6	28	14	9	170	269	79	81	155	5.2	47.7	402	12.2	36.9	23.2	367	16.6	0.85	141	3.8	101	8.9	3.9	92	14.7	15.5	0.6	0.8
68	M	164.7	66.9	24.66	26.1	86.5	124	80	94.67	55	13.4	7.7	4.4	1.3	0.6	38	31	27	167	202	139	66	107	5.5	71.1	509	15.9	48.5	13.5	324	11.3	0.72	143	4.2	100	9.3	5.6	111	8.1	8.5	0.8	1.1
64	F	147.8	53.1	24.31	35.1	86.5	100	62	74.67	61	18.8	7.3	4.4	1.5	0.8	64	97	39	517	190	63	79	90	11	94.3	509	15.3	45.3	18.7	338	17.1	0.55	140	4	97	9.6	2.9	80	17	19.1	0.8	0.7
61	M	163.1	62.3	23.42	23.7	82.5	154	94	114	58	15.7	7.5	4.5	1.5	1.4	23	20	171	323	214	137	67	116	5.1	63.5	470	15.3	45.3	20	280	17.8	0.92	144	4.9	103	9.8	6.8	102	11.4	11.2	0.7	1
62	F	146.9	50.1	23.22	29.3	76.5	127	68	87.67	61	7.5	7.1	4.3	1.5	0.6	20	21	34	244	229	61	71	145	6.3	47.2	451	14	42.1	27	247	14.1	0.62	143	3.8	103	9	5.2	79	12.5	13.9	0.8	1.5
59	F	158.9	52.9	20.95	28.4	73	134	83	100	58	9.4	6.9	4.3	1.7	0.7	19	13	13	236	195	52	53	123	5.5	48.8	461	13.2	40.9	26.6	238	16.4	0.65	144	4.4	104	9.3	3.5	61	10.3	12.6	1.5	1
59	M	181.2	89	27.11	30.2	102	145	85	105	83	16.4	7.3	4.1	1.3	2.3	29	25	92	162	243	78	72	156	4.6	41.7	490	16.6	47.1	12.2	226	7.3	0.6	142	3.2	99	8.7	4.7	63	15.7	16.6	0.8	0.9
56	F	159.1	53.9	21.29	29.5	83.5	125	76	92.33	67	6.7	7.2	4.3	1.5	0.9	28	37	59	331	204	180	47	125	6.7	47.5	444	13.6	41.2	26.9	276	15	0.56	143	3.8	102	9.4	4.9	58	10.4	14.1	0.6	1.3
63	M	169.8	75	26.01	22.2	85	134	75	94.67	59	11.7	7.3	4.2	1.4	1.4	21	21	23	114	166	78	67	81	5.9	51.4	406	12.7	39.1	26.6	250	15.8	0.77	143	4.3	103	8.6	5.6	89	7.9	7.8	0.8	0.9
56	F	161.3	91	34.98	45.7	102.5	118	68	84.67	68	110	6.8	4.1	1.5	0.6	21	23	17	190	206	166	51	134	6.5	116.9	545	15.2	47.7	22.1	318	14.2	0.71	142	4.1	100	9.1	6.4	64	13.1	14.5	0.6	0.8
60	M	160.7	70	27.11	28.4	91	147	85	105.7	50	20.3	7.5	4.3	1.3	1	23	24	32	343	218	103	65	140	6.1	73.1	483	15.4	46	19.1	241	17.9	0.77	141	4	102	8.8	5	88	13.8	14.4	0.8	0.9
61	M	160.9	68.4	26.42	28.5	87.5	137	80	99	64	14.7	6.8	4.3	1.7	0.6	31	41	36	267	193	159	42	130	5.3	79.1	509	15.4	47.1	23.7	318	15.3	1.04	141	3.8	102	9.2	5.1	81	14	11.6	0.8	1.1
60	F	146.8	39.1	18.14	19.1	58.5	96	59	71.33	61	11.6	7	4.1	1.4	0.5	29	20	24	457	185	46	84	75	5.5	56.1	476	14.8	43.9	21.4	244	15.1	0.53	140	4	100	8.9	3.7	67	11.3	11	0.6	0.8
67	M	159.7	59.1	23.17	20	80.5	144	76	98.67	84	8.9	7.2	4.2	1.4	0.4	30	19	110	135	251	386	50	143	5.2	44.7	449	14.3	43.5	21.8	276	12.5	0.6	142	3.9	101	9	5.5	91	14.9	15.4	3	2.6
55	F	152.5	59.1	25.41	34.4	93	149	78	101.7	92	11.4	7.4	4.3	1.4	0.8	24	25	18	287	255	57	82	153	5.3	67.3	460	13.6	43	22	324	17.4	0.48	143	3.7	103	9	3.9	93	14.5	15.7	0.6	0.8
50	F	174.5	77.7	25.52	34.6	90.5	112	73	86	63	10.4	7.2	4.5	1.7	0.7	19	14	11	251	207	87	59	113	5.2	39.5	489	14.4	44.1	15.6	218	12.1	0.65	141	4.1	100	9.6	4.4	78	12.4	9.4	0.7	0.7
66	M	163.7	60.1	22.43	23.2	81	136	92	106.7	85	10	8.1	4.4	1.2	0.5	27	41	56	273	255	178	61	158	6.4	53	546	17	52.3	22.9	250	17.3	0.75	141	4	100	9.1	5.1	82	14.9	14.6	0.8	0.7
61	F	153.1	60.2	25.68	35.5	92.5	123	69	87	61	11	8.1	4.2	1.1	0.7	28	23	24	289	210	78	60	123	5.9	42.2	431	13.5	41	24.3	331	12.1	0.6	142	4	103	9.2	4	79	11.3	12	0.7	0.8
61	M	174.5	77.2	25.35	29.4	94.5	149	90	109.7	62	5.4	7.7	4.5	1.4	0.6	26	22	44	199	249	134	62	166	5.4	47.7	495	15.4	46.8	21.7	301	9.7	0.73	140	4	100	9.6	6.3	73	15.5	17.4	0.7	0.7
57	F	153.5	48.8	20.71	26.7	82	135	73	93.67	60	25.8	7.7	4.5	1.4	0.6	26	22	14	309	232	132	62	132	5.4	62.9	428	13.4	40.3	18.8	276	12.1	0.49	142	4.1	100	9.3	3.6	105	14.9	15.4	0.6	0.6
51	F	156.5	60.2	24.58	37.5	85.5	100	58	72	53	9.7	7.1	4.2	1.4	1.2	22	21	33	290	241	61	72	152	5.2	43.1	496	14.8	45.6	28.3	367	10.7	0.6	143	4.4	104	9.6	4.9	70	10.3	9.1	0.8	0.6

61	F	151.7	46.6	20.25	25.1	72	140	72	94.67	91	11	7.1	4.4	1.6	0.5	22	10	16	173	224	110	67	128	5.8	53.8	465	13.4	42	25.3	301	13.1	0.5	142	4.3	102	9.6	4.6	96	14.1	13.9	0.9	1
67	M	166.9	60.1	21.58	18.3	74	162	97	118.7	62	16.5	7.1	4.3	1.5	0.5	18	12	35	233	199	107	59	108	5.5	72.2	477	14.9	45	18.7	301	14.9	0.73	142	4.3	102	9.3	4.9	96	13.7	15	0.7	0.8
67	F	144.9	48.5	23.1	29.5	81.5	125	72	89.67	93	28.1	7.6	4.6	1.5	0.7	18	18	22	278	194	147	51	117	6.6	73.4	486	14.2	44.1	19.9	301	13.6	0.68	143	4.1	101	9.4	4.6	103	19.5	16.7	1.2	1.3
57	M	156.3	56.3	23.05	20.9	76	150	94	112.7	51	10.3	7	4.3	1.6	0.8	19	12	37	160	198	126	54	127	5.6	73	508	15.2	47	22.3	271	17.3	0.84	139	3.9	97	9.4	5.3	85	13.8	11.2	0.7	0.8
75	M	162.6	59.1	22.35	17.7	78	142	78	99.33	51	45.8	7.9	4.7	1.5	0.9	17	11	25	162	195	67	66	102	5.4	49.1	449	13.9	42.6	14.4	440	15.1	0.79	142	4	100	9.8	6.6	80	12.4	11.9	2.5	1.9
67	M	153.8	54.5	23.04	20.5	77.5	149	87	107.7	56	9.7	7.3	4.2	1.4	0.5	24	17	26	267	223	136	49	143	5.1	69.1	473	15.9	48.7	23.8	290	17	0.97	144	3.7	104	9.5	6.6	92	10.4	12.5	0.7	0.7
68	F	155.7	53.8	22.19	25.7	77.5	176	80	112	68	20.5	7.3	4.3	1.4	0.8	20	15	28	253	247	68	68	164	5.2	65.6	411	12.8	39.7	25.5	280	23.9	0.53	142	3.9	101	9.6	2.8	101	17.8	15.8	1.3	1.4
64	F	149.5	45.1	20.18	24.3	62	116	73	87.33	80	44.2	7.6	4.5	1.5	0.7	20	15	12	195	191	61	62	103	5.3	65.3	436	13.8	41	21.4	352	11.4	0.51	142	3.5	100	9.5	4.2	64	10.6	10.6	0.7	0.6
63	F	155.3	51.4	21.31	27.4	89	109	54	72.33	63	22.4	7	4.3	1.6	0.6	23	22	22	118	218	62	56	144	5.3	46	426	12.5	38.3	23.5	254	16.9	0.65	142	3.9	103	9.3	4.6	82	8.7	9.9	0.7	0.7
54	M	164.4	65	24.05	24.4	82.3	130	85	100	53	7.3	6.9	4.3	1.7	0.2	26	23	114	201	209	801	34	60	5.6	67.3	472	14.6	43.7	29.7	262	15.5	0.87	143	4.2	102	9.7	6.2	62	10.6	10.6	0.6	0.6
70	M	166.5	54.2	19.55	17.2	74.5	151	89	109.7	60	10.7	7.3	4.5	1.6	0.8	29	23	147	229	160	105	44	83	5.5	48.9	449	13.5	40.3	22.6	216	25	1.14	143	4.5	104	9.4	7.8	95	9.5	9.5	0.7	0.8
77	F	148.3	50.9	23.14	26.7	90.5	125	72	89.67	77	9.1	7.7	4.4	1.3	0.6	23	15	17	367	186	96	58	99	5.3	64.2	459	13.9	43.1	22.8	307	7.5	0.53	140	3.4	98	9.4	3.6	86	9.3	7.8	0.9	0.6
83	F	149.7	48.2	21.51	28.7	85.5	131	70	90.33	69	38.6	8.7	5.1	1.4	0.9	34	21	28	186	200	80	62	113	5.4	67.7	461	14.2	43	23.7	307	19.1	0.61	138	4.1	97	10.1	4.6	72	10.6	11.3	0.8	0.7
66	F	156.1	57.7	23.68	27.6	69.5	128	73	91.33	62	12.3	7.3	4.5	1.6	1.3	17	15	30	169	228	113	75	125	5.5	47.6	473	14.8	45	20.4	188	12.7	0.58	143	4	101	9.9	4.1	78	17.3	13.7	0.7	0.7
66	F	157.2	50.6	20.48	23.1	76	123	77	92.33	53	38.3	6.9	4.3	1.7	0.5	23	17	22	265	199	114	67	100	5.1	55.9	461	14.1	42.3	13.3	276	11.7	0.59	144	3.8	103	9.5	4.6	97	12.5	12.5	0.7	0.6
67	M	166	63.7	23.12	22.9	83	132	78	96	43	5.9	7.6	4.2	1.2	0.8	24	18	23	172	171	102	39	95	6	49.9	482	12.9	41.2	17.1	202	18.6	0.76	142	3.7	102	9.3	7.3	85	13.6	12.6	0.6	0.7
43	F	169.7	54.3	18.86	22.9	69.5	92	57	68.67	50	6.8	8.1	4.6	1.3	1	18	9	10	135	228	44	74	126	5.5	49	449	13.8	41.9	21.4	235	7.1	0.55	141	4.5	99	9.6	2	74	11.5	13.4	0.5	0.5
69	F	159.7	63	24.7	34.8	81.5	145	76	99	66	13.8	7.3	4.3	1.4	0.5	22	19	15	188	235	100	64	136	5.7	69.7	489	13.3	41.9	27.1	347	13.1	0.52	142	3.8	103	9.3	5.3	62	11.5	16.5	0.9	1
71	M	176.5	67.8	21.76	22.9	83	118	76	90	66	7.3	7.2	4.1	1.3	0.6	19	16	26	221	200	122	42	145	5.4	82.4	503	15.6	47.3	22	446	14.2	0.75	142	4.1	101	9.2	5.2	69	8	9.4	0.6	0.8
68	F	150.5	52.5	23.18	31	83.5	122	67	85.33	74	21.3	7.9	4.7	1.5	0.5	26	24	21	219	199	301	46	113	5.7	67.8	512	14.9	46.4	23.5	328	13.8	0.56	141	4.2	100	9.7	6	66	13	11.4	0.6	0.6
71	F	153	54.4	23.24	36.9	92.5	112	56	74.67	63	16.5	7.6	4.2	1.2	0.6	21	16	33	315	187	130	52	101	5.7	69.6	452	13.5	41.5	19.8	334	15.5	0.55	140	3.9	100	9.4	4.9	81	11.9	12	0.6	1
60	M	169.6	83.4	28.99	32.7	94.5	115	72	86.33	64	8.3	7.5	4.4	1.4	0.5	15	20	55	245	223	115	48	143	5.8	59.3	477	15.2	47.2	20.7	291	21	0.8	142	4	103	9.6	6.7	42	10.4	9.6	0.9	0.9
68	F	152.5	50.4	21.67	28.7	72.5	127	68	87.67	63	33.6	7.4	4.5	1.6	0.8	30	21	14	183	226	80	101	93	5.1	32.8	439	13.6	41.8	12.6	322	18.4	0.53	142	4	103	9.4	5.1	70	10.4	10.2	0.8	0.8
65	F	152.9	55.3	23.65	28	81	124	69	87.33	56	30.8	7	4.1	1.4	1	24	29	31	269	212	53	67	118	5	41.7	467	14	42.8	26.3	244	16.2	0.64	139	4	102	9	3.9	100	9.4	9.9	0.7	0.8
66	F	155.7	55.5	22.89	29.1	85.5	107	60	75.67	54	18.9	7.6	4.4	1.4	0.7	27	21	22	209	211	96	43	135	5.3	55.9	440	13.9	42.9	16.7	247	13.4	0.52	140	3.8	101	9.4	6.1	91	10	11.4	1	1
28	M	176.1	68	21.93	23.5	75.5	127	69	88.33	72	4.9	8.1	5.1	1.7	0.8	21	16	34	195	216	120	56	138	5	82.2	553	16	48.4	20.8	291	13.1	0.76	140	3.6	98	10	6.3	58	15.9	17.2	0.5	0.5
75	F	150.5	60.2	26.58	35	92	140	77	98	67	20.3	7.4	4.4	1.5	0.4	20	16	16	258	211	63	57	129	5.6	55.5	441	12.7	39.4	17.8	375	13.2	0.49	144	4.1	104	9.2	4.5	135	13.2	13.8	0.8	0.6
75	F	149	44.2	19.91	23.2	70.5	134	70	91.33	63	39.7	7	4.1	1.4	0.4	15	16	39	230	164	91	42	95	6.6	69.8	455	11.9	37.9	22	247	15.5	0.55	140	4	104	9	5.7	51	22.8	19.6	0.8	1.2
48	F	157.1	50.5	20.46	22.4	64	103	55	71	72	8.7	6.9	4.2	1.6	1.7	17	13	12	126	187	42	70	93	5	53.2	411	12.1	38	20.8	212	14.5	0.58	138	3.9	104	8.9	4.2	81	18.6	19.3	0.6	0.6
79	M	177.8	72.8	23.03	21.2	89	144	84	104	55	16	6.8	4.1	1.5	1.1	18	11	18	263	186	153	36	123	5.1	45.3	424	13.6	41.4	16.9	250	12.6	0.89	139	3.8	106	9	5.5	112	9.1	7.8	0.9	1.1
76	M	172.1	51.8	17.49	16.4	77	127	79	95	100	12.5	7.3	4.4	1.5	1.2	48	30	76	249	232	47	115	89	4.9	49.4	454	15	43.5	28	277	9.4	0.66	140	3.8	101	9.3	5.7	52	9.8	11.9	0.7	0.6
76	F	138.7	41.9	21.78	26	87	128	71	90	71	50.1	7.2	4.2	1.4	0.4	31	19	15	255	203	111	53	117	5.5	47	465	13.1	41.7	17.5	416	11.3	0.55	143	3.7	104	9.2	3.8	69	14.4	15.4	0.7	0.6
86	F	146.3	51	23.83	26.5	80.5	153	76	101.7	71	36.5	7.6	4.5	1.5	0.4	24	25	22	127	165	216	48	76	5.8	73.5	422	13.6	39.4	17.6	217	21.2	0.9	140	4	103	9.3	5.5	77	10.8	12.3	0.6	0.9
70	M	167.7	79.2	28.16	35	96.5	127	65	85.67	51	7.2	7.5	4.2	1.3	1.7	21	17	19	192	175	92	51	93	4.9	70.5	477	15.6	46.4	19.9	322	20.1	0.98	139	4.7	102	9.7	6.2	65	13.1	11.9	0.6	1.2
66	M	170.2	67.9	23.44	22.4	85.8	141	89	106.3	51	42	7.2	4.3	1.5	0.5	20	14	11	158	204	122	50	115	5.2	51.5	488	13.7	42.3	23.2	257	13.8	0.84	140	3.9	101	9.3	5.3	70	17.8	15.3	0.6	0.9
65	F	156.2	54.6	22.38	27	87	134	84	100.7	69	37.4	6.7	4.2	1.7	0.6	26	26	29	208	217	110	58	130	5.4	43.6	414	12.9	39.5	24.1	316	15.2	0.68	141	4.1	104	9.1	4.2	140	11.1	13.2	0.6	0.8
57	F	150.2	43.4	19.24	22.1	74.5	132	81	98	68	17.5	7.6	4.1	1.2	0.5	23	14	12	279	197	62	62	105	5	56.8	440	13.2	41.8	23.1	334	15.7	0.62	143	4.3	106	9.2	3.9	121	15.2	13.6	0.8	0.9
82	M	156.3	71.4	29.23	29.4	97.5	115	63	80.33	91	25.1	7.3	4.3	1.4	0.7	27	30	35	337	237	146	53	167	5.3	97	474	14.7	45.6	26.3	296	14.6	0.82	139	4	103	9.2	5.2	69	10.2	12.4	0.9	0.8
74	M	162.1	73.2	27.86	23.5	99.5	122	72	88.67	81	20.2	7.5	4.2	1.3	0.6	25	23	36	299	184	94	42	119	5.3	55.6	467	13.8	42.8	22.5	340	13.2	0.63	139	3.8	102	9	4.8	100	8.3	7.7	0.9	0.8
79	M	164.9	50.5	18.57	15.4	69.7	141	84	103	83	27.1	7.6	4.3	1.36	0.3	30	21	18	280	153	45	70	60	7.8	73.4	425	13.6	41.7	24.7	431	10.5	0.68	138	4.2	98	9.3	4	131	12.1	12.1	1	0.8
69	M	160.1	57.5	22.43	19	81.5	140	81	100.7	71	28.2	7.2	4.5	1.7	1.9	27	30	42	198	186	90	86	72	4.7	39.2	364	13.4	39.5	13	247	11.4	0.59	138	3.7	100	9.1	3.5	44	8.4	8.2	0.6	1.8
66	M	160	51.9	20.27	15.3	77.3	151	82	105	53	17.1	7.1	4.5	1.7	0.8	33	23	124	128	244	193	74	125	5.2	45	417	14.7	43.1	20	322	24.5	0.68	143	4	104	9.2	6	60	12.8	12.4	0.8	0.7
61	M	160.8	66.4	25.68	25.1	91.3	136	79	98	83	11.4	7.8	4.5	1.4	0.6	58	51	170	262	186	116	55	115	5.7	69.5	452	15	44.6	18.2	291	11.5	0.72	143	4.5	105	9.6	4.5	56	13.2	16	1.8	1.6
62	M	170.9	62	21.23	18.6	89.5	114	64	80.67	59	22.9	7	4.6	1.9	0.8	17	11	30	274	230	144	53	144	5.3	52.4	422	14.1	41.5	27.6	286	11.5	0.64	143	4	104	9.7	6.3	99	14.1	12.1	0.8	0.7
76	M	163.3	54	20.25	11.7	74.5	121	73	89	43	36.1	7.5	4.3	1.3	0.5	17	10	48	337	232	61	72	141	5.4	92.3	460	13.2	40.8	26.8	416	11.2	0.94	141	4	100	9.3	4.3	82	9.1	8.7	0.7	0.7
65	F	155.3	58.2	24.13	24.5	94	130	74	92.67	59	29.2	7	4.2	1.5	0.4	26	18	11	151	141	73	56	60	5.4	43	404	12.5	38.5	15.3	334	17.1	0.65	141	4.6	102	9.6	4.2	79	15.9	11.3	0.8	1.7
66	M	156.9	51.2	20.8	17.7	81.2	141	81	101	55	18.1	6.9	4.3	1.7	0.4	15	12	21	255	193	135	33	141	6.1	90.1	555	16.7	50.7	22.8	431	15.5	0.76	142	4.3	101	9.5	4.5	49	13.3	14.6	1	1
36	M	172.1	59.5	20.09	21.7	74.2	109	69	82.33	63	10.8	7.5	4.6	1.6	0.8	22	24	68	242	199	82	53	131	5.2	57	526	15.4	46.2	16.1	247	11.3	0.89	142	3.9	103	9.1	6	59	14.1	12.8	0.7	0.5
71	F	151.5	69.3	30.19	42.1	105.3	140	73	95.33	72	14.1	7.4	4.4	1.5	1.5	15	9	20	183	191	64	64	103	5.3	54.2	462	14.2	42.7	18.5	446	18.4	0.66	142	3.8	103	9.6	6.3	77	9.7	13	0.8	0.8
68	M	163.3	66.8	25.05	23.5	84.7	129	87	101	71	33.1	7.2	3.8	1.1	0.5	26	22	57	225	167	118	57	43	5.4	48.7	466	15	45.7	21.6	416	13.1	1.03	141	4.1	102	9	4.2	146	10.4	11.2	0.9	2.2
60	M	165.4	66	24.13	18.8	82.7	151	90	110.3	59	20.8	6.7	4.4	1.9	0.7	18	19	60	191	168	101	62	72	5.3	58.1	475	13.5	41.9	16.8	235	13	0.8	138	4.3	102	9.3	5.9	42	12.9	12.7	0.7	0.9
61	M	162.5	63.2	23.93	22.8	88.5	147	101	116.3	60	9.7	7.7	4.5	1.4	1.2	30	16	85	157	251	200	63	146	5.5	84.7	481	14.6	43.5	28.2	250	14.1	0.86	142	4.2	103	9.5	4.9	93	13	14.3	2.5	1.4
60	M	181	97.6	29.79	30.3	105.7	137	78	97.67	54	16.9	7.5	4.1	1.2	0.6	17	18	29	176	202	177	43	128	5.5	66	484	10.4	34.7	38.7	212	14.8	1.02	140	4.2	106	9.1	7.5	86	13.6	13.4	0.8	0.7
70	F	155.3	55.3	22.93	27.3	91.2	116	70	85.33	77	21.9	7.2	4.2	1.4	0.7	19	10	19	154	230	87	81	126	6.3	55.2	432	12.8	40	22.4	204	15.8	0.71	140	4.2	102	9.5	5.5	66	10.6	11.2	0.8	0.8
64	M	163.3	50.3	18.86	18.8	70.6	111	65	80.33	72	35.3	7.3	4.3	1.4	0.7	71	101	71	173	198	64	79	98	5.2	66.6	463	14.1	43.3	16.4	241	15.6	0.91	143	4	104	8.4	6.3	75	12.9	12.6	0.8	0.8
60	M	168	62.7	22.22	23.4	87.5	114	75	88	82	40.6	7.6	4.4	1.4	0.9	15	1	24	211	200	136	42	128	5.4	47	476	14.4	43.6	20.6	286	16.1	0.73	142	4.3	104	9	5.1	90	15.2	15.4	0.5	0.5
81	M	155	59.9	24.93	16.7	88	134	60	84.67	77	25.4	7.2	4.3	1.4	0.8	21	14	32	484	182	50	96	73	5.4	42.4	452	13.9	41	11.4	291	11.1	0.88	143	3.6	107	9	7.5	142	9.3	10.4	2.8	1.6
33	M	166.5	62	22.36	22.8	80.7	104	55	71.33	51	13.5	6.8	4.4	1.8	0.3	16	12	15	195	166	94	51	82	5.2	80.8	534	15.4	45.7	27.9	185	13.2	0.88	139	3.8	103	8.8	7.1	59	16.7	13.7	0.6	0.5
38	M	162.1	58.3	22.19	23.5	78.8	103	59	73.67	48	4.8	7.4	4.6	1.6	0.5	33	17	23	370	227	100	63	138	4.9	54.5	507	15.4	44.5	16.3	254	12.6	0.79	142	3.5	102	9.4	5	56	14.5	10.7	0.5	0.5

59	F	158.2	44.3	17.7	8.9	75.3	126	72	90	63	56.2	6.8	4.4	1.8	2	88.6	140	94	222	247	88	150	46	5	55	432	13.9	41.6	17.8	188	11.9	0.53	138	3.4	99	9.2	3.4	42	16.8	15.4	1.7	1.4
65	F	158.5	65.2	25.95	35.2	91.7	148	94	112	69	34.3	7.3	4.2	1.4	0.7	47	58	47	346	159	64	60	74	5.2	60.6	435	13.6	40	23.6	194	13.3	0.55	139	4	102	9.2	4.1	59	12.1	10.6	0.9	1
79	F	147.4	46.1	21.22	22.4	86.5	129	72	91	65	22.1	7.2	4.4	1.6	0.7	23	13	15	203	205	64	67	110	5.5	50.2	413	12.3	37.9	17.4	316	23.8	0.65	141	3.6	100	9.2	5.1	88	10.8	11.7	0.6	0.7
62	M	173.4	74.7	24.84	23.2	86.2	134	80	98	64	10.3	7.8	4.6	1.4	0.8	56	101	46	259	233	188	59	135	5.7	63.4	525	15.8	47.3	13.7	227	12.6	0.94	138	4	99	9.7	6.2	90	15.5	17.2	0.8	1
72	F	152.5	37.8	16.25	14	62	141	75	97	57	37.7	7.3	4.5	1.6	1.8	53	23	43	386	197	80	76	92	5	33.4	408	13	39.8	11.9	244	10.8	0.57	141	3.3	101	9.4	4.3	83	10.8	13.9	0.9	0.8
67	M	161.3	56.2	21.6	19.6	83	139	66	90.33	65	35.5	7.2	4.2	1.4	0.8	29	22	71	201	206	56	86	96	5.3	51.6	459	15.1	45	29.7	291	12.7	0.76	140	4.8	102	9.3	4.6	91	14.4	13	0.7	0.6
63	M	167.3	76.7	27.4	37.8	94.5	135	84	101	66	16.9	8	4.7	1.4	0.6	17	13	27	264	167	177	56	44	9.9	60.8	534	15.1	46.3	24.5	176	19.2	0.6	137	4.1	100	9.3	5	35	17.7	20.4	1.1	0.7
74	M	167.5	74.7	26.63	22.8	96.8	126	71	89.33	90	94.7	7	4.4	1.7	0.8	24	22	23	188	159	127	44	89	5.4	62.1	458	14.9	43.7	19.7	311	13.4	0.83	140	4	104	8.8	6.2	67	17.1	15.1	0.7	1.6
59	M	168.8	60.8	21.34	18.1	83	124	80	94.67	62	14.6	6.5	4	1.6	0.7	21	15	122	174	181	78	80	82	5.5	49.5	460	15.1	44.1	22.6	277	11.8	0.85	141	4	102	8.8	5.1	102	10.5	9.5	1.1	1.4
60	F	160.9	67.5	26.07	33.1	99.3	129	84	99	62	26.7	6.8	4.2	1.6	0.8	24	24	29	156	249	142	71	150	5.6	45.5	512	15.4	46.3	15.9	186	14.7	0.52	141	4.2	103	9.4	6.4	75	10.8	11.5	0.7	0.7
68	M	169.7	83	28.82	31.4	94.3	151	95	113.7	66	8.5	7.9	4.5	1.3	1	32	32	37	233	129	82	39	71	5.7	70.8	475	15.6	45.6	20.3	238	14.5	0.75	141	4.1	104	9.1	6.8	58	13.5	14.2	1.2	1.4
70	M	169.2	70.2	24.52	20	94.5	114	55	74.67	77	50	7.6	4.3	1.3	0.7	29	24	83	195	173	59	79	72	5.4	53.5	384	12.9	39.4	26.5	222	14.6	0.69	139	4.1	102	9.2	4	68	11	8	1.1	1.3
62	M	152.5	61.4	26.4	21.9	83.3	148	83	104.7	78	33.8	7.2	4.4	1.6	0.8	26	24	41	353	206	136	52	132	5.3	59.2	465	14.6	43.6	22.2	241	10.5	0.7	139	3.9	99	8.9	5	69	11.2	9.9	2.5	1
54	M	166.6	66.6	24	19.9	85.5	96	55	68.67	54	17.1	6.7	4.5	2	0.9	16	13	15	162	187	97	57	102	5.4	51.2	488	4.5	43.1	15.5	204	12.6	0.83	142	4	104	9	6.1	82	12.5	15.5	0.7	0.6
57	M	160.4	47.2	18.35	11.3	64.5	104	59	74	43	18.8	7.1	4.4	1.6	1	20	18	35	226	256	75	86	141	5.7	61.5	455	14.6	43.2	19.8	217	17.1	0.89	142	4.1	103	9.4	3.4	69	11	7.9	1	0.6
61	F	146.3	43.2	20.18	21	61	138	75	96	68	18.5	7.1	4.5	1.7	1.1	47	65	17	248	235	107	82	127	5.5	46.4	481	13.8	42.7	20.6	277	12.9	0.62	142	4.2	102	9.3	3.7	193	8.6	9.1	0.6	0.8
67	M	157.4	51	20.59	20	73.5	114	65	81.33	55	14.1	6.7	4	1.5	1.1	19	11	165	295	164	106	55	83	5.2	36.5	432	14.2	42.4	28.3	277	9.8	0.79	138	4.2	104	8.8	2.8	102	11.9	9.5	0.8	0.9
58	M	172.8	57.6	19.29	15	75	156	83	107.3	66	71.5	6.8	3.9	1.3	0.7	19	17	1	189	154	71	52	85	5.6	67.3	504	14.7	44.7	20	465	17.1	0.79	142	4.1	101	9	4.6	72	7.5	7.6	1.1	0.6
59	F	154.5	48.9	20.49	25.3	74.6	134	72	92.67	66	9.8	7.4	4.4	1.5	0.6	23	20	19	161	229	88	77	128	5.4	50.1	415	12.2	36.7	26.8	291	13.7	1.56	140	3.4	102	9	4	165	14	12	0.7	0.8
73	M	170.4	76.7	26.42	22.5	94	115	70	85	57	16.7	6.5	4.1	1.7	0.5	19	19	38	162	144	116	35	88	5.2	56.9	458	14.6	42.5	15.2	172	14.4	0.68	139	3.6	103	8.6	5.7	46	7	7	0.7	0.8
36	M	166.1	85.6	31.03	30.5	104	140	80	100	58	14.1	7.1	4.5	1.7	0.6	34	75	75	226	261	272	44	177	5.8	116.7	508	15.8	46.5	26.9	269	14.3	0.74	139	4.2	101	9.4	4.6	50	10	10	0.6	0.6
41	M	170.9	70.1	24	29.4	89.5	123	76	91.67	64	6.9	7.7	4.7	1.6	0.4	17	27	63	209	327	353	59	222	5.5	118.4	509	16	47.2	34.5	244	10.7	0.83	140	4	100	9.9	6.1	91	14	16	0.8	0.6
42	M	163.2	86.6	32.51	34.9	106	161	80	107	64	24.9	7.1	4.4	1.6	0.7	36	52	94	163	196	131	46	135	5.8	73.5	580	17.5	50.3	22.3	235	9.9	0.87	140	3.8	100	9.3	7.4	46	14	16	0.9	1
65	M	157.3	50.1	20.25	16.6	72.3	120	72	88	83	16.7	7.5	4.6	1.6	1.1	23	17	15	176	229	151	43	127	6.2	75.2	413	13.3	40.1	23.5	322	16.7	1.03	141	4	101	9.4	6.6	75	17.7	17.6	0.7	0.6
67	F	158.1	78.2	31.29	36.2	101	147	66	93	72	29.1	7.4	4.5	1.6	1	42	58	40	298	239	68	42	173	6.1	55.8	492	15	45.1	16.3	265	17.8	0.63	144	4.3	102	9.6	5.7	32	8.9	9.3	0.8	1
57	F	153	44.7	19.1	22.9	74.5	93	49	63.67	61	7.3	7.6	4.3	1.3	0.7	18	13	9	270	252	85	79	132	5.2	32.6	431	13.3	40.9	21	230	15.2	0.55	139	4.4	100	9.5	4.6	81	9.8	14.2	0.6	1.2
61	F	162.5	52.3	19.81	21.2	72.7	130	71	90.67	50	97.3	6.6	4	1.5	1.4	21	15	42	212	225	46	65	137	5.6	44.3	440	13	39.3	14.9	250	15.5	0.75	143	4.1	104	9.1	5.8	54	7.8	7.6	0.5	0.7
72	F	155.3	55.9	23.18	29.1	90.8	143	78	99.67	67	38.6	7.8	4.5	1.4	0.5	24	19	15	200	257	88	57	192	5.5	94.4	510	14.6	43.8	21.7	269	18.5	0.69	141	4.2	104	9.4	7.5	73	12.6	12.7	0.8	0.8
60	F	157.3	56.2	22.71	29.6	78.5	126	83	97.33	75	19.4	8.1	4.5	1.3	0.6	22	17	24	351	226	92	54	148	5.1	58.6	448	13.1	39.9	23.3	208	10.9	0.46	142	3.6	104	9.1	2.6	71	15.5	17.2	0.6	0.7
73	F	155.4	45.3	18.76	19.4	83.5	150	79	102.7	71	73.3	7.3	4.5	1.6	0.9	31	15	23	196	174	80	55	93	6.6	58.3	461	12.7	39.8	16.5	192	20.6	0.65	140	4.4	99	9.2	5	78	13.4	15.6	0.6	0.6
64	M	163.6	68.5	25.59	29	87.5	144	91	108.7	53	34.8	7.7	4.7	1.6	2.3	24	23	59	200	223	96	66	139	5.6	67.8	519	17.5	51	22.8	446	14.4	0.89	139	4.3	98	9.7	7	63	8.8	10	1.4	0.6
69	M	170.1	64.7	22.36	21.5	89.3	140	81	100.7	60	19.1	7.6	4.7	1.6	1	39	37	29	191	194	76	40	133	5.4	38.8	460	14.4	42.5	16.4	261	14.2	0.76	141	3.7	101	9.2	6	87	18.9	17.7	0.7	0.7
77	F	146.5	48.1	22.41	27.7	85.2	113	63	79.67	65	12.5	7.4	4	1.2	0.5	22	13	16	439	211	121	50	144	6	79.3	435	13.2	40.7	29.6	431	15.2	0.56	141	4.3	101	9.1	4	61	16.7	16.1	0.8	0.9
50	F	151.6	62.9	27.37	40.5	87.5	148	95	112.7	63	15.8	7.6	4.4	1.4	0.9	21	20	18	203	273	97	69	174	6.4	49.6	482	14.5	45.3	21.3	277	12.1	0.61	142	4.1	101	9.5	6.8	50	17.3	16.9	0.8	0.6
61	M	166.9	54.7	19.64	13.3	74	135	95	108.3	72	7.8	7.3	4.5	1.6	0.6	31	30	36	134	237	117	61	164	5.5	56.7	465	14.5	44.2	20.7	224	10	0.65	141	4.2	102	8.9	7.3	88	15.1	15	0.6	1
82	F	134.4	42.9	23.75	29.1	84.5	139	69	92.33	59	83.8	7.4	4	1.2	0.6	29	16	30	174	233	168	64	125	5.5	59	424	12.9	40.6	18	250	16.3	0.76	142	3.6	104	8.9	5.2	70	13.8	12.9	0.7	0.7
73	F	152.1	41.3	17.85	23.9	75.5	89	57	67.67	59	19.6	8.1	5	1.6	0.5	27	21	30	347	311	94	105	180	5.2	61.6	448	13.9	42.3	23.6	340	19.9	0.73	141	4.7	101	10.1	4.3	70	9.1	10.1	1.4	1.1
74	F	150.6	54.1	23.85	34.2	89.5	114	73	86.67	61	12	7.1	4.6	1.8	1.8	24	19	19	276	261	168	44	174	5.4	43.9	480	15.3	47.6	20.2	277	13.1	0.6	144	3.7	103	9.2	4.4	100	9	10.4	1.5	1.1
35	F	155.9	60.2	24.77	31.1	77.7	112	57	75.33	59	16.3	7.2	4.4	1.6	0.5	19	16	24	161	263	60	73	165	5	38.5	412	12.5	36.7	25	227	7.7	0.53	138	4	101	9.1	3.3	92	10.3	11.7	0.5	0.5
85	M	165.2	71.4	26.16	21.3	93.5	163	86	111.7	72	38.4	7.7	4.5	1.4	0.8	36	41	47	245	201	80	56	135	5.7	69.9	461	14.5	42.3	21.4	257	20.6	1.09	141	4.1	102	9.4	7.6	230	11.6	10.6	0.9	1.1
66	M	164.9	59.9	22.03	11.1	83.8	115	65	81.67	64	13.1	6.7	4.3	1.8	0.5	26	26	67	219	169	79	66	86	4.9	65.4	464	15	43.1	18.4	291	18.5	0.88	140	4.3	102	8.9	5.6	179	11.5	16.1	1.3	2.3
65	F	154.1	52.4	22.07	28.7	83.8	167	87	113.7	75	12.3	7.4	4.3	1.4	0.6	19	18	20	261	194	116	50	119	5.7	67.8	466	14.2	44.2	32.1	296	16.3	0.49	141	4.6	101	9.6	5.1	100	14.4	19	0.8	1.1
84	F	143.7	38.7	18.74	24.9	78.8	126	66	86	81	30.5	7.8	4.4	1.3	0.6	23	14	19	315	201	89	69	113	6.1	49.7	423	13.3	40	30.7	401	14.5	0.47	143	3.8	104	9.4	4.7	73	9.1	13.9	1	1.3
67	F	149.2	59.4	26.68	32	85.5	117	65	82.33	57	26.2	7.2	4.3	1.5	0.4	23	16	17	202	211	148	64	120	5.5	65.8	470	13.9	41.8	22.8	334	16	0.61	141	4.2	101	9.2	5.4	121	13.1	11.1	0.8	0.6
40	M	161.7	77.9	29.79	31.8	95	128	76	93.33	62	11.1	7.8	4.5	1.4	0.5	26	49	55	251	204	170	49	135	5.5	71.7	514	14.7	45.7	17.8	269	10.4	0.63	141	4.2	102	9.2	4.9	63	14.7	15.2	0.6	0.6
69	F	148.5	36.9	16.73	15.8	66.8	96	53	67.33	58	28.7	7.3	4.3	1.4	0.6	27	16	20	120	253	89	84	128	5	42.2	419	13	39.6	17.8	194	13.4	0.53	143	4.6	106	10	5.5	110	11.8	11.9	0.8	0.7
76	M	155.3	69.7	28.9	33.3	99	115	71	85.67	82	13.7	8.1	4.4	1.2	1.2	25	21	75	265	178	120	43	104	5.1	48.3	436	14	41.9	13.8	269	15.1	0.69	140	3.5	102	9.5	5.3	104	11.7	14.2	3.4	4
66	F	155.9	68.4	28.14	37.8	96.6	124	75	91.33	52	22.9	7.4	4.1	1.2	0.6	26	23	23	192	235	102	53	144	5.7	60.1	436	13.4	41	25.6	286	19.1	0.78	141	3.9	102	9.5	6.6	91	15.5	15.8	0.9	0.9
65	M	168.9	59.5	20.86	14.8	79	128	80	96	74	6.1	7.5	4.6	1.6	1	28	29	43	296	246	88	91	121	5	66.2	480	15.9	45.9	16	235	12	0.65	139	3.7	98	9	4.6	44	17.4	18.1	0.5	0.8
76	M	161.7	62.4	23.87	23.1	86.5	140	82	101.3	56	26.2	8	4.6	1.4	0.9	25	25	52	141	247	139	45	158	5.6	55.2	543	15.9	47.4	15.3	340	15.8	0.83	138	4.6	99	9.4	6.5	62	15.6	18.6	0.7	0.8
92	M	159.9	59.2	23.15	16.6	80	158	76	103.3	67	122	7.1	4.1	1.4	0.5	24	15	15	307	174	76	60	78	5.6	62.7	385	11.7	36.4	17.1	311	25.4	1.16	138	4.3	101	9	6.1	155	7.2	10	1.4	3.6
61	F	159.2	57.4	22.65	27.5	85	125	65	85	66	5.9	7.3	4.5	1.6	1	27	25	32	189	198	157	56	108	5.4	50.2	476	14.1	43.1	19.6	254	16.5	0.64	141	4	102	9.5	4.6	46	10.9	10.3	1.4	1.5
64	M	164.9	65.1	23.94	28.9	90	114	77	89.33	68	17.8	7.1	4.3	1.5	1	17	18	31	192	231	130	44	170	5.3	56	520	16.3	49.3	23.1	286	12.3	0.79	139	4.1	103	9.2	5.6	83	17.6	14.6	0.6	0.7
60	F	156.3	57.4	23.5	31.9	88	121	65	83.67	75	19.3	7.2	4.5	1.7	1.1	18	14	25	256	231	83	71	137	5	52.3	432	14	42.1	21.3	208	12.1	0.54	139	3.7	101	9.4	5.2	77	14.6	14.8	0.7	0.8
65	F	150.2	42.1	18.66	22	69.5	134	63	86.67	51	26.3	7.5	4.3	1.3	0.7	29	19	48	165	195	60	93	75	5	37	457	14	43.1	12.8	261	14.9	0.5	140	4	101	9.5	5.3	74	13.5	13.8	1.1	1.3
69	F	147.9	46.2	21.12	24	76.5	114	65	81.33	71	9.4	8.1	4.5	1.3	0.9	32	15	14	184	203	148	60	113	5.3	41.5	436	13.2	40.2	25.2	306	13.3	0.55	140	4.7	98	9.6	3.6	102	16.7	15.4	0.8	0.7
76	F	145.7	48.9	23.04	28.6	83.8	98	51	66.67	87	153	7.1	4.4	1.6	0.6	26	20	39	311	194	87	75	91	5.6	49.4	432	13.3	41	19.2	241	9.4	0.58	141	4.2	101	9.3	4.8	105	12.6	12.3	0.9	0.8
33	F	150.3	53.3	23.59	33.7	81.5	108	59	75.33	65	19	7	4.4	1.7	0.4	27	18	24	248	225	241	55	128	4.9	75.1	453	13	40.7	31.9	257	20.1	0.55	139	4.6	103	9.1	5.2	93	10.5	14.9	0.7	0.6
60	F	143.1	51.5	25.15	33	91.5	108	56	73.33	69	16.7	6.8	4.4	1.8	0.6	22	15	144	210	193	120	53	113	4.6	50.5	438	13.6	39.6	26.7	238	10.1	0.6	141	4.4	102	9.2	4.9	75	9.3	10.5	0.6	0.8

63 F	145.6	55.6	26.23	37.2	84.5	164	98	120	70	7.9	7.7	4.7	1.6	0.7	17	17	33	341	231	313	49	119	5	56.6	501	15	44.5	24.8	217	21.9	0.46	139	3.9	99	9.6	4.7	115	15.3	16.2	0.8	0.8
87 M	161.4	59.3	22.76	14.7	81.5	124	60	81.33	67	272	7.4	4.1	1.2	0.6	18	11	15	227	184	40	71	100	4.9	30.4	371	10	33.3	18.7	296	13.3	0.65	140	4.3	102	8.9	4	113	7.6	7.3	3.2	1.6
64 M	162.9	65.9	24.83	26.9	87.2	137	87	103.7	58	10.3	7.5	4.2	1.3	0.8	19	12	136	325	215	163	73	109	8.9	66.1	520	16.5	49	24.5	265	14.7	0.67	140	4.3	101	9.6	5.2	37	20	20.5	1	0.8
65 M	164.1	53.3	19.79	21	75.5	106	63	77.33	57	5.3	7.4	4.2	1.3	0.6	14	11	12	337	205	342	44	104	5.2	50.3	504	14.3	43.3	24.3	322	12.9	0.77	140	3.8	99	9.3	5.7	58	13.1	7.8	0.7	0.7
60 M	171.7	66.5	22.56	20.6	84.2	147	67	93.67	57	25.8	7.7	4.7	1.6	0.9	28	18	44	219	195	129	73	92	5.5	41.2	440	14.9	46.1	22.9	217	12.6	0.85	140	4.1	97	9.8	5.2	77	12.6	12.5	1.1	0.8
62 F	155.2	46.5	19.3	21.5	78	127	86	99.67	60	15.1	7.1	4.5	1.7	0.8	30	36	19	305	231	127	74	126	5.2	42.1	506	15.4	47.2	17.8	238	9.6	0.51	141	3.6	100	10.5	3.8	109	11.4	13.5	0.6	0.8
60 M	172.1	80.4	27.15	25.9	93.4	135	87	103	95	5.1	7.1	4.5	1.7	0.9	27	29	96	278	180	409	49	56	5.4	64	528	16.7	48.8	20.1	282	12	0.84	139	4.2	99	9.6	6.1	71	11.6	12.3	0.6	0.8
68 F	151	46	20.17	28.4	80.6	136	74	94.67	75	17	8	4.5	1.3	0.5	23	13	19	278	212	127	68	107	5.3	60.1	477	14.3	44.4	20.8	291	13.6	0.71	143	4	103	9.5	5.1	72	14.9	12.7	0.7	0.8
60 F	155.3	61.9	25.67	37.5	82.2	118	62	80.67	63	21.3	7.6	4.2	1.2	0.6	18	16	17	293	208	86	62	125	7.8	63.9	409	12.4	38.6	32.4	254	18.5	0.73	143	3.8	104	9	5.2	108	13.3	13.9	0.8	0.8
66 M	168.9	83.9	29.41	26.4	106	145	85	105	68	17	7.7	4.2	1.2	1.5	44	49	79	218	201	80	69	109	5.6	73.5	498	16.4	47.9	15.5	269	10.9	0.62	140	3.9	101	9.3	3.5	103	14.2	14.4	0.8	1
60 F	158.9	47	18.61	22.5	70	103	61	75	74	6	8.6	4.9	1.3	0.9	32	28	27	181	260	82	82	140	5	46.5	473	14.9	44.5	23.8	257	11.2	0.41	141	3.6	99	10	4.4	136	12.6	11.5	0.7	0.7
71 F	166.1	53.5	19.39	23.6	77.5	127	73	91	70	25.1	7.8	4.5	1.4	1.2	23	19	15	257	209	68	79	108	5.7	75.5	456	14.8	45.4	19.3	322	11.1	0.51	142	3.9	99	9.4	4.6	100	16.2	15	0.6	0.7
59 F	146.6	61	28.38	40.2	89	98	59	72	57	9.9	7.9	4.4	1.3	0.7	36	61	72	216	242	125	54	160	7	83.6	496	15.9	45.9	26	311	10.9	0.44	140	3.7	102	9.2	4.9	33	13.9	14.9	0.7	0.7
63 M	158.6	48.8	19.4	15.2	74.7	114	60	78	55	9.6	8	4.9	4.6	0.7	26	13	22	231	211	106	78	110	5.6	58.8	493	14.8	45.4	15	222	14.3	0.83	140	3.8	99	9.5	6.7	112	20.8	19.4	1.2	0.8
65 M	175.2	79.1	25.77	27.5	91	144	79	100.7	51	41.7	7.6	4.3	1.3	0.4	33	31	71	239	179	61	66	104	5.8	93.7	451	14.7	44.9	19.7	257	11.5	0.66	141	4.1	103	9.2	5.5	65	10.4	12.1	1.2	1.4
75 M	161.3	69	26.52	24.8	88.2	144	75	98	63	355	7.5	4.7	1.7	0.7	16	23	65	168	160	73	55	87	5.6	42.8	459	15.1	46.3	11.9	247	22.5	1.07	143	3.8	103	9.1	6.8	97	7.9	10.5	0.9	1.1
70 F	154.7	48.1	20.1	21.8	77.3	143	81	101.7	70	111	8.4	4.3	1	0.6	28	17	31	251	170	195	45	69	5.6	34.4	466	12.4	39.1	9.6	286	22.3	0.54	139	3.7	102	9.3	3.6	109	12.4	13.3	0.8	0.7
49 F	155	54.8	22.81	28.6	77.2	113	66	81.67	65	14.6	6.9	4.1	1.5	0.7	19	20	17	256	231	54	78	135	5.4	59	412	12.6	39.1	40.8	269	7.7	0.52	139	3.6	101	9	3.4	69	11.4	12.1	0.7	0.6
78 M	154.1	48.3	20.34	20.8	73.5	156	72	100	65	37.5	7.1	4.1	1.4	0.9	28	17	28	195	171	105	55	100	5.3	71.4	519	15.7	47	20.6	208	11.8	0.79	140	4	101	9.1	7.3	71	10.8	12.6	0.8	0.9
79 F	145.2	49.9	23.67	31.3	86.3	160	75	103.3	61	97	7.6	4	1.1	1.3	21	14	17	182	240	132	41	167	5.1	44.3	392	13.3	40.3	26.5	254	16.6	0.76	140	4.7	103	9.5	3.4	79	14	12.8	1.1	1.2
69 F	158.5	53.5	21.3	23.8	80	122	64	83.33	78	24.3	7.5	4.3	1.3	0.7	24	18	17	214	250	99	63	156	5.3	57.9	441	13.5	42.3	15.4	277	19.7	0.83	140	4	99	9.4	5.5	95	10.4	12.3	0.7	0.8
49 F	159.3	53.9	21.24	25.6	74.7	95	54	67.67	61	33.2	7.6	4.3	1.3	0.5	22	16	13	118	223	28	97	105	5.4	48.3	420	9.7	32	28.1	244	11.1	0.55	139	4	101	9	3.1	71	11.3	12.1	0.6	0.6
70 F	155.7	54.6	22.52	34.7	85.8	144	83	103.3	56	45.7	6.8	4.2	1.6	1	23	17	54	172	215	83	72	121	5.2	75.3	473	14.7	44.6	20.4	282	15.6	0.71	140	3.9	99	9.2	5	77	15	16.7	1	2.3
69 F	148.5	43.4	19.68	23.3	80	127	64	85	67	25	6.9	3.9	1.3	0.6	20	15	13	169	193	93	60	112	5.3	57.4	393	12.3	37.7	19.8	482	11.9	0.52	141	3.7	101	8.5	3.8	97	12.5	15.9	1.7	0.7
81 M	162.9	56.4	21.25	16	84.2	154	82	106	81	19.6	7.2	4.6	1.8	0.6	25	17	21	269	216	200	52	140	5.8	92.6	410	13.1	38.6	15.4	301	12.2	0.88	139	3.7	100	9.3	4.2	151	11.1	11.9	2.6	1.8
75 F	148.9	42.3	19.08	18.5	68.5	153	81	105	79	30.5	7.3	4.1	1.3	0.5	23	15	15	272	207	91	74	109	5.8	49.1	363	11.2	35.5	32.6	368	10.4	0.63	141	4.4	103	9.5	4.7	109	10.9	11.1	0.7	0.8
61 M	170.7	73.1	25.09	21.7	94.2	139	87	104.3	76	12.9	7.5	4.6	1.6	1.1	24	25	225	239	251	251	49	154	5.3	59.3	515	15.2	45.2	20	257	11	0.69	139	3.4	100	8.7	3.9	118	18.4	17.6	0.6	0.8
70 M	165.7	70.9	25.82	32.5	92.8	123	75	91	55	8.7	7.5	4.5	1.5	0.8	52	97	242	309	173	59	52	102	5.2	52.9	501	15.7	45.1	15.7	375	19.4	0.87	140	3.7	101	9.1	7	74	10.5	9.3	0.7	0.8
78 F	145.7	45.9	21.62	28.3	80.5	142	91	108	89	14.2	7.8	4.8	1.6	0.9	29	43	64	254	258	148	68	149	6	46.2	510	15.1	45.8	21.2	227	14.8	0.59	141	4.3	102	9.6	3.8	58	14.7	16.3	1	1.4
63 F	158.5	55.1	21.93	27.8	87.3	141	84	103	103	12.6	7.7	4.7	1.6	0.6	19	19	45	234	238	152	5	143	5.3	83.7	473	14.6	45.9	16.3	254	19.1	0.64	140	3.8	101	9.7	4.6	84	11.8	10.3	0.8	0.8
63 M	164.1	64.4	23.91	26.9	89.7	159	96	117	68	136	7.2	4.3	1.5	0.6	17	17	117	256	201	186	70	107	5.2	94.8	424	14.4	44.3	25.9	388	9.1	0.83	142	4	105	9.1	4.4	68	10.8	11.1	0.7	1.1
57 F	168.5	75.6	26.63	38.5	102	127	75	92.33	51	9.9	7.8	4.7	1.5	0.8	22	17	15	277	276	89	62	189	5.6	48.2	477	14.8	44.9	20.6	334	16.6	0.6	138	4	102	10.6	4.6	59	9.9	9.4	1	1.2
60 M	175.9	67.2	21.72	16.9	83.5	128	82	97.33	45	36.9	7.4	4.6	1.6	0.6	18	21	25	229	173	155	42	114	5.3	55	490	15.6	47.6	18.9	306	10.4	0.75	142	4	99	9.2	6.2	57	14.3	16.1	0.9	1
61 F	154.9	52.2	21.76	28.9	81	120	72	88	57	5.8	7.9	4.6	1.4	0.8	22	17	23	275	235	99	61	132	5.4	57.7	485	14.2	43.4	21.9	322	15.9	0.61	140	3.9	100	9.5	4.5	94	8.6	7.7	0.8	1.2
33 M	178.7	64.1	20.07	17.5	71.3	128	81	96.67	67	2.8	7.6	4.9	1.8	1	25	17	23	160	188	82	58	115	4.9	65.2	491	15.2	44.3	21.9	179	11.4	0.9	139	4.4	100	9.6	5.1	93	14.8	11.6	0.6	0.6
72 F	158.1	40.9	16.36	17.1	68.5	126	61	82.67	67	52.8	7.4	4.6	1.6	0.7	31	19	21	219	201	94	98	80	5	25.5	395	13.6	40.1	12.7	241	12.8	0.67	140	4.1	100	9.4	4.8	60	13.6	12.6	0.8	0.9
74 M	161.7	65.1	24.9	27.2	91	157	87	110.3	61	12	8.2	4.9	1.5	0.6	23	15	32	339	281	275	61	183	5.6	55.2	506	15.4	45.8	23.3	328	11.4	0.65	140	4.1	99	9.8	3.7	116	13.8	17.6	0.9	0.9
78 F	147.8	43.6	19.96	22.9	79.6	118	65	82.67	99	22.2	7.1	4.1	1.4	0.6	16	10	14	252	169	112	45	115	5.2	68.8	422	13	39.8	18.8	291	16.9	0.6	142	3.7	104	8.9	4.3	61	15.9	13.4	0.8	0.7
61 M	160.5	59.3	23.02	22.2	89	145	86	105.7	62	15.6	8.2	4.8	1.4	0.9	36	28	83	260	213	152	63	117	5.7	55.3	490	16	49.3	21.2	494	8.4	0.72	141	3.6	99	10.1	5.8	71	10.8	10.2	1	1.9
60 M	167.6	58.3	20.75	16.9	81.7	125	81	95.67	60	109	6.7	4.3	1.8	1.1	16	8	29	263	213	136	74	99	5.2	59.7	450	14.8	44.3	15.8	190	19	0.82	142	4.2	105	9.1	4.9	119	13.3	11.4	0.6	0.8
63 F	150.9	52.4	23.01	30.2	84.2	145	83	103.7	71	10.6	7.3	4.5	1.6	1.1	22	19	16	209	257	136	61	159	5.7	70.9	458	14.7	44.5	16.8	224	15.2	0.65	140	4.1	101	9.4	4.7	81	13.2	13.8	0.8	0.9
70 M	167.4	62.5	22.3	19.2	84	147	82	103.7	76	11.9	7.1	4.4	1.6	0.8	15	11	28	229	219	158	53	145	6.2	70.6	525	16.9	49.4	16	206	13.7	0.77	143	3.7	105	8.9	5.5	72	16.3	15.3	1.4	1.1
62 F	154.2	50.7	21.32	25.9	79.7	122	71	88	72	19.9	7.9	4.5	1.3	0.6	25	31	36	313	241	159	46	171	5.7	85.4	490	14.3	45	22	322	15	0.6	141	3.7	101	9.1	5.3	71	16.9	15.5	1	1.1
56 F	149.4	52.7	23.61	34.5	77.7	118	68	84.67	63	8.8	7.9	4.6	1.4	0.9	17	10	16	237	301	98	69	203	5.6	50.7	479	14	43.1	19	273	16.8	0.55	142	3.9	103	9.3	5.5	60	13.3	10.9	0.6	0.5
68 M	161.8	63.5	24.26	24.8	90	121	64	83	59	11.3	7.5	4.4	1.4	0.8	32	35	115	409	205	65	54	135	5.6	71.2	480	14.3	43.6	24.9	443	13.5	0.63	142	3.7	100	9.1	4.9	82	13.6	14.7	0.9	1
65 M	171.5	65.4	22.24	22	82.5	132	77	95.33	57	7.8	7.1	4.3	1.5	0.9	15	10	19	327	158	94	52	84	5.2	97	456	14.9	45.2	19.1	240	13.6	0.73	142	3.8	102	8.9	5.2	78	15.2	15.2	0.8	1
68 F	154.9	60	25.01	35.6	95.4	111	56	74.33	63	22.9	7.4	4.3	1.4	0.7	16	15	16	257	243	118	57	142	5.5	47.3	465	14.5	44.4	18.5	422	17.4	0.62	141	4.4	102	9.3	4.5	75	17.5	16.4	0.8	0.8
62 F	155.7	50	20.62	24.3	71.2	91	53	65.67	50	9.6	7.8	4.5	1.4	1	19	17	17	375	228	81	60	144	5.8	30.5	461	13.7	41	22.2	254	10.8	0.54	140	3.6	100	9.5	4.2	53	13	12.8	0.6	1
71 M	162.2	66	25.09	22.9	91	130	73	92	62	110	7.5	4.2	1.3	0.5	24	20	19	193	189	111	62	96	5.5	51.8	436	14.1	43.1	22.2	273	20.1	0.78	139	4.8	101	9.1	6.7	114	10.5	11.8	1.2	1.3
67 F	143.9	43.8	21.15	27.2	76	122	65	84	56	13.1	7	4.3	1.6	0.8	16	11	15	254	236	57	80	128	5.3	71.7	434	14.2	42.2	28.2	295	13.3	0.56	140	4.5	100	9.4	4.7	69	14.2	14	1	0.8
60 M	169.3	76.9	26.83	28.3	97.6	139	92	107.7	83	5.2	7.7	4.7	1.6	0.3	40	45	65	256	240	315	80	123	6	73.9	521	16.2	49.6	20.9	221	15.1	0.76	141	4.3	99	9.3	5	70	13.6	12.2	1.1	1
49 M	184.3	68	20.02	17.1	78.5	124	64	84	55	9.1	7.6	4.4	1.4	1.1	22	15	11	209	204	35	81	101	5.4	55	470	14.5	44.8	17.2	200	17.4	0.83	139	3.6	98	9.2	5.2	94	8.5	9.5	1	0.8
65 M	157.3	62.7	25.34	22.2	83	130	82	98	64	11.2	6.7	4.3	1.8	1.7	24	20	28	209	209	97	58	128	5.6	58.8	456	14.9	42.9	23.6	240	16.4	1.02	141	4.4	103	9.3	5	83	12.7	9.8	1.1	1.2
91 M	164.3	49.3	18.26	14.1	67.8	122	66	84.67	66	110	7.7	4.4	1.3	0.5	40	23	23	258	163	49	55	85	5.5	41	417	12.6	39	12.5	300	19.5	0.8	137	4.5	95	9.2	4.5	134	7.9	8.6	1.4	0.9
65 F	148	47	21.46	27.5	79.5	124	78	93.33	89	12.1	8.1	4.4	1.2	0.7	18	12	16	186	191	42	71	96	5.6	45.1	424	13	39.8	23.8	374	14.9	0.52	140	3.8	98	9.5	4.7	81	14.7	14.8	0.7	0.8
61 F	156.7	54.9	22.36	31.5	82	127	74	91.67	76	8.7	7.3	4.2	1.4	0.9	19	14	14	239	239	65	68	137	5.5	41.2	453	13.9	41.8	17.1	221	15.9	0.61	140	3.5	101	9.5	3.3	89	17.6	15.3	1	0.7
56 M	165.6	83.8	30.56	33.6	96	145	95	111.7	91	3.8	8	4.4	1.2	1.6	32	55	38	260	226	265	36	165	5.3	74.2	524	16.2	49	23.5	269	10.9	1.04	140	3.9	100	9.8	8.6	66	13.5	13	0.8	0.8
89 M	161.1	49.5	19.07	12.1	86.3	107	55	72.33	63	227	6.8	4	1.4	0.3	23	19	11	249	158	63	34	105	5.6	60.7	363	11.9	35.9	20.2	334	33.3	1.09	141	4.2	105	8.8	6.6	95	8.9	10.4	1.2	1.4
70 M	154.5	62.9	26.35	22.4	89.8	147	81	103	73	13.5	7.2	4.7	1.9	1.2	21	30	25	107	191	79	46	124	5.7	57.7	475	15.8	44.5	17.8	237	11.6	0.69	139	3.5	103	8.8	3.8	85	14.7	13.4	1.2	1.7

50	M	168.9	76.8	26.92	31.1	92.5	120	73	88.67	61	3.8	7.3	4.6	1.7	1	18	27	37	176	229	139	57	135	5.4	65.7	479	14.5	43.9	26.5	250	10.4	0.65	141	3.9	102	9.5	6	74	14.4	14.3	0.8	0.7
45	F	164	56.2	20.9	27.2	83.5	122	68	86	64	16.5	6.8	4.1	1.5	0.7	14	12	12	123	184	70	71	82	5.2	42.9	441	14	41.6	17.2	240	15.8	0.67	141	4.3	105	8.8	4.7	91	16.2	12	0.7	0.7
72	M	162.8	53.4	20.15	11.2	69	102	51	68	73	300	6.5	3.7	1.3	0.5	29	22	43	291	227	87	54	144	5.9	35.9	381	12.6	38.7	16.1	307	17.5	0.96	143	3.9	106	8.8	6.2	84	8.7	7.7	1.8	1.8
72	F	146.9	45.6	21.13	26.8	81.3	113	60	77.67	72	10	7.1	4.1	1.4	0.8	18	13	14	128	212	87	81	107	5.4	59.8	440	13.1	39.6	22.7	307	15.2	0.61	143	3.7	102	9.1	5	105	8.5	10.2	1.1	1
67	M	166.5	64	23.09	22.6	92.5	124	70	88	52	4.7	7	4.3	1.6	0.8	22	22	40	137	212	78	51	145	5.5	48.3	480	14.6	43.6	21.1	275	19.1	0.79	143	3.8	104	9.3	4.9	46	11.4	12	1.2	1.4
67	F	151.3	55	24.03	34	82.5	141	86	104.3	63	18.4	7	4.3	1.6	0.6	19	12	26	179	255	138	92	141	5.6	56.4	415	13.3	39.9	22.1	218	12.2	0.53	142	4.1	103	9.1	3.4	91	14.2	12.5	1.1	1.5
67	M	165.3	73.7	26.97	21.9	96.5	143	97	112.3	73	8.3	7.4	4.3	1.4	0.5	29	25	85	230	229	287	53	130	6.1	62	434	14	40	25.3	264	17.3	0.89	140	4.4	102	9.7	6.1	120	16.4	13.2	1.1	2
79	F	144.1	36.2	17.43	17.8	73.5	132	71	91.33	78	14.7	7.3	4.4	1.5	0.5	23	19	16	204	202	83	88	52	6.1	53	442	13.3	40.3	18.9	297	16.2	0.5	143	4	103	9.5	4.6	109	11.8	12.9	1.4	1
78	M	156.1	58	23.8	17.7	82.3	135	82	99.67	57	27.4	6.5	3.8	1.4	0.6	29	15	63	286	180	39	74	83	5.1	48.6	385	13.5	39.8	21.4	312	14.2	0.6	144	4.1	105	8.7	5.6	68	12.4	17.4	2.8	2.6
75	F	150	40.5	18	19.1	82.5	133	87	102.3	66	28.4	7	4.1	1.4	0.6	24	22	30	215	218	65	81	104	5.2	48.5	395	13.3	39.1	24.6	250	14.6	0.47	144	3.9	105	8.7	3.4	96	16	15.9	0.7	0.7
58	M	166.9	61.2	21.97	17.3	81	159	79	105.7	57	7.8	7.3	4.4	1.5	0.4	36	25	23	164	143	87	52	74	5.3	46.8	460	15.4	45.5	19.3	226	12.4	0.8	144	4	105	8.8	4.8	88	9.8	8.1	0.9	0.8
74	F	144.1	45.4	21.86	26.4	84	155	87	109.7	79	18.8	7.5	4.4	1.4	0.6	34	36	24	166	216	79	65	123	5.2	59.3	448	13.6	42.4	22	271	17.3	0.77	137	3.9	96	9.3	6.2	63	9.3	10	0.6	0.8
66	F	146.9	39.9	18.49	17.3	69	131	75	93.67	63	35.3	6.7	4.1	1.6	0.6	27	21	17	352	259	46	112	124	5.5	45.9	431	11.9	37.2	28.4	257	14.7	0.48	142	4.3	102	9.1	2.8	150	16.4	17.3	2	0.8
61	M	167.5	63.9	22.78	22.2	90.2	109	72	84.33	73	25.7	7.7	4.3	1.3	0.3	19	12	25	205	178	151	40	114	5.6	53	461	14.4	43	21.1	297	17.5	0.71	141	4.2	105	9.3	5.6	112	14.9	15.2	0.9	0.8
59	M	159	60	23.73	21.6	84.3	168	89	115.3	60	11.1	7.3	4.3	1.4	0.5	22	10	35	266	216	229	64	106	5.5	52.9	438	14.1	43.4	21	340	17.3	0.88	141	4	99	9.5	6.7	62	20	20	0.9	0.8
58	M	169.2	73.9	25.81	22.8	86	126	80	95.33	65	10.5	6.6	4.3	1.9	0.9	24	28	68	197	225	97	61	150	6	42.7	487	15.9	47	15.4	201	28.6	0.87	143	4.3	106	9.3	4.7	97	7.8	9.7	0.8	0.8
63	F	150.6	61.1	26.94	32.7	85	127	67	87	84	8.1	7.1	4.3	1.5	0.6	21	14	11	158	255	44	77	157	5.4	41.6	418	13.3	38.6	19.8	294	10.8	0.73	141	4.4	104	9.6	6	45	18.9	18.9	1	0.8
53	M	161.7	61.2	23.41	21.7	84.8	149	76	100.3	71	5.7	7.2	4.5	1.7	0.4	35	41	82	368	202	363	61	89	6.1	58.8	479	15.1	45	26.3	223	15	0.8	141	4.4	103	9.4	6.5	43	8.1	9	1.2	1
73	F	145.4	48.5	22.94	28.3	89	148	83	104.7	65	8.9	7.4	4.4	1.5	0.6	17	15	43	265	238	125	48	146	5.2	59.4	418	13.3	39.5	20.3	302	14.5	0.64	143	3.9	104	9.2	4.3	78	14.2	11.3	0.9	1.8
38	F	164.1	52.6	19.53	23.8	80	104	62	76	58	5.5	7.1	4.3	1.5	0.6	18	12	10	137	168	107	57	86	5.1	56.2	433	11.8	36.7	19.2	201	9.9	0.53	139	3.8	105	8.9	2.9	84	12.3	10.2	0.8	0.7
56	M	154.1	72.9	30.7	25.9	94	136	94	108	70	3.8	6.8	4.2	1.6	0.7	21	29	34	163	200	104	50	128	5.7	57.9	476	15.1	44.8	21.5	237	14.5	0.84	144	3.7	107	8.9	5.6	105	8.7	9.6	1	1.2
55	M	158.1	54.8	21.92	19.5	79.8	118	67	84	54	6.6	7.3	4.3	1.4	0.6	20	14	16	183	247	96	67	161	5	48.5	452	14.4	42.6	22.6	210	10.4	1.03	142	4.5	106	9.8	4.7	92	14.9	12.7	0.8	0.8
37	M	172.8	61.7	20.66	22.2	76.8	117	67	83.67	54	8.3	7.5	4.6	1.6	0.5	26	46	92	216	214	88	48	148	5.2	61.7	505	15.4	45.1	16.3	246	15.7	0.85	143	3.8	105	9.1	5.7	82	13.2	14.6	0.6	0.8
61	M	180.9	86.6	26.46	26	97.3	130	74	92.67	49	13.5	7.2	4	1.3	0.7	17	13	23	182	202	93	50	133	5.2	57	459	13.8	41	28.5	234	11.2	1.03	144	4.2	107	9.3	7	98	14.1	12.8	1.9	0.8
69	F	151.1	60.2	26.37	35.7	90.5	154	79	104	63	28.1	7.8	4.2	1.2	0.7	29	30	27	247	193	322	35	116	5.6	55.8	536	15.2	47.5	20.4	240	16.9	0.63	142	4.6	102	9.4	4.6	87	11.2	14.4	3.1	1.3
67	M	167.8	54.6	19.39	16.5	75.5	139	68	91.67	54	8.3	7.3	3.7	1	0.6	33	28	163	476	190	78	64	105	5.7	56.7	417	13.3	39.5	19.5	312	15.4	0.77	142	3.8	103	8.8	4.4	99	13.6	16.6	2.2	2.4
63	M	162.1	71.7	27.29	29.4	91.8	121	67	85	66	15.6	7.4	4.2	1.3	0.7	21	33	28	311	228	121	50	157	6.7	65.6	500	16.1	47.5	23.6	302	10.9	0.64	143	3.8	102	9.3	4.4	63	17.2	17.1	0.8	1
58	F	158.5	53.1	21.14	25.9	82	117	69	85	57	27	7.8	4.5	1.4	1.1	19	17	14	240	204	34	83	102	5.4	55.4	470	14.4	42.4	25.5	246	9.6	0.51	141	3.8	105	9.1	3	65	9.6	11	1	1
42	M	171.7	85	28.83	32.4	97.5	147	86	106.3	72	2.4	7.8	4.5	1.4	0.8	25	38	162	272	191	550	42	90	5.1	93.1	505	17.2	48.4	29.8	237	9.6	0.74	140	3.9	102	9.4	6.3	53	13.1	15.3	0.8	0.7
37	M	174.1	63.9	21.08	23.5	81.3	111	66	81	68	2	8.2	4.9	1.5	0.5	21	20	25	243	220	572	40	87	5.3	105.2	525	16.3	49.1	16	226	14.6	0.72	140	4.1	100	10.2	6.6	61	8.9	10.2	0.9	0.7
53	M	174.5	65.9	21.64	18.2	77.8	147	89	108.3	68	7.5	6.3	4.1	1.9	1.7	21	21	42	191	197	57	95	80	4.9	42.1	468	15.5	46	18.1	213	12.8	0.79	143	3.9	108	9.1	4.9	99	12.5	13.9	1	0.8
61	F	159.6	45.5	17.86	19.4	68.7	94	49	64	61	4.4	8	4.7	1.4	0.7	24	29	48	248	273	108	72	178	5.6	65.9	469	14.1	42.3	23.4	271	17	0.7	144	3.8	104	10.4	6.6	53	10.2	11.4	0.8	0.9
65	M	172.4	64.3	21.63	16.5	82.5	135	71	92.33	48	30.6	7.1	4.2	1.4	0.6	17	11	34	172	232	122	78	132	5.4	51.2	412	13.5	40.8	25.6	288	22	0.77	142	4.1	104	9.8	6.7	97	11.4	8.4	2.2	1.1
78	F	146.7	48.4	22.49	30.6	90.5	120	60	80	66	41.4	7.7	4.3	1.3	0.5	19	11	19	211	205	92	76	103	5.6	86.5	409	12.6	38.3	30.5	406	13.7	0.71	140	4.2	103	9.4	5.8	97	12.1	14.2	3.1	3.1
74	F	153.7	59	24.97	33	93	111	58	75.67	51	33.9	7.6	4.3	1.3	0.8	18	18	20	242	252	200	48	144	6.2	69.6	414	12.9	38.7	19.5	253	17.4	0.83	143	4.2	106	9.8	5	58	14	16.4	2.8	2.5
63	M	162.9	59.4	22.38	14.4	78	128	73	91.33	57	12.6	6.8	4.1	1.5	1.1	26	19	27	224	247	106	67	157	5.9	72.3	480	15.2	44.5	22.3	250	16.7	0.83	141	3.8	104	9.3	5.9	72	7.9	9.3	1.7	2.8
59	F	155.7	52	21.45	29	92.5	120	60	80	60	15.3	6.9	4.3	1.7	0.7	22	20	37	262	189	76	76	89	5.7	42.7	407	12.3	36.7	25.9	360	19.4	0.69	141	4.2	101	9.8	5.1	117	8.5	8.5	1.7	2.4
62	M	172.9	74.9	25.05	22.5	87.5	165	92	116.3	66	19.4	6.6	4.2	1.8	0.6	20	19	23	190	181	87	56	103	5.5	72.6	507	15.4	44.9	20.8	253	17.8	1.04	141	4.2	104	9.2	7.5	55	17.9	19.9	0.8	1.1
59	F	142.6	56.8	27.93	33.4	84	126	74	91.33	68	7.5	7.2	4.4	1.6	0.7	31	30	13	170	275	62	103	147	5.6	50.1	438	14.1	40.6	29.4	271	13.5	0.56	143	4.2	104	9.4	4.2	99	15.5	17.7	0.8	0.8
57	F	158	57.6	23.07	29.6	82.8	107	76	86.33	63	12.6	7.3	4.2	1.4	0.5	22	18	38	178	275	75	65	186	5.4	46.1	433	13.8	41.4	24.7	271	9.2	0.59	143	4.3	104	9.5	5	88	12.5	14.4	0.8	0.7
69	F	150.1	45.1	20.02	20.2	75	145	95	111.7	76	89	6.9	4	1.4	1	32	21	88	193	226	62	79	121	6.4	49.1	357	10.9	33.5	23.7	275	14.1	0.67	142	4.1	104	9.4	5.9	62	11.6	9.4	2.4	1.9
67	F	138.9	41.8	21.67	24.4	81	110	56	74	55	15.4	7.1	4.4	1.6	0.6	16	11	10	137	220	95	63	115	5.4	38.3	371	12.2	36.2	21.4	268	15.9	0.52	144	4	105	9.3	3	51	11.6	9.7	0.8	1.4
72	M	167.3	61.3	21.9	15.7	81	116	68	84	61	8.2	7.3	4.3	1.4	0.6	28	17	14	174	240	115	58	153	5.4	43.6	434	14.3	41	21.7	284	18.6	0.77	142	4.2	105	9	6.5	77	9.8	11.9	1.1	0.9
46	F	159.5	59.8	23.51	31.1	82.5	104	58	73.33	59	24.6	6.9	4.1	1.5	0.6	17	13	17	122	209	80	47	138	5	40.6	411	12.3	36.9	25.1	220	15	0.62	139	4.3	104	9.3	5.1	90	9.2	10.3	0.7	0.7
65	M	163.5	57.4	21.47	20	80.5	139	72	94.33	58	12.9	7.2	4.2	1.4	0.6	30	27	33	202	179	65	64	102	5.1	67.5	445	15.8	45.8	20.2	240	18.7	0.5	141	4.2	102	9.3	7.4	68	11.6	12.9	1.2	1
65	F	155.4	62.1	25.72	30.7	94.5	136	86	102.7	67	36.5	7.6	4.5	1.5	0.8	18	16	19	216	240	88	77	133	5.4	35.1	436	14.1	41.6	15.3	220	19	0.56	142	3.8	104	9.6	5	45	13.4	15.2	0.7	1
75	F	148.5	37.4	16.96	17.6	70.6	120	57	78	72	38.3	7.3	4.2	1.4	0.6	20	12	17	122	218	112	68	105	5.6	48.2	394	12.4	37.6	17.6	250	23.4	0.72	143	4.3	105	9.4	5.4	137	9.1	8.3	1.2	1.2
73	M	166.5	61.1	22.04	18.5	82.5	123	79	93.67	58	33.3	7.1	4.2	1.4	0.6	18	13	23	104	190	50	99	60	5.5	36.6	415	14.4	41.5	17.3	260	12.4	1	144	4.2	107	9	5	79	9.1	9.4	0.8	1
87	M	154.1	48.8	20.55	17.6	74	115	63	80.33	58	80.7	6.7	4.1	1.6	0.8	18	14	38	271	166	88	60	76	5.9	74.7	491	14	42.3	17.5	293	11	0.83	140	4.2	99	9.2	6.2	70	10.7	10.4	1.9	2.5
83	F	132.9	37.1	21.01	28.8	76	116	69	84.67	67	18.7	8.2	4.7	1.3	0.5	27	12	17	202	209	76	57	132	5.1	59.6	457	14.6	43.4	21.2	323	17.3	0.44	144	4.5	101	10	3.8	75	10	9.7	1.4	1.3
77	M	158.9	57.5	22.77	16.9	78.6	150	75	100	61	8	7.7	4.4	1.3	0.9	25	27	41	232	208	78	51	135	4.6	52.9	400	13.8	39.4	16.2	231	18.5	0.97	144	3.9	102	9.3	5.6	122	8	8.5	1.3	2
57	M	176.1	67.7	21.83	21	83	108	76	86.67	55	9.4	7.4	4.2	1.3	0.5	23	23	46	262	188	76	68	92	5	38.5	458	15.5	45.6	15.1	253	15.4	0.8	143	3.9	103	9.6	6.3	115	10.2	10.3	0.9	0.7
67	M	166.9	76.3	27.39	30.8	101	146	81	102.7	105	4.8	7.9	4.7	1.5	0.5	47	34	96	238	209	120	48	147	5.7	55.2	465	15.3	44.5	24.7	215	16.2	0.78	143	4.2	102	10	7.7	72	16.8	20.7	0.7	0.9
62	F	155.9	53.9	22.18	29.1	88.8	175	93	120.3	59	13.9	8	4.4	1.2	0.6	26	21	15	252	197	190	37	121	5.4	52	442	14.1	42.1	21.7	271	11.6	0.63	145	3.8	103	9.7	5	93	12.5	10.2	1	1.2
63	M	168.5	59.5	20.96	16	81.5	131	76	94.33	47	47.3	6.5	4.3	2	0.8	18	17	45	194	228	210	54	117	5.4	47.8	470	15.7	44.8	17.4	226	13.4	0.74	145	3.9	103	9.6	5.8	86	10.9	9.6	2.5	0.8
61	F	149.7	63.5	28.34	38.8	94.3	139	79	99	67	23.8	8.6	4.7	1.2	0.7	19	14	25	364	222	118	63	142	5.6	79.7	442	13.8	41.6	31.6	317	11.6	0.52	141	4.3	99	10.3	4.1	63	15.9	14.6	1.8	2.7
73	M	163	64.2	24.16	21.2	83.6	151	84	106.3	89	10.9	7.7	4.6	1.5	0.8	18	15	28	191	175	74	49	98	5.1	62.1	457	14.6	43.6	18.6	257	18.6	0.81	141	4.3	102	9.5	5.3	97	13.8	14	1.6	1.3
61	M	174.6	71.3	23.39	26.7	82	132	87	102	62	6.2	8	4.8	1.5	0.9	46	43	200	243	251	262	57	151	5.4	66.2	432	15.1	44.3	22.9	250	13.6	1.02	141	4	104	9.7	5.4	54	17.3	16.2	1.8	1.8
71	M	167.3	69.4	24.8	22.3	96	137	87	103.7	63	40	7.6	4.6	1.5	1.3	23	14	34	260	179	105	62	90	6	54.9	499	15.7	46	19.7	246	11.7	0.75	143	4.5	104	9.8	5.1	53	13.4	13.8	0.8	0.9

63	F	154.1	62.5	26.32	35.2	92.5	132	64	86.67	78	12.4	7.9	4.6	1.4	0.4	20	15	14	272	217	88	57	129	6.7	84.2	478	13.5	41.9	27.4	307	14.1	0.73	141	4.1	101	9.8	5.3	103	15.3	14.6	0.9	0.7
68	F	146.2	37.7	17.64	17.4	61.5	132	69	90	77	15.8	7.2	4.3	1.5	0.6	23	14	18	124	261	67	84	138	5.1	46.1	400	12.5	38.3	23.1	260	19.4	0.48	143	3.7	101	9.7	4.8	101	9.6	9.3	0.9	0.8
64	F	148.9	47.6	21.47	27.7	69.3	117	72	87	64	16.5	7.6	4.4	1.4	0.6	30	28	19	421	158	83	53	81	5.6	46.4	453	14.2	43.3	27.3	213	15.6	0.58	142	4.3	101	9.6	5	147	15	14.8	1.2	0.9
65	M	178.9	70.7	22.09	17	84.4	126	76	92.67	58	10.7	7.3	4.5	1.6	0.9	23	19	20	186	198	114	51	123	5.9	50	485	15	44.5	21.9	234	12.9	0.77	144	3.8	101	9.5	4.8	82	9.6	8.6	1	0.9
66	F	150.4	44.1	19.5	22.4	77	126	62	83.33	95	17.4	7.3	4.3	1.4	0.9	20	22	41	268	260	133	79	129	5.4	48.1	458	14.9	44.5	17.7	302	16	0.66	144	3.9	101	9.5	4.5	109	22.2	18.8	0.6	0.8
56	F	159.3	58.6	23.09	34	89.7	116	68	84	68	10.6	7.9	4.5	1.3	0.5	19	16	16	219	232	56	64	142	5.7	65.6	455	14.3	42.1	23.3	288	13.8	0.55	145	4.4	102	9.8	5.1	45	10.9	10.3	0.8	0.8
65	M	171.7	68	23.07	19.8	84.6	102	64	76.67	55	4.7	7.1	4.4	1.6	0.8	23	21	40	248	182	99	47	109	5.1	48.9	483	15.9	45.4	22.1	240	21	0.99	142	4.3	99	9.2	5.5	95	11.3	8.5	0.9	0.9
58	M	173.5	70.9	23.55	25.6	90.6	111	71	84.33	58	4.8	6.9	4.5	1.9	0.7	27	43	116	178	224	134	45	150	5.6	59.3	515	15.4	45.6	22.3	243	10.8	0.84	142	4.1	101	9.2	5.3	78	13.9	12.1	0.7	0.6
55	F	157.3	43.4	17.54	21.4	73	148	83	104.7	58	13	7.7	4.7	1.6	0.9	25	24	13	261	275	104	64	184	4.9	45.1	451	14.9	42.1	16.2	264	13	0.46	146	4.2	103	9.7	3.8	177	13.5	13.5	1.4	0.8
42	M	165.3	59.9	21.92	25.2	82.8	119	76	90.33	59	4.3	7.3	4.6	1.7	0.3	76	98	36	261	283	66	51	236	5.6	71.8	478	14.8	44.3	30.8	293	20.3	0.9	144	4.1	103	9.3	7.2	99	10.2	10.1	0.7	0.8
61	F	151.8	50.5	21.92	28.1	79.8	126	80	95.33	70	3.8	7.1	4.4	1.6	0.7	20	20	30	193	167	82	51	100	5.4	58.1	421	13.3	39.8	20.3	228	15.9	0.68	145	3.5	105	9.5	4	96	12.4	8.9	0.9	0.8
61	F	145.8	42.8	20.13	22.9	76.5	117	77	90.33	84	11	7.6	4.7	1.6	0.6	21	17	31	235	215	93	40	153	5.8	38.7	420	13	38.2	23.2	257	14	0.43	143	3.7	103	9.5	4.1	56	8	8	0.9	0.8
59	M	162.4	58	21.99	26.1	87	105	76	85.67	75	7.7	7.8	4.6	1.4	0.4	37	31	30	160	202	135	36	145	7.3	53.3	481	15.2	45.6	23.8	280	11.2	0.97	141	4.4	103	9.8	7.5	84	11.6	13.2	0.7	0.7
68	F	145.3	49.3	23.35	29.3	87.2	135	77	96.33	80	10	7.3	4.4	1.5	2.1	31	31	29	204	191	99	60	102	5.4	42.5	407	13.1	38.9	22.9	317	10.5	0.59	144	3.8	106	8.9	4.3	80	16.2	16.7	0.8	0.6
67	M	162.8	54.5	20.56	15	75.5	125	73	90.33	70	38.3	7.1	4.1	1.4	0.8	25	24	37	162	179	85	74	87	5.7	34.1	499	14.7	43.9	16	189	12.7	0.67	143	3.8	104	9.2	4.8	57	9.2	9.5	0.9	1
59	M	171.5	68.7	23.36	23.7	90.3	152	88	109.3	68	7	7.5	4.5	1.5	0.8	26	30	83	240	218	138	60	139	5.7	58.3	461	15.4	44.6	20.5	243	10.4	0.78	141	3.9	102	9.6	7	54	11.7	12.7	0.8	0.9
66	M	157.6	62.9	25.32	28.1	90	101	62	75	69	13.9	7.9	4.4	1.3	0.6	25	21	78	206	230	64	74	133	5.5	52.7	411	14.4	42	23.5	250	12	0.74	140	4.6	102	9.5	5.9	58	9.5	9.4	2	1.4
55	F	152.9	64.9	27.76	40.1	103	126	76	92.67	76	7	7.6	4.5	1.5	2.2	18	18	20	217	199	83	50	128	5.7	61.6	482	14.6	43.6	21.3	250	12.2	0.58	143	4	104	9.8	6.8	48	20	19	0.9	1.2
52	M	179.8	93	28.77	32.8	102	141	85	103.7	71	4.4	7.8	4.7	1.5	0.5	25	31	27	176	226	110	43	163	5.7	78.6	515	15.3	45.6	26	253	18.3	0.94	143	4.7	107	9.8	5	63	16	16	0.9	1.2
55	F	161.4	57	21.88	30	87.7	145	88	107	77	7	8	4.4	1.2	0.8	21	18	16	274	184	69	68	96	5.1	47.8	475	14.3	41.8	19.9	260	12.6	0.54	142	3.6	103	9.4	6.2	60	16	16	0.8	0.9
52	F	161.7	52.8	20.19	25.6	73	102	58	72.67	59	5.8	7.8	4.6	1.4	0.7	23	21	60	267	185	34	79	85	5.4	46.6	432	13.8	41.9	22.9	297	19.3	0.61	140	3.8	101	10.1	2.8	122	14	11	0.8	1.4
66	M	155.7	64.9	26.77	34.7	96.5	109	74	85.67	56	4.3	7.1	4.7	2	0.6	24	16	28	231	230	95	59	145	6.1	87	512	16.5	48.1	19.5	297	22.1	0.89	143	3.8	102	10.1	5.7	88	15	12	1.6	2.4
63	M	164.1	65.4	24.29	20.4	79.8	113	71	85	57	5.7	7.5	4.2	1.3	0.5	43	23	147	213	164	84	88	55	5.5	31.4	473	15.2	44.5	21.2	223	18.1	0.83	142	5	103	8.9	6.7	91	10	10	0.8	1
67	F	155.3	45.7	18.95	25.1	83.5	127	63	84.33	93	17.8	7.8	4.5	1.4	0.6	23	33	29	246	249	116	53	161	6.4	42.8	425	13.9	40.1	27.2	284	13.1	0.55	143	4.3	104	10	4.3	52	13	13	1.2	1
69	M	167.7	68.1	24.21	29	92	130	80	96.67	77	6.9	7.7	4.6	1.5	0.9	23	23	24	123	250	156	54	167	6.6	76.6	463	14.6	42.9	23.9	275	19.8	1.06	140	4.3	100	10	7.9	67	13	12	0.8	0.8
43	M	178.1	100.4	31.65	33.4	105	141	82	101.7	63	12.2	7.6	4.3	1.3	0.7	21	22	79	201	222	240	39	132	5.3	69	502	16	47.4	31.2	271	14.8	0.9	141	4.2	104	9.6	6.3	84	15	16	0.7	0.8
67	M	168.2	71.3	25.2	20.8	87.3	118	70	86	73	3.4	7.4	4.7	1.7	0.9	25	24	55	229	162	160	47	95	5.2	78.1	492	16.8	48.3	24.7	317	11	0.8	144	4.3	105	10.2	5.3	93	8	10	2	2.3
68	F	152.1	59.5	25.72	31.4	92	158	81	106.7	68	6.2	7.4	4.2	1.3	0.4	32	30	18	71	158	72	53	84	5.6	41.6	384	11.8	34.4	34.6	210	11.9	0.58	143	4.6	107	9.4	3.2	50	11	13	1	1.4
62	F	154.1	61.1	25.73	38.9	95	112	63	79.33	59	10.4	7.7	4.3	1.3	0.9	23	20	16	164	234	54	86	122	6	43.9	444	14.3	41.6	24.7	297	20.8	0.66	143	4.1	103	9.3	4.6	50	14	19	1.3	0.8
62	F	156.9	64.8	26.32	35.5	87.5	106	71	82.67	69	5.3	7.4	4.6	1.6	0.5	16	13	17	238	198	113	45	130	5.6	42.3	475	14.5	43.2	24.1	260	13	0.57	143	4.2	106	9.4	3.2	72	13	12	0.6	0.5
73	F	151.5	66.7	29.06	38.4	103.5	140	71	94	62	83.7	7.4	4.4	1.5	1.2	20	16	22	184	227	82	68	127	5.4	76.3	456	14.2	42.3	19.4	302	14.6	0.62	143	4.2	103	9.9	5	83	10	13	1.6	1.2
58	F	148.7	59.1	26.73	38.4	92.7	128	67	87.33	59	22	7.5	4.6	1.6	0.8	17	23	25	306	204	109	69	104	5.4	49.5	482	14.9	43.3	24.8	268	15.2	0.61	142	4	101	9.9	4.9	96	17	18	1.2	1.2
62	F	151.6	56.5	24.58	38.9	81	117	60	79	62	13.8	7.6	4.4	1.4	1	23	21	20	145	195	102	38	126	6	49.2	429	12.8	38.6	31.4	312	13.1	0.53	141	4	103	9.3	3.9	77	14	14	1.8	1.1
52	M	163.3	77.9	29.21	33.1	94.6	164	96	118.7	90	4.2	8.5	4.6	1.2	0.6	25	27	43	205	193	93	59	111	5.4	54.4	501	16.1	45.7	30.3	340	12.7	0.74	142	4.2	103	9.7	6.6	34	16	17	1	0.7
34	F	149.2	44.3	19.9	23.5	72.6	111	60	77	84	6.1	7.4	4.5	1.6	0.7	12	10	11	153	206	109	70	107	5.3	84.5	448	13.3	38.6	30.6	231	16.6	0.66	139	3.7	105	9.1	5.6	123	15	18	0.9	0.6
73	M	157.9	59.7	23.94	28.9	90	123	75	91	68	45.5	7.7	4.7	1.6	0.6	33	48	23	226	233	184	43	165	9.7	59.3	506	15.1	44.4	18.8	213	18.6	0.88	140	4.3	100	9.8	3.5	83	15	13	1.3	1.3
71	F	144.5	49.3	23.61	47.6	89	145	78	100.3	71	11.6	7.3	4.7	1.8	0.6	22	21	38	286	160	94	43	88	6.5	66.8	463	13.9	42.4	23.8	257	17.3	0.64	144	4.9	105	10	4.4	77	13	15	1.4	1.7
68	M	150.2	56.4	25	27.8	84.2	151	86	107.7	58	15.3	7.4	4.6	1.6	0.9	28	13	27	115	188	101	55	116	5	61.1	526	16.2	46.2	22.6	231	17.3	1.97	141	4.2	103	9.6	5.7	106	16	14	0.8	0.7
65	F	152.4	49.7	21.4	29.7	85.3	170	90	116.7	74	19.8	8.2	4.9	1.5	0.5	19	16	51	306	236	132	52	159	5.5	75.1	480	14.2	42.1	30.7	293	14.1	0.54	143	4	103	10.1	3.8	100	17	18	1	1.4
68	F	150.5	50.3	22.21	23.7	88.3	129	85	99.67	62	5.1	7.6	4.6	1.5	0.5	24	21	21	196	246	146	67	127	6	50.2	469	14.9	44.7	25	275	11.3	0.59	144	3.8	105	9.8	4.4	57	13	15	1.1	1.1
70	M	170.1	69.3	23.95	17.7	88.5	134	77	96	68	13.5	7.3	4.5	1.6	0.9	25	19	37	210	257	105	67	154	5.5	62	491	15.2	44.9	22.7	243	14.5	0.81	142	4.1	103	9.3	5.5	76	11	12	0	0
70	M	153.3	60.3	25.66	27.1	84.3	116	61	79.33	78	4.1	7.9	4.3	1.2	0.6	25	26	47	220	180	151	47	108	6.2	66.8	521	16.8	47.9	24.1	568	14.2	0.74	140	4.3	101	9.7	4.8	72	13	10	0.9	0.9
82	F	146.5	51.4	23.95	31.2	91	128	79	95.33	67	25.4	7.5	4.5	1.5	0.5	17	13	26	213	232	138	53	150	5.9	65.3	470	14.4	42.9	20.1	260	20.8	0.59	143	3.9	105	9.6	4	96	13	12	1	1.3
54	F	155.6	45.3	18.71	22.6	73	145	85	105	84	6.7	7.8	4.4	1.3	0.6	19	15	15	250	271	104	66	152	5.2	29.3	447	14.2	40.8	22.2	302	14	0.64	143	3.9	104	9.5	5.4	64	12	14	1.1	0.7
70	M	163.2	70.8	26.58	33.5	100	106	66	79.33	68	12.2	7.4	4.3	1.4	1	26	26	78	198	191	75	42	119	5.6	53.8	514	15.7	46.1	19.8	223	25.9	0.84	141	4.1	102	9.9	5.3	100	11	12	1.7	1.9
64	F	150.5	58.2	25.7	37.6	96.5	123	62	82.33	65	5.8	7.7	4.3	1.3	0.8	32	39	46	261	220	55	75	116	6.4	54.4	460	14.1	42.2	26.2	250	24.5	0.55	142	4.4	105	9.8	5.5	75	17	16	1	0.8
63	F	152	49.1	21.25	24.2	79	147	88	107.7	70	23	7.3	4.5	1.6	0.6	23	17	16	254	289	76	92	154	5.4	76	463	14.5	43.5	20.9	302	19.2	0.59	141	3.8	102	9.6	4.8	89	15	16	0.8	1
63	M	170.1	72.9	25.2	25.7	87.3	148	106	120	66	13.1	7.8	4.6	1.4	0.9	21	26	76	118	226	188	58	144	5.6	54.9	519	15.5	46.5	24.1	271	13.9	0.91	141	3.7	99	9.3	7	101	11	13	0.9	0.9
73	F	149.7	42.7	19.05	23.7	66.5	158	80	106	61	54.1	7.8	4.7	1.5	0.5	28	21	20	231	245	59	77	148	5.3	31.8	438	13.7	41.7	20.6	302	18.6	0.69	143	4.5	104	9.3	6	99	18	20	1.6	1.6
74	F	147.4	39.9	18.36	22.4	75	127	67	87	81	44.3	8.2	4.5	1.2	0.5	22	13	26	278	220	61	73	127	5.2	50.6	424	13.4	40.9	29.5	407	10.6	0.44	140	3.8	100	9.4	4.2	84	10	10	0.6	0.6
67	F	160	37.3	14.57	12.3	55.5	110	53	72	56	10.9	7.7	4.4	1.3	0.5	27	16	22	198	184	66	66	98	6	60.2	533	16.4	48.7	17.9	264	14.2	0.42	143	5.1	102	9.7	4.1	102	12	10	1	1.6
54	M	168.5	64.9	22.86	24.9	83.8	132	84	100	66	2.5	7.7	4.7	1.6	1.1	34	48	37	245	209	140	42	155	5.3	59.4	559	17	48.7	16.2	227	13	0.89	142	4.1	104	9.7	5.3	80	14	13	0.6	0.5
62	M	164.6	56.9	21	21.8	77	141	93	109	65	28.8	7.7	4.5	1.4	0.7	20	16	28	309	199	97	63	109	4.7	63.1	468	16.1	47	23.7	357	13.3	0.6	142	4.2	99	9.8	5	65	15	19	0.6	0.6
63	F	156.5	58.2	23.76	33.6	94.5	135	86	102.3	98	8.9	7.9	4.6	1.4	0.7	30	28	21	414	208	169	54	105	7.7	82.4	485	14.8	43.6	24.8	286	15.5	0.77	142	3.9	99	9.8	3.9	96	15	16	2.3	1.9
76	M	153.3	52.2	22.21	16.4	82	147	84	105	51	20.3	7.3	4.5	1.6	0.9	28	21	24	175	223	58	82	118	5	55.8	457	14.8	42.6	17.7	242	18.5	1.02	143	4.3	104	9.7	6.4	117	14	15	1.2	2.9
27	M	172.9	65.5	21.91	19	81.3	107	62	77	69	2.7	7	4.6	1.9	0.5	15	9	24	150	186	114	34	133	4.9	46	558	17.2	48.1	17.8	159	11.6	0.75	143	4.4	106	9.1	6.4	46	10	11	0.7	0.6
70	M	170.2	77.9	26.89	31.6	100.7	159	96	117	75	112	7.1	4.1	1.4	0.7	20	14	50	299	204	168	45	142	5.6	96.4	541	13.7	42.4	25.1	420	21	0.95	142	4.2	103	9.5	6	89	13	13	3.2	0.9
73	F	150.7	50.5	22.24	31.8	83.8	154	84	107.3	61	31.4	6.8	4.1	1.5	0.6	29	19	35	157	216	122	65	122	5.7	68.9	468	14.2	42.7	26.2	264	20	0.63	144	4.4	103	9	5.1	88	9	8	0.9	0.8
64	F	149.3	42.2	18.93	21.6	72.5	97	56	69.67	73	21.1	8.1	4.4	1.2	0.3	35	27	15	274	214	56	66	130	5.4	59.1	426	13	39.3	26.9	301	17.9	0.57	140	3.4	98	9.3	5.2	91	12	11	0.9	0.8

79	F	144.4	44.7	21.44	21.4	87.7	127	55	79	87	18.7	7.7	4.1	1.1	0.6	19	13	12	263	187	149	51	102	6.9	45.1	415	13.1	40	24.5	306	13.6	0.63	145	3.8	104	9.2	3.5	81	9	10	1.1	3.1
75	M	157.8	60.3	24.22	27.3	83.2	143	78	99.67	53	48.1	7.4	4.4	1.5	0.4	28	20	21	160	184	60	67	90	5.3	48.2	466	13.9	41.2	20.3	330	14.6	0.57	140	4	102	9.3	5.1	62	17	15	2.7	2.1
74	F	146.6	39.7	18.47	22.7	77	129	73	91.67	55	11.1	7.4	4.3	1.4	0.4	18	16	21	190	191	198	40	112	6	61.6	449	13.9	40.3	24.9	318	13.2	0.51	142	3.7	100	9	4	78	13	12	0.8	1.4
71	M	161.3	57.5	22.1	17.9	81.8	152	75	100.7	79	13.6	7.3	4.5	1.6	2.3	37	41	64	233	178	149	80	68	4.9	34.8	351	13.4	38.9	12.4	209	16.9	0.56	141	3.9	102	8.9	4.1	47	9	10	1.7	0.8
83	M	158.9	51.6	20.44	20.1	78.5	133	55	81	53	12.5	7.7	4.6	1.5	0.7	22	13	23	228	184	120	51	92	6.3	58.8	447	13.5	39.7	21.4	291	15.6	0.9	141	4.5	102	9.7	4.8	62	14	12	1.1	2.7
75	F	153.3	45	19.15	24.4	79.7	139	79	99	70	28.7	7.6	4.3	1.3	0.6	21	14	17	178	172	132	54	82	5.7	62.5	393	12.4	38.7	19.5	383	19.3	0.86	144	5	105	9.3	7.3	74	10	12	1.4	1.3
69	M	153.3	54.6	23.23	20.2	84.7	131	76	94.33	53	7.5	7.5	4.3	1.3	0.8	27	24	27	289	211	106	46	140	5.2	75.9	479	16.2	48.2	21.4	268	18.7	0.97	144	3.9	106	9.3	6.8	97	10	10	1.4	1.1
64	M	171.5	56.4	19.18	15	76	106	69	81.33	63	6.7	7.4	4.6	1.6	0.6	31	24	40	133	227	73	94	111	6.1	43.7	438	15.3	44.8	20.2	245	11.8	0.76	142	4	100	9.3	3.7	81	20	12	3.4	3
47	M	164	70.9	26.36	31.5	88.5	131	92	105	66	4.5	7.3	4.7	1.8	0.2	37	58	146	216	160	242	54	78	7.6	69.9	532	16.6	48.3	26.1	221	16.3	0.67	141	4.7	103	9.3	6.3	37	18	17	0.9	0.9
68	F	148.5	44.7	20.27	25	76.7	171	95	120.3	65	17.8	7.8	4.4	1.3	0.7	22	16	21	234	291	146	60	182	5.5	71.2	428	13.3	39.7	23.7	318	17.8	0.74	140	3.8	100	9.4	4.8	155	11	14	1.2	1.6
60	M	184.1	90	26.55	26.2	102.3	121	80	93.67	57	13.7	7	4.2	1.5	0.6	34	49	88	159	205	196	52	122	6.3	53.5	501	16.5	46.9	16.6	242	12.8	0.99	142	4.1	104	9.2	5.7	46	9	11	0.8	0.9
68	M	169.7	67.3	23.37	18.1	87	124	78	93.33	61	27.9	7.5	4.4	1.4	0.4	22	20	27	174	186	66	59	96	5.6	86.7	507	14.7	43.6	21.2	343	17.8	0.84	141	4.3	102	9.2	6.3	81	12	12	1.2	0.9
66	F	153.3	52.1	22.17	26.7	83.4	118	62	80.67	57	8.6	8.1	4.6	1.3	0.8	20	13	14	307	253	59	75	156	5	44.3	473	14.1	41.7	19.5	242	18.8	0.52	141	4.3	101	9.9	6.3	71	14	13	0.9	1.3
70	M	176.1	61.2	19.73	17	81.3	125	53	77	50	27.4	7.3	4.4	1.5	0.8	26	16	18	208	201	78	72	115	5.2	45.3	456	14.7	43.3	18.6	256	14.1	0.79	141	4.2	101	9.4	6.2	64	8	11	1	1.3
67	F	149.1	56.3	25.33	32.9	87.8	108	63	78	49	15.2	7.9	4.6	1.4	0.7	19	16	27	235	240	88	74	143	5.4	37.6	448	14.5	42.8	18.1	238	12.4	0.49	143	4	103	9.3	4.9	63	13	14	0.8	0.9
66	M	170.5	75.6	26.01	26.1	95	140	76	97.33	68	8	7	4.5	1.8	0.8	21	26	44	223	168	172	77	56	5.3	55.1	459	14.9	43.4	28.3	252	15.9	0.59	143	4.1	101	9.2	4.8	44	14	13	2.1	0.9
73	F	149.1	42.4	19.07	22.2	73	139	97	111	86	42.7	7.7	4.3	1.3	0.8	25	16	37	140	209	61	87	92	4.7	44	411	14.1	41.7	18	224	12.6	0.57	144	3.9	105	9.1	5.2	124	18	16	0.9	0.8
72	M	172.2	77.1	26	28.8	96.5	135	80	98.33	52	33.2	7.9	4.3	1.2	1.4	23	16	28	159	212	65	59	141	5.3	53.2	460	15	44	26.1	296	15	0.89	140	3.9	100	9.2	6	99	12	13	0.9	0.8
70	F	152.1	43.3	18.72	26	86.8	146	85	105.3	83	17.4	7.6	4.3	1.3	0.6	21	13	21	321	226	87	81	99	6	56.9	478	13.8	42.2	24.1	264	15	0.35	142	3.9	103	9	2.7	69	12	12	2.8	1.7
68	M	168.2	64.8	22.9	20.7	88.5	136	88	104	64	26.4	6.7	4	1.5	0.9	18	17	34	205	202	101	50	137	4.8	39.9	431	13.6	40.3	24	238	13.2	0.83	144	3.9	107	9	7.5	77	14	12	0.9	1.3
63	M	167.4	63.3	22.59	22.8	89.5	125	78	93.67	55	5.4	8	4.4	1.2	0.9	27	21	31	214	197	131	49	114	5.2	63.9	450	13.7	40.8	29.3	312	16.5	0.72	142	4.3	104	9.4	6.6	75	14	12	2.4	3.3
78	F	155.6	49.1	20.28	22.7	73.5	135	63	87	84	60.3	7.4	4.3	1.4	0.8	23	24	21	193	154	130	63	59	5.5	46.3	456	14.7	43.5	17	238	18.6	0.6	145	4.3	102	9.6	5.2	56	14	17	0.7	1
70	F	154.5	60.9	25.51	32.3	100	132	76	94.67	68	27.4	6.9	4	1.4	0.5	15	13	40	348	187	86	73	88	5.4	48.4	475	14	41.3	24.7	202	16.6	0.58	143	4.1	106	9.2	4.3	43	9	10	0.9	1.3
42	M	172	66.6	22.51	16.9	77.3	154	92	112.7	56	24.3	7.9	4.6	1.4	0.9	18	15	61	189	209	160	67	110	4.9	42.2	522	16.4	46.5	21.6	281	16	0.93	141	3.9	99	9.4	7	58	22	21	1	0.9
60	M	168.7	71	24.95	26.7	93.5	142	83	102.7	63	7.8	7.6	4.1	1.2	0.9	17	13	23	212	232	210	58	114	5.5	56	481	15.5	45.1	26.3	330	16.9	1.03	142	4	104	9.6	7.8	55	11	13	2.1	2.7
55	F	151.7	46.2	20.08	25.3	74	117	65	82.33	60	2.7	7.1	4.4	1.6	0.8	18	17	15	218	265	54	88	150	5.6	42.1	502	15.2	44.5	21.3	216	16	0.59	142	3.7	102	9.8	3.9	102	12	11	0.8	0.8
46	F	160	64.8	25.31	31.3	91.5	133	90	104.3	65	11.1	7.5	4.2	1.3	0.6	19	16	27	126	184	62	68	101	5.6	69.8	451	8	28.2	24.2	221	15.4	0.63	141	4.6	104	9.5	5	64	14	12	0.7	0.8
61	M	162.1	51.6	19.64	16.4	74.6	116	75	88.67	68	8.1	7.5	4.3	1.3	1.2	24	14	16	162	214	63	73	118	5.7	27.7	469	15.4	46.5	18	286	20	0.85	143	5	103	9.3	5.9	120	8	13	0.9	1.5
70	M	168.1	68.7	24.31	24.5	86.5	151	81	104.3	69	24.8	7.5	4.4	1.4	1.5	27	16	44	169	196	76	67	106	6.2	58.7	443	14.3	41.9	18.8	318	22.4	0.85	142	4.3	104	9.5	7.6	43	13	15	2.5	3.6
62	M	181.3	89.6	27.26	29.5	99.8	138	89	105.3	50	9.9	7.3	4.1	1.3	0.7	18	15	24	171	213	133	50	144	5.1	62.3	488	14.9	42.9	28	227	11.3	1.07	143	4.3	105	9.4	7	82	10	14	1.9	0.8
38	M	173.3	61.4	20.44	22.5	76.5	119	72	87.67	51	6	7.5	4.6	1.6	1.1	23	39	120	247	219	108	50	147	5.1	56.1	521	15.8	45.7	17.3	264	11.9	0.98	143	3.8	102	9.5	6.3	58	14	13	0.7	0.8
83	M	154.6	59.6	24.94	16.6	88	97	42	60.33	76	36.7	7.4	4.3	1.4	0.8	23	16	29	519	198	84	89	103	5.3	46.9	437	14.5	40.4	10.2	286	11.2	0.92	146	3.9	106	9.2	6.5	159	8	8	2.3	2.9
77	M	166.9	51.4	18.45	11.7	70	87	60	69	68	56.5	7.8	4.4	1.3	0.5	23	12	90	349	197	72	76	99	5.4	58.4	515	16.4	47.1	15	224	20.1	1	139	5.1	101	9.5	5.4	210	8	8	1	1.1
62	M	167.2	64.8	23.18	27	91.3	119	75	89.67	63	28	7.6	4.3	1.3	0.5	17	14	23	229	197	192	38	126	5.6	50.5	459	14.4	41.7	23.7	301	19.9	0.75	140	4.3	102	9.2	4.8	98	15	15	1.1	0.8
63	M	162.4	62.4	23.66	22.2	87.5	129	70	89.67	57	8	7.1	4.1	1.4	0.6	32	16	68	145	200	171	50	118	5.6	85.4	457	14.2	40.8	24	224	18.7	0.88	143	4.7	104	9.1	5	97	15	14	2.6	1.7
62	F	161.5	52.8	20.24	24.2	74	103	57	72.33	59	20.4	7.7	4.6	1.5	0.8	46	29	40	177	196	125	76	95	5.4	87.4	421	13.6	38.8	21.3	272	14.5	0.58	142	4.5	102	9.6	3.9	84	8	9	1	1.1
71	M	164.7	63.1	23.26	20.8	81.5	129	67	87.67	52	17.2	7	4.3	1.6	1.1	16	16	18	262	202	192	30	133	5.3	39.7	493	15.2	44.6	15.9	229	19.8	0.92	141	3.9	106	9	6.6	74	11	11	1.1	1.4
