# Supplementary material for: Systemic factors associated with intraocular pressure among subjects in a health examination program in Japan
Source: PLoS One. 2020 Jun 3;15(6):e0234042. doi: 10.1371/journal.pone.0234042 (PMC7269229; doi:10.1371/journal.pone.0234042)
Supplement: S2 Table — (PDF) [file pone.0234042.s002.pdf]

Table S2. Univariate analyses for possible correlations between each pair of parameters other than IOPs.

Parameter 1	Parameter 2	Spearman's ρ	p-value
Male=0/Female=1	Age	0.0564	0.0256*
Height	Age	−0.2711	<.0001*
Height	Male=0/Female=1	−0.7760	<.0001*
Weight	Age	−0.2388	<.0001*
Weight	Male=0/Female=1	−0.6049	<.0001*
Weight	Height	0.7109	<.0001*
BMI	Age	−0.1003	<.0001*
BMI	Male=0/Female=1	−0.1918	<.0001*
BMI	Height	0.1511	<.0001*
BMI	Weight	0.7838	<.0001*
%Fat	Age	−0.1019	<.0001*
%Fat	Male=0/Female=1	0.4983	<.0001*
%Fat	Height	−0.3694	<.0001*
%Fat	Weight	0.1827	<.0001*
%Fat	BMI	0.6022	<.0001*
Waist	Age	0.0009	0.9731
Waist	Male=0/Female=1	−0.3121	<.0001*
Waist	Height	0.3306	<.0001*
Waist	Weight	0.7535	<.0001*
Waist	BMI	0.783	<.0001*
Waist	%Fat	0.4164	<.0001*
SBP	Age	0.1637	<.0001*
SBP	Male=0/Female=1	−0.1667	<.0001*
SBP	Height	0.0916	0.0003*
SBP	Weight	0.2083	<.0001*
SBP	BMI	0.207	<.0001*
SBP	%Fat	0.0357	0.1581
SBP	Waist	0.2274	<.0001*
DBP	Age	−0.0046	0.8548
DBP	Male=0/Female=1	−0.2642	<.0001*
DBP	Height	0.2162	<.0001*
DBP	Weight	0.2951	<.0001*
DBP	BMI	0.2216	<.0001*
DBP	%Fat	0.0088	0.7276
DBP	Waist	0.2701	<.0001*

DBP	SBP	0.7639	<.0001*
MBP	Age	0.0698	0.0057*
MBP	Male=0/Female=1	-0.2367	<.0001*
MBP	Height	0.1738	<.0001*
MBP	Weight	0.2725	<.0001*
MBP	BMI	0.2263	<.0001*
MBP	%Fat	0.0186	0.4624
MBP	Waist	0.2656	<.0001*
MBP	SBP	0.9176	<.0001*
MBP	DBP	0.9546	<.0001*
Pulse	Age	0.1276	<.0001*
Pulse	Male=0/Female=1	0.2065	<.0001*
Pulse	Height	-0.2046	<.0001*
Pulse	Weight	-0.1306	<.0001*
Pulse	BMI	-0.0167	0.5076
Pulse	%Fat	0.1743	<.0001*
Pulse	Waist	0.0336	0.1832
Pulse	SBP	0.1223	<.0001*
Pulse	DBP	0.1375	<.0001*
Pulse	MBP	0.1401	<.0001*
BNP	Age	0.4011	<.0001*
BNP	Male=0/Female=1	0.1509	<.0001*
BNP	Height	-0.2011	<.0001*
BNP	Weight	-0.2297	<.0001*
BNP	BMI	-0.1506	<.0001*
BNP	%Fat	-0.1080	<.0001*
BNP	Waist	-0.1388	<.0001*
BNP	SBP	0.0532	0.0349*
BNP	DBP	-0.0655	0.0094*
BNP	MBP	-0.0150	0.5517
BNP	Pulse	-0.0691	0.0062*
TP	Age	0.0381	0.1316
TP	Male=0/Female=1	0.1078	<.0001*
TP	Height	-0.0819	0.0012*
TP	Weight	0.0061	0.808
TP	BMI	0.0903	0.0003*
TP	%Fat	0.2328	<.0001*
TP	Waist	0.0681	0.0069*

TP	SBP	0.0889	0.0004*
TP	DBP	0.0539	0.0327*
TP	MBP	0.0733	0.0037*
TP	Pulse	0.214	<.0001*
TP	BNP	−0.1329	<.0001*
Alb	Age	−0.1614	<.0001*
Alb	Male=0/Female=1	−0.0045	0.8595
Alb	Height	0.0488	0.0532
Alb	Weight	0.043	0.0885
Alb	BMI	0.0334	0.1859
Alb	%Fat	0.0893	0.0004*
Alb	Waist	0.0041	0.8697
Alb	SBP	0.0741	0.0033*
Alb	DBP	0.0834	0.0009*
Alb	MBP	0.0834	0.0009*
Alb	Pulse	0.1156	<.0001*
Alb	BNP	−0.2321	<.0001*
Alb	TP	0.4998	<.0001*
A/G	Age	−0.2117	<.0001*
A/G	Male=0/Female=1	−0.1208	<.0001*
A/G	Height	0.1353	<.0001*
A/G	Weight	0.0494	0.0506
A/G	BMI	−0.0419	0.097
A/G	%Fat	−0.1390	<.0001*
A/G	Waist	−0.0575	0.0227*
A/G	SBP	−0.0115	0.6498
A/G	DBP	0.0313	0.2146
A/G	MBP	0.013	0.6076
A/G	Pulse	−0.1107	<.0001*
A/G	BNP	−0.0912	0.0003*
A/G	TP	−0.5227	<.0001*
A/G	Alb	0.4048	<.0001*
TBil	Age	−0.0072	0.7766
TBil	Male=0/Female=1	−0.1539	<.0001*
TBil	Height	0.1803	<.0001*
TBil	Weight	0.1208	<.0001*
TBil	BMI	0.0229	0.3653
TBil	%Fat	−0.1002	<.0001*

TBil	Waist	0.0263	0.2981
TBil	SBP	0.0515	0.0414*
TBil	DBP	0.0971	0.0001*
TBil	MBP	0.0815	0.0012*
TBil	Pulse	−0.0639	0.0114*
TBil	BNP	0.0219	0.3868
TBil	TP	0.0292	0.248
TBil	Alb	0.1205	<.0001*
TBil	A/G	0.0918	0.0003*
AST	Age	0.1108	<.0001*
AST	Male=0/Female=1	−0.1569	<.0001*
AST	Height	0.0588	0.0199*
AST	Weight	0.0696	0.0058*
AST	BMI	0.0662	0.0087*
AST	%Fat	−0.0438	0.0832
AST	Waist	0.093	0.0002*
AST	SBP	0.0641	0.0111*
AST	DBP	0.0695	0.0059*
AST	MBP	0.0736	0.0035*
AST	Pulse	0.0737	0.0035*
AST	BNP	−0.0014	0.9552
AST	TP	0.1814	<.0001*
AST	Alb	0.1047	<.0001*
AST	A/G	−0.0919	0.0003*
AST	TBil	0.0456	0.0709
ALT	Age	−0.1223	<.0001*
ALT	Male=0/Female=1	−0.2342	<.0001*
ALT	Height	0.1911	<.0001*
ALT	Weight	0.3225	<.0001*
ALT	BMI	0.3081	<.0001*
ALT	%Fat	0.1097	<.0001*
ALT	Waist	0.3038	<.0001*
ALT	SBP	0.0872	0.0005*
ALT	DBP	0.1271	<.0001*
ALT	MBP	0.1188	<.0001*
ALT	Pulse	0.0871	0.0005*
ALT	BNP	−0.1819	<.0001*
ALT	TP	0.1556	<.0001*

ALT	Alb	0.1713	<.0001*
ALT	A/G	0.0151	0.5513
ALT	TBil	0.0682	0.0069*
ALT	AST	0.7106	<.0001*
γGTP	Age	−0.0968	0.0001*
γGTP	Male=0/Female=1	−0.4853	<.0001*
γGTP	Height	0.3772	<.0001*
γGTP	Weight	0.4311	<.0001*
γGTP	BMI	0.3043	<.0001*
γGTP	%Fat	−0.0482	0.0564
γGTP	Waist	0.3393	<.0001*
γGTP	SBP	0.1813	<.0001*
γGTP	DBP	0.2356	<.0001*
γGTP	MBP	0.2272	<.0001*
γGTP	Pulse	0.005	0.8434
γGTP	BNP	−0.1509	<.0001*
γGTP	TP	0.0494	0.0504
γGTP	Alb	0.0741	0.0033*
γGTP	A/G	0.0292	0.2482
γGTP	TBil	0.0697	0.0058*
γGTP	AST	0.3914	<.0001*
γGTP	ALT	0.556	<.0001*
ALP	Age	0.0855	0.0007*
ALP	Male=0/Female=1	0.1147	<.0001*
ALP	Height	−0.1370	<.0001*
ALP	Weight	−0.0795	0.0016*
ALP	BMI	0.0089	0.7241
ALP	%Fat	0.1177	<.0001*
ALP	Waist	0.0329	0.193
ALP	SBP	0.0638	0.0115*
ALP	DBP	0.0451	0.0743
ALP	MBP	0.0559	0.0269*
ALP	Pulse	0.1341	<.0001*
ALP	BNP	0.0079	0.7533
ALP	TP	0.142	<.0001*
ALP	Alb	0.0238	0.3466
ALP	A/G	−0.1124	<.0001*
ALP	TBil	−0.0637	0.0116*

ALP	AST	0.0716	0.0045*
ALP	ALT	0.1091	<.0001*
ALP	γGTP	0.0668	0.0081*
TCho	Age	−0.0921	0.0003*
TCho	Male=0/Female=1	0.2354	<.0001*
TCho	Height	−0.1456	<.0001*
TCho	Weight	−0.0960	0.0001*
TCho	BMI	−0.0149	0.5562
TCho	%Fat	0.1882	<.0001*
TCho	Waist	−0.0210	0.4064
TCho	SBP	−0.0402	0.1114
TCho	DBP	−0.0216	0.3925
TCho	MBP	−0.0286	0.257
TCho	Pulse	0.0666	0.0083*
TCho	BNP	−0.0989	<.0001*
TCho	TP	0.1181	<.0001*
TCho	Alb	0.1536	<.0001*
TCho	A/G	0.0128	0.6112
TCho	TBil	0.0416	0.0994
TCho	AST	−0.0217	0.3896
TCho	ALT	−0.0040	0.8743
TCho	γGTP	−0.0367	0.1467
TCho	ALP	0.0442	0.0803
TG	Age	−0.0724	0.0041*
TG	Male=0/Female=1	−0.1955	<.0001*
TG	Height	0.1729	<.0001*
TG	Weight	0.3451	<.0001*
TG	BMI	0.3481	<.0001*
TG	%Fat	0.1966	<.0001*
TG	Waist	0.3652	<.0001*
TG	SBP	0.195	<.0001*
TG	DBP	0.2169	<.0001*
TG	MBP	0.2211	<.0001*
TG	Pulse	0.0812	0.0013*
TG	BNP	−0.1991	<.0001*
TG	TP	0.1186	<.0001*
TG	Alb	0.0969	0.0001*
TG	A/G	−0.0285	0.2596

TG	TBil	−0.0623	0.0135*
TG	AST	0.0711	0.0048*
TG	ALT	0.2487	<.0001*
TG	γGTP	0.3373	<.0001*
TG	ALP	0.0828	0.0010*
TG	TCho	0.1635	<.0001*
HDL	Age	0.0297	0.2389
HDL	Male=0/Female=1	0.2664	<.0001*
HDL	Height	−0.2251	<.0001*
HDL	Weight	−0.3883	<.0001*
HDL	BMI	−0.3569	<.0001*
HDL	%Fat	−0.1374	<.0001*
HDL	Waist	−0.3778	<.0001*
HDL	SBP	−0.1092	<.0001*
HDL	DBP	−0.1349	<.0001*
HDL	MBP	−0.1290	<.0001*
HDL	Pulse	0.0169	0.5024
HDL	BNP	0.137	<.0001*
HDL	TP	−0.0644	0.0107*
HDL	Alb	0.0298	0.2382
HDL	A/G	0.0831	0.0010*
HDL	TBil	0.0968	0.0001*
HDL	AST	0.0394	0.1187
HDL	ALT	−0.1606	<.0001*
HDL	γGTP	−0.1418	<.0001*
HDL	ALP	−0.0761	0.0025*
HDL	TCho	0.2595	<.0001*
HDL	TG	−0.5136	<.0001*
LDL	Age	−0.0788	0.0018*
LDL	Male=0/Female=1	0.1717	<.0001*
LDL	Height	−0.1017	<.0001*
LDL	Weight	0.0051	0.8415
LDL	BMI	0.0865	0.0006*
LDL	%Fat	0.2355	<.0001*
LDL	Waist	0.0636	0.0117*
LDL	SBP	−0.0379	0.1338
LDL	DBP	−0.0246	0.33
LDL	MBP	−0.0317	0.2091

LDL	Pulse	0.0159	0.5294
LDL	BNP	−0.1109	<.0001*
LDL	TP	0.1215	<.0001*
LDL	Alb	0.13	<.0001*
LDL	A/G	−0.0111	0.6592
LDL	TBil	0.0375	0.1373
LDL	AST	−0.1060	<.0001*
LDL	ALT	−0.0237	0.3472
LDL	γGTP	−0.0946	0.0002*
LDL	ALP	0.046	0.0684
LDL	TCho	0.8233	<.0001*
LDL	TG	0.1179	<.0001*
LDL	HDL	−0.0731	0.0038*
HbA1c	Age	0.1694	<.0001*
HbA1c	Male=0/Female=1	−0.0089	0.7257
HbA1c	Height	−0.0429	0.0897
HbA1c	Weight	0.0624	0.0134*
HbA1c	BMI	0.1162	<.0001*
HbA1c	%Fat	0.0972	0.0001*
HbA1c	Waist	0.1711	<.0001*
HbA1c	SBP	0.0709	0.0049*
HbA1c	DBP	0.0046	0.8553
HbA1c	MBP	0.034	0.1782
HbA1c	Pulse	0.0454	0.0722
HbA1c	BNP	0.0065	0.7977
HbA1c	TP	0.0537	0.0333*
HbA1c	Alb	−0.0195	0.4395
HbA1c	A/G	−0.0827	0.0010*
HbA1c	TBil	−0.1773	<.0001*
HbA1c	AST	0.0469	0.0632
HbA1c	ALT	0.111	<.0001*
HbA1c	γGTP	0.0799	0.0015*
HbA1c	ALP	0.1148	<.0001*
HbA1c	TCho	0.0381	0.1319
HbA1c	TG	0.1686	<.0001*
HbA1c	HDL	−0.1452	<.0001*
HbA1c	LDL	0.0476	0.0595
WBC	Age	−0.0628	0.0128*

WBC	Male=0/Female=1	−0.2137	<.0001*
WBC	Height	0.1354	<.0001*
WBC	Weight	0.2434	<.0001*
WBC	BMI	0.2311	<.0001*
WBC	%Fat	0.1126	<.0001*
WBC	Waist	0.245	<.0001*
WBC	SBP	0.1226	<.0001*
WBC	DBP	0.1361	<.0001*
WBC	MBP	0.1398	<.0001*
WBC	Pulse	0.0465	0.0657
WBC	BNP	−0.0934	0.0002*
WBC	TP	0.0745	0.0032*
WBC	Alb	−0.0025	0.9219
WBC	A/G	−0.0787	0.0018*
WBC	TBil	−0.0901	0.0004*
WBC	AST	0.0144	0.5676
WBC	ALT	0.1444	<.0001*
WBC	γGTP	0.2164	<.0001*
WBC	ALP	0.0878	0.0005*
WBC	TCho	−0.0327	0.195
WBC	TG	0.3127	<.0001*
WBC	HDL	−0.2823	<.0001*
WBC	LDL	0.0442	0.0801
WBC	HbA1c	0.1936	<.0001*
RBC	Age	−0.2372	<.0001*
RBC	Male=0/Female=1	−0.4208	<.0001*
RBC	Height	0.406	<.0001*
RBC	Weight	0.457	<.0001*
RBC	BMI	0.2994	<.0001*
RBC	%Fat	0.0438	0.0826
RBC	Waist	0.3431	<.0001*
RBC	SBP	0.1649	<.0001*
RBC	DBP	0.2867	<.0001*
RBC	MBP	0.2492	<.0001*
RBC	Pulse	−0.0050	0.842
RBC	BNP	−0.2571	<.0001*
RBC	TP	0.1194	<.0001*
RBC	Alb	0.2183	<.0001*

RBC	A/G	0.0892	0.0004*
RBC	TBil	0.1467	<.0001*
RBC	AST	0.0698	0.0057*
RBC	ALT	0.2826	<.0001*
RBC	γGTP	0.2925	<.0001*
RBC	ALP	0.0948	0.0002*
RBC	TCho	0.0108	0.6686
RBC	TG	0.2856	<.0001*
RBC	HDL	−0.2543	<.0001*
RBC	LDL	0.0605	0.0166*
RBC	HbA1c	0.0877	0.0005*
RBC	WBC	0.2978	<.0001*
Hb	Age	−0.1826	<.0001*
Hb	Male=0/Female=1	−0.6287	<.0001*
Hb	Height	0.5463	<.0001*
Hb	Weight	0.5461	<.0001*
Hb	BMI	0.3094	<.0001*
Hb	%Fat	−0.1063	<.0001*
Hb	Waist	0.3757	<.0001*
Hb	SBP	0.205	<.0001*
Hb	DBP	0.3062	<.0001*
Hb	MBP	0.2793	<.0001*
Hb	Pulse	−0.0646	0.0105*
Hb	BNP	−0.2702	<.0001*
Hb	TP	0.0788	0.0018*
Hb	Alb	0.2242	<.0001*
Hb	A/G	0.1434	<.0001*
Hb	TBil	0.2232	<.0001*
Hb	AST	0.1858	<.0001*
Hb	ALT	0.3639	<.0001*
Hb	γGTP	0.449	<.0001*
Hb	ALP	0.0296	0.2414
Hb	TCho	−0.0266	0.2931
Hb	TG	0.2916	<.0001*
Hb	HDL	−0.2271	<.0001*
Hb	LDL	−0.0031	0.9034
Hb	HbA1c	0.0142	0.574
Hb	WBC	0.2769	<.0001*

Hb	RBC	0.7969	<.0001*
Ht	Age	−0.1688	<.0001*
Ht	Male=0/Female=1	−0.5844	<.0001*
Ht	Height	0.5128	<.0001*
Ht	Weight	0.5094	<.0001*
Ht	BMI	0.2858	<.0001*
Ht	%Fat	−0.0850	0.0008*
Ht	Waist	0.3564	<.0001*
Ht	SBP	0.2125	<.0001*
Ht	DBP	0.3114	<.0001*
Ht	MBP	0.2866	<.0001*
Ht	Pulse	−0.0491	0.052
Ht	BNP	−0.2556	<.0001*
Ht	TP	0.0822	0.0011*
Ht	Alb	0.2036	<.0001*
Ht	A/G	0.1162	<.0001*
Ht	TBil	0.202	<.0001*
Ht	AST	0.1706	<.0001*
Ht	ALT	0.3359	<.0001*
Ht	γGTP	0.4085	<.0001*
Ht	ALP	0.0542	0.0319*
Ht	TCho	−0.0099	0.6945
Ht	TG	0.2826	<.0001*
Ht	HDL	−0.2114	<.0001*
Ht	LDL	0.0164	0.515
Ht	HbA1c	0.0477	0.0587
Ht	WBC	0.2983	<.0001*
Ht	RBC	0.8465	<.0001*
Ht	Hb	0.9552	<.0001*
Plt	Age	−0.1683	<.0001*
Plt	Male=0/Female=1	0.1233	<.0001*
Plt	Height	−0.0925	0.0002*
Plt	Weight	−0.0115	0.65
Plt	BMI	0.0513	0.0423*
Plt	%Fat	0.1785	<.0001*
Plt	Waist	−0.0097	0.7022
Plt	SBP	−0.0372	0.1407
Plt	DBP	−0.0342	0.1752

Plt	MBP	−0.0364	0.1497
Plt	Pulse	0.0393	0.1198
Plt	BNP	−0.1631	<.0001*
Plt	TP	0.0717	0.0045*
Plt	Alb	−0.0090	0.7231
Plt	A/G	−0.0801	0.0015*
Plt	TBil	−0.1823	<.0001*
Plt	AST	−0.1174	<.0001*
Plt	ALT	−0.0532	0.0353*
Plt	γGTP	0.0148	0.5588
Plt	ALP	0.0571	0.0238*
Plt	TCho	0.1373	<.0001*
Plt	TG	0.1092	<.0001*
Plt	HDL	−0.0180	0.4766
Plt	LDL	0.1258	<.0001*
Plt	HbA1c	0.0363	0.1506
Plt	WBC	0.2529	<.0001*
Plt	RBC	−0.0491	0.0517
Plt	Hb	−0.1246	<.0001*
Plt	Ht	−0.1253	<.0001*
Fib	Age	0.1543	<.0001*
Fib	Male=0/Female=1	0.122	<.0001*
Fib	Height	−0.1595	<.0001*
Fib	Weight	−0.1098	<.0001*
Fib	BMI	−0.0269	0.287
Fib	%Fat	0.0869	0.0006*
Fib	Waist	−0.0510	0.0435*
Fib	SBP	0.0376	0.1362
Fib	DBP	−0.0186	0.4611
Fib	MBP	0.0018	0.9418
Fib	Pulse	0.0902	0.0003*
Fib	BNP	0.0464	0.0659
Fib	TP	0.1151	<.0001*
Fib	Alb	−0.0769	0.0023*
Fib	A/G	−0.1934	<.0001*
Fib	TBil	−0.1732	<.0001*
Fib	AST	−0.0490	0.0525
Fib	ALT	−0.1200	<.0001*

Fib	γGTP	−0.1243	<.0001*
Fib	ALP	0.1643	<.0001*
Fib	TCho	0.0372	0.1403
Fib	TG	0.0009	0.9711
Fib	HDL	−0.0647	0.0104*
Fib	LDL	0.1014	<.0001*
Fib	HbA1c	0.1062	<.0001*
Fib	WBC	0.1805	<.0001*
Fib	RBC	−0.1197	<.0001*
Fib	Hb	−0.1228	<.0001*
Fib	Ht	−0.0910	0.0003*
Fib	Plt	0.1669	<.0001*
BUN	Age	0.1669	<.0001*
BUN	Male=0/Female=1	−0.0723	0.0042*
BUN	Height	0.0049	0.8448
BUN	Weight	−0.0106	0.6759
BUN	BMI	−0.0187	0.4583
BUN	%Fat	−0.0529	0.0362*
BUN	Waist	0.0054	0.8317
BUN	SBP	0.0619	0.0142*
BUN	DBP	0.0329	0.1924
BUN	MBP	0.0476	0.0594
BUN	Pulse	−0.0284	0.2609
BUN	BNP	0.0489	0.0529
BUN	TP	−0.0128	0.6118
BUN	Alb	−0.0429	0.0893
BUN	A/G	−0.0099	0.6945
BUN	TBil	0.0059	0.8164
BUN	AST	0.0339	0.1798
BUN	ALT	0.014	0.5801
BUN	γGTP	−0.0264	0.2963
BUN	ALP	−0.0511	0.0431*
BUN	TCho	−0.0426	0.0916
BUN	TG	−0.0840	0.0009*
BUN	HDL	−0.0089	0.726
BUN	LDL	−0.0197	0.4354
BUN	HbA1c	0.0908	0.0003*
BUN	WBC	0.015	0.5516

BUN	RBC	−0.0337	0.1824
BUN	Hb	−0.0491	0.0517
BUN	Ht	−0.0490	0.0525
BUN	Plt	−0.1116	<.0001*
BUN	Fib	0.0014	0.9565
Crea	Age	−0.0296	0.242
Crea	Male=0/Female=1	−0.7243	<.0001*
Crea	Height	0.5888	<.0001*
Crea	Weight	0.4666	<.0001*
Crea	BMI	0.1617	<.0001*
Crea	%Fat	−0.3686	<.0001*
Crea	Waist	0.2207	<.0001*
Crea	SBP	0.1494	<.0001*
Crea	DBP	0.215	<.0001*
Crea	MBP	0.2001	<.0001*
Crea	Pulse	−0.1784	<.0001*
Crea	BNP	−0.0960	0.0001*
Crea	TP	−0.0721	0.0043*
Crea	Alb	−0.0058	0.819
Crea	A/G	0.0777	0.0021*
Crea	TBil	0.135	<.0001*
Crea	AST	0.0964	0.0001*
Crea	ALT	0.1492	<.0001*
Crea	γGTP	0.3266	<.0001*
Crea	ALP	−0.1439	<.0001*
Crea	TCho	−0.1621	<.0001*
Crea	TG	0.1795	<.0001*
Crea	HDL	−0.2449	<.0001*
Crea	LDL	−0.0923	0.0003*
Crea	HbA1c	0.0036	0.8861
Crea	WBC	0.2038	<.0001*
Crea	RBC	0.3184	<.0001*
Crea	Hb	0.4507	<.0001*
Crea	Ht	0.4216	<.0001*
Crea	Plt	−0.1282	<.0001*
Crea	Fib	−0.0612	0.0153*
Crea	BUN	0.197	<.0001*
Na	Age	−0.0546	0.0307*

Na	Male=0/Female=1	0.1422	<.0001*
Na	Height	-0.1311	<.0001*
Na	Weight	-0.0875	0.0005*
Na	BMI	0.0025	0.9211
Na	%Fat	0.0587	0.0201*
Na	Waist	-0.1105	<.0001*
Na	SBP	-0.0192	0.4483
Na	DBP	-0.0823	0.0011*
Na	MBP	-0.0605	0.0166*
Na	Pulse	-0.0386	0.1267
Na	BNP	-0.0249	0.324
Na	TP	-0.1081	<.0001*
Na	Alb	0.0638	0.0114*
Na	A/G	0.1741	<.0001*
Na	TBil	-0.0278	0.2711
Na	AST	-0.0687	0.0065*
Na	ALT	-0.0681	0.0070*
Na	γGTP	-0.1029	<.0001*
Na	ALP	-0.0039	0.8764
Na	TCho	0.0201	0.4265
Na	TG	-0.0472	0.0614
Na	HDL	0.0393	0.1194
Na	LDL	0.0643	0.0108*
Na	HbA1c	-0.1037	<.0001*
Na	WBC	-0.0909	0.0003*
Na	RBC	-0.0774	0.0021*
Na	Hb	-0.0394	0.1191
Na	Ht	-0.0376	0.1365
Na	Plt	-0.0025	0.9201
Na	Fib	0.0678	0.0072*
Na	BUN	-0.0239	0.3433
Na	Crea	-0.0501	0.0472*
K	Age	0.049	0.0522
K	Male=0/Female=1	-0.1553	<.0001*
K	Height	0.1119	<.0001*
K	Weight	0.0772	0.0022*
K	BMI	0.0261	0.3011
K	%Fat	-0.0706	0.0051*

K	Waist	0.0266	0.292
K	SBP	0.0076	0.7649
K	DBP	−0.0028	0.911
K	MBP	0.0021	0.9332
K	Pulse	−0.0578	0.0221*
K	BNP	−0.0225	0.3729
K	TP	−0.0083	0.7437
K	Alb	0.0223	0.3777
K	A/G	0.0428	0.0905
K	TBil	−0.0694	0.0060*
K	AST	0.0728	0.0039*
K	ALT	0.0263	0.2974
K	γGTP	0.0658	0.0092*
K	ALP	−0.0213	0.4
K	TCho	−0.0683	0.0068*
K	TG	0.052	0.0393*
K	HDL	−0.0457	0.07
K	LDL	−0.0435	0.0848
K	HbA1c	0.0955	0.0002*
K	WBC	0.087	0.0006*
K	RBC	0.0467	0.0641
K	Hb	0.1035	<.0001*
K	Ht	0.1017	<.0001*
K	Plt	0.0925	0.0002*
K	Fib	0.0789	0.0018*
K	BUN	0.1378	<.0001*
K	Crea	0.1772	<.0001*
K	Na	−0.0059	0.8153
Cl	Age	−0.1177	<.0001*
Cl	Male=0/Female=1	0.0554	0.0282*
Cl	Height	−0.0322	0.203
Cl	Weight	0.0372	0.1408
Cl	BMI	0.0929	0.0002*
Cl	%Fat	0.0368	0.1456
Cl	Waist	−0.0101	0.6891
Cl	SBP	−0.0840	0.0009*
Cl	DBP	−0.0926	0.0002*
Cl	MBP	−0.0942	0.0002*

Cl	Pulse	−0.1331	<.0001*
Cl	BNP	0.036	0.154
Cl	TP	−0.2947	<.0001*
Cl	Alb	−0.1521	<.0001*
Cl	A/G	0.1516	<.0001*
Cl	TBil	−0.0450	0.0747
Cl	AST	−0.1603	<.0001*
Cl	ALT	−0.0972	0.0001*
Cl	γGTP	−0.0809	0.0013*
Cl	ALP	−0.1193	<.0001*
Cl	TCho	−0.0727	0.0040*
Cl	TG	−0.0435	0.085
Cl	HDL	−0.0772	0.0022*
Cl	LDL	0.0326	0.1965
Cl	HbA1c	−0.1413	<.0001*
Cl	WBC	−0.0566	0.0249*
Cl	RBC	−0.1533	<.0001*
Cl	Hb	−0.1415	<.0001*
Cl	Ht	−0.1693	<.0001*
Cl	Plt	0.0312	0.2168
Cl	Fib	−0.0583	0.0210*
Cl	BUN	0.0144	0.5699
Cl	Crea	0.0407	0.1067
Cl	Na	0.5363	<.0001*
Cl	K	0.1074	<.0001*
Ca	Age	−0.0961	0.0001*
Ca	Male=0/Female=1	0.1373	<.0001*
Ca	Height	−0.0547	0.0302*
Ca	Weight	−0.0160	0.5269
Ca	BMI	0.025	0.3231
Ca	%Fat	0.1784	<.0001*
Ca	Waist	0.0313	0.2154
Ca	SBP	0.0282	0.2642
Ca	DBP	0.0364	0.1491
Ca	MBP	0.0338	0.1802
Ca	Pulse	0.0919	0.0003*
Ca	BNP	−0.1521	<.0001*
Ca	TP	0.4304	<.0001*

Ca	Alb	0.5069	<.0001*
Ca	A/G	0.0448	0.0762
Ca	TBil	0.0563	0.0256*
Ca	AST	0.0418	0.0975
Ca	ALT	0.0799	0.0015*
Ca	γGTP	0.0063	0.8021
Ca	ALP	0.0667	0.0082*
Ca	TCho	0.2129	<.0001*
Ca	TG	0.09	0.0004*
Ca	HDL	0.0507	0.0445*
Ca	LDL	0.1658	<.0001*
Ca	HbA1c	0.0728	0.0039*
Ca	WBC	0.0806	0.0014*
Ca	RBC	0.1222	<.0001*
Ca	Hb	0.1142	<.0001*
Ca	Ht	0.106	<.0001*
Ca	Plt	0.1247	<.0001*
Ca	Fib	0.098	0.0001*
Ca	BUN	−0.0020	0.9361
Ca	Crea	−0.0260	0.3028
Ca	Na	0.0133	0.5994
Ca	K	0.1824	<.0001*
Ca	Cl	−0.1907	<.0001*
UA	Age	−0.0723	0.0042*
UA	Male=0/Female=1	−0.5344	<.0001*
UA	Height	0.4506	<.0001*
UA	Weight	0.4892	<.0001*
UA	BMI	0.3044	<.0001*
UA	%Fat	−0.1107	<.0001*
UA	Waist	0.3424	<.0001*
UA	SBP	0.1677	<.0001*
UA	DBP	0.2189	<.0001*
UA	MBP	0.2093	<.0001*
UA	Pulse	−0.1050	<.0001*
UA	BNP	−0.1114	<.0001*
UA	TP	0.0467	0.0644
UA	Alb	0.0285	0.2599
UA	A/G	−0.0135	0.594

UA	TBil	0.0551	0.0290*
UA	AST	0.1641	<.0001*
UA	ALT	0.2525	<.0001*
UA	γGTP	0.4053	<.0001*
UA	ALP	−0.1223	<.0001*
UA	TCho	−0.1216	<.0001*
UA	TG	0.2386	<.0001*
UA	HDL	−0.2733	<.0001*
UA	LDL	−0.0648	0.0102*
UA	HbA1c	0.0277	0.2722
UA	WBC	0.2359	<.0001*
UA	RBC	0.3026	<.0001*
UA	Hb	0.3924	<.0001*
UA	Ht	0.3688	<.0001*
UA	Plt	−0.0436	0.0845
UA	Fib	−0.0277	0.272
UA	BUN	0.1419	<.0001*
UA	Crea	0.5719	<.0001*
UA	Na	−0.0535	0.0342*
UA	K	0.147	<.0001*
UA	Cl	0.0326	0.1966
UA	Ca	0.0663	0.0086*
Amy	Age	0.1806	<.0001*
Amy	Male=0/Female=1	0.0393	0.1195
Amy	Height	−0.1133	<.0001*
Amy	Weight	−0.2444	<.0001*
Amy	BMI	−0.2485	<.0001*
Amy	%Fat	−0.1641	<.0001*
Amy	Waist	−0.2232	<.0001*
Amy	SBP	−0.0826	0.0011*
Amy	DBP	−0.0954	0.0002*
Amy	MBP	−0.0937	0.0002*
Amy	Pulse	0.0306	0.2255
Amy	BNP	0.0868	0.0006*
Amy	TP	0.0348	0.1677
Amy	Alb	−0.0712	0.0048*
Amy	A/G	−0.0845	0.0008*
Amy	TBil	−0.0117	0.6434

Amy	AST	0.0418	0.0983
Amy	ALT	−0.0892	0.0004*
Amy	γGTP	−0.1564	<.0001*
Amy	ALP	0.0255	0.3124
Amy	TCho	0.0112	0.6568
Amy	TG	−0.1464	<.0001*
Amy	HDL	0.137	<.0001*
Amy	LDL	−0.0072	0.7769
Amy	HbA1c	−0.0666	0.0083*
Amy	WBC	−0.1269	<.0001*
Amy	RBC	−0.0868	0.0006*
Amy	Hb	−0.1059	<.0001*
Amy	Ht	−0.0832	0.0010*
Amy	Plt	−0.0254	0.3148
Amy	Fib	0.057	0.0239*
Amy	BUN	0.0744	0.0032*
Amy	Crea	0.0757	0.0027*
Amy	Na	−0.0193	0.4441
Amy	K	0.028	0.2676
Amy	Cl	−0.1149	<.0001*
Amy	Ca	0	1
Amy	UA	−0.1030	<.0001*
IMT_R	Age	0.3642	<.0001*
IMT_R	Male=0/Female=1	−0.0528	0.0364*
IMT_R	Height	−0.0334	0.1862
IMT_R	Weight	0.0104	0.6794
IMT_R	BMI	0.0415	0.1001
IMT_R	%Fat	−0.0332	0.1887
IMT_R	Waist	0.1502	<.0001*
IMT_R	SBP	0.1973	<.0001*
IMT_R	DBP	0.0989	<.0001*
IMT_R	MBP	0.1474	<.0001*
IMT_R	Pulse	0.0144	0.5692
IMT_R	BNP	0.1249	<.0001*
IMT_R	TP	0.0554	0.0281*
IMT_R	Alb	−0.0726	0.0040*
IMT_R	A/G	−0.1375	<.0001*
IMT_R	TBil	−0.0306	0.2255

IMT_R	AST	0.033	0.1918
IMT_R	ALT	-0.0113	0.6534
IMT_R	γGTP	-0.0139	0.5836
IMT_R	ALP	0.0708	0.0050*
IMT_R	TCho	-0.0217	0.3914
IMT_R	TG	0.0192	0.4463
IMT_R	HDL	-0.0469	0.0631
IMT_R	LDL	-0.0194	0.4423
IMT_R	HbA1c	0.1594	<.0001*
IMT_R	WBC	0.0262	0.3
IMT_R	RBC	0.0177	0.4846
IMT_R	Hb	0.0412	0.1026
IMT_R	Ht	0.0384	0.1281
IMT_R	Plt	-0.0256	0.3107
IMT_R	Fib	0.0267	0.2899
IMT_R	BUN	0.1077	<.0001*
IMT_R	Crea	0.0363	0.1504
IMT_R	Na	-0.0743	0.0032*
IMT_R	K	0.0353	0.1627
IMT_R	Cl	-0.1430	<.0001*
IMT_R	Ca	0.0157	0.5333
IMT_R	UA	0.0265	0.2939
IMT_R	Amy	0.0717	0.0045*
IMT_L	Age	0.4187	<.0001*
IMT_L	Male=0/Female=1	-0.0234	0.3545
IMT_L	Height	-0.0719	0.0044*
IMT_L	Weight	-0.0096	0.7035
IMT_L	BMI	0.0423	0.0939
IMT_L	%Fat	-0.0132	0.6016
IMT_L	Waist	0.147	<.0001*
IMT_L	SBP	0.236	<.0001*
IMT_L	DBP	0.1256	<.0001*
IMT_L	MBP	0.1808	<.0001*
IMT_L	Pulse	0.0277	0.2735
IMT_L	BNP	0.1434	<.0001*
IMT_L	TP	0.0449	0.0757
IMT_L	Alb	-0.0797	0.0016*
IMT_L	A/G	-0.1375	<.0001*

IMT_L	TBil	−0.0347	0.1694
IMT_L	AST	0.0377	0.1351
IMT_L	ALT	0.0139	0.5835
IMT_L	γGTP	0.0206	0.4145
IMT_L	ALP	0.0851	0.0007*
IMT_L	TCho	−0.0396	0.1165
IMT_L	TG	0.0501	0.0471*
IMT_L	HDL	−0.0932	0.0002*
IMT_L	LDL	−0.0124	0.6222
IMT_L	HbA1c	0.1882	<.0001*
IMT_L	WBC	0.0507	0.0449*
IMT_L	RBC	−0.0282	0.2647
IMT_L	Hb	0.001	0.9691
IMT_L	Ht	0.0062	0.8072
IMT_L	Plt	−0.0540	0.0325*
IMT_L	Fib	0.0639	0.0113*
IMT_L	BUN	0.102	<.0001*
IMT_L	Crea	0.0111	0.6593
IMT_L	Na	−0.0962	0.0001*
IMT_L	K	0.0437	0.0834
IMT_L	Cl	−0.1511	<.0001*
IMT_L	Ca	0.0139	0.5829
IMT_L	UA	0.0058	0.8178
IMT_L	Amy	0.0155	0.5406
IMT_L	IMT_R	0.5943	<.0001*
